# Overexpressed HSF1 cancer signature genes cluster in human chromosome 8q

**DOI:** 10.1186/s40246-017-0131-5

**Published:** 2017-12-21

**Authors:** Christopher Q. Zhang, Heinric Williams, Thomas L. Prince, Eric S. Ho

**Affiliations:** 1 Department of Biology, Lafayette College, Easton, PA 18042 USA; 2 Department of Computer Science, Lafayette College, Easton, PA 18042 USA; 3 Urology Department, Geisinger Medical Center, Danville, PA 17822 USA; 4 Weis Research Center, Geisinger Medical Center, Danville, PA 17822 USA

**Keywords:** Heat shock factor 1 (HSF1), HSF1 cancer signature (CanSig), Malignancy, Pre-mRNA 3′ processing, Chromosome 8q, Synteny

## Abstract

**Background:**

HSF1 (heat shock factor 1) is a transcription factor that is found to facilitate malignant cancer development and proliferation. In cancer cells, HSF1 mediates a set of genes distinct from heat shock that contributes to malignancy. This set of genes is known as the HSF1 Cancer Signature genes or simply HSF1-CanSig genes. HSF1-CanSig genes function and operate differently than typical cancer-causing genes, yet it is involved in fundamental oncogenic processes.

**Results:**

By utilizing expression data from 9241 cancer patients, we identified that human chromosome 8q21-24 is a location hotspot for the most frequently overexpressed HSF1-CanSig genes. Intriguingly, the strength of the HSF1 cancer program correlates with the number of overexpressed HSF1-CanSig genes in 8q, illuminating the essential role of HSF1 in mediating gene expression in different cancers. Chromosome 8q21-24 is found under selective pressure in preserving gene order as it exhibits strong synteny among human, mouse, rat, and bovine, although the biological significance remains unknown. Statistical modeling, hierarchical clustering, and gene ontology-based pathway analyses indicate crosstalk between HSF1-mediated responses and pre-mRNA 3′ processing in cancers.

**Conclusions:**

Our results confirm the unique role of chromosome 8q mediated by the master regulator HSF1 in cancer cases. Additionally, this study highlights the connection between cellular processes triggered by HSF1 and pre-mRNA 3′ processing in cancers.

**Electronic supplementary material:**

The online version of this article (10.1186/s40246-017-0131-5) contains supplementary material, which is available to authorized users.

## Introduction

Heat-shock factor 1 (HSF1) is a master transcription factor that initiates the expression of heat shock proteins (HSPs) and other genes in response to cellular stress, thus allowing cells to adapt and prolong survival. Cancer cells, however, hijack this protective mechanism to allow them to continue proliferating in their toxic microenvironments. Several studies have linked increased HSF1 activity to malignant cell growth [1]. This pro-malignant activity is associated with HSF1 binding to the promoters and initiating the expression of certain genes independent of heat shock [1]. Identified by Mendillo, Santagata, and coworkers, the HSF1 Cancer Signature (HSF1-CanSig) is a set of 475 genes overexpressed in a number of highly malignant cancer cells and primary tumors [1].

HSF1 itself is observed to be overexpressed in across different tumor types and to promote proliferation, migration, and invasion [2–5]. Frequently, overexpressed genes across different tumors or cancer types are understood to be likely oncogenic and contribute to tumorigenesis [6]. Consequently, this drives the overexpression of the HSF1-CanSig in tumor cells and possibly the surrounding stroma leading to metastasis and poor clinical outcomes [1, 2]. The biological processes and features that link the HSF1-CanSig to malignant cell growth, however, are still unclear.

To elucidate the role of the HSF1-CanSig in cancer, we mined and analyzed the overexpression in 9241 cancer cases from 27 unique primary tumor sites from The Cancer Genome Atlas (TCGA) hosted in cBioPortal [7]. We found that 27 of the top 100 most frequently overexpressed HSF1-CanSig genes are clustered in a highly syntenic region of chromosome 8q. We observed that this HSF1-driven chromosome 8q gene is set to be overexpressed in a majority of cancer types. Furthermore, our gene ontology analysis indicates a link between HSF1-driven transcription and mRNA processing located on chromosome 8q.

## Results

### Top 100 high-scoring HSF1-CanSig genes are disproportionately located in chromosome 8q

**Fig. 1:**
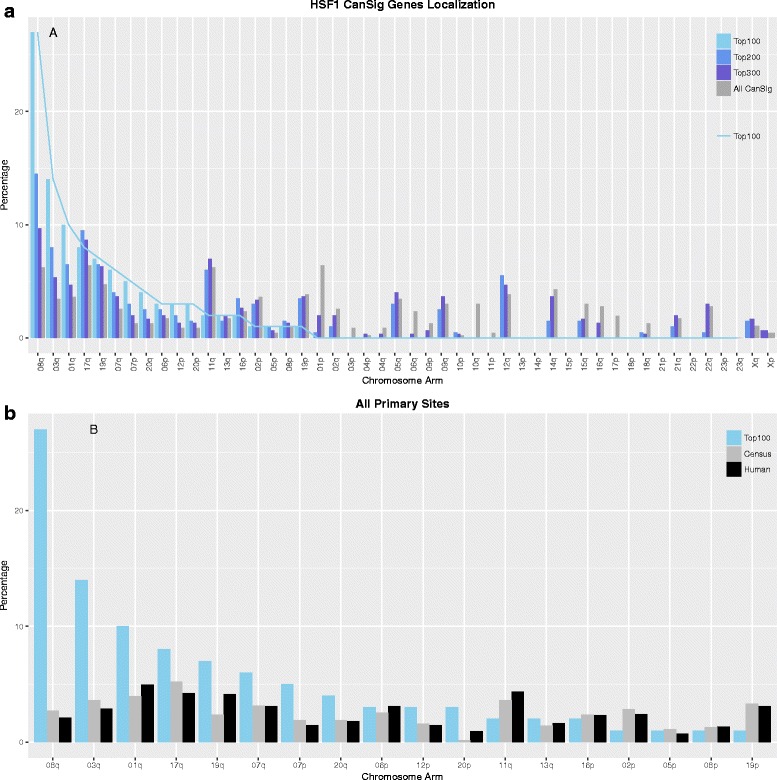
**a** Percentage distribution of HSF1-CanSig genes by chromosome arms from different tiers: top 100, top 200, top 300, and all HSF1-CanSign genes. **b** Percentage distribution of the top 100 high-scoring HSF1-CanSig genes by chromosome arm. Two references were used for comparison: 633 cancer census genes (labeled census) from COSMIC [49] and 19,073 human protein-coding genes (labeled all) based on HuGO [48]

We devised an overexpression score to quantify the prevalence of HSF1-CanSig genes being overexpressed among cancer cases (see the “Materials and methods” section for details). The overexpression score reflects the prevalence of a gene being overexpressed ≥ 2 standard deviations above the control reference as calculated by cBioPortal for different primary tumor sites [8]. A gene associated with a high overexpression score indicates that the gene is often upregulated in cancer cases. Overexpression scores of all 475 HSF1-CanSig genes from different primary tumor sites were aggregated, ranked, and examined by their percentage distribution per chromosome arm in tiers: top 100, 200, 300, and all HSF1-CanSig genes. If ranking has no effect on distribution, the distribution of genes in each tier should resemble the distribution of all HSF1-CanSig genes, meaning that they distribute like the result of random sampling of HSF1-CanSig genes. Intriguingly, 27% of the top 100 groups comprises of genes encoded in chromosome 8q (Fig. 1a, leftmost light blue bar) given that 8q consists of only 6% (29 of 475) of all HSF1-CanSig (leftmost gray bar). Such percentage declines continuously with from the top 200 group to top 300 group.

To assess the statistical significance of the distribution exhibited in Fig. 1a, we tested the probability of observing certain number of genes per chromosome arm in different tiers by hypergeometric test and Fisher’s exact test (Additional file 1: Table S1). The four chromosome arms that encode the highest number of HSF1-CanSig genes are 1p (30), 8q (29), 11q (29), and 17q (30). When genes were examined in tiers by ranking, HSF1-CanSig genes encoded in 8q are included most disproportionately in different tiers but not for genes in the other three chromosome arms, i.e., 1p, 11q, and 17q. The group that attained the smallest *p* value of Fisher’s test was the top 100 group (5.41e^−20^), whereas hypergeometric test found that the top 50 group achieved the lowest *p* value (3.13e^−18^) followed by the top 100 group (1.09e^−17^). But the former captures only 22 8q genes out of 29 versus 27 8q genes by the latter. To balance statistical rigor and coverage, we decided to focus on the top 100 frequently overexpressed HSF1-CanSig genes. Importantly, regardless of which tier we choose, the most striking finding is that a large proportion of top-ranking genes are encoding in chromosome 8q as supported by Additional file 1: Table S1.

Next, we examine whether the skew distribution of 8q genes in the top 100 group is influenced by the intrinsic distribution of cancer or protein-coding genes. To rule out this possibility, the aggregated overexpression scores of the top 100 high-scoring HSF1-CanSig genes were compared with two null models or references: census cancer gene and all protein-coding genes, which consist of only 2–3% of 8q genes (gray and black bars in Fig. 1b, respectively). A high correlation between the two references based on the percentage of genes per chromosome arm was shown (*R* = 0.92, *p* < 5.98e^−8^), suggesting that cancer genes exhibit similar distribution as protein coding genes in the human genome. To ascertain the bias of HSF1-CanSig 8q genes quantitatively, a hypergeometric test was used. The probability of observing 27 or more HSF1-CanSig 8q genes (listed in Table 1) in a sample of 100 genes by chance is 1.02 × 10^−22^.

**Table 1:** Twenty-seven out of the top 100 high-scoring HSF1-CanSig genes are clustered near the end of chromosome 8q

Gene symbol	Name	Location	Rank
PUF60	Poly(U) binding splicing factor 60	08q24.3	1
HSF1	Heat shock transcription factor 1	08q24.3	2
NUDCD1	Nudc domain containing 1	08q23.1	3
MRPL13	Mitochondrial ribosomal protein L13	08q24.12	4
ENY2	ENY2, transcription and export complex 2 subunit	08q23.1	5
CPSF1	Cleavage and polyadenylation specific factor 1	08q24.3	7
SHARPIN	SHANK associated RH domain interactor	08q24.3	8
AZIN1	Antizyme inhibitor 1	08q22.3	9
MAF1	MAF1 homolog, negative regulator of RNA polymerase III	08q24.3	10
PABPC1	Poly(A) binding protein cytoplasmic 1	08q22.3	11
TRAPPC9	Trafficking protein particle complex 9	08q24.3	12
ZNF250	Zinc finger protein 250	08q24.3	13
ZNF34	Zinc finger protein 34	08q24.3	15
NBN	Nibrin	08q21.3	16
JRK	Jrk helix-turn-helix protein	08q24.3	19
DPY19L4	Dpy-19 Like 4 (*C. elegans*)	08q22.1	22
CYHR1	Cysteine and histidine rich 1	08q24.3	23
TPD52	Tumor protein D52	08q21.13	28
PLEC	Plectin	08q24.3	32
MFSD3	Major facilitator superfamily domain containing 3	08q24.3	35
KIFC2	Kinesin family member C2	08q24.3	40
C8ORF37	Chromosome 8 open reading frame 37	08q22.1	49
NDRG1	N-Myc downstream regulated 1	08q24.22	54
SLC45A4	Solute carrier family 45 member 4	08q24.3	56
MROH6	Maestro heat like repeat family member 6	08q24.3	59
LY6K	Lymphocyte antigen 6 family member K	08q24.3	66
LRP12	LDL receptor related protein 12	08q22.3	69

Rank is based on the aggregated overexpression score of all primary tumor sites

### Overexpression of HSF1-CanSig 8q genes is primary tumor site-specific

**Fig. 2:**
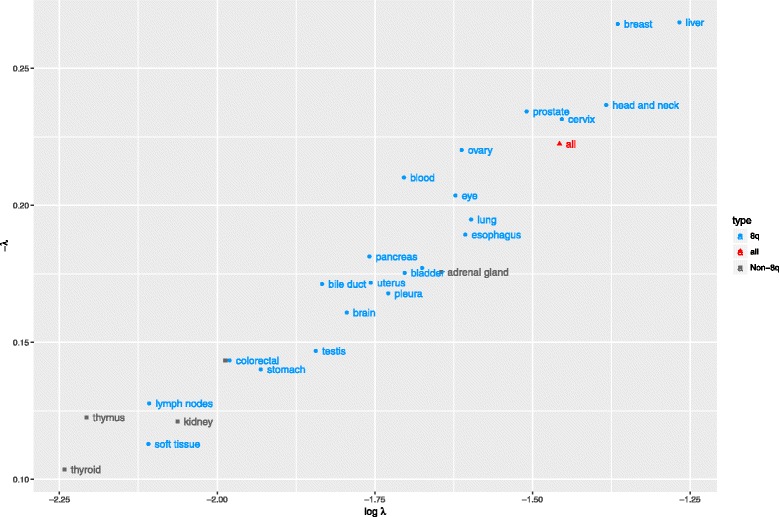
Quantification of 8q skewness. The percentages of the top 10 chromosomes were fitted to an exponential model. The model outputs (*λ* = slope, ln(*λ*) = intercept) are plotted. Primary tumor sites with 8q and without 8q clustering are in blue and gray, respectively. The red triangle represents the fitting of data when 8q genes from all primary sites are ranked together

Furthermore, we examined the distribution of HSF1-CanSig 8q genes by individual primary tumor sites. Skewed distribution of HSF1-CanSig genes on chromosome 8q, similar to that in Fig. 1b, was observed in 22 out of 27 primary sites with a variable degree of 8q bias (plots for individual primary site can be found in Additional file 2). The five primary tumor sites that showed no bias are the adrenal gland, kidney, nervous system, thymus, and thyroid. An exponential distribution (*p*_*i*_ = *λe* ^−*λi*^ or in linear form: ln*p*_*i*_ =  − *λi* + ln *λ*) was used to quantify the extent of skewness: *p*_*i*_ is the probability or proportion of the *i*th-ranked chromosome, *λ* is a coefficient, and *i* is the rank in the range 1 to 10. A large magnitude of *λ* indicates a high degree of skewness and vice versa. The *λ* values of primary sites span a wide spectrum of chromosome 8q bias as shown in Fig. 2, where higher 8q bias are observed in breast and liver cancers than in lymph nodes and soft tissue, for instance. Primary sites lacking 8q bias primary tumor sites tend to be cornered at a region with small *λ* value (labeled in gray). This result reveals that the overexpression of these HSF1-CanSig 8q genes is primary tumor site-specific.

### HSF1-CanSig genes clustered at a 44-Mbp region near the end of chromosome 8q

The 27 HSF1-CanSig 8q genes ranked in the top 100 high-scoring HSF1-CanSig genes are tabulated in Table 1. Remarkably, all HSF1-CanSig 8q genes including HSF1 itself occupy the last three cytogenetic bands of chromosome 8q, i.e., 8q21.3 to 8q.24.3 (~ 44 Mbps). The 3′-most gene ZNF250 is ~ 237 Kbps from the chromosome’s 3′ end with only three genes encoded further downstream.

### Frequently overexpressed HSF1 associates with the overexpression of HSF1-CanSig genes in 8q

Cancer is heterogeneous both within and between cancer types. Hence, we sought to rank individual HSF1-CanSig 8q genes in different primary tumor sites (Table 2). To cover the entire chromosomal segment encoding all 29 HSF1-CanSig 8q genes, two 8q genes, GPT and KLF10, were included even though they were not ranked in the top 100. The one-sample two-sided Student’s *t* test was used to assess the ranking distribution of them (the second column from the right in Table 2) for each primary tumor site where the *p* values of the *t* test are shown in the last column of Table 2. At 95% confidence level, ranks of 8q genes in 20 primary tumor sites (from the ovary to pleura in Table 2) are detected with statistically significant deviation from the mean rank of 233 (see the “Materials and methods” section for the determination of the mean rank). No single primary tumor site had all 29 HSF1-CanSig 8q genes overexpressed in the top 100 HSF1-CanSig genes. Surprisingly there were no primary tumor sites with significant low-average rank (the lowest two are the thymus, *p* = 0.896, and the thyroid, *p* = 0.283, in Table 2), suggesting the absence of a strong suppression among HSF1-CanSig 8q genes in those primary tumor sites.

**Table 2:**
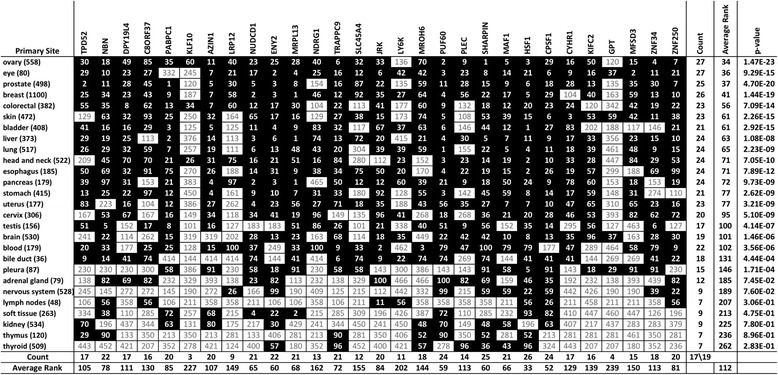
Ranks of HSF1-CanSig 8q genes by primary tumor site. The bracketed number next to the name of a primary site denotes the number of cases recruited in the study. Genes displayed in the table heading are arranged in a syntenic order. The number inside a cell denotes the rank of the gene among HSF1-CanSig genes with the most frequently overexpressed genes ranked 1. Ranks ≤ 100 are highlighted in solid black. Some genes may share the same rank if they have the same overexpression score. Count includes only genes with rank ≤ 100. Average rank is the arithmetic mean of ranks. HSF1-CanSig genes GPT and LRP12 are not part of the top 100 high-scoring genes. *p* value is determined by one-sample two-sided Student’s *t* test. The table is sorted by average rank and count in ascending and descending order, respectively

While no single HSF1-CanSig 8q gene is ranked in the top 100 across all primary sites, HSF1 is frequently overexpressed in most primary tumor sites with kidney as the only exception (26 out of 27, shown at the bottom of HSF1 column in Table 2). HSF1 is also the gene with the highest average rank (33) followed by CPSF1 (52). Additionally, the rank of HSF1 correlates with the average rank of primary sites (*R* = 0.71), but the most correlated 8q gene is CYHR1, not HSF1. Importantly, HSF1 is the only gene in which its rank is always lower than the average rank (Additional file 3: Figure S1).

Lastly, primary tumor sites associated with a small number of high-scoring HSF1-CanSig 8q genes, such as the lymph nodes, soft tissue, thymus, and thyroid, tend to exhibit lower skewness (see Fig. 2). These results suggest a minor role of HSF1-mediated activities in those primary tumor sites.

### Primary tumor sites are segregated by ranks

**Fig. 3:**
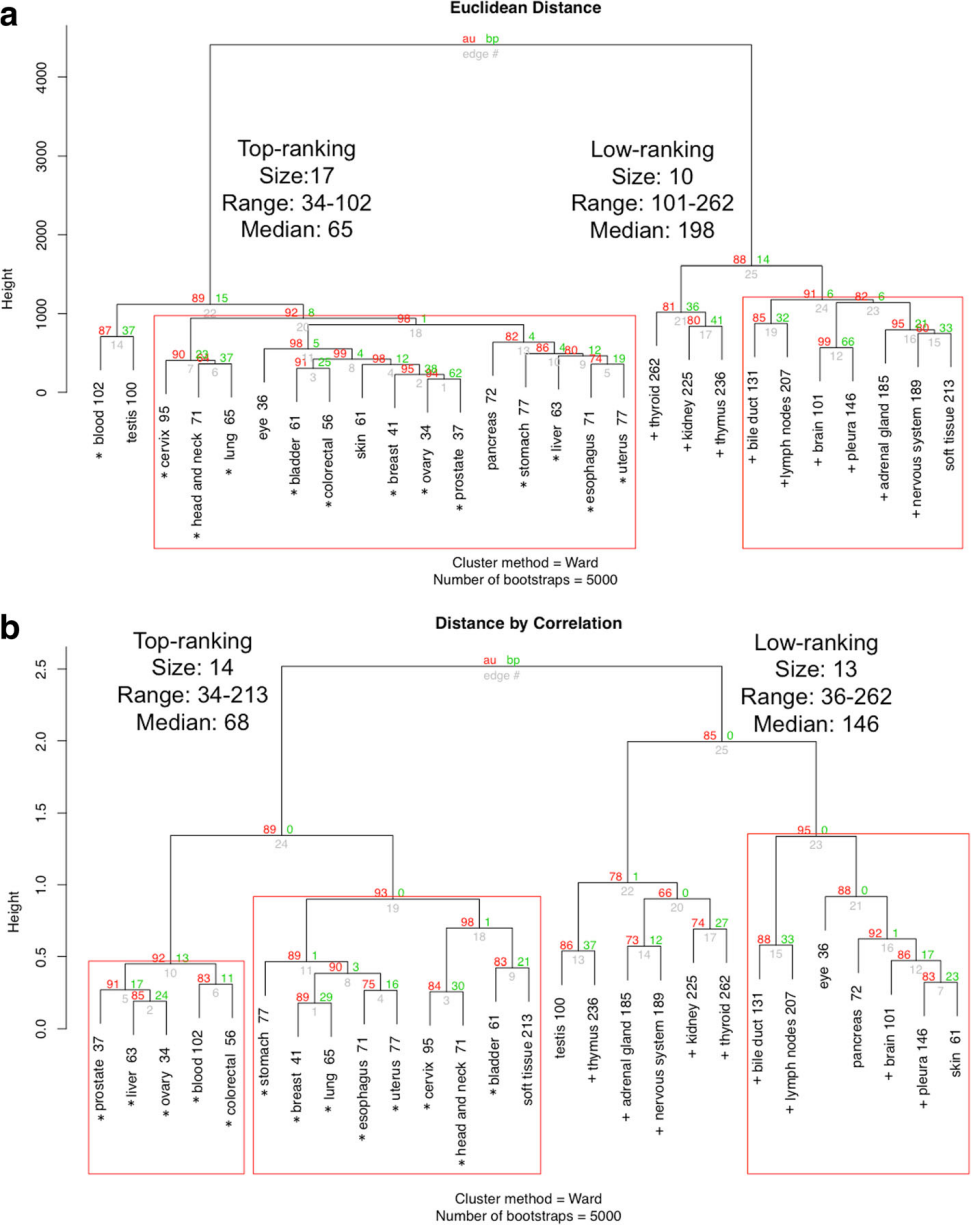
Rank analysis by hierarchical clustering with bootstrapping. The number beside each primary tumor site is the average rank. Subtrees with high bootstrap value (≥ 90) are highlighted in red boxes. Each dendrogram was built after bootstrapping 5000 times. The red and green numbers at the branch point represent approximately unbias (AU) *p* value and bootstrap probability (BP), respectively. AU is recommended as a better parameter than BP to assess the clustering reliability at each branch point [54]. Cluster subtree highlighted by red box indicates AU ≥ 90. For the top-ranking groups, primary tumor sites shared between two distance methods are marked by asterisks. Similarly, for the low-ranking groups, primary sites are marked by plus signs. **a** Cluster dendrogram based on Euclidean distance. **b** Same input data as panel **a** but correlation was used as the distance calculation method

We next asked if the ranks of HSF1-CanSig 8q genes in different primary tumor sites share similar patterns. By using hierarchical clustering with two distinct distance methods, primary sites were clustered using the ranks of HSF1-CanSig 8q genes as shown in Fig. 3. Both panels in Fig. 3 show that primary tumor sites can be reliably split into two groups by the average rank, namely, the top-ranking and the low-ranking groups. The medians of the top-ranking groups produced by the two distance methods reflect higher consistency as they vary within a narrow range of 65–68. But the medians of the low-ranking groups show a larger difference from 148 to 198.

Regarding the range of average rank within each group, if the outlier, i.e., soft tissue with average rank 213, is taken out from the top-ranking correlation-distance group in Fig. 3b, the range of average ranks in both becomes identical, 34 to 102. A cutoff value of 102 seems to divide the two groups. Moreover, top-ranking groups created by the two distance methods share 13 out of 14 to 17 primary sites (marked by asterisks in Fig. 3). The low-ranking groups however display a larger variation in rank medians 146–198, but 9 out of 10 to 13 primary sites are clustered together by both methods (marked by plus signs in Fig. 3). This result suggests the feasibility of stratifying primary tumor sites by the overexpression of HSF1-CanSig 8q genes.

### HSF1-CanSig 8q genes are highly syntenic in mammals

Since the proximity of HSF1-CanSig 8q genes seems to be a contributing factor for their cohesive expression pattern, we asked whether their proximity reveals biological significance. We sought answers in the light of evolution by examining the synteny of the genomic region spanning the 5′- and 3′-most HSF1-CanSig 8q genes, i.e., TPD53 and ZNF250, respectively. It means that other non-HSF1-CanSig genes were also included in the syntenic analysis. This region consists of 267 genes with known genomic coordinates in human where 29 of them are HSF1-CanSig genes. We aligned these human genes with homologs from bovine, mouse, and rat. Their pairwise percentage of synteny can be found in Table 3A, which varies from the lowest 50% (mouse and bovine) to the highest 84% (human and mouse). As our focus is on the HSF1-CanSig genes, we highlighted their genomic order in Table 3B. Syntenic information of all 267 genes can be found in Additional file 1: Table S5.

**Table 3:**
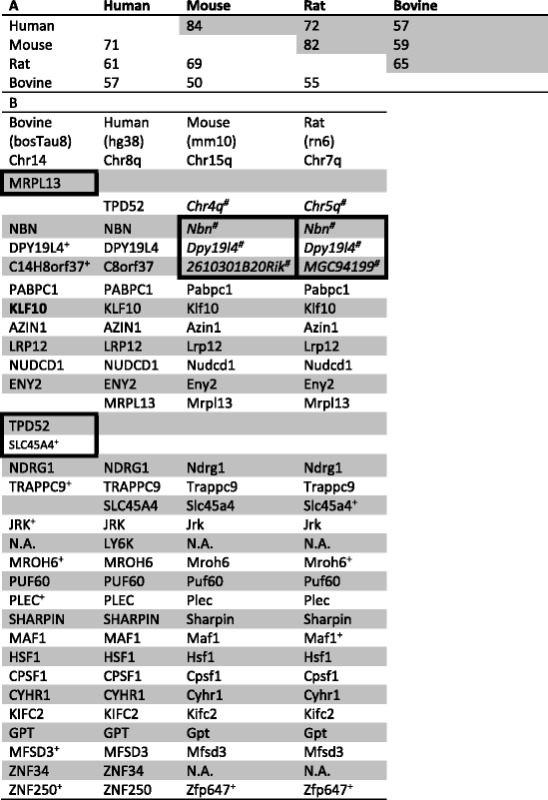
Synteny analysis of HSF1-CanSig 8q genes. A. Percentage of syntenic genes between species. Percentages are based on the number of genes in the column’s species. The percentages in lower triangle excluded the separated chr4q in mouse and chr5q in rat. Whereas, the percentages in the upper triangle (shaded) include the separated chr4q in mouse and chr5q in rat. B. Order of human genes is used as the reference. Genes are ordered in 5′-to-3′ direction according to respective reference genomes from top to bottom except for bovine, which is in reverse order. Non-syntenic genes are marked in boxes. Superscript + indicates the chromosomal location of the gene was determined by BLAT. Exact genomic coordinates of these genes can be found in Additional file 1: Table S6

Twenty-three out of 29 human HSF1-CanSig 8q genes are syntenic to mouse and rat. Although three non-syntenic genes (Nbn, Dpy19l4, and 2610301B20Rik in mouse and Nbn, Dpy19l4, and MGC94199 in rat) reside in other chromosomes, the order of their locations still agrees with that in humans. If these three genes are included in calculating the percentage of synteny, human-rat and human-mouse share 72 and 84% of synteny, respectively. In comparison between bovine and human, all 8q homologs in bovine are found in a single chromosome, i.e., chromosome 14, but in the complementary strand of the bovine’s reference genome. Twenty-five of the 29 8q genes are syntenic. If the entire 8q region is considered, bovine’s genes share only 57% of synteny with human versus 61–71% in rat and mouse, indicating bovine genome has accumulated a larger scale of genome rearrangement than human, mouse, and rat since divergence. Regardless, the gene order is largely preserved between human and bovine. This finding is congruent with the estimated divergence time among these four mammals [9]. According to the report, bovine and human and mouse and human diverge in about 71–113 million years ago (MYA) and 62–101 MYA, respectively. Furthermore, our findings concur with the synteny analyses from the genome sequencing project of human (Figure 46 of [10]), Brown Norway rat (Figure 4 of [11]), and Taurine cattle (Figure S12 of [12]). To further investigate the synteny of human 8q in mouse, rat, and bovine, the tool Cinteny [13] was used to visualize the extent of synteny and they can be found in Additional file 3: Figure S2.

There are two possible explanations to account for the observed syntenic conservation among these genes: (i) the divergence time between taxa is insufficient to observe significant rearrangement or (ii) the proximity of genes is essential for biological functions. Classic examples are the Hox and globin gene clusters. As the estimation from [9] is adequate to disregard the former, we were intrigued to explore the latter, i.e., the biological function shared among these genes.

### Synteny does not explain coexpression

To determine whether or not the overexpression of HSF1-CanSig 8q genes in cancers is related to synteny, we analyzed the expression pattern of genes flanking HSF1 (genomic coordinates of HSF1’s neighboring genes can be found in Additional file 1: Table S7) in each cancer case to shed light on the clustering of HSF1-CanSig 8q genes. We reasoned that if the expression level of HSF1’s neighboring genes correlates with their distance from HSF1, synteny may be a factor accounting for the clustering of CanSig 8q genes; otherwise reasons other than synteny may shape the clustering.

**Fig. 4:**
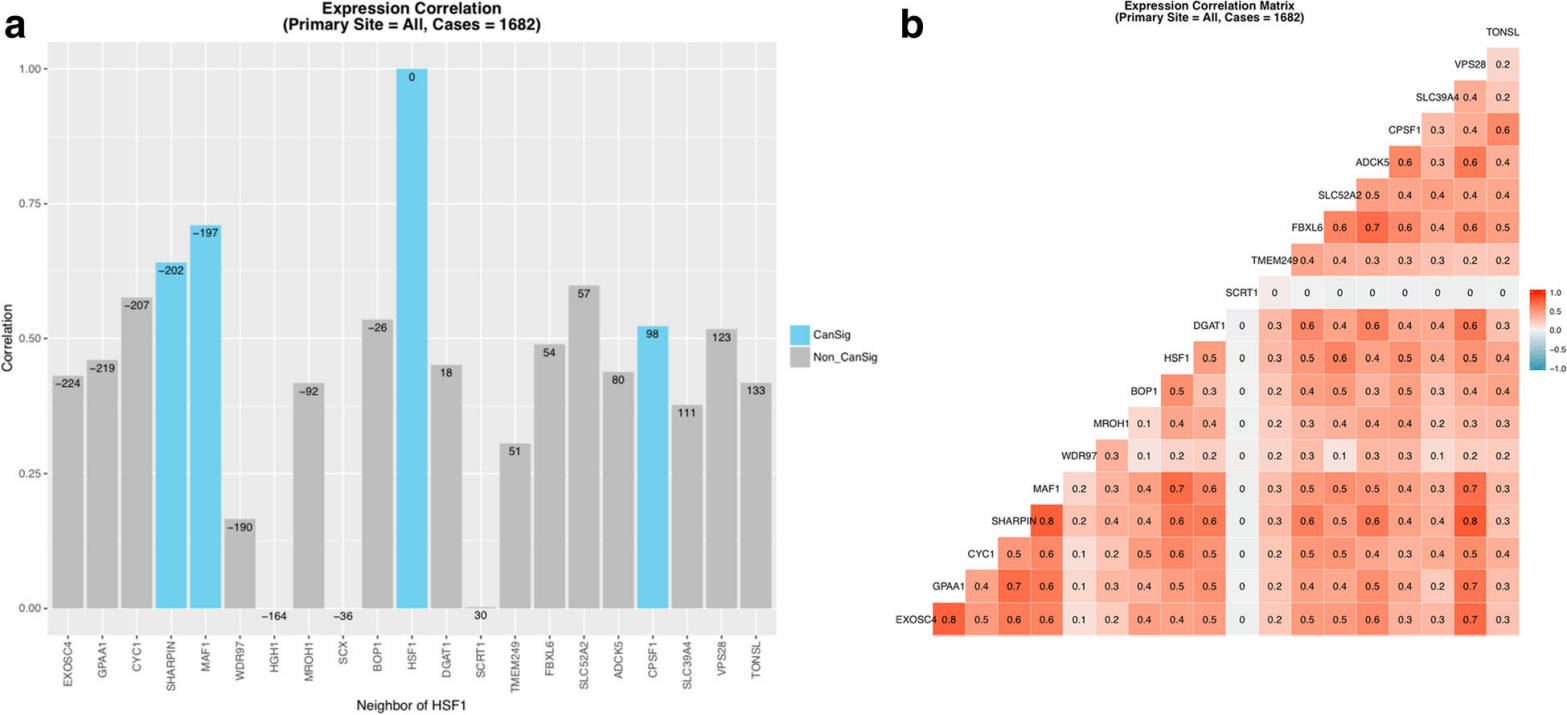
Expression correlation between genes in the neighborhood of HSF1. **a** HSF1 is set as the focal gene. No expression data can be obtained for HGH1 and SCX in cBioPortal; therefore, their correlations were set to zero. The numbers on the top of the bars represent the distance of the gene from HSF1 in Kbps. Negative signs indicate genes are located upstream of HSF1. **b** Correlation matrix of all 21 genes. Blank boxes represent insufficient number of cases to determine the correlation

We selected 1682 cancer cases with HSF1 overexpressed two or more standard deviations above the reference. As shown in Fig. 4a, expression of HSF1 and its neighboring genes do not correlate with the distance between them. Genes close to HSF1 such as MROH1, BOP1, and DGAT1 do not show higher correlation (in terms of overexpression with HSF1) than HSF1-CanSig 8q genes that are farther from them. For example, although 8q genes SHARPIN, MAF1, and CPSF1 are far apart from HSF1 than MROH1, BOP1, and DGAT1, they exhibit higher correlation with HSF1’s expression. Similar variability is also observed when correlations are examined by individual primary tumor site (Additional file 4). Thus, our data suggests that synteny is not a contributing factor for the co-overexpression of HSF1-CanSig genes clustered in chromosome 8q21-24. It is however intriguing to see that several overexpressed non-HSF1-CanSig genes are correlated with HSF1 such as MAF1 (*R* = 0.7), CYC1 (*R* = 0.6), and SLC52A2 (*R* = 0.6) (see Fig. 4b).

### Network analysis links HSF1-mediated responses to pre-mRNA 3’ processing

Next, we took a systems approach to determine if HSF1-CanSig 8q genes work cooperatively in biological networks. WebGestalt gene network (or pathway) analysis was performed to determine whether shared biological processes are common to these genes. The basic mechanism of WebGestalt is to detect the enrichment of Gene Ontology (GO) terms associated with a set of genes with statistical support. We used 29 HSF1-CanSig 8q genes to query 33 TCGA RNA-Seq studies in various primary tumor sites (the list of studies queried can be found in Additional file 1: Table S8). A sample of result generated by WebGestalt can be found in Additional file 3.

Out of the 33 TCGA datasets, eight studies were found to be enriched by HSF1-CanSig 8q genes: adrenocortical carcinoma, colon adenocarcinoma, esophageal carcinoma, kidney chromophobe, hepatocellular carcinoma, lung adenocarcinoma, ovarian serous cystadenocarcinoma, and stomach adenocarcinoma. Intriguingly, all results pointed to the enrichment of RNA polyadenylation (GO:0043631, *p* values 2.40e-3 to 4.34e-2), mRNA polyadenylation (GO:0006378, *p* values 2.40e-3 to 4.34e-2), and 3′ end processing (GO:0031124, *p* values 3.03–4.30e-2). With no exception, the CanSig 8q genes that contributed to these hits were HSF1, PABPC1, and CPSF1.

**Fig. 5:**
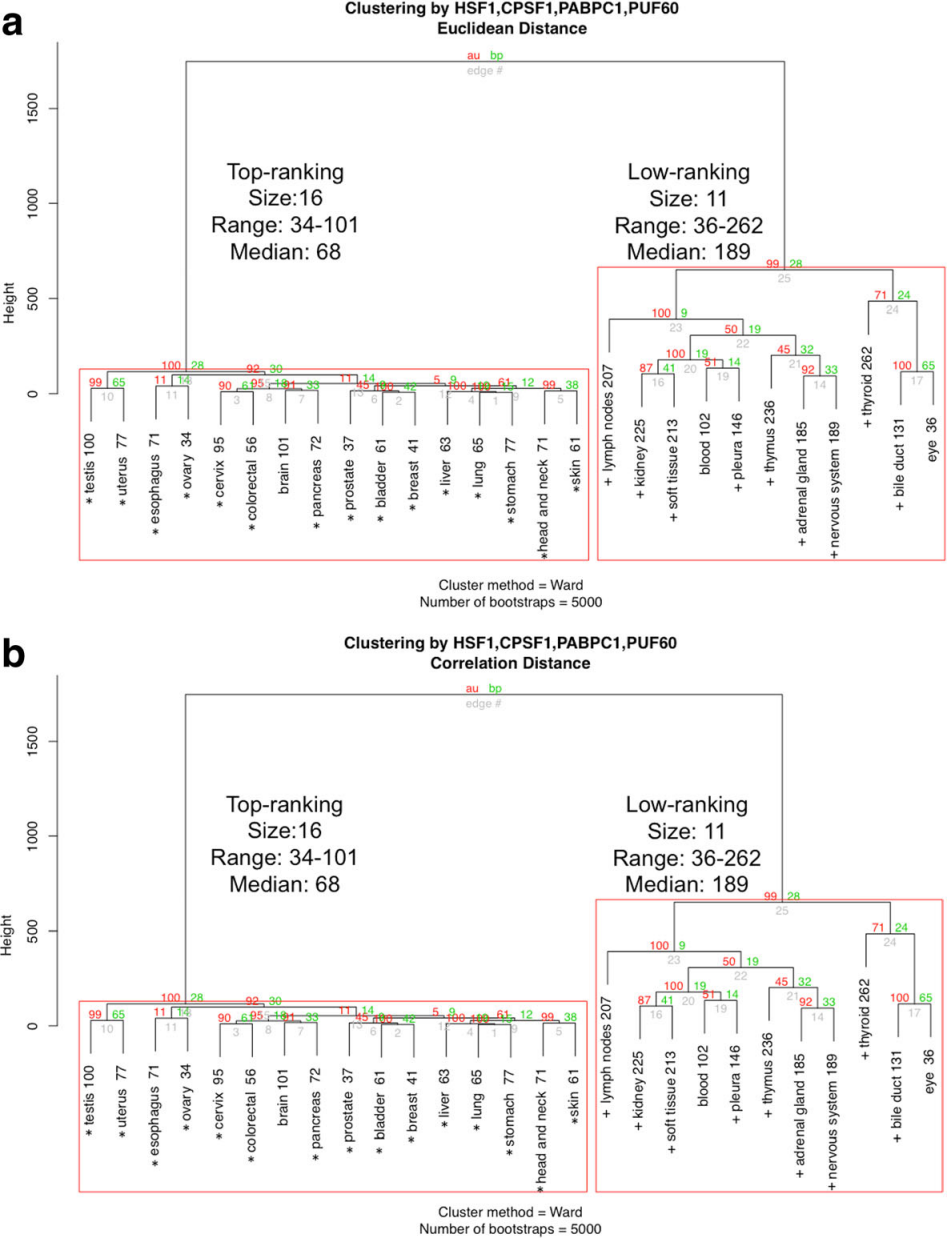
**a** Hierarchical clustering of rank data (Table 2) by HSF1 and three pre-mRNA 3′ processing factors: PABPC1, CPSF1, and PUF60. Distance method is based on the Euclidean distance. In the top-ranking group, the asterisk denotes the primary tumor site shared with Fig. 3a. In the low-ranking group, the plus sign indicates the primary site shared with Fig. 3a. **b** Clustering by correlation distance

To corroborate WebGestalt’s results, we repeated the hierarchical clustering analysis of rank data (Table 2) but using only HSF1 and three pre-mRNA 3′ processing genes: PABPC1, CPSF1, and PUF60. (Note that the clustering method requires a minimum of four genes; therefore, PUF60, a RNA processing factor, was included here even though WebGestalt did not highlight it.) If these four genes dominate the average rank of HSF1-CanSig 8q genes in different primary tumor sites, the clustering results produced by them should highly resemble to the results in Fig. 3. Figure 5a, b shows two cluster dendrograms produced by the two distance methods which share high similarity with Fig. 3a, b, respectively. When the two cluster dendrograms generated by the Euclidean distance (Figs. 3a and 5a) are compared, the top-ranking and low-ranking groups are found to share 15 and 9 common primary sites, respectively, which means 24 out of 27 primary sites. Similarly, the two dendrograms using correlation distance method (Figs. 3b and 5b) share 12 and 9 common primary sites, respectively. It means that these four genes are representative for all HSF1-CanSig 8q genes, indicating influential role played by them. It also suggests that HSF1 mediates pre-mRNA 3′ processing in cancer development.

## Discussion

We have undertaken a cancer bioinformatics approach to study the clustering of HSF1-CanSig genes in chromosome 8q. Based on the TCGA expression data obtained from cancer cases, we found that HSF1-CanSig genes are clustered at chromosome 8q substantiated by compelling statistical evidence under two null models: all protein-coding genes and all HSF1-CanSig genes. *λ* value analysis further reveals variation of 8q prevalence in 27 primary tumor sites (Fig. 2). This result may support the use of *λ* to characterize the strength of HSF1 cancer program in various cancers: bone [14], breast [1], colon [1], esophagus [3], head and neck [15], liver [16], lung [1], ovarian [5], pancreas [17], prostate [18], uterine [4], and skin cancer [19]. Crucially, this perspective has been confirmed by the hierarchical clustering analysis as all of the above cancers, except bone, are members of the top-ranking group (Figs. 3 and 5).

The rank analysis discovers the association between HSF1 and HSF1-CanSig genes encoded in chromosome 8q in 27 primary tumor sites. HSF1’s rank correlates with and always falls below the average rank across primary sites (Additional file 3: Figure S1). The ranks of 20 out of 27 primary tumor sites are asymmetrically skewed to high ranks with statistical significance, suggesting the overexpression of HSF1-CanSig 8q genes is coordinated in those tumor sites. In contrast, when the rank of HSF1 is greater than the cutoff rank ~ 102, more diverse overexpression patterns are observed among HSF1-CanSig 8q genes, implying the diminishing control of HSF1 in those cancers. These observations engender HSF1 to be an important target for the development of cancer treatment.

Clustered HSF1-CanSig 8q genes co-express at a high level in several primary tumor sites. Is the clustering of HSF1-CanSig 8q genes a contributing factor for co-expression biologically? As “Nothing in biology makes sense except in the light of evolution” [20], we sought answers from synteny. Our results (Table 3B) indicate the majority (22–23) of HSF1-CanSig 8q genes are syntenic among human, mouse, rat, and bovine. Despite the neighboring gene model suggesting an evolutionary explanation of this co-expression [21, 22], our results do not align with such a model. When the expressions of genes flanking HSF1 were examined, our results demonstrate incoherent overexpression levels attributed to distance. Thus, other forces such as the transcriptional activation by HSF1 or its downstream products may be the cause of the co-expression of HSF1-CanSig 8q genes in tumors. It still remains as a question whether this is common for other cancer-related transcription factors. Related to this analysis is the finding that a few non-HSF1-CanSig genes encoded in chromosome 8q whose expressions correlate with HSF1: GPAA1 (*R* ~ 0.8) and EXOSC4 (*R* ~ 0.75) in the ovary and FBXL6 (*R* ~ 0.8) in the prostate (Additional file 4). These findings concur with previous studies that genes in regions 8q21, 8q22, and 8q24 are notable for cancers [23–25].

Hierarchical clustering and network analysis have fostered the connection between HSF1 and pre-mRNA 3′ processing. Three essential pre-mRNA processing factors are discovered in relation to the overexpression of HSF1-CanSig genes through network analysis: PABPC1, CPSF1, and PUF60. CPSF1 is an essential polyadenylation factor. To date, no biochemical evidence supports any association between HSF1 and CPSF1. However, the interaction between the two processes, heat shock and polyadenylation, has been confirmed by two previous studies [26, 27], suggesting intermediaries may be involved in the crosstalk between these two processes. The poly(A) tail of a mRNA serves as a checkpoint for mRNA nuclear export. Intriguingly, HSF1-TRP interaction had been reported to facilitate the export of HSP70 mRNA under stress [28]. HSF1 had also been shown to form complexes with two core polyadenylation factors: symplekin and CstF64 [29, 30]. Alternative polyadenylation (APA) is ubiquitous and tissue-specific in mammals [31–33]. Importantly, it has been confirmed to play an essential role in many tumor types [34–43]. In rare cases, APA interferes with the protein encoded by the gene. Two classic, non-cancer examples are calcitonin-related polypeptide-alpha gene (CALCA) [44] and immunoglobulin M (IgM) [45]. But more often, APA alters the 3′ untranslated regions (UTRs) by either shortening or lengthening the constitutive 3′ UTRs. 3′ UTR is known to harbor binding sites of regulatory proteins, and miRNAs, in addition to secondary structures [46]. Their binding can either attenuate or bolster mRNA stability, affecting downstream protein synthesis and subsequently the proteome of cells.

It is noteworthy that two other HSF1-CanSig 8q genes are associated with pre-mRNA 3′-end processing: PUF60 and PABPC1. PUF60 is a member of the highly conserved nucleic acid-binding protein family: pumilio and FBF homology protein (PUF). PUF60 involves in pre-mRNA splicing and transcriptional regulation. Notably, PABPC1 binds to poly(A) tail of eukaryotic mRNA, promoting mRNA translatability. A proteomic study had identified the presence of PUF60 and PABPC1 in the polyadenylation complex [47], ascertaining the linkage between heat shock and polyadenylation.

## Conclusion

The role of HSF1 played in cancer proliferation and malignancy is critical and well established. The cancer bioinformatics approach taken by us provides new information about the overexpression of HSF1-CanSig 8q genes mediated by HSF1 in different tumor types, illuminating the connection between malignancy progression driven by HSF1 and pre-mRNA 3′ processing. However, the true underlying biological mechanisms that drives HSF1 relationship with chromosome 8q is multifaceted and largely unknown but may hold the key to understanding tumor development in certain tissues and organs. As the activation of the master regulator HSF1 varies among different primary tumor sites, HSF1-CanSig 8q genes may be developed as prognosis biomarkers for improving clinical outcomes.

## Materials and methods

### HSF1-CanSig genes and cancer expression data

The list of 456 HSF1 CanSig genes was downloaded from Table S5 of [1]. To facilitate searching by gene symbols, they were adhered to the standard HUGO Gene Nomenclature [48]. As a result, 87 non-HuGO gene symbols from [1] were replaced by the official HuGO gene symbols. Six of them were converted to multiple gene symbols, expanding the original list to 475 HSF1-CanSig genes. The complete list of HSF1-CanSig genes and their full names and chromosome locations can be found in Additional file 1: Table S2.

mRNA expression (in fold change) of the HSF1-CanSig genes in different cancer primary sites were retrieved from cBioPortal via its Web API [7]. At the time of writing, cBioPortal contains 159 cancer studies conducted in 29 primary tumor sites (Additional file 1: Table S3). Our goal is to include one RNA-Seq study per primary site. The following criteria were used to select expression data for our analysis:

1. The study is focused on a specific primary tumor.
2. Transcriptome data is generated by the study.
3. Only one study is selected per primary tumor site. If multiple transcriptome datasets are found for a primary site, the study recruited the largest number of cancer cases is chosen.
4. Only expressions of HSF1-CanSig genes are considered.

Transcriptome data were found in the majority, but not all, of cancer studies. In this report, 27 studies were selected according to the above criteria and they are tabulated in Additional file 1: Table S9.

### Overexpression score of HSF1-CanSig genes

A cancer study consists of a group of cancer cases or patients, and each cancer case is associated with a set of mRNA expression *z*-scores of genes. But in this report, our focus is solely on the 475 HSF1-CanSig genes mentioned above. cBioPortal pre-computes a *z*-score for each gene, representing the number of standard deviations of its expression deviates from the reference gene population [8]. For example, a *z*-score value 2.8318 for gene ANAT in the case TCGA-OR-A5J1-01 from the Adrenocortical Carcinoma study indicates that the gene ANAT is overexpressed by 2.8318 standard deviations above the reference.

To quantify the prevalence of overexpression of HSF1-CanSig genes among cancer cases, we devised the following scoring scheme to assess the magnitude of a gene’s overexpression as:

$$ Overexpression Score= Number of overexpressed cases/ Total number of cases $$

A gene is considered overexpressed if its cBioPortal’s expression *z*-score is ≥ 2, the same threshold used in cBioPortal website. For instance, the Adrenocortical Carcinoma study consists of 79 cancer cases (see Table 3), UBE2B gene was detected to overexpress in 33 cases. The overexpression score of UBE2B is 33/79 = .4177. The full list of genes’ overexpression scores by primary site can be found in Additional file 1: Table S4.

### Chromosome localization of top 100 HSF1-CanSig genes

When expression data of different primary tumor sites was combined for analysis, the overexpression score of an HSF1-CanSig gene was calculated by adding the overexpression scores of the gene in all primary sites. The top 100 highest scoring genes were used for the chromosome localization analysis. The complete list of the top 100 HSF1-CanSig genes can be found in Additional file 1: Table S5. Two sets of genes were chosen as the reference or null model: all human protein-coding genes based on HuGO [48] and genes implicated in cancer when mutated according to the COSMIC Cancer Gene Census [49]. In total, 19,073 and 633 of protein-coding genes and cancer census genes were used as references, respectively.

A hypergeometric model was used to assess the probability of localization. *P*(*i* ≥ *k*) = $1-{\Sigma}_{i=0}^{i=k-1}\left(\genfrac{}{}{0pt}{}{K}{i}\right)\left(\genfrac{}{}{0pt}{}{N-K}{n-i}\right)/\left(\genfrac{}{}{0pt}{}{N}{n}\right)$, where *K* is the number of genes encoded in the chromosome 8q, *k* is the number of 8q genes among the top 100 high-scoring HSF1-CanSig genes, *n* is the sample size, and *N* is the total number of protein-coding genes. For *K* = 401, *k* = 27, *n* = 100, and *N* = 19,073, the probability of observing 27 or more HSF1-CanSig 8q genes in a random sample of 100 genes by chance is 1.02 × 10^−22^.

There are 29 HSF1-CanSig genes in 8q. The probability of finding 27 out of 29 HSF1-CanSig 8q genes in a sample of 100 HSF1-CanSig genes can be determined by hypergeometric model. Using the same formula above: *K* (= 29) is the number of HSF1-CanSig genes encoded in 8q, *k* (= 27) is the number of 8q genes in the top 100, *n* (= 100) is the sample size, and *N* (= 475) is the total number of HSF1-CanSig genes. The probability is 6.85 × 10^−18^.

*λ* value analysis was performed by fitting of the linear form of the exponential distribution, i.e., ln*p*_*i*_ =  − *λi* + ln *λ*, to the rank data in Additional file 1: Table S5. Only the percentages of the top 10 high-ranking chromosome arms were used for fitting. Fitting was done by lm() function in *R*.

### Synteny analysis

The genomic segment encoding human HSF1-CanSig 8q genes were analyzed for syntenic conservation. This segment spans from 80,034,869 (TPD52) to 144,901,461 (ZNF250) in chromosome 8 of human. By using the homology information from NCBI HomoloGene database (build 68) [50], 267, 165, 237, and 199 homologous genes were identified with genomic coordinates in human (hg38), bovine (bosTau8), mouse (mm10), and rat (rn6), respectively. Their genomic locations were retrieved automatically from the UCSC Genome Browser [51] through the Python package CruzDb [52]. For genomic locations that cannot be obtained by this approach, they were either determined manually by aligning the cDNA sequences on the genome by BLAT [53] or discarded. Genomic coordinates of the genes can be found in Additional file 1: Table S6.

### Coexpression analysis

Ten genes immediately flanking the upstream and downstream regions of HSF1 were identified in the human genome (hg38) using the UCSC Genome Browser [51]. This region spans about 365 kbps. The ten immediate upstream genes of HSF1 are EXOSC4, GPAA1, CYC1, SHARPIN*, MAF1*, WDR97, HGH1, MROH1, BOP1, and SCX. The ten downstream genes are DGAT1, SCRT1, TMEM249, FBXL6, SLC52A2, ADCK5, CPSF1*, SLC39A4, VPS28, and TONSL (HSF1-CanSig genes are suffixed by an asterisk). Genomic coordinates of them can be found in Additional file 1: Table S7. Expressions (in fold changes) of these 21 genes, including HSF1, were retrieved from cBioPortal if HSF1’s expression of a cancer case is ≥ 2 standard deviations higher than the reference. Correlation of expression fold change was computed between each of the 20 genes and HSF1 using the function cor.test in *R* where the method was Pearson.

### Student’s *t* test and hierarchical clustering

Our goal is to assess the statistical significance of the average rank of HSF1-CanSig 8q genes per primary site (listed in the second column from the right in Table 2). By assuming ranks are independent and uniformly distributed, the average rank of a sample consisting of 29 ranks randomly drawn from 1 to 466 without replacement does distribute normally with mean 233. (Note that the largest rank is 466 instead of 475 because of ties.) Thus, *t* test is suitable for this analysis. *R*’s t.test() function was used with mean 233 (null hypothesis). The mean was 233 as the range of rank is 1 to 466. *p* values of the *t* test can be found in the rightmost column in Table 2.

Hierarchical clustering was performed using *R*’s package pvclust [54]. One distinct feature offered by pvclust is the assessment of cluster subtrees by resampling bootstrap. The input to pvclust was the gene ranks in Table 2. The cluster method and the number of bootstrapping were “Ward” and 5000, respectively. Two distance calculation methods were used for cross-validation purpose: Euclidean distance and correlation.

### WebGestalt analysis

We used WebGestalt to uncover possible gene networks commonly perturbed by HSF1-CanSig 8q genes [55]. At WebGestalt website [56], we selected “hsapiens” as the organism, “Network Topology-based Analysis (NTA)” as the method of interest, “network” as the functional database, and “genesymbol” as gene ID type. Based on these parameters, WebGestalt automatically shortlisted 33 cancer studies from The Cancer Genome Atlas (TCGA) that were available for searching. We used 27 HSF1-CanSig 8q genes, including HSF1, identified from the top 100 most frequently overexpressed genes to query WebGestalt while values of other parameters remained in default settings. We repeated the search for all 33 TCGA cancer studies (Additional file 1: Table S8).

## Additional files

Additional file 1: Table S1. Statistical tests of observing HSF1-CanSig genes by chromosome arm. **Table S2.** List of 475 HSF1-CanSig genes. **Table S3.** List of 29 primary tumor sites. **Table S4.** Overexpression scores of HSF1-CanSig genes in each primary site. **Table S5.** Top 100 most frequently overexpressed HSF1-CanSig genes. **Table S6.** Genomic coordinates of syntenic genes. **Table S7.** Genomic coordinates of HSF1’s flanking genes. **Table S8.** List of TCGA studies queried in WebGestalt website. (XLSX 643 kb)

Additional file 2: Percentage distribution of HSF1-CanSig genes by chromosome arm for each primary tumor site. For each primary site, two plots are included, one using all protein-coding genes and cancer census genes as references. The other uses HSF1-CanSig genes as the reference. (PDF 186 kb)

Additional file 3: Rank of HSF1 among primary sites. Syntenty visualization by Cinteny. An example showing the output web page of WebGestalt (PDF 738 kb)

Additional file 4: Expression correlation analysis of syntenic genes for each primary tumor sites. (PDF 240 kb)

## Abbreviations

GO: Gene Ontology
HSF1-CanSig Gene: Heat Shock Factor 1 Cancer Signature Gene
HuGO: Human Genome Organization
MYA: Million years ago
TCGA: The Cancer Genome Atlas
UTR: Untranslated region
