# Supplementary material for: Overexpressed HSF1 cancer signature genes cluster in human chromosome 8q
Source: Hum Genomics. 2017 Dec 21;11:35. doi: 10.1186/s40246-017-0131-5 (PMC5740759; doi:10.1186/s40246-017-0131-5)
Supplement: Supplementary file 2 — Percentage distribution of HSF1-CanSig genes by chromosome arm for each primary tumor site. For each primary site, two plots are included, one using all protein-coding genes and cancer census genes as references. The other uses HSF1-CanSig genes as the reference. (PDF 186 kb) [file 40246_2017_131_MOESM2_ESM.pdf]

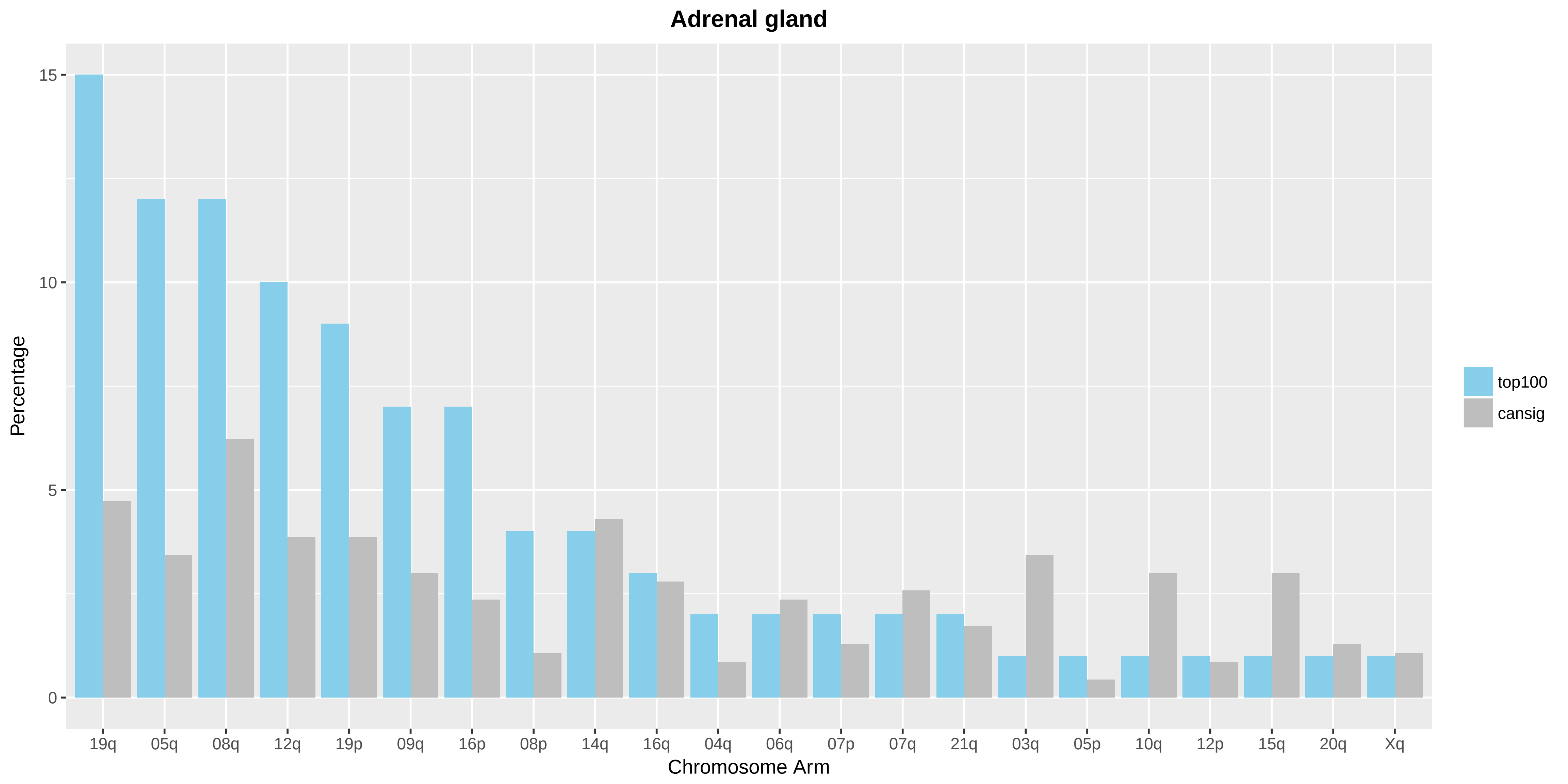

Adrenal gland
15
10
5
0
Percentage
19q
05q
08q
12q
19p
09q
16p
08p
14q
16q
04q
06q
07p
07q
21q
03q
05p
10q
12p
15q
20q
Xq
Chromosome Arm
top100
cansig

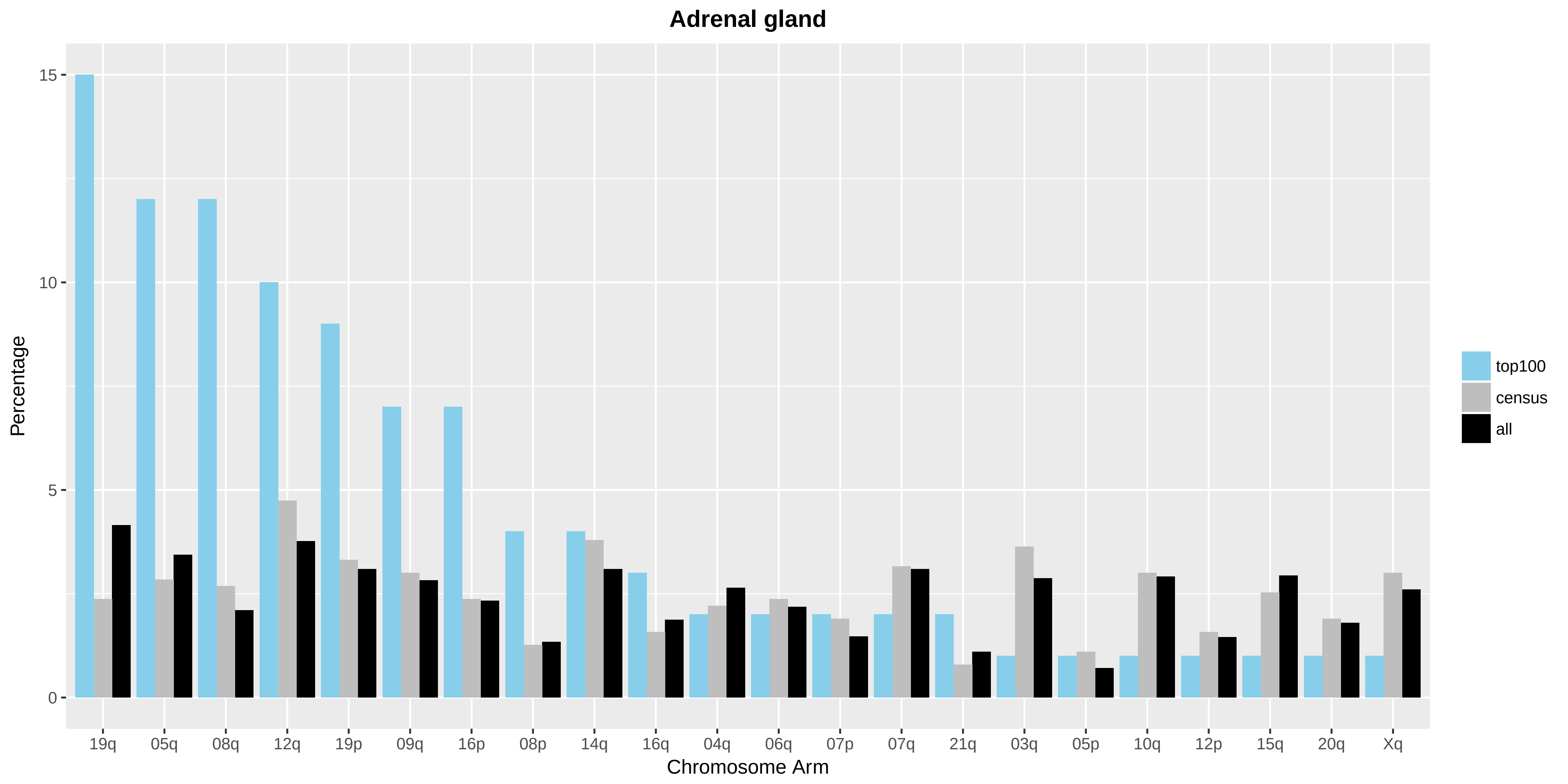

Adrenal gland
Percentage
15
10
5
0
19q
05q
08q
12q
19p
09q
16p
08p
14q
16q
04q
06q
07p
07q
21q
03q
05p
10q
12p
15q
20q
Xq
Chromosome Arm
top100
census
all

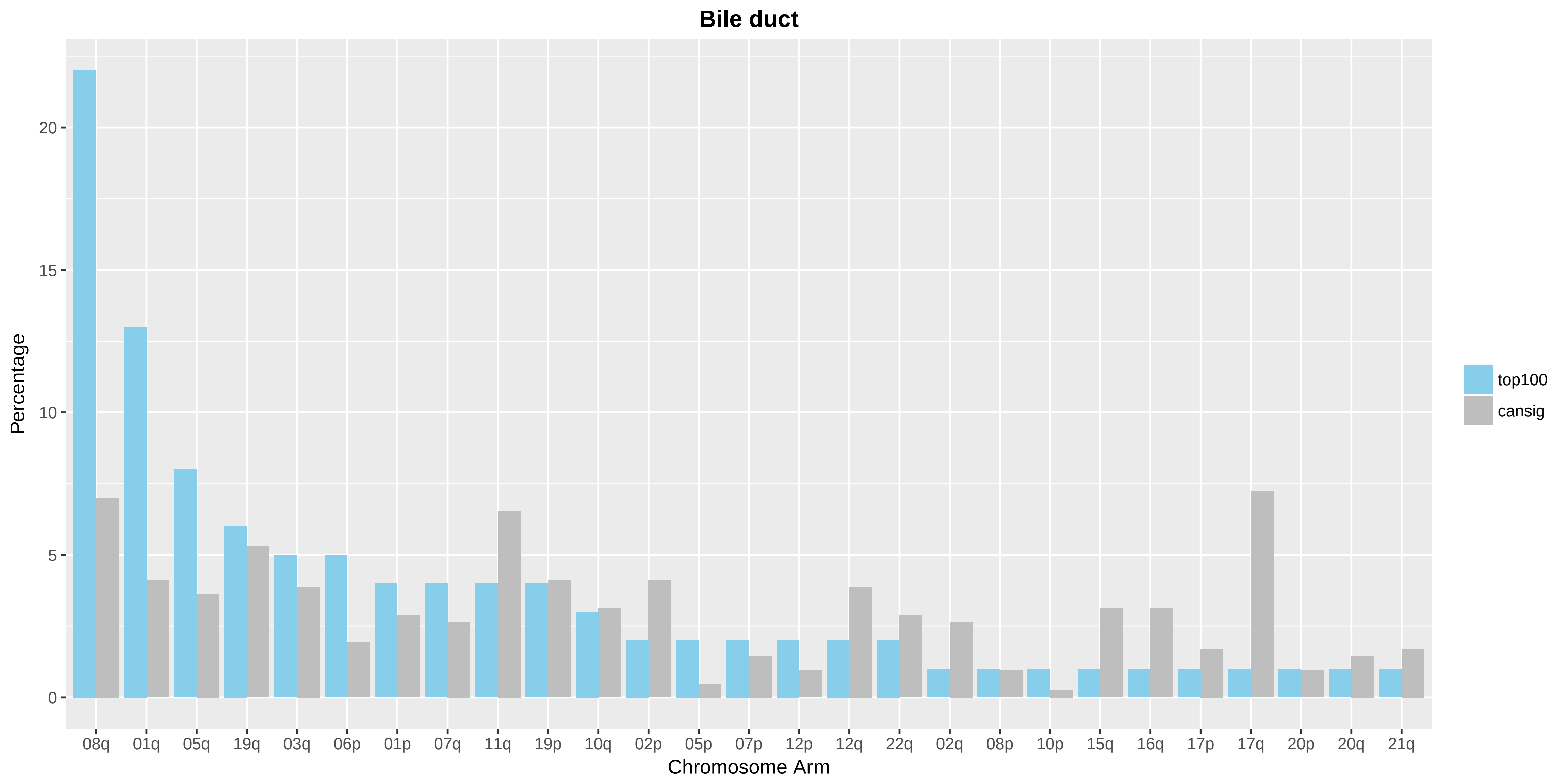

Bile duct
Percentage
0
5
10
15
20
08q
01q
05q
19q
03q
06p
01p
07q
11q
19p
10q
02p
05p
07p
12p
12q
22q
02q
08p
10p
15q
16q
17p
17q
20p
20q
21q
Chromosome Arm
top100
cansig

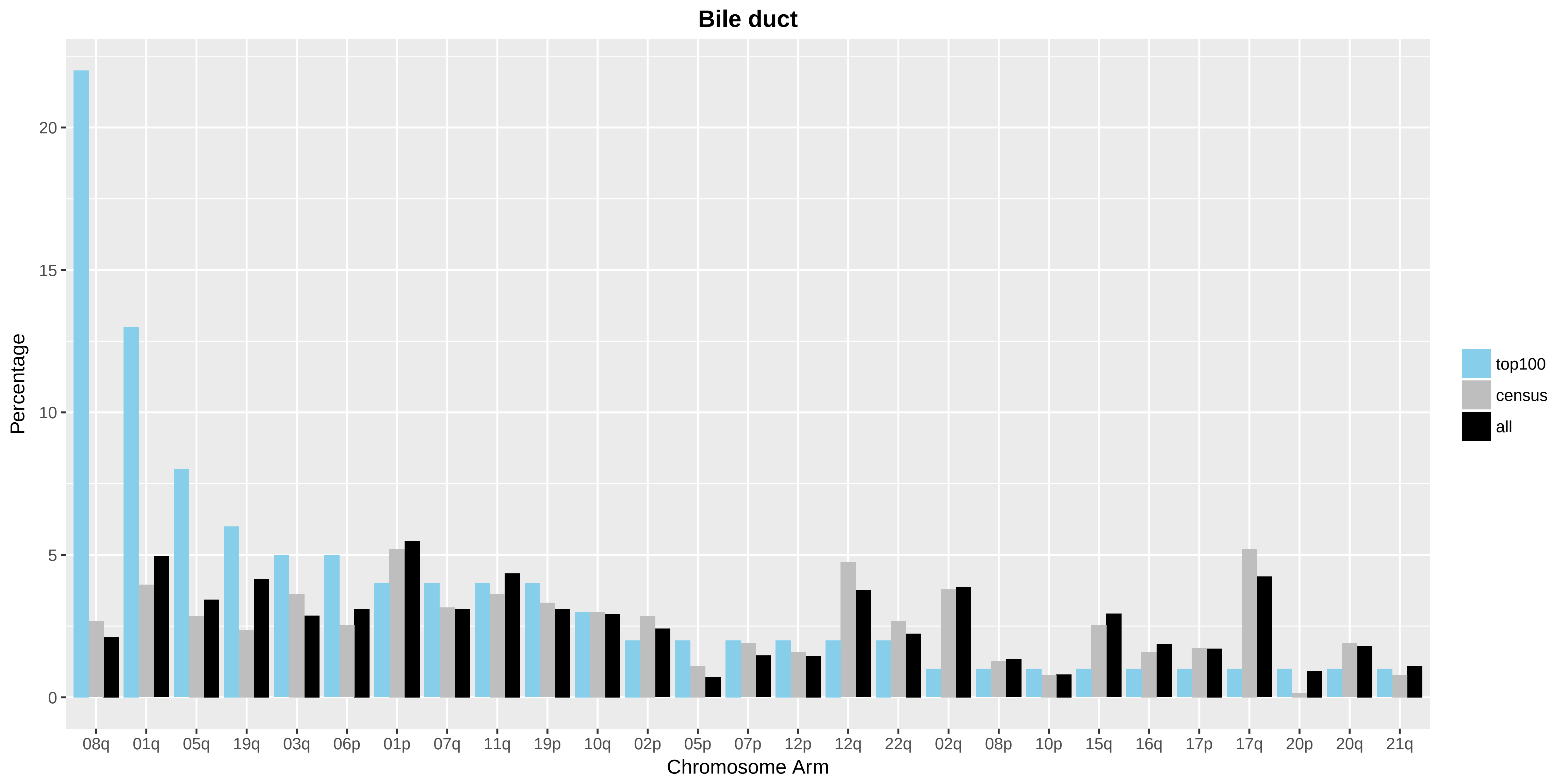

Bile duct
Percentage
0
5
10
15
20
08q
01q
05q
19q
03q
06p
01p
07q
11q
19p
10q
02p
05p
07p
12p
12q
22q
02q
08p
10p
15q
16q
17p
17q
20p
20q
21q
Chromosome Arm
top100
census
all

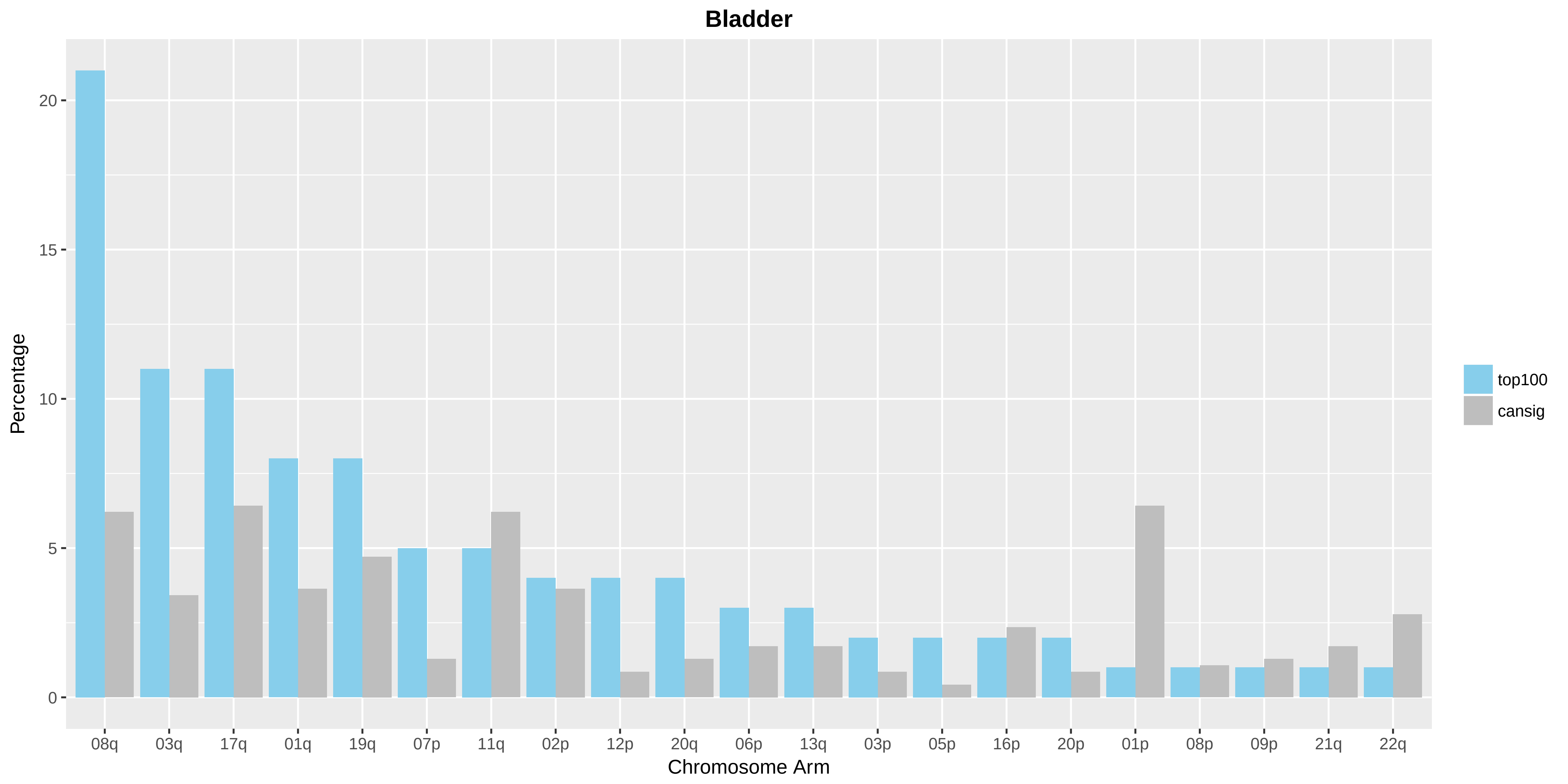
Bladder
Percentage
20
15
10
5
0
08q
03q
17q
01q
19q
07p
11q
02p
12p
20q
06p
13q
03p
05p
16p
20p
01p
08p
09p
21q
22q
Chromosome Arm
top100
cansig

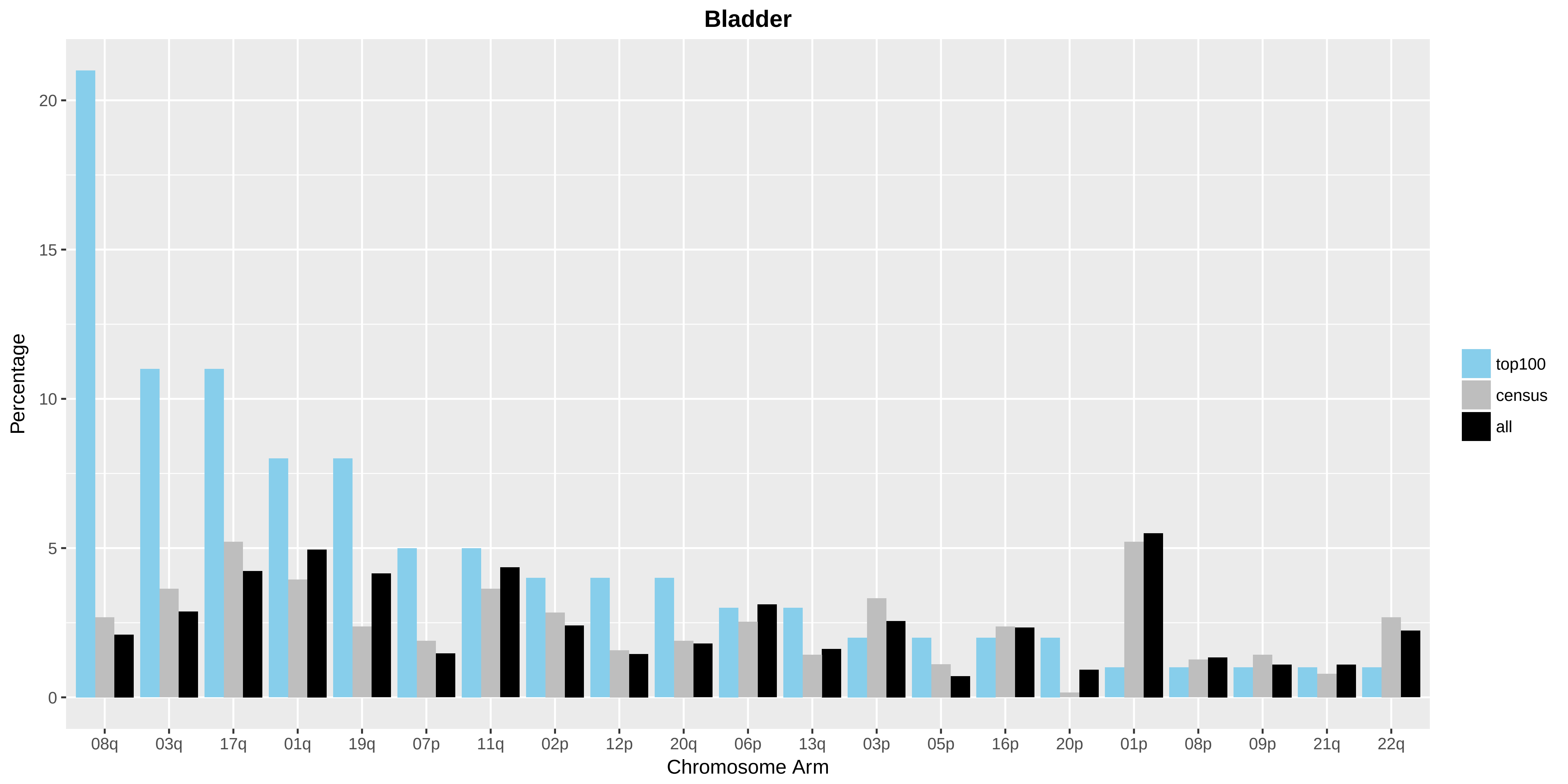
Bladder
Percentage
0
5
10
15
20
08q
03q
17q
01q
19q
07p
11q
02p
12p
20q
06p
13q
03p
05p
16p
20p
01p
08p
09p
21q
22q
Chromosome Arm
top100
census
all

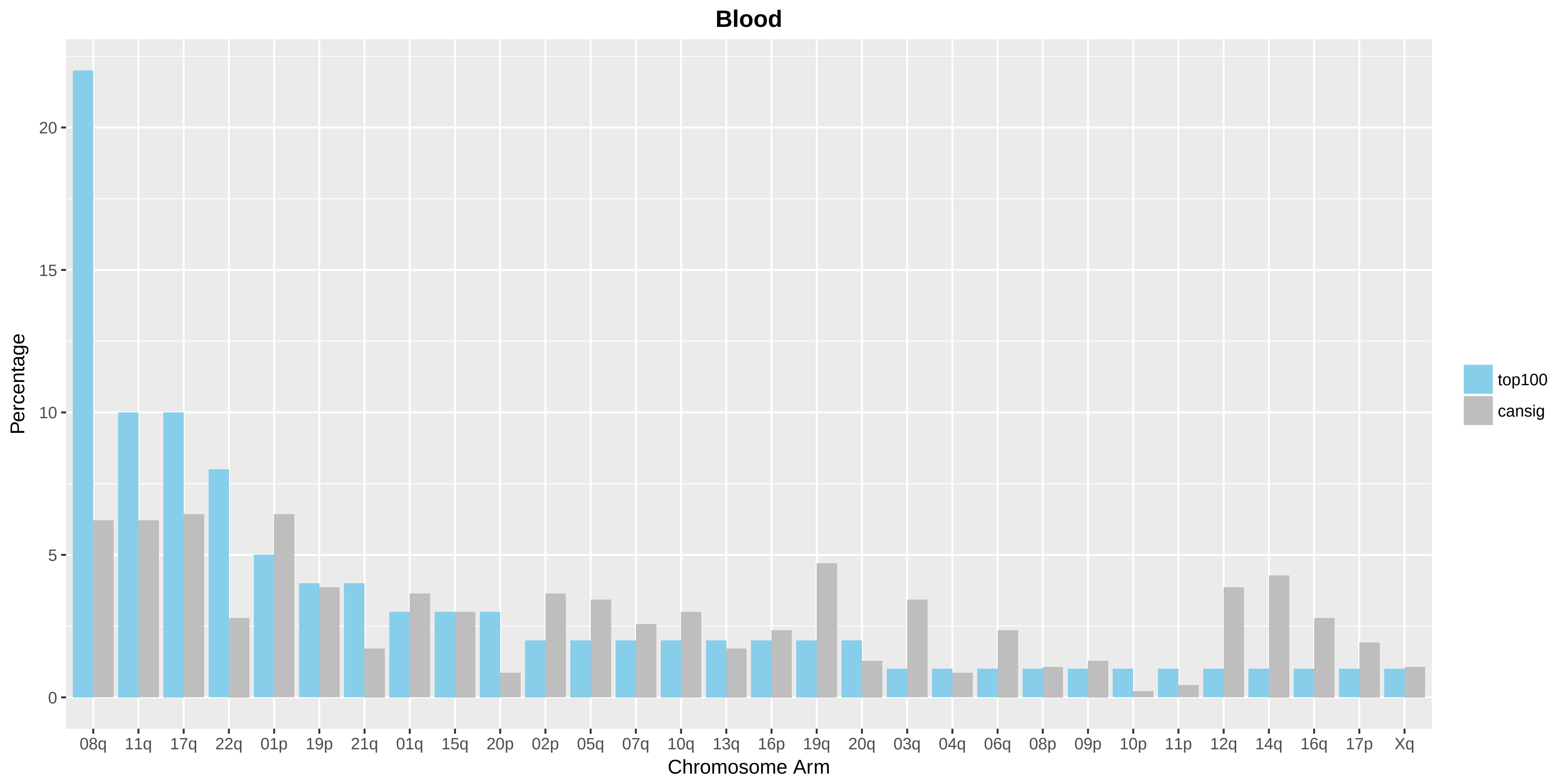

Blood
Percentage
0
5
10
15
20
08q
11q
17q
22q
01p
19p
21q
01q
15q
20p
02p
05q
07q
10q
13q
16p
19q
20q
03q
04q
06q
08p
09p
10p
11p
12q
14q
16q
17p
Xq
Chromosome Arm
top100
cansig

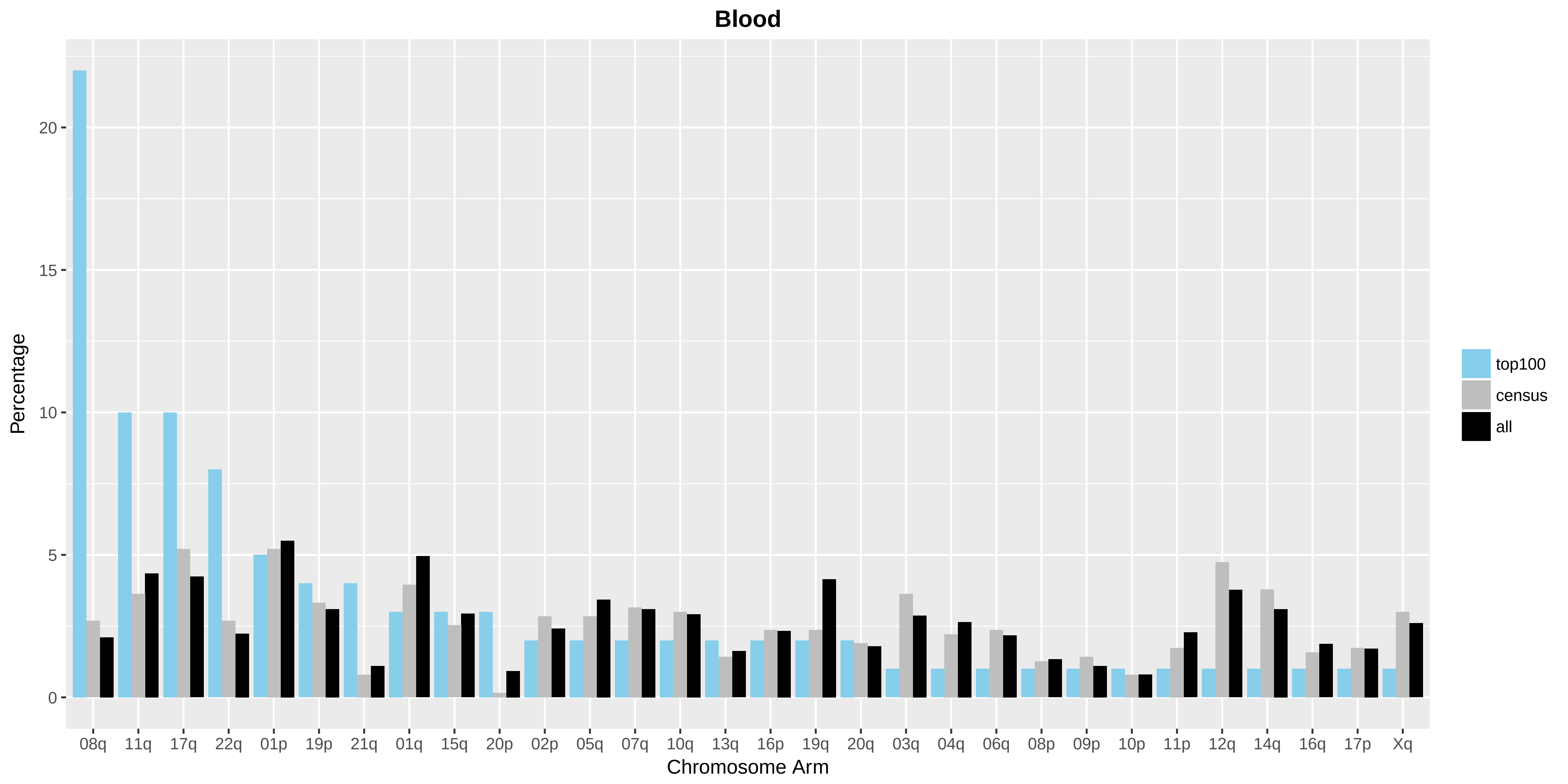
Blood
Percentage
0
5
10
15
20
08q
11q
17q
22q
01p
19p
21q
01q
15q
20p
02p
05q
07q
10q
13q
16p
19q
20q
03q
04q
06q
08p
09p
10p
11p
12q
14q
16q
17p
Xq
Chromosome Arm
top100
census
all

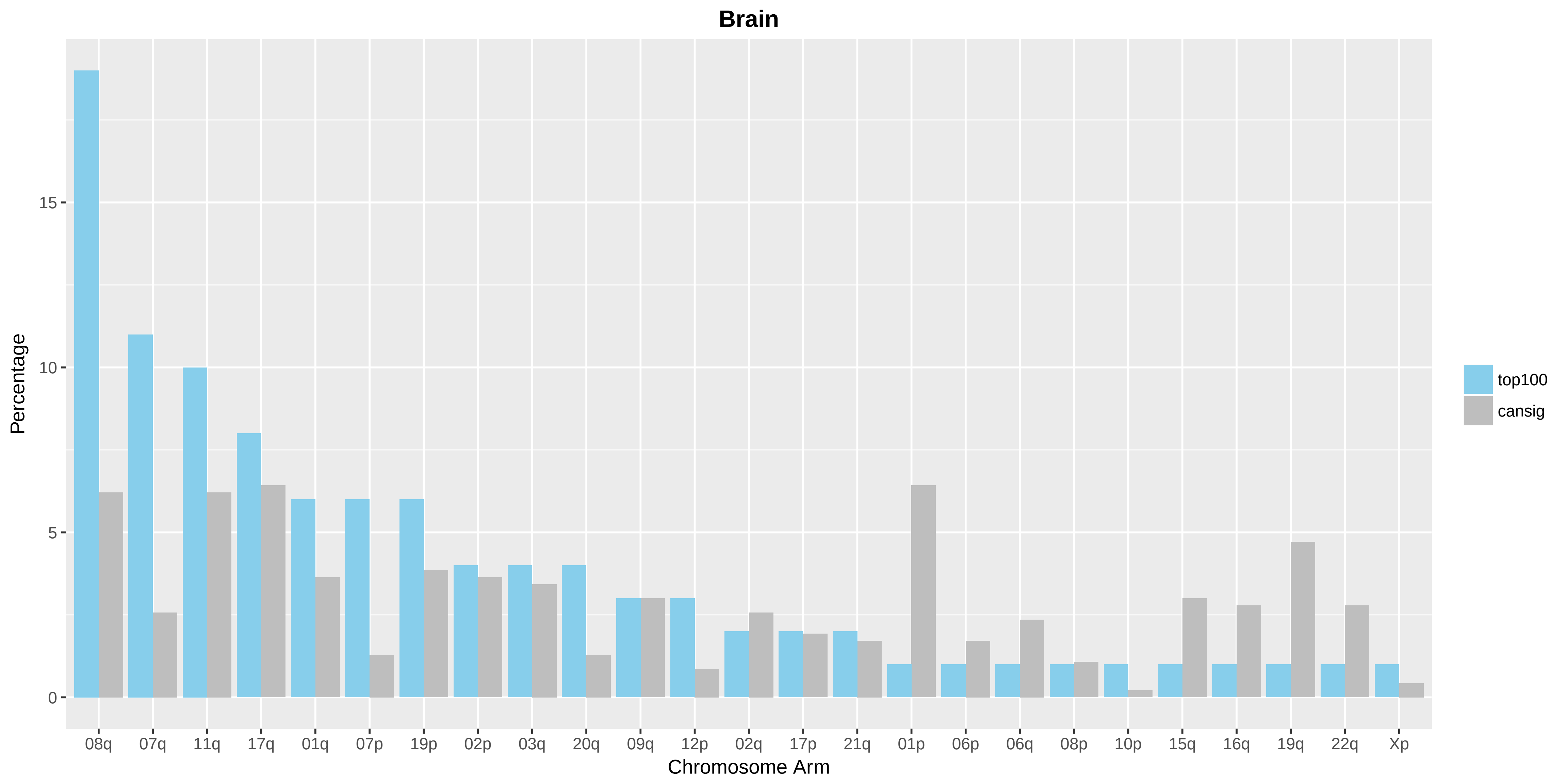
Brain
Percentage
0
5
10
15
08q
07q
11q
17q
01q
07p
19p
02p
03q
20q
09q
12p
02q
17p
21q
01p
06p
06q
08p
10p
15q
16q
19q
22q
Xp
Chromosome Arm
top100
cansig

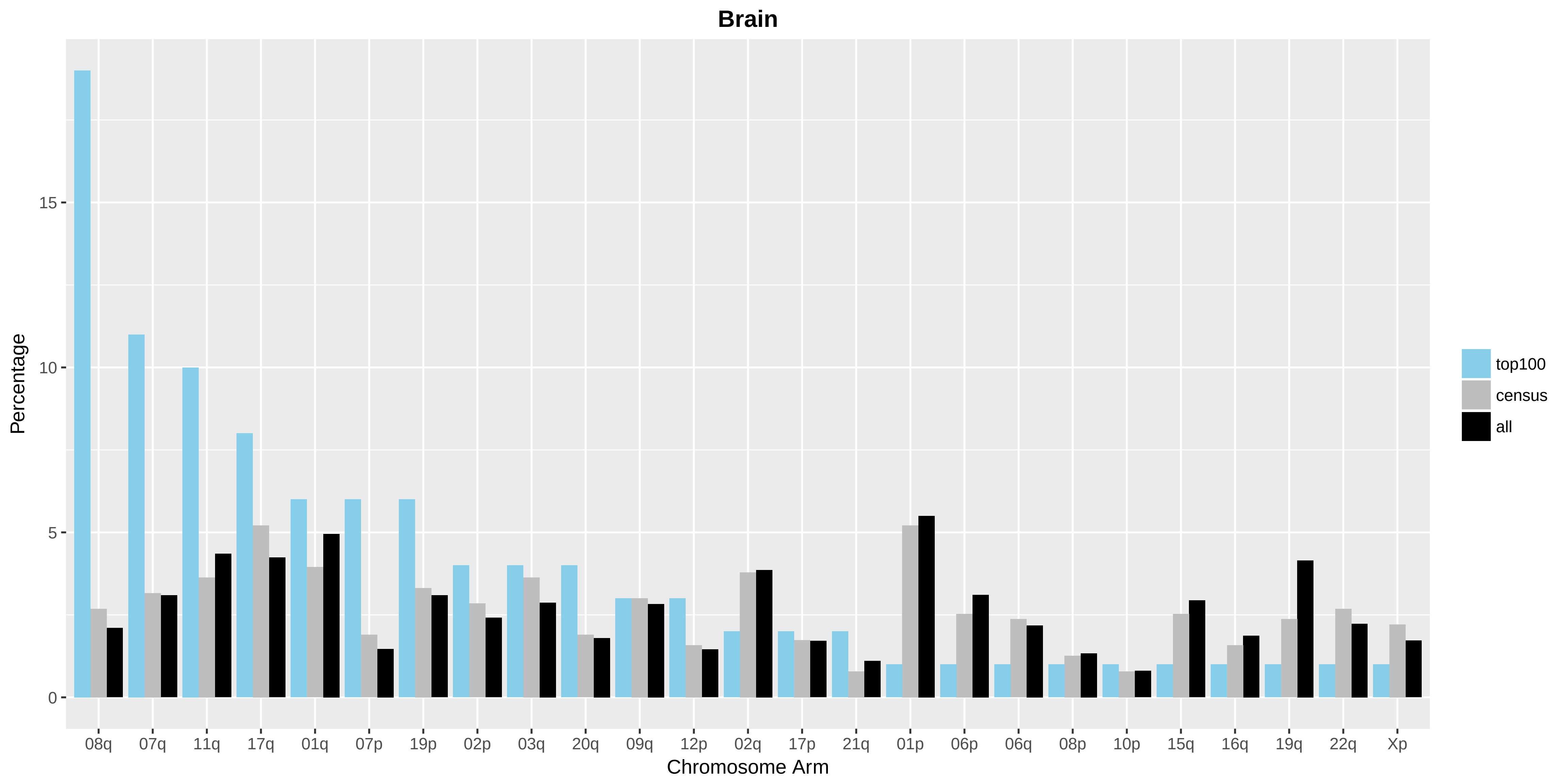

Brain
Percentage
15
10
5
0
08q
07q
11q
17q
01q
07p
19p
02p
03q
20q
09q
12p
02q
17p
21q
01p
06p
06q
08p
10p
15q
16q
19q
22q
Xp
Chromosome Arm
top100
census
all

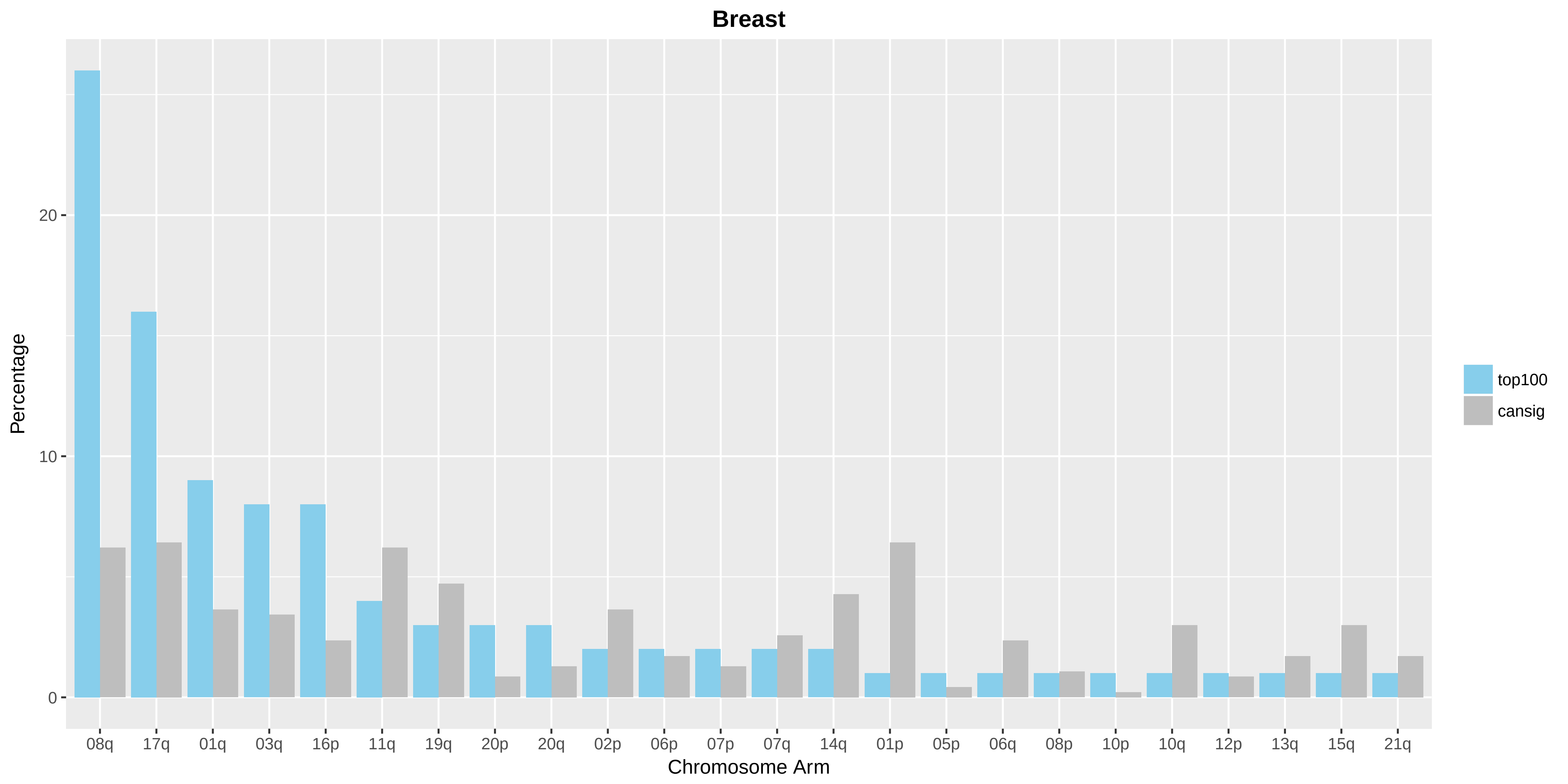
Breast
Percentage
0
10
20
08q
17q
01q
03q
16p
11q
19q
20p
20q
02p
06p
07p
07q
14q
01p
05p
06q
08p
10p
10q
12p
13q
15q
21q
Chromosome Arm
top100
cansig

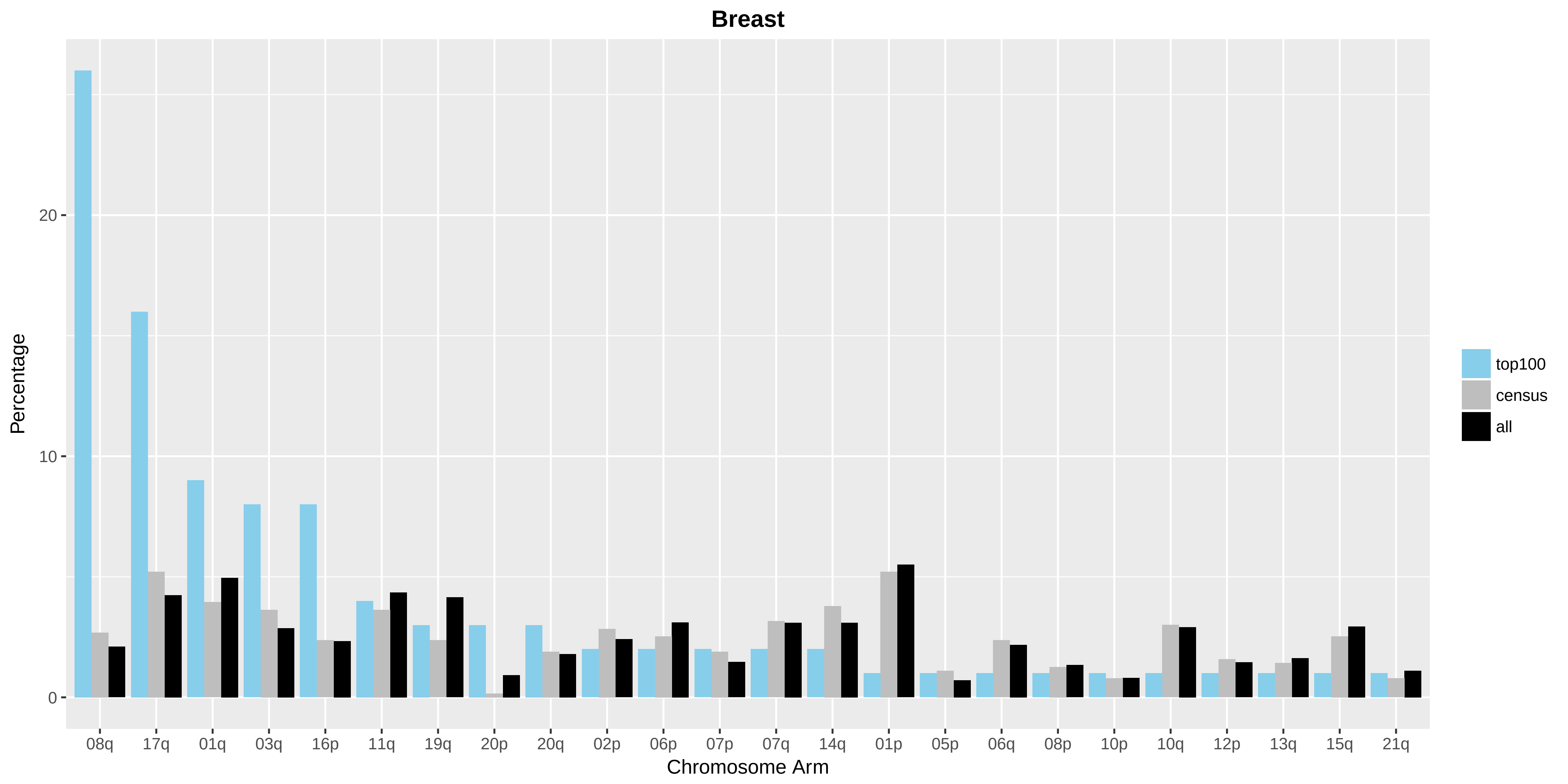
Breast
Percentage
0
10
20
08q
17q
01q
03q
16p
11q
19q
20p
20q
02p
06p
07p
07q
14q
01p
05p
06q
08p
10p
10q
12p
13q
15q
21q
Chromosome Arm
top100
census
all

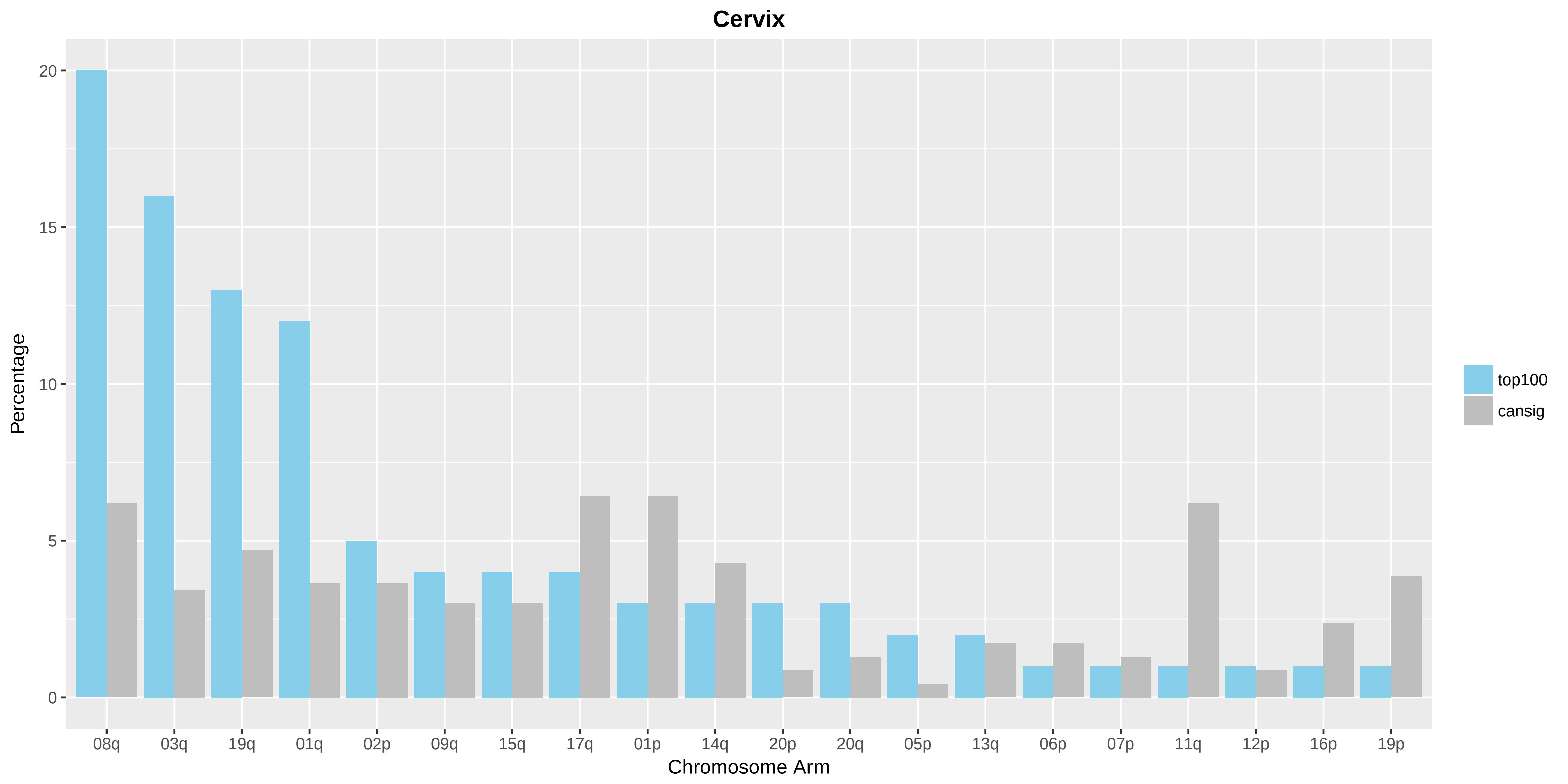

Cervix
Percentage
0
5
10
15
20
08q
03q
19q
01q
02p
09q
15q
17q
01p
14q
20p
20q
05p
13q
06p
07p
11q
12p
16p
19p
Chromosome Arm
top100
cansig

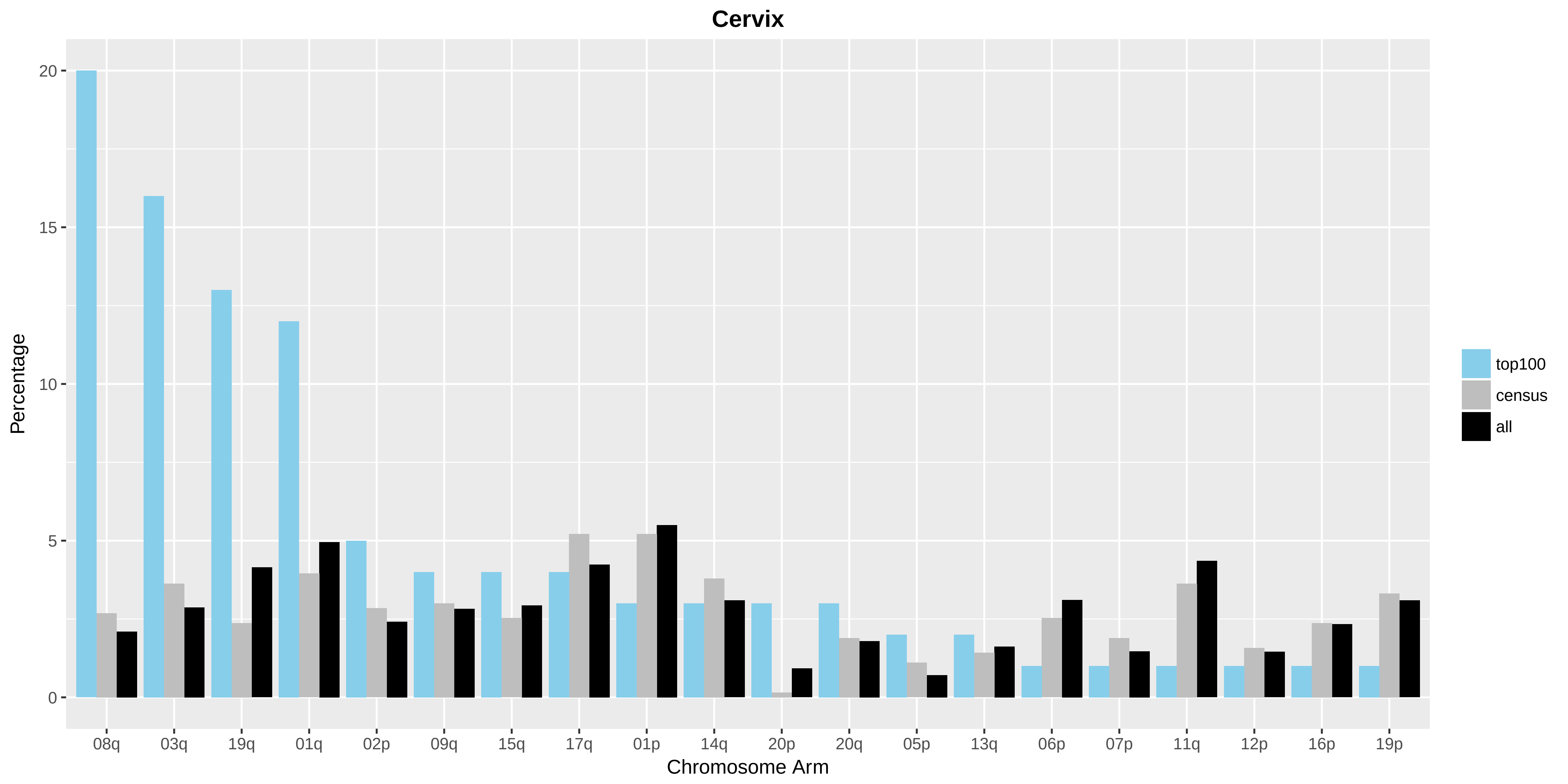

Cervix
Percentage
20
15
10
5
0
08q
03q
19q
01q
02p
09q
15q
17q
01p
14q
20p
20q
05p
13q
06p
07p
11q
12p
16p
19p
Chromosome Arm
top100
census
all

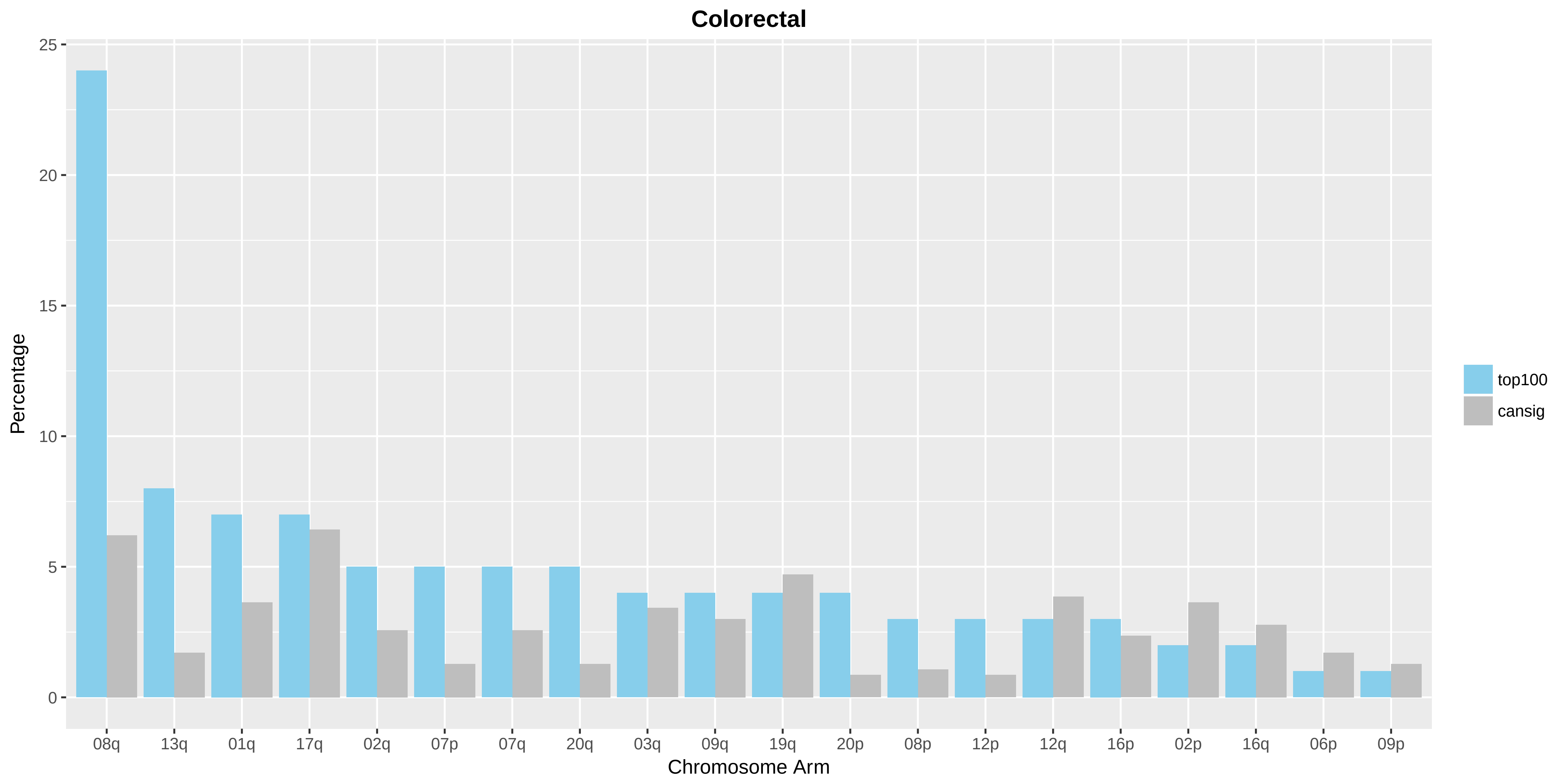
Colorectal
Percentage
25
20
15
10
5
0
08q
13q
01q
17q
02q
07p
07q
20q
03q
09q
19q
20p
08p
12p
12q
16p
02p
16q
06p
09p
Chromosome Arm
top100
cansig

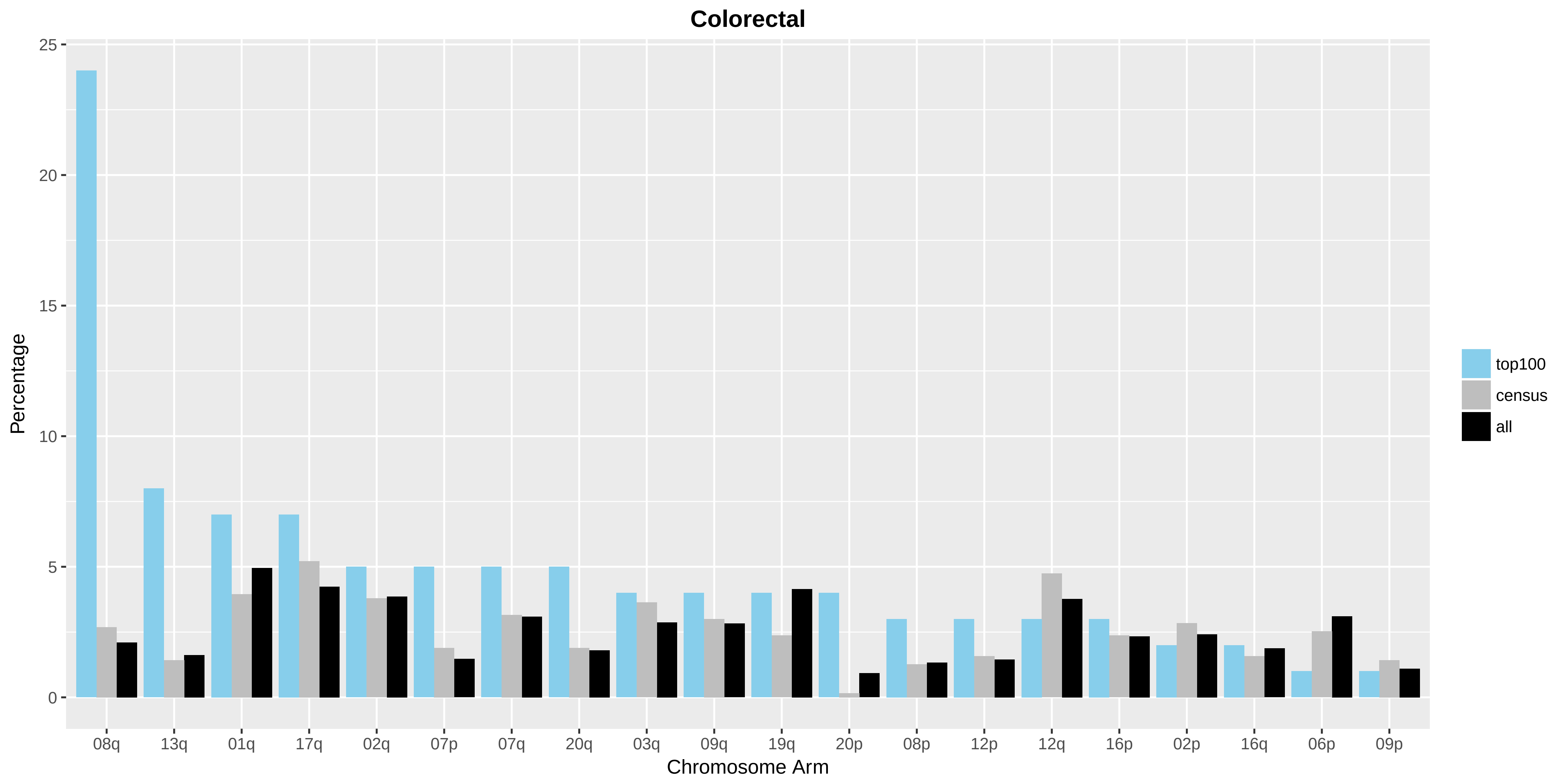

Colorectal
Percentage
0
5
10
15
20
25
08q
13q
01q
17q
02q
07p
07q
20q
03q
09q
19q
20p
08p
12p
12q
16p
02p
16q
06p
09p
Chromosome Arm
top100
census
all

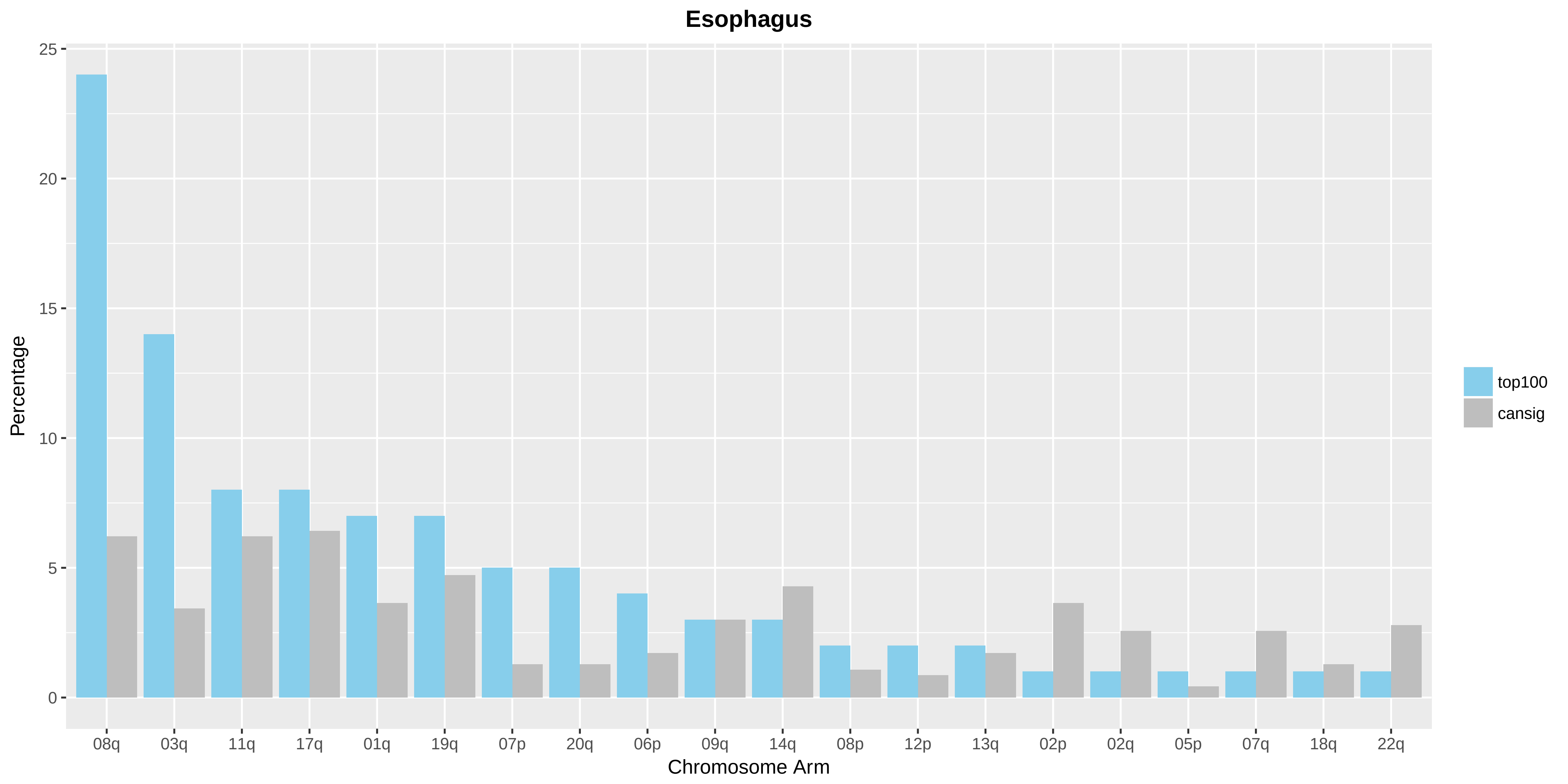

Esophagus
Percentage
0
5
10
15
20
25
08q
03q
11q
17q
01q
19q
07p
20q
06p
09q
14q
08p
12p
13q
02p
02q
05p
07q
18q
22q
Chromosome Arm
top100
cansig

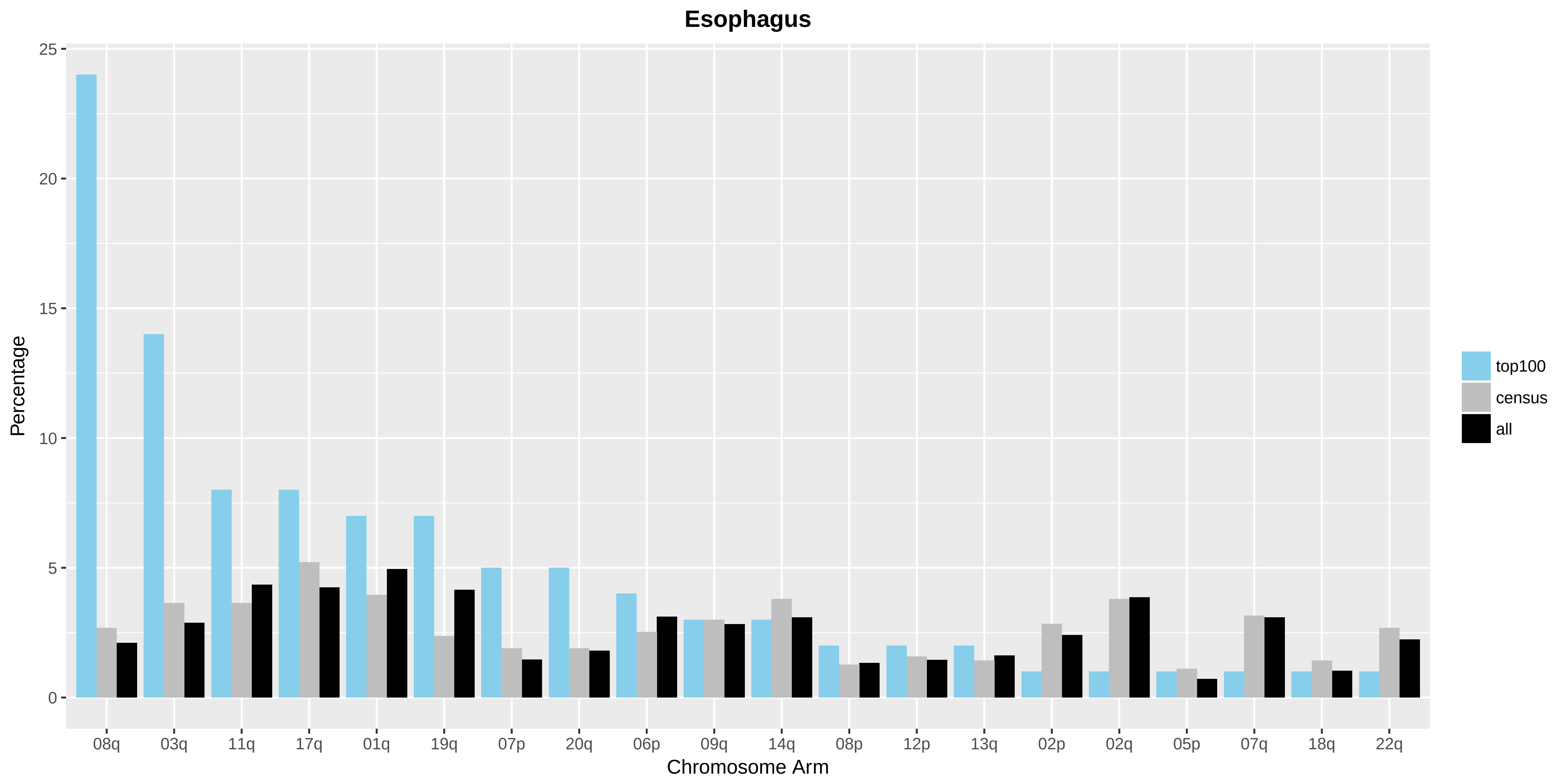

Esophagus
Percentage
25
20
15
10
5
0
08q
03q
11q
17q
01q
19q
07p
20q
06p
09q
14q
08p
12p
13q
02p
02q
05p
07q
18q
22q
Chromosome Arm
top100
census
all

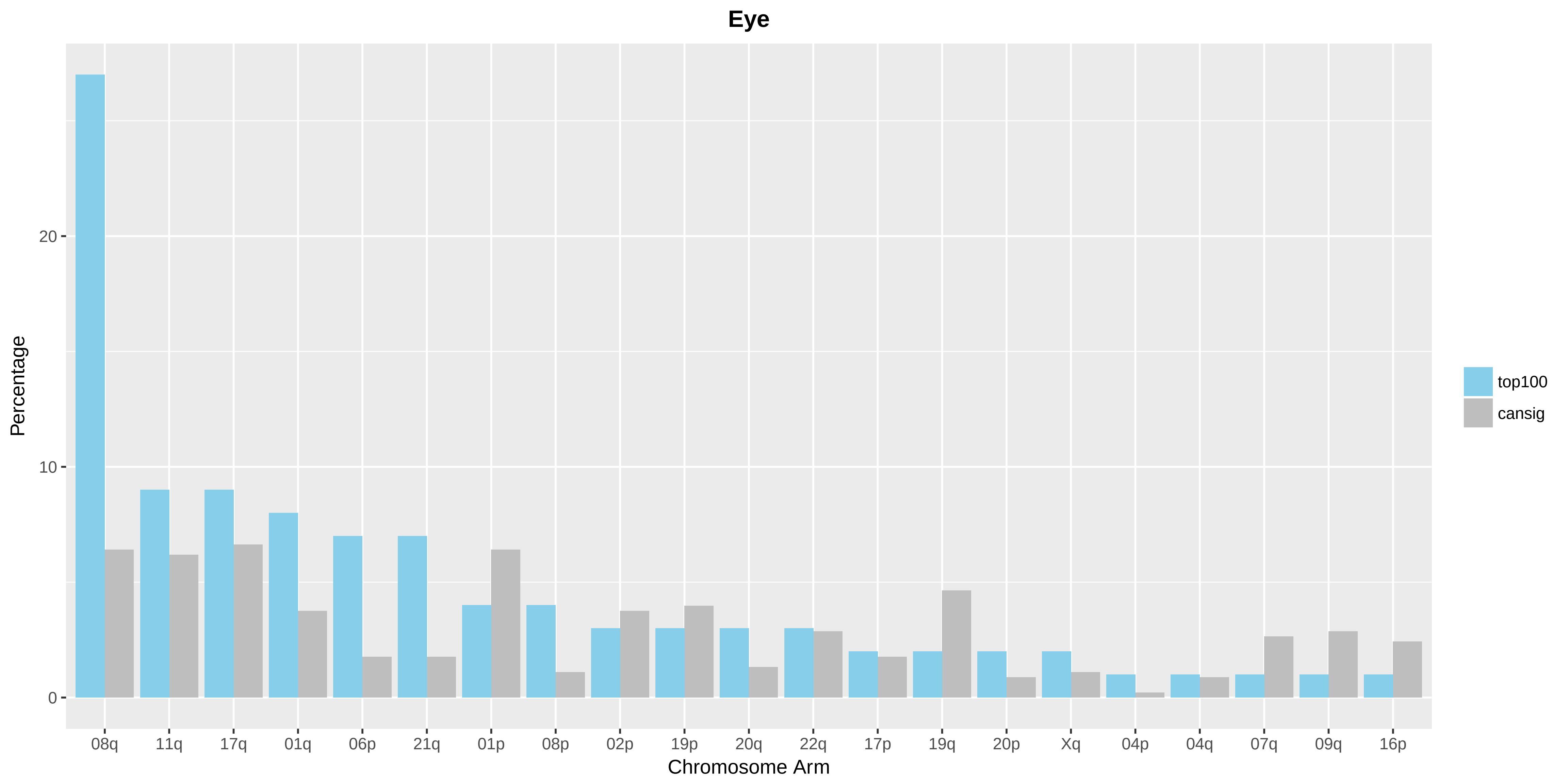

Eye
Percentage
0
10
20
08q
11q
17q
01q
06p
21q
01p
08p
02p
19p
20q
22q
17p
19q
20p
Xq
04p
04q
07q
09q
16p
Chromosome Arm
top100
cansig

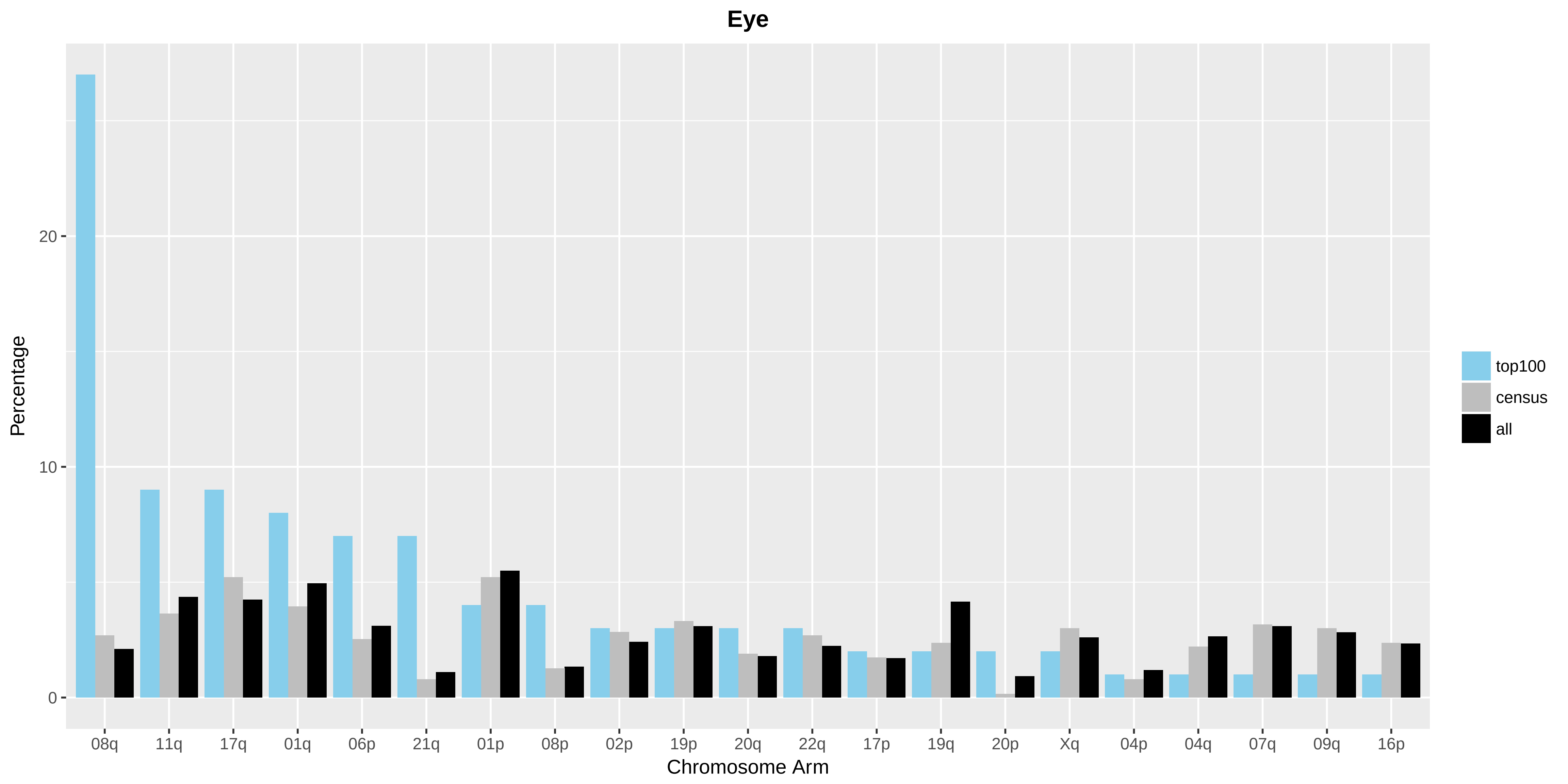

Eye
Percentage
0
10
20
08q
11q
17q
01q
06p
21q
01p
08p
02p
19p
20q
22q
17p
19q
20p
Xq
04p
04q
07q
09q
16p
Chromosome Arm
top100
census
all

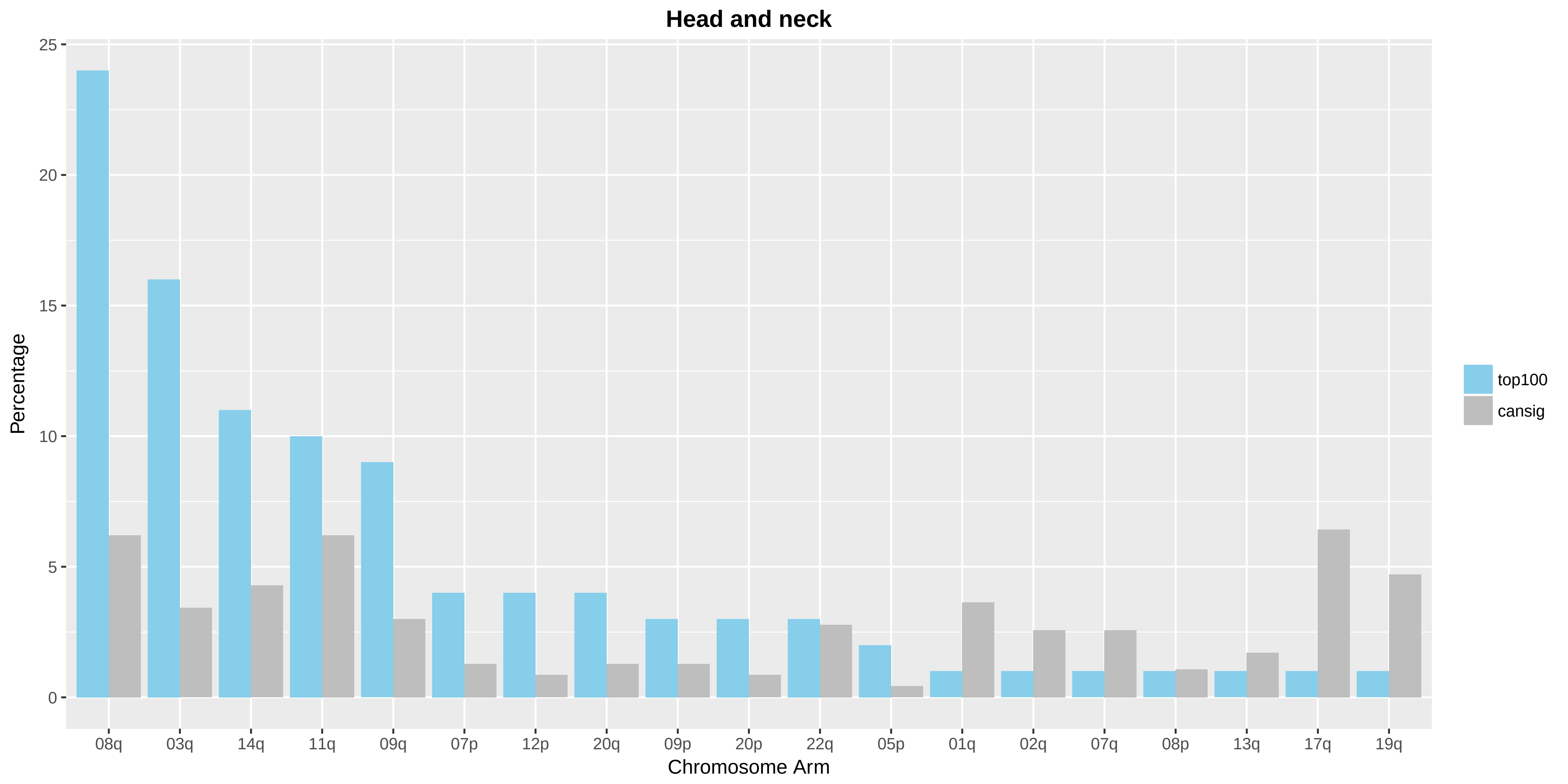

Head and neck
Percentage
0
5
10
15
20
25
08q
03q
14q
11q
09q
07p
12p
20q
09p
20p
22q
05p
01q
02q
07q
08p
13q
17q
19q
Chromosome Arm
top100
cansig

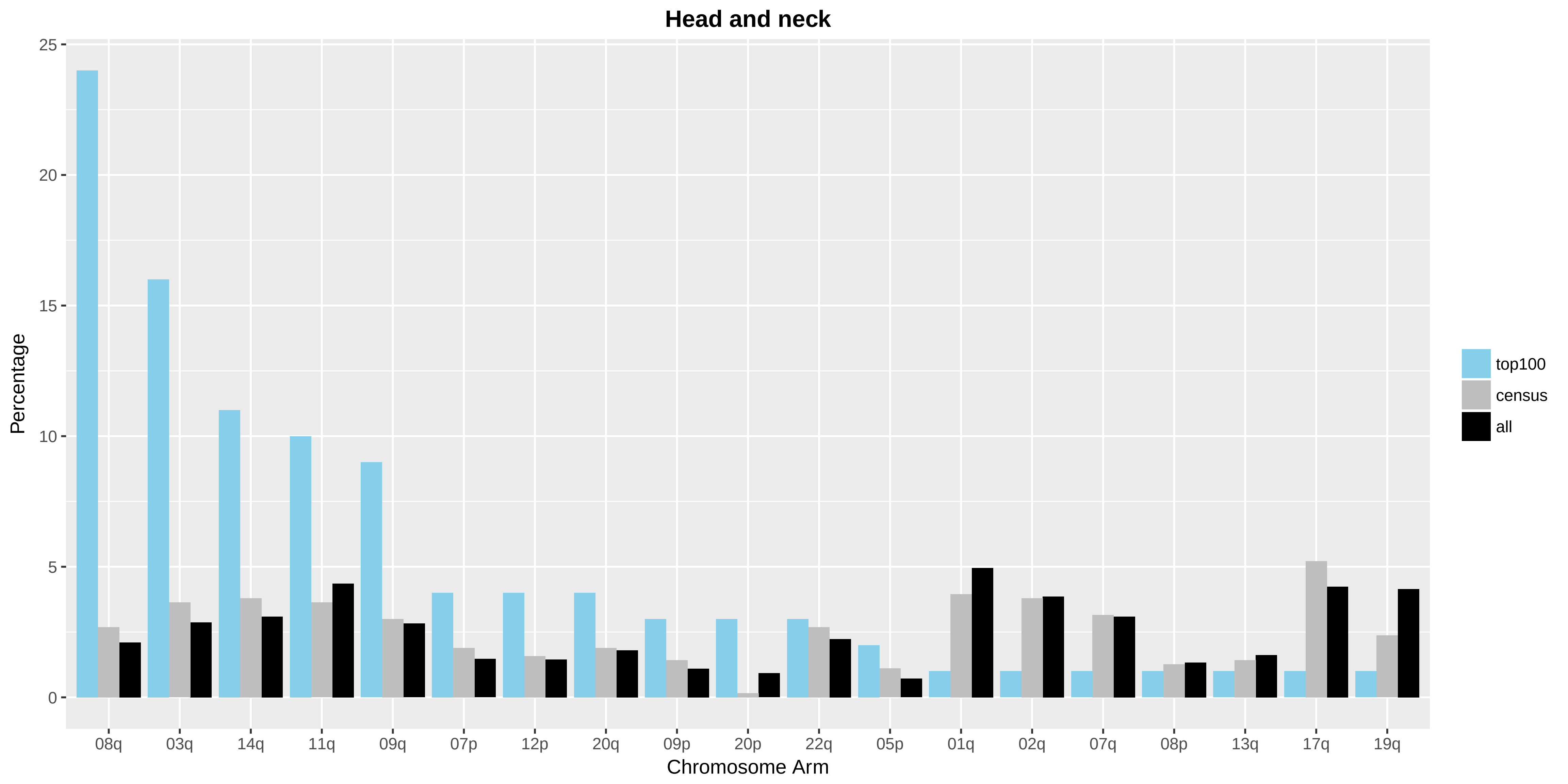
Head and neck
Percentage
25
20
15
10
5
0
08q
03q
14q
11q
09q
07p
12p
20q
09p
20p
22q
05p
01q
02q
07q
08p
13q
17q
19q
Chromosome Arm
top100
census
all

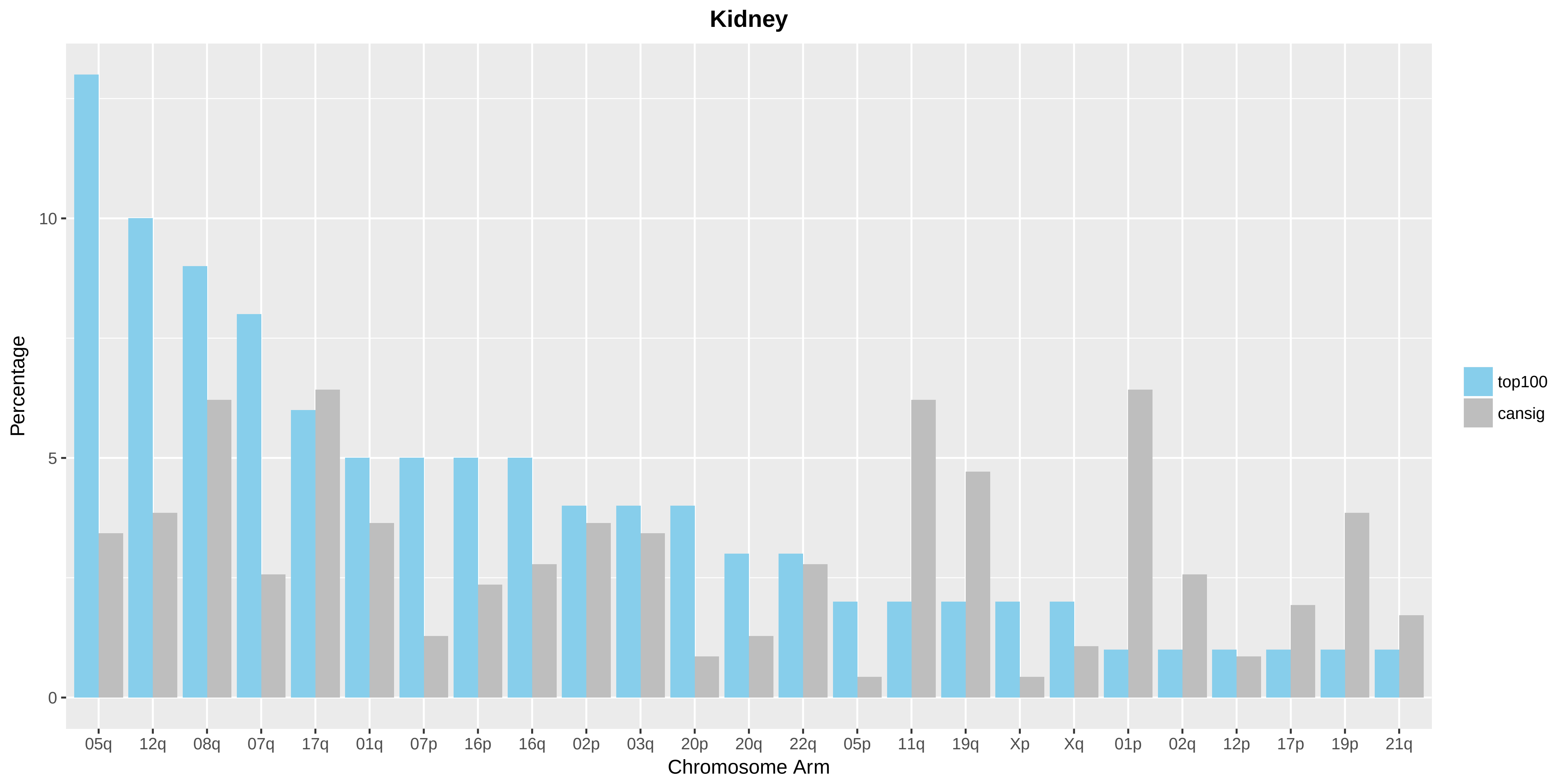

Kidney
Percentage
0
5
10
05q
12q
08q
07q
17q
01q
07p
16p
16q
02p
03q
20p
20q
22q
05p
11q
19q
Xp
Xq
01p
02q
12p
17p
19p
21q
Chromosome Arm
top100
cansig

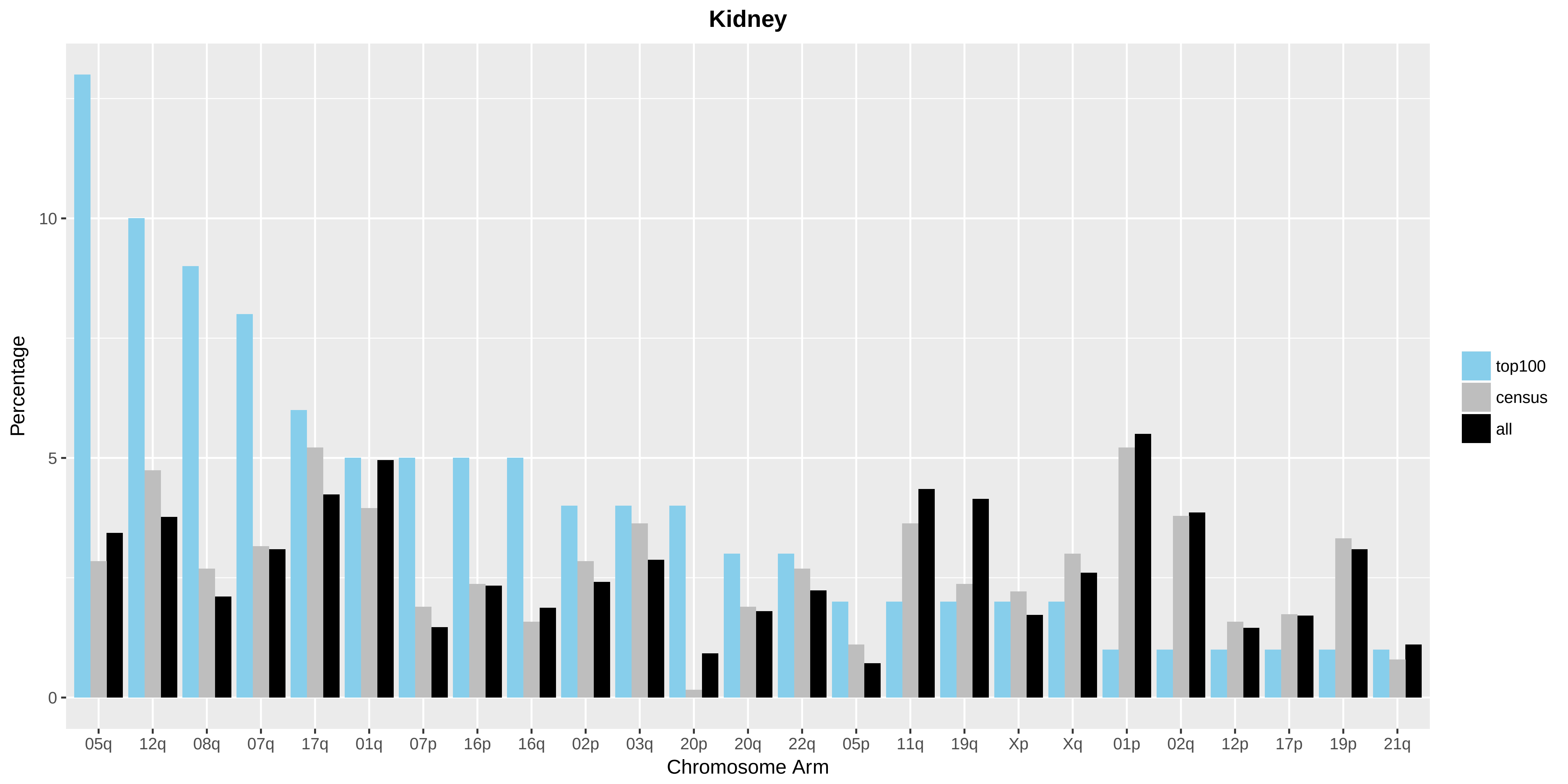
Kidney
Percentage
0
5
10
05q
12q
08q
07q
17q
01q
07p
16p
16q
02p
03q
20p
20q
22q
05p
11q
19q
Xp
Xq
01p
02q
12p
17p
19p
21q
Chromosome Arm
top100
census
all

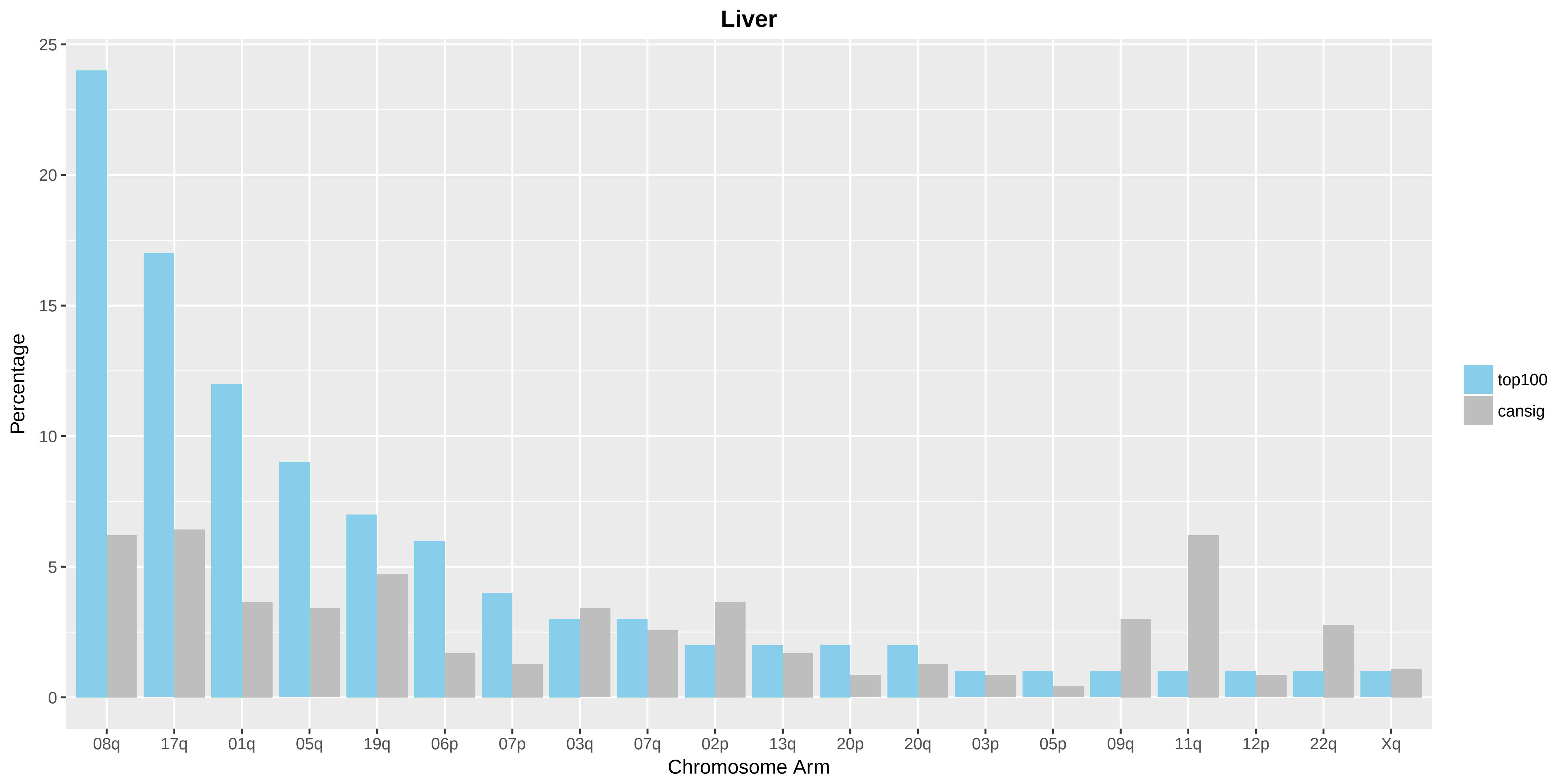
Liver
Percentage
0
5
10
15
20
25
08q
17q
01q
05q
19q
06p
07p
03q
07q
02p
13q
20p
20q
03p
05p
09q
11q
12p
22q
Xq
Chromosome Arm
top100
cansig

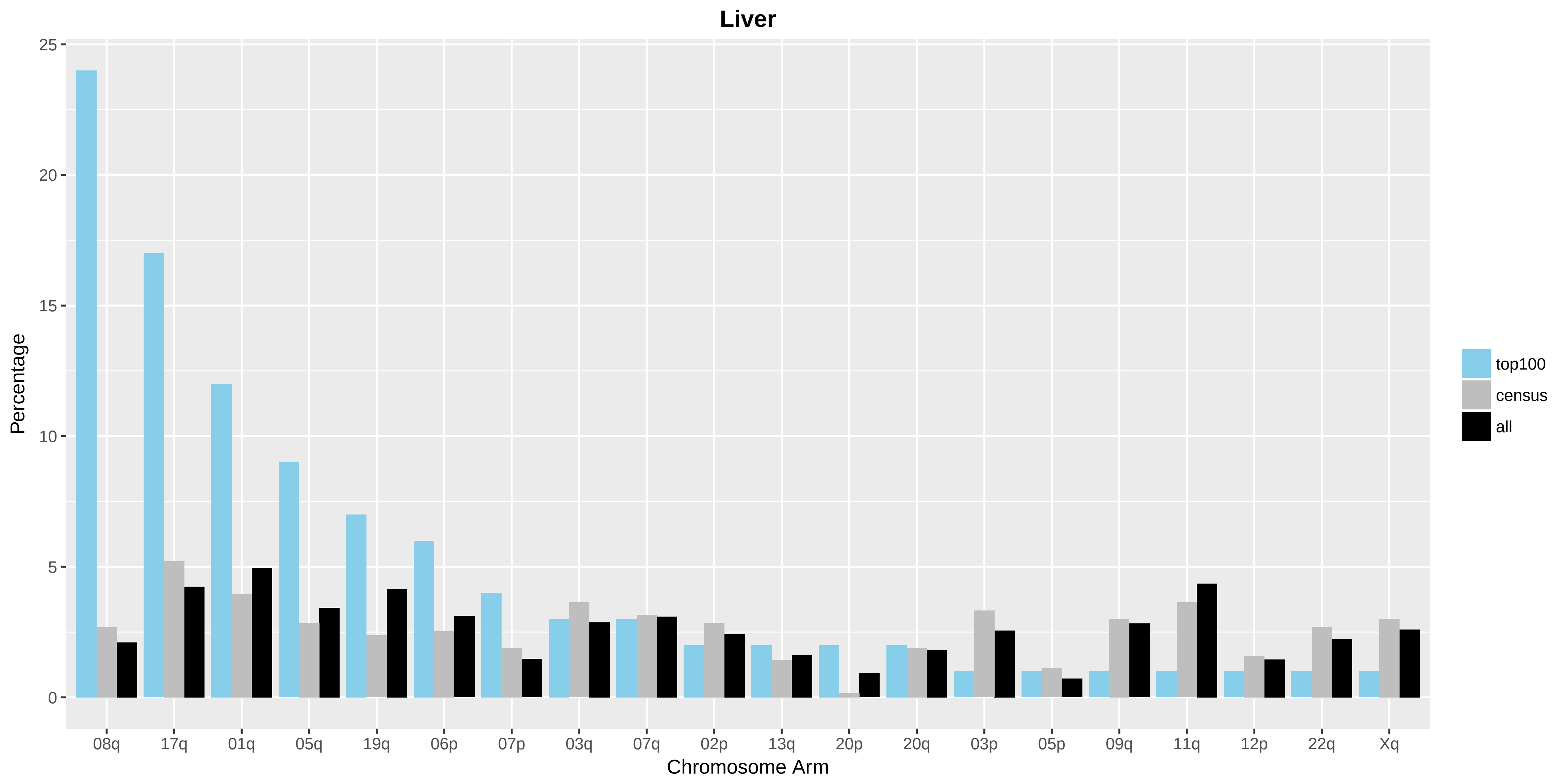
Liver
Percentage
0
5
10
15
20
25
08q
17q
01q
05q
19q
06p
07p
03q
07q
02p
13q
20p
20q
03p
05p
09q
11q
12p
22q
Xq
Chromosome Arm
top100
census
all

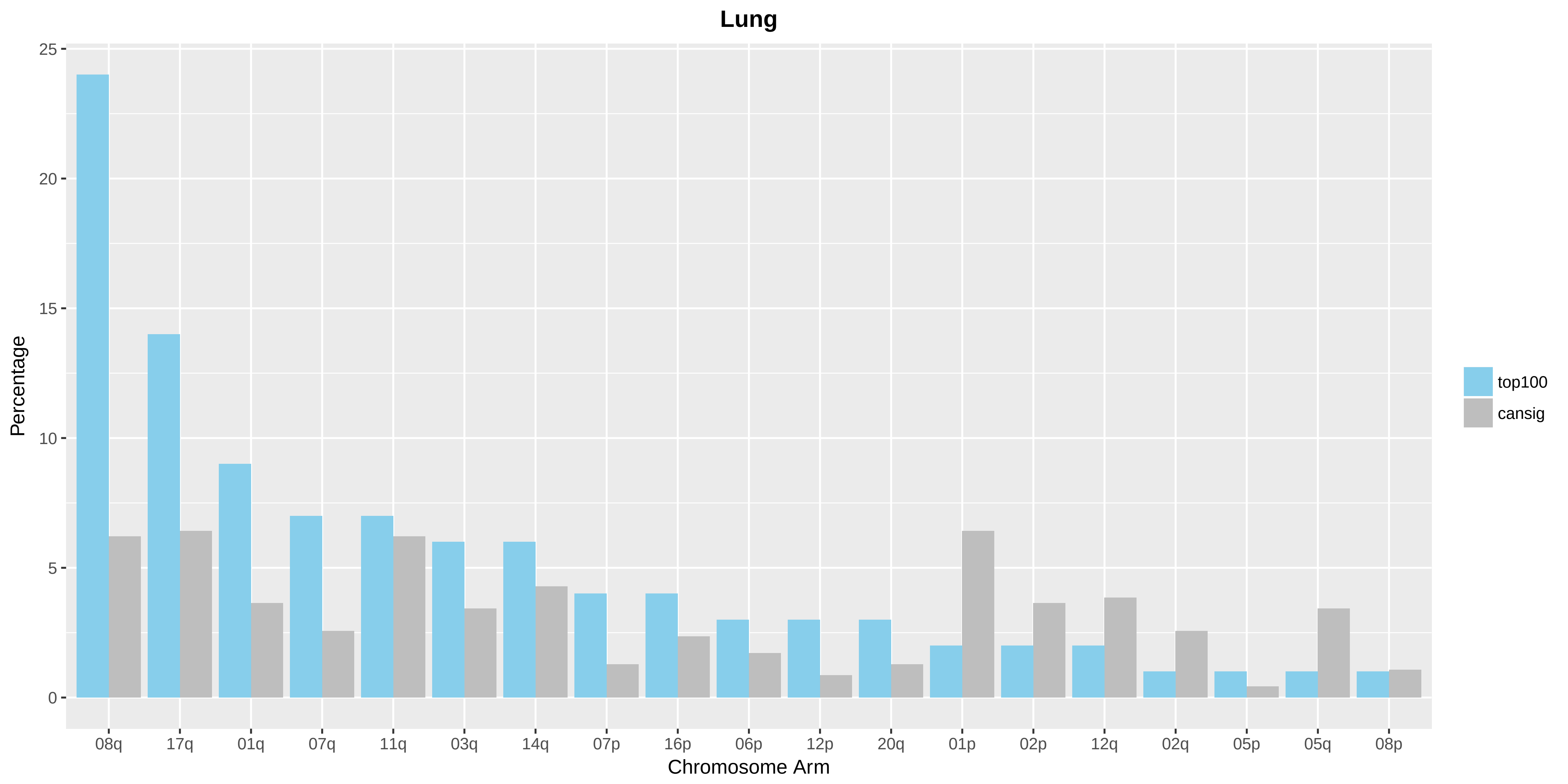
Lung
Percentage
0
5
10
15
20
25
08q
17q
01q
07q
11q
03q
14q
07p
16p
06p
12p
20q
01p
02p
12q
02q
05p
05q
08p
Chromosome Arm
top100
cansig

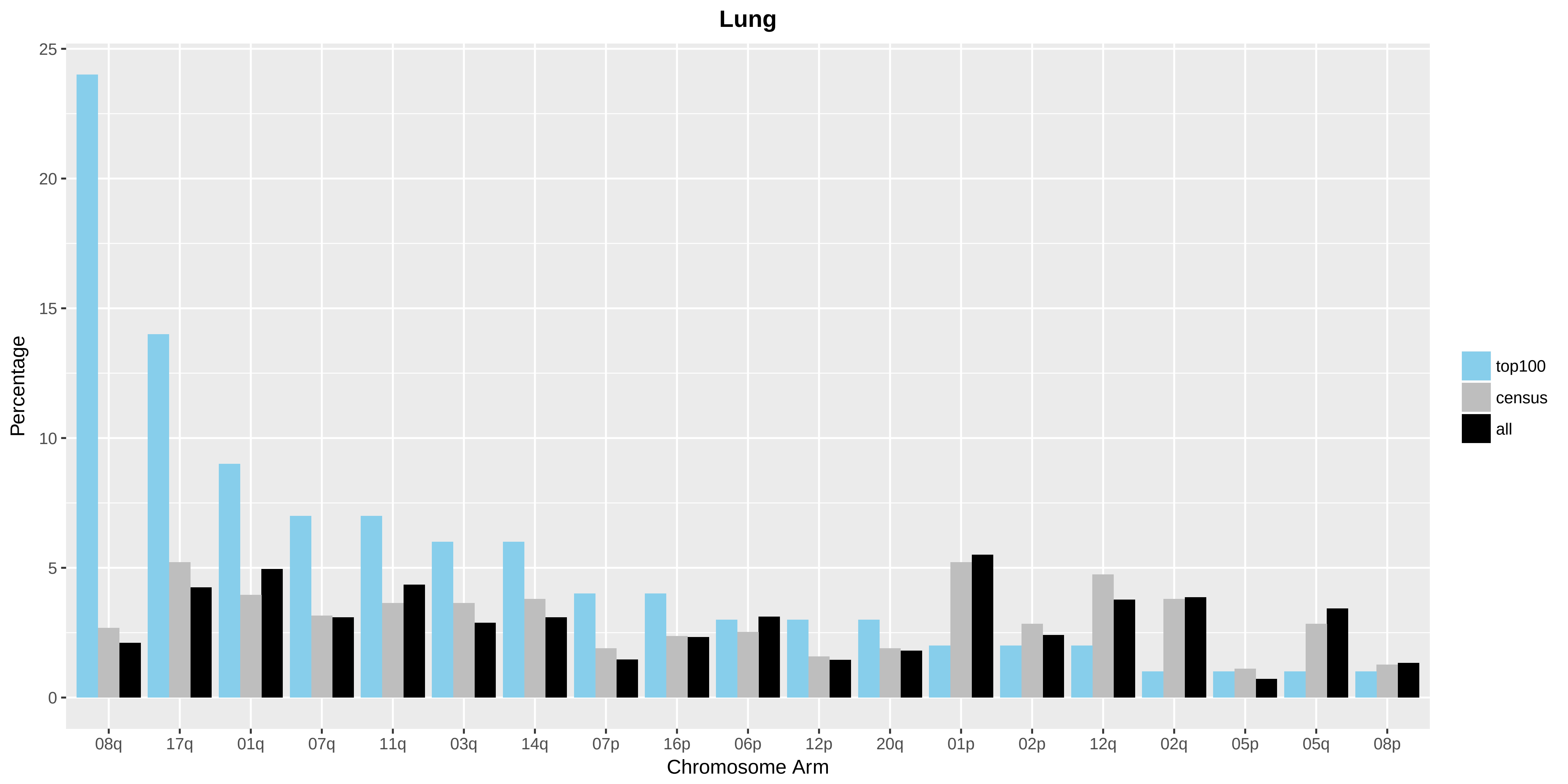
Lung
Percentage
0
5
10
15
20
25
08q
17q
01q
07q
11q
03q
14q
07p
16p
06p
12p
20q
01p
02p
12q
02q
05p
05q
08p
Chromosome Arm
top100
census
all

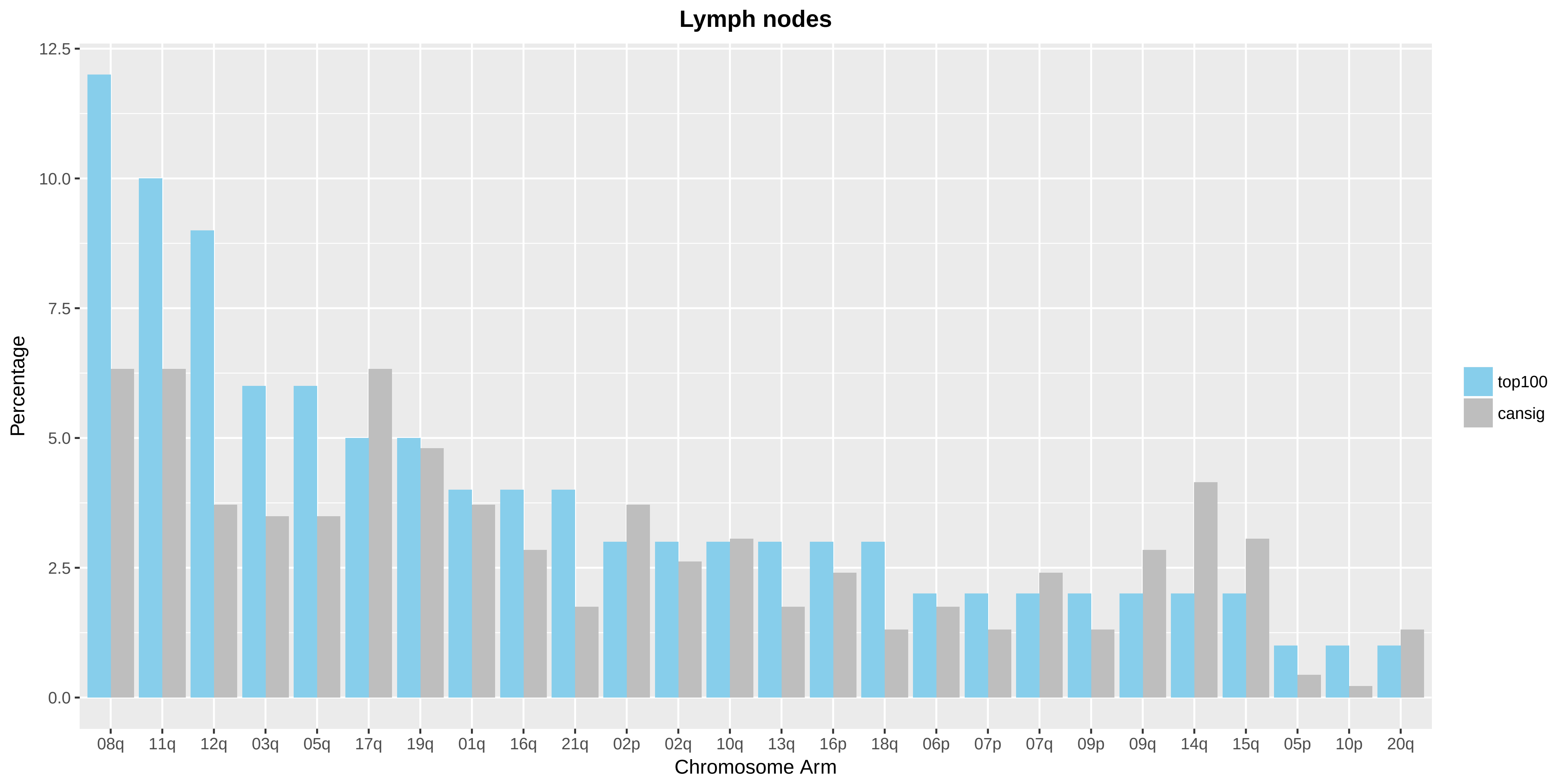

Lymph nodes
Percentage
12.5
10.0
7.5
5.0
2.5
0.0
08q
11q
12q
03q
05q
17q
19q
01q
16q
21q
02p
02q
10q
13q
16p
18q
06p
07p
07q
09p
09q
14q
15q
05p
10p
20q
Chromosome Arm
top100
cansig

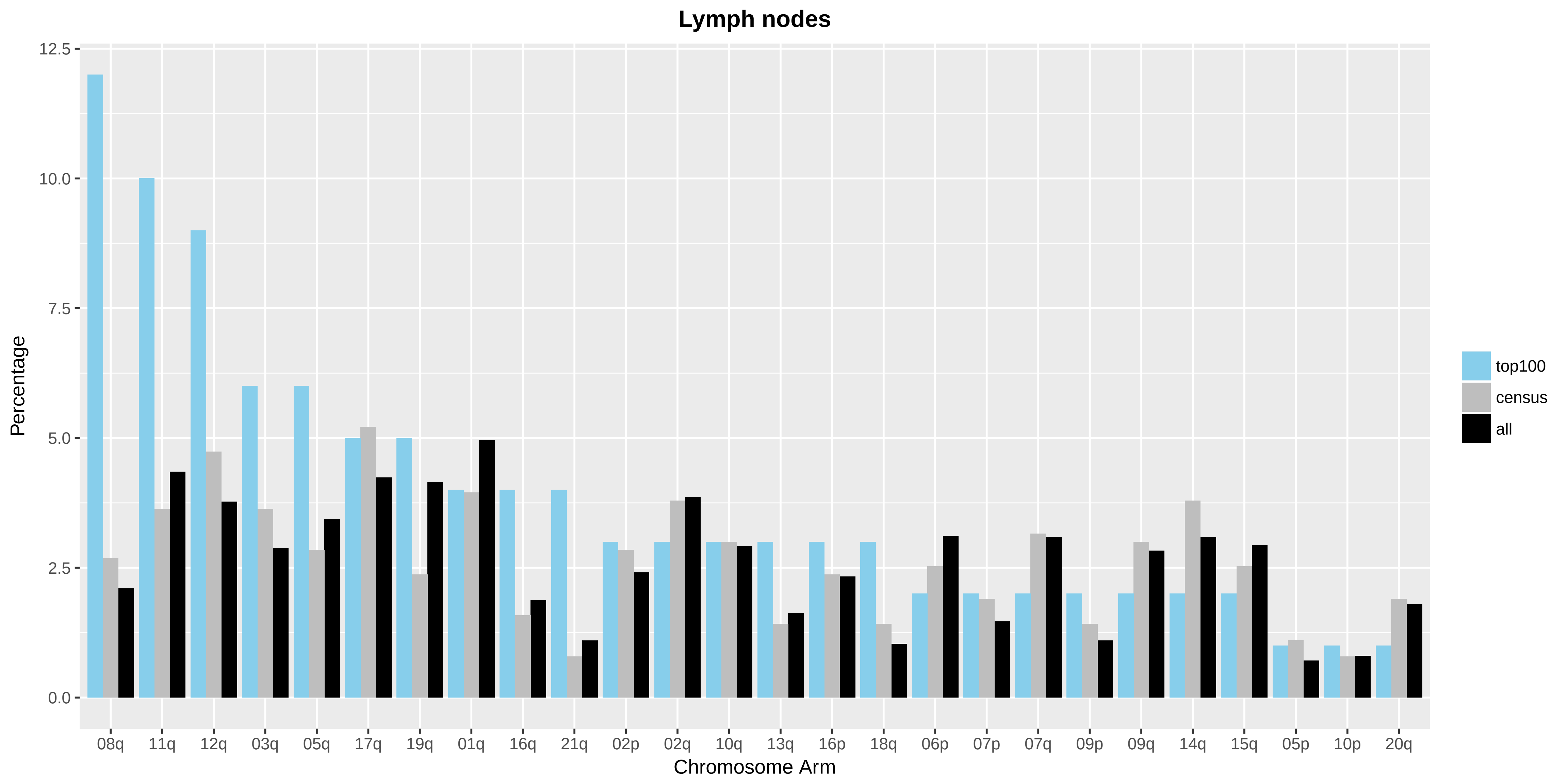

Lymph nodes
Percentage
Chromosome Arm
12.5
10.0
7.5
5.0
2.5
0.0
08q
11q
12q
03q
05q
17q
19q
01q
16q
21q
02p
02q
10q
13q
16p
18q
06p
07p
07q
09p
09q
14q
15q
05p
10p
20q
top100
census
all

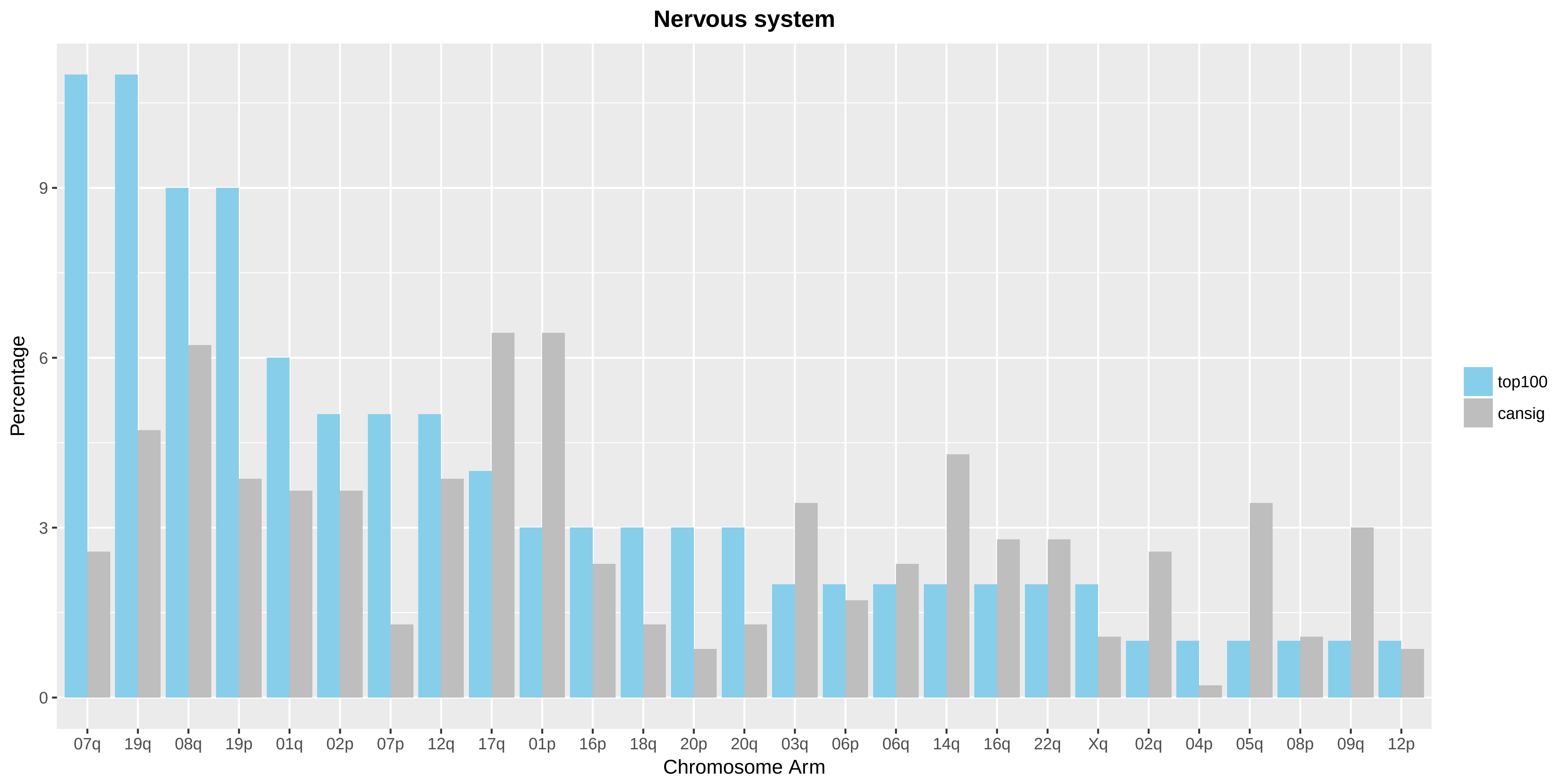

Nervous system
Percentage
Chromosome Arm
0
3
6
9
07q
19q
08q
19p
01q
02p
07p
12q
17q
01p
16p
18q
20p
20q
03q
06p
06q
14q
16q
22q
Xq
02q
04p
05q
08p
09q
12p
top100
cansig

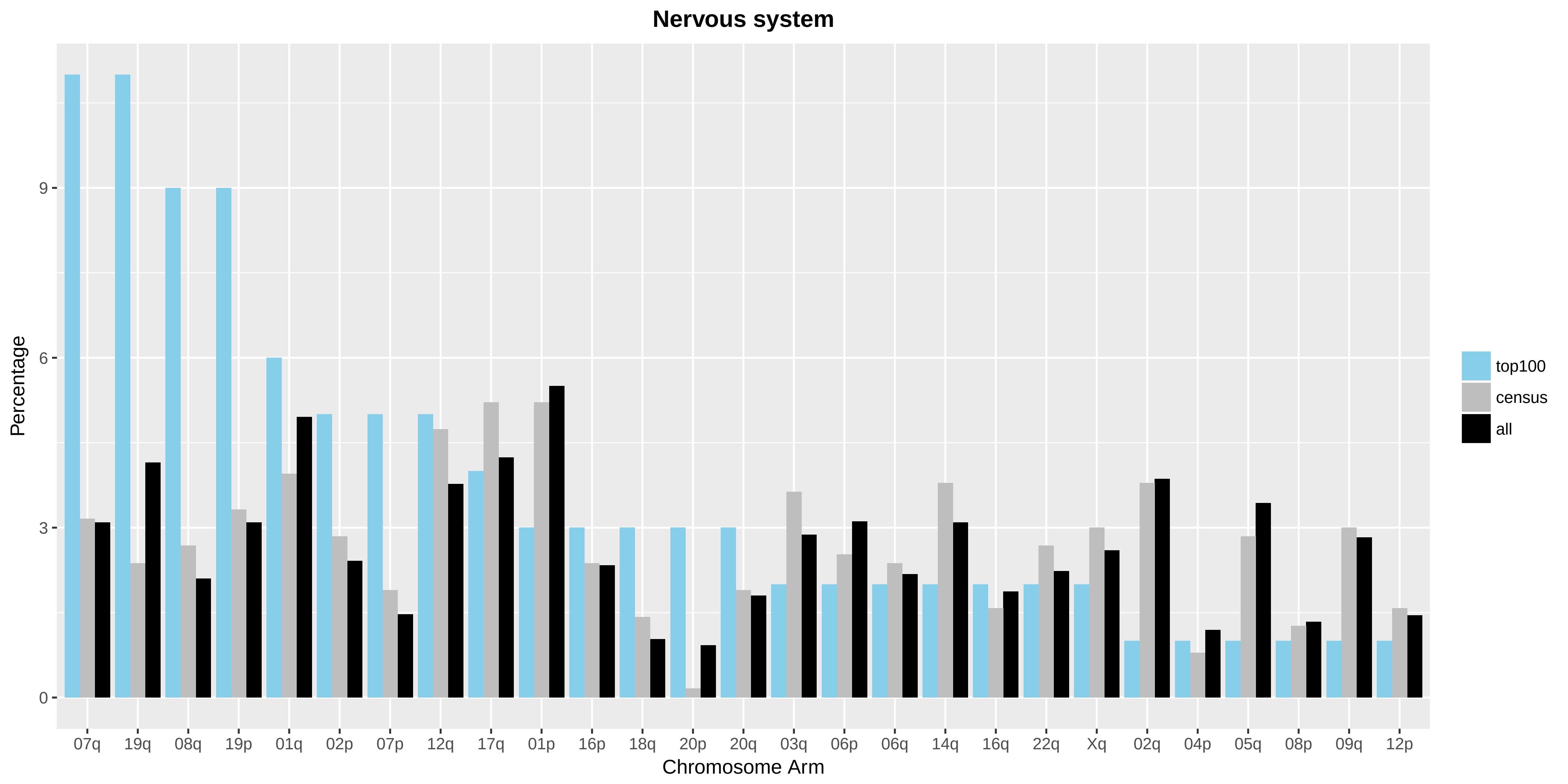

Nervous system
Percentage
0
3
6
9
07q
19q
08q
19p
01q
02p
07p
12q
17q
01p
16p
18q
20p
20q
03q
06p
06q
14q
16q
22q
Xq
02q
04p
05q
08p
09q
12p
Chromosome Arm
top100
census
all

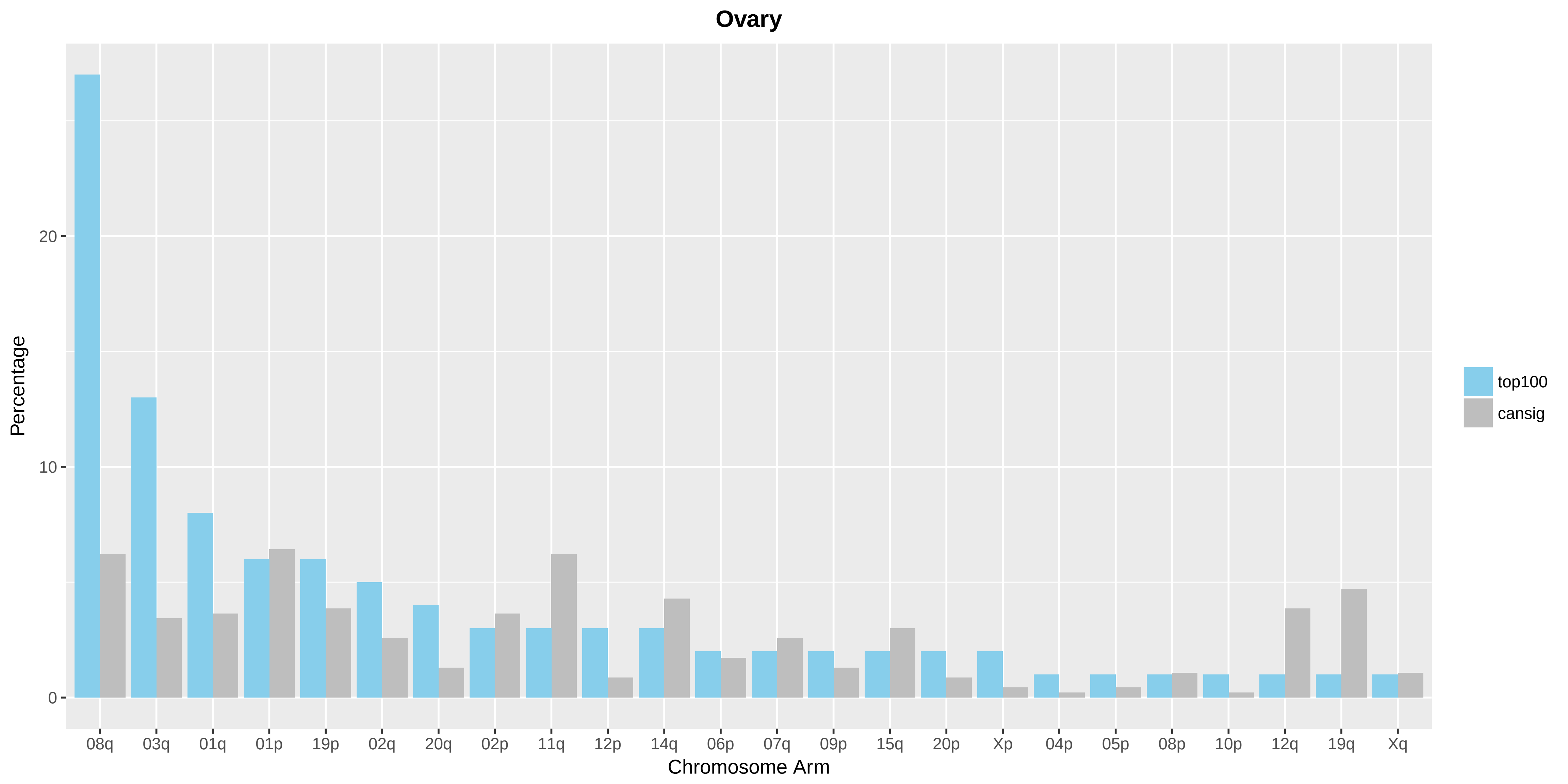

Ovary
Percentage
20
10
0
08q
03q
01q
01p
19p
02q
20q
02p
11q
12p
14q
06p
07q
09p
15q
20p
Xp
04p
05p
08p
10p
12q
19q
Xq
Chromosome Arm
top100
cansig

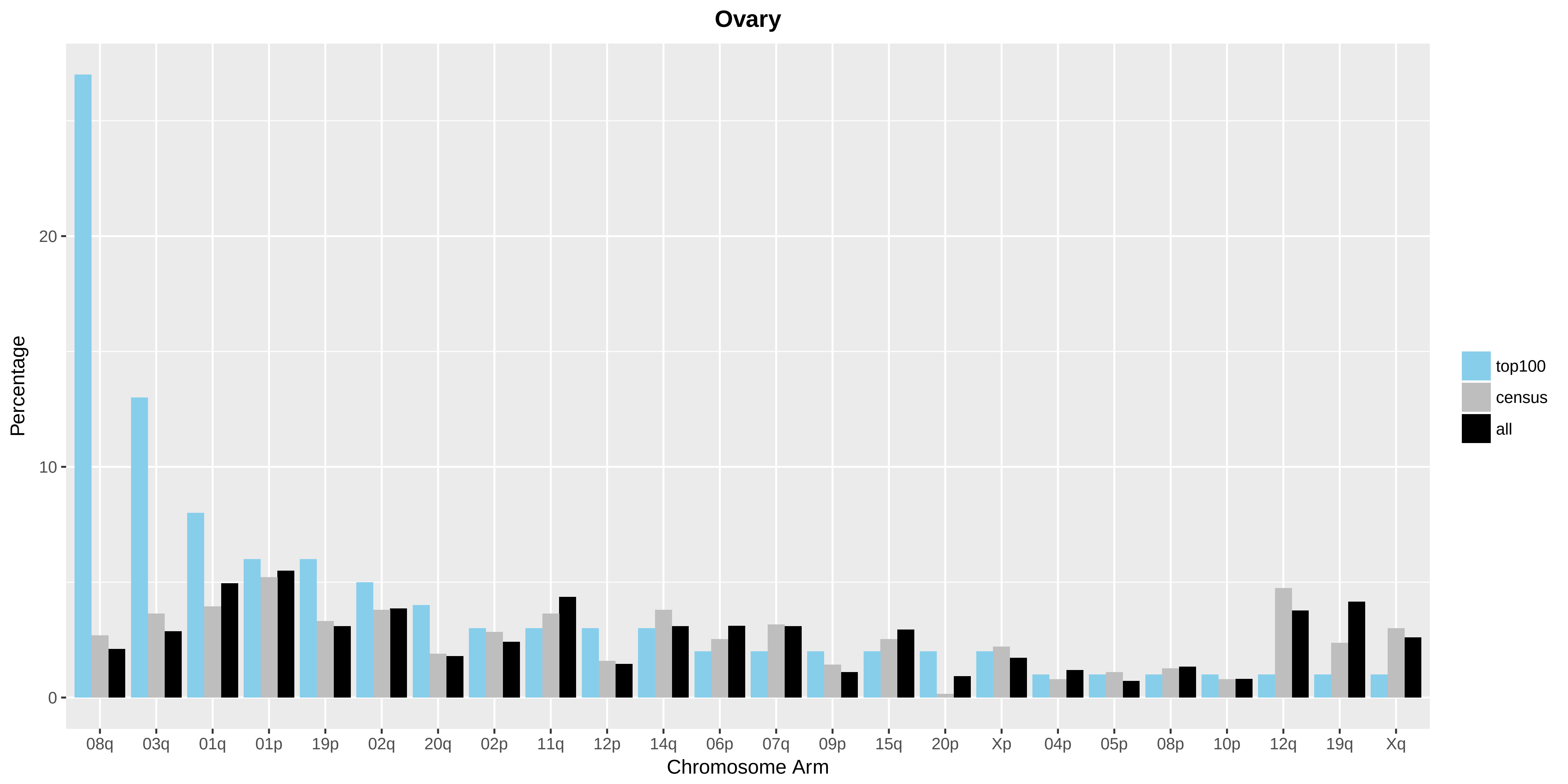

Ovary
Percentage
0
10
20
08q
03q
01q
01p
19p
02q
20q
02p
11q
12p
14q
06p
07q
09p
15q
20p
Xp
04p
05p
08p
10p
12q
19q
Xq
Chromosome Arm
top100
census
all

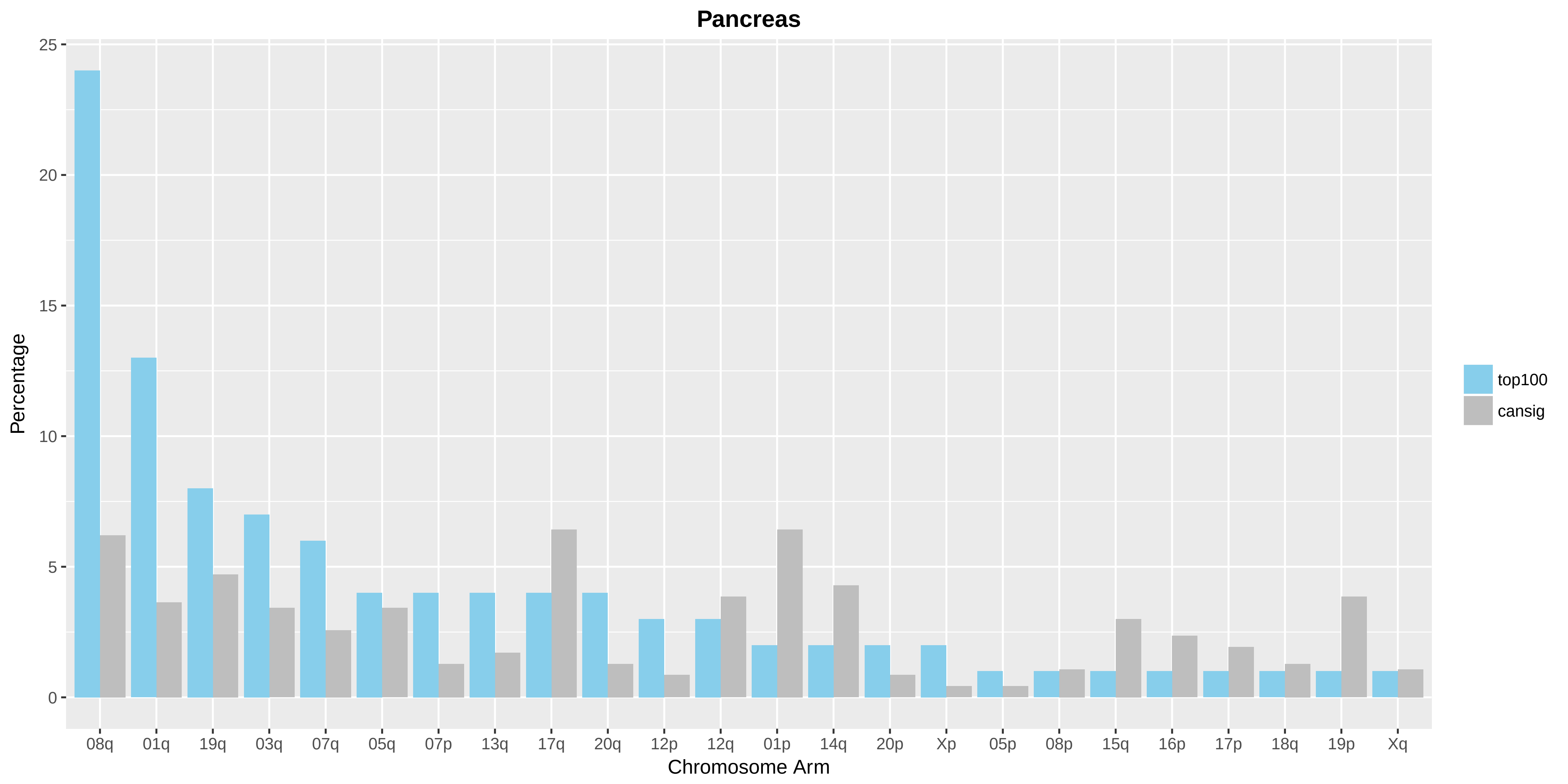

Pancreas
Percentage
25
20
15
10
5
0
08q
01q
19q
03q
07q
05q
07p
13q
17q
20q
12p
12q
01p
14q
20p
Xp
05p
08p
15q
16p
17p
18q
19p
Xq
Chromosome Arm
top100
cansig

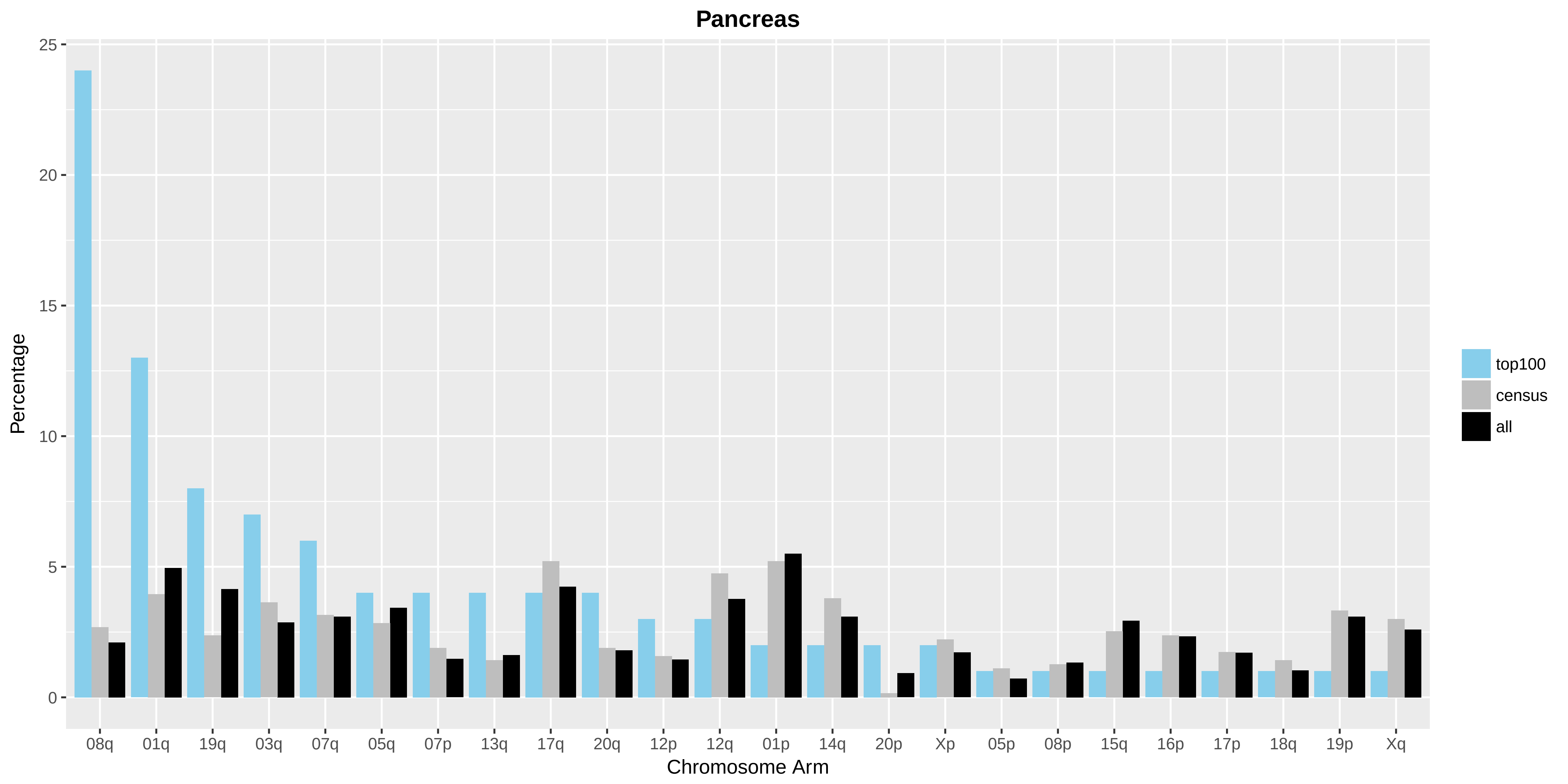
Pancreas
Percentage
Chromosome Arm
0
5
10
15
20
25
08q
01q
19q
03q
07q
05q
07p
13q
17q
20q
12p
12q
01p
14q
20p
Xp
05p
08p
15q
16p
17p
18q
19p
Xq
top100
census
all

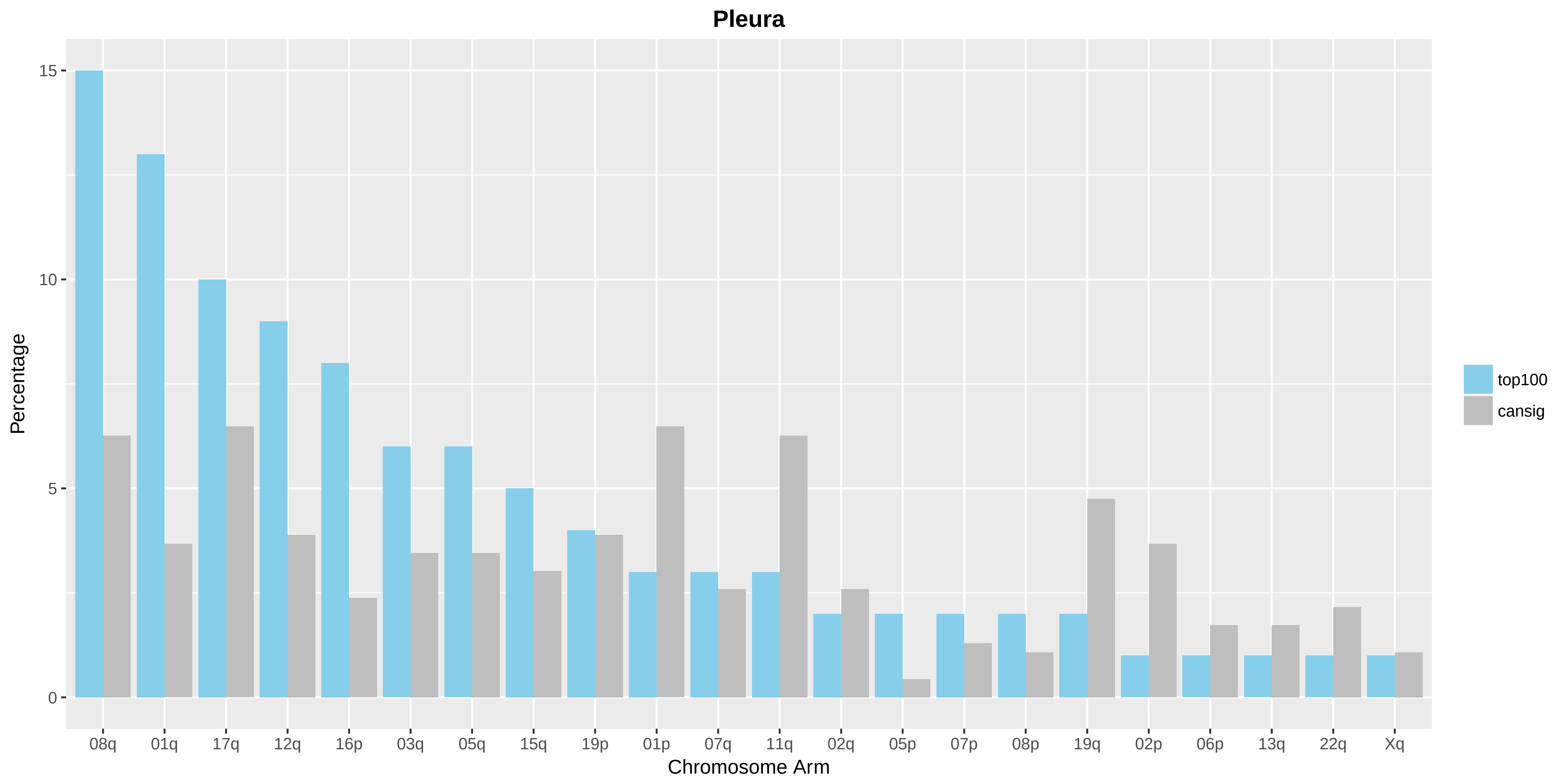

Pleura
Percentage
15
10
5
0
08q
01q
17q
12q
16p
03q
05q
15q
19p
01p
07q
11q
02q
05p
07p
08p
19q
02p
06p
13q
22q
Xq
Chromosome Arm
top100
cansig

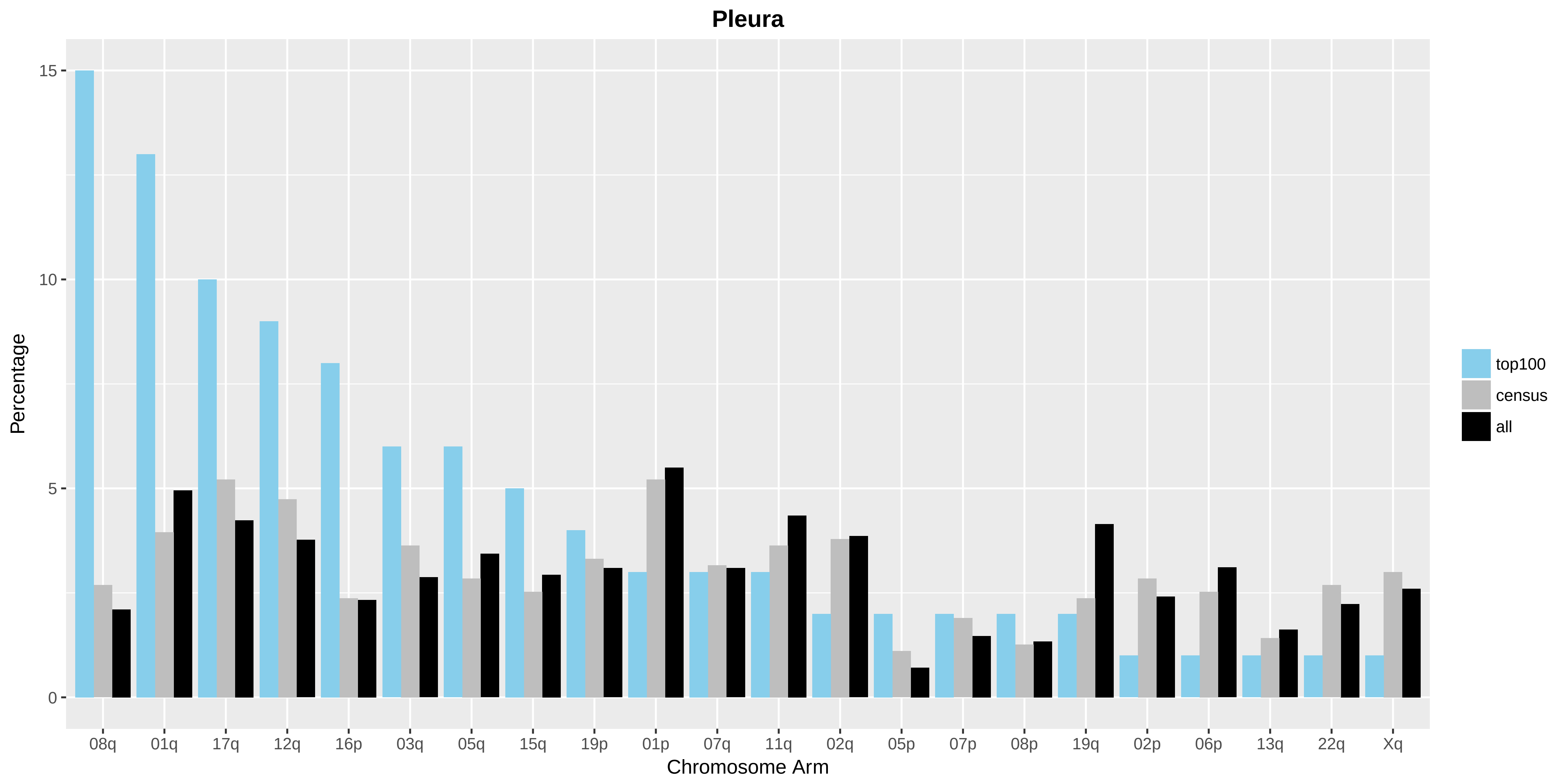

Pleura
Percentage
15
10
5
0
08q
01q
17q
12q
16p
03q
05q
15q
19p
01p
07q
11q
02q
05p
07p
08p
19q
02p
06p
13q
22q
Xq
Chromosome Arm
top100
census
all

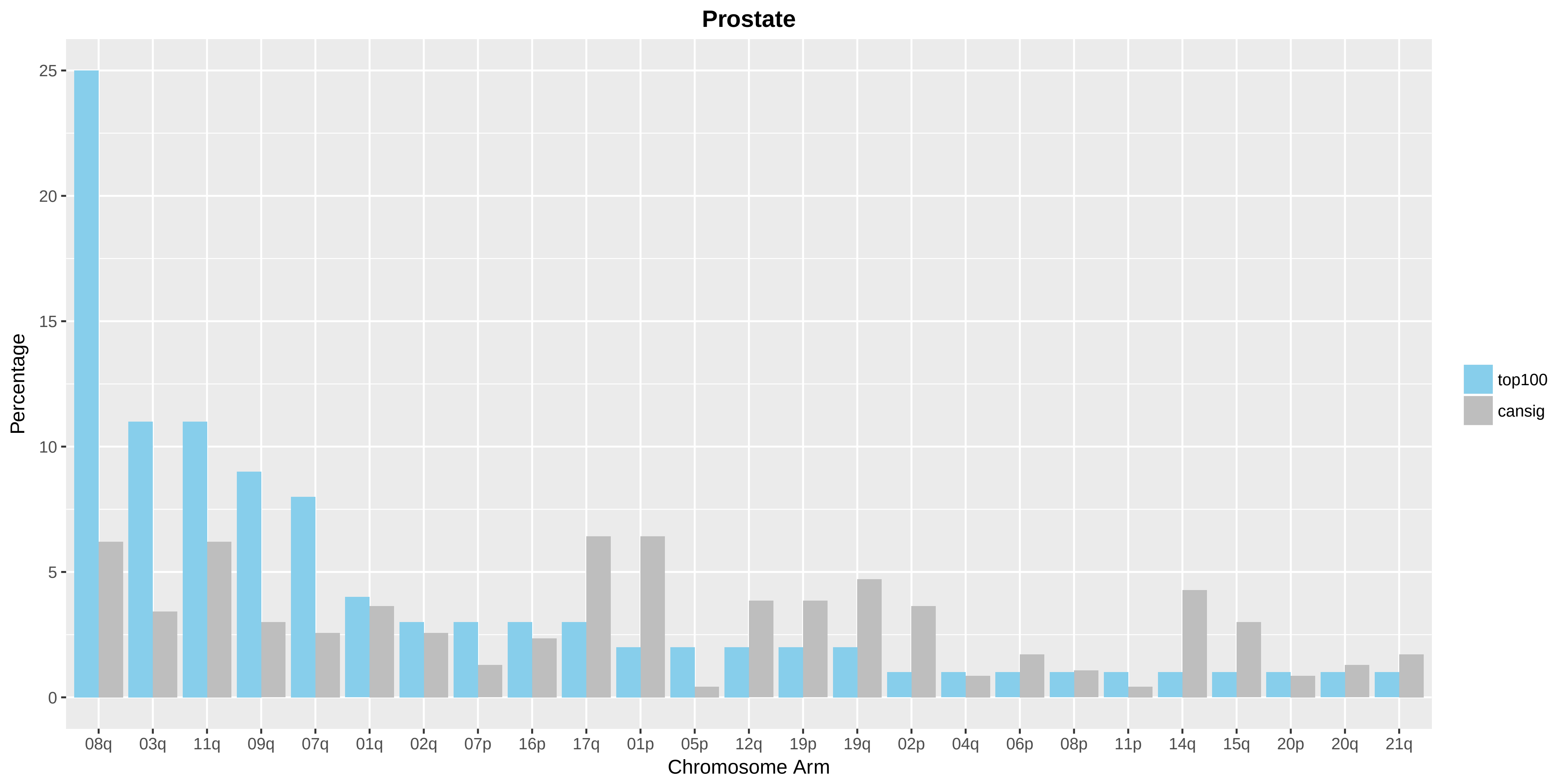

Prostate
Percentage
0
5
10
15
20
25
08q
03q
11q
09q
07q
01q
02q
07p
16p
17q
01p
05p
12q
19p
19q
02p
04q
06p
08p
11p
14q
15q
20p
20q
21q
Chromosome Arm
top100
cansig

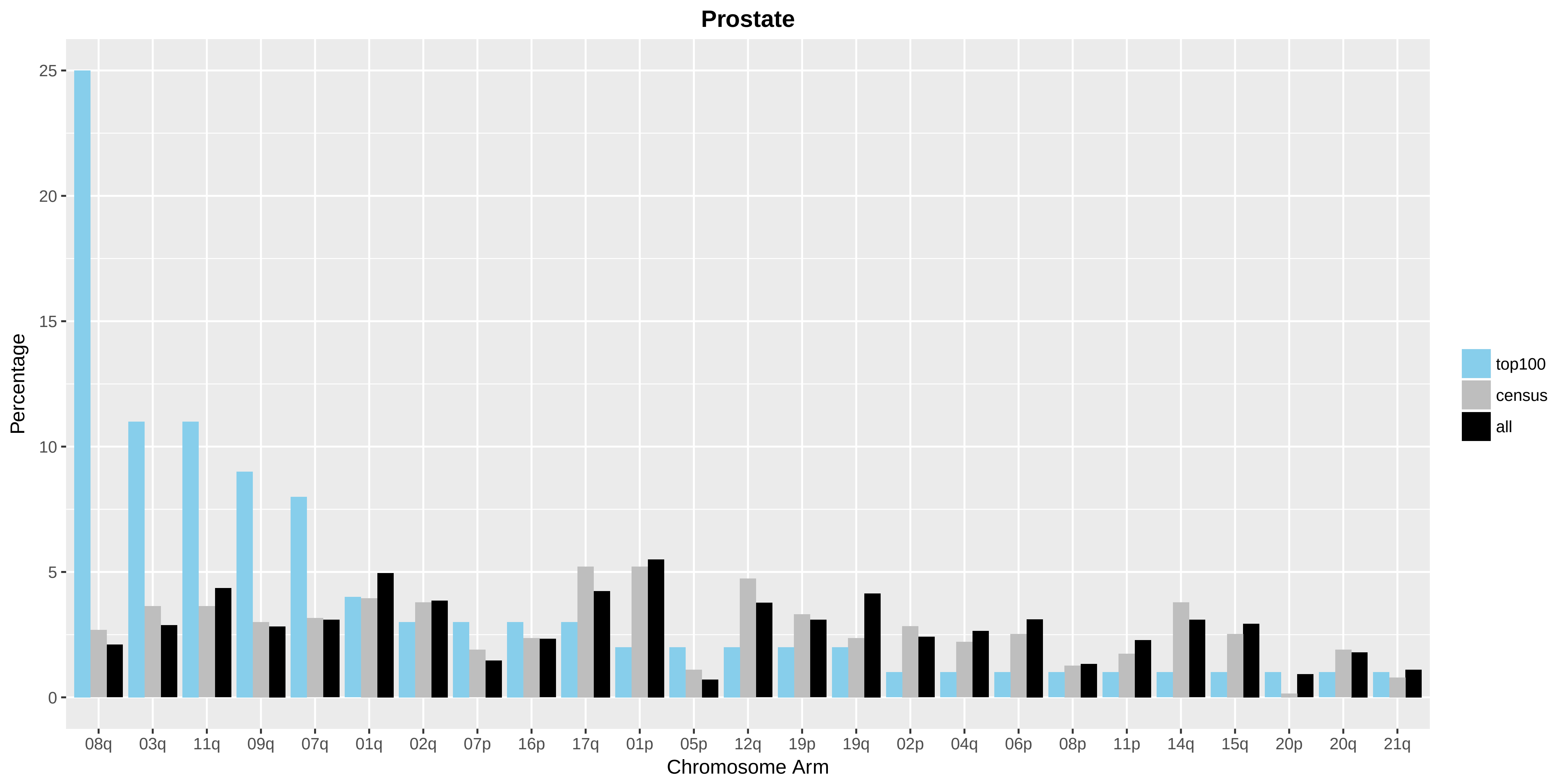

Prostate
Percentage
Chromosome Arm
0
5
10
15
20
25
08q
03q
11q
09q
07q
01q
02q
07p
16p
17q
01p
05p
12q
19p
19q
02p
04q
06p
08p
11p
14q
15q
20p
20q
21q
top100
census
all

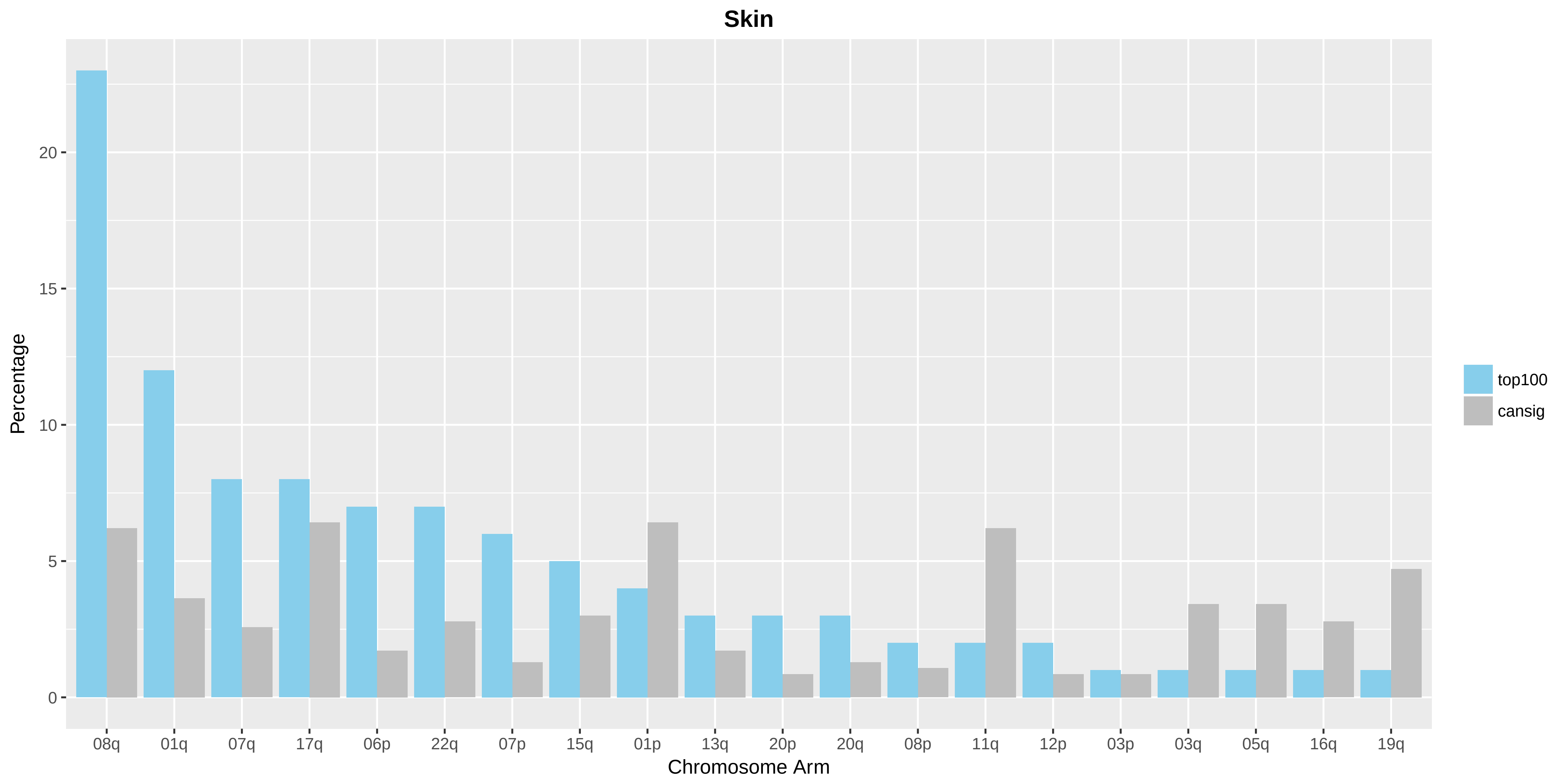

Skin
Percentage
0
5
10
15
20
08q
01q
07q
17q
06p
22q
07p
15q
01p
13q
20p
20q
08p
11q
12p
03p
03q
05q
16q
19q
Chromosome Arm
top100
cansig

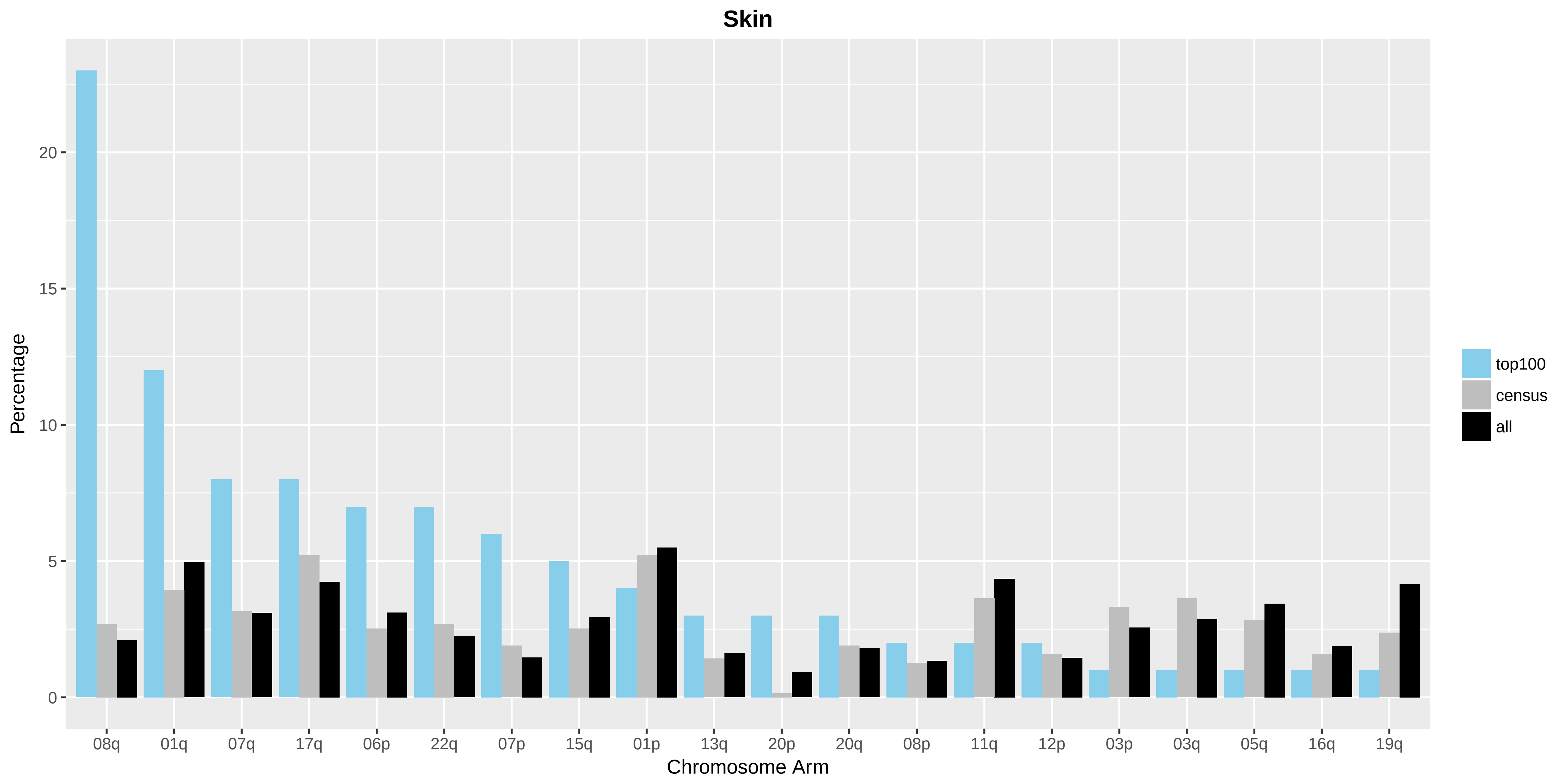
Skin
Percentage
0
5
10
15
20
08q
01q
07q
17q
06p
22q
07p
15q
01p
13q
20p
20q
08p
11q
12p
03p
03q
05q
16q
19q
Chromosome Arm
top100
census
all

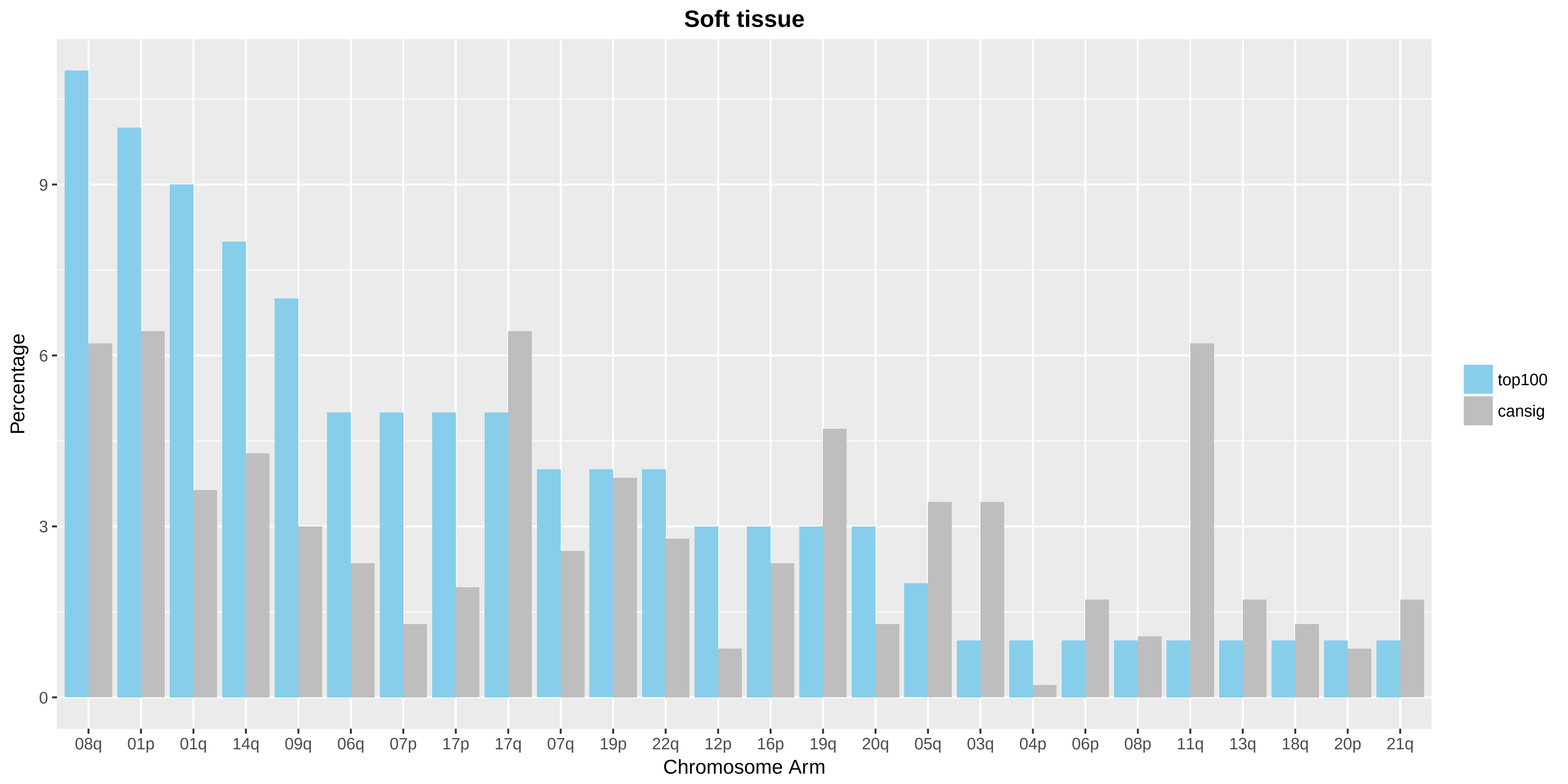

Soft tissue
Percentage
0
3
6
9
08q
01p
01q
14q
09q
06q
07p
17p
17q
07q
19p
22q
12p
16p
19q
20q
05q
03q
04p
06p
08p
11q
13q
18q
20p
21q
Chromosome Arm
top100
cansig

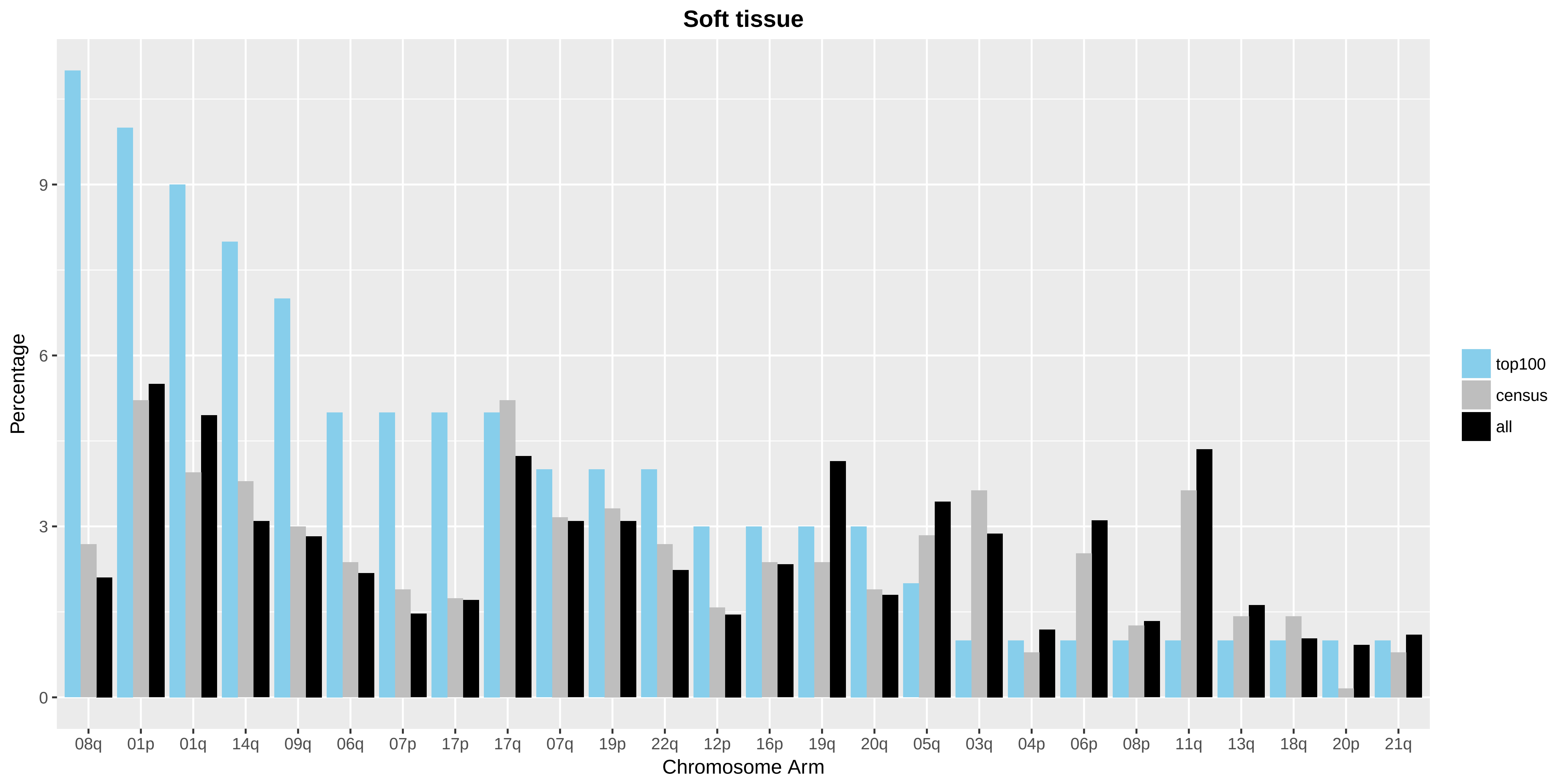
Soft tissue
Percentage
0
3
6
9
08q
01p
01q
14q
09q
06q
07p
17p
17q
07q
19p
22q
12p
16p
19q
20q
05q
03q
04p
06p
08p
11q
13q
18q
20p
21q
Chromosome Arm
top100
census
all

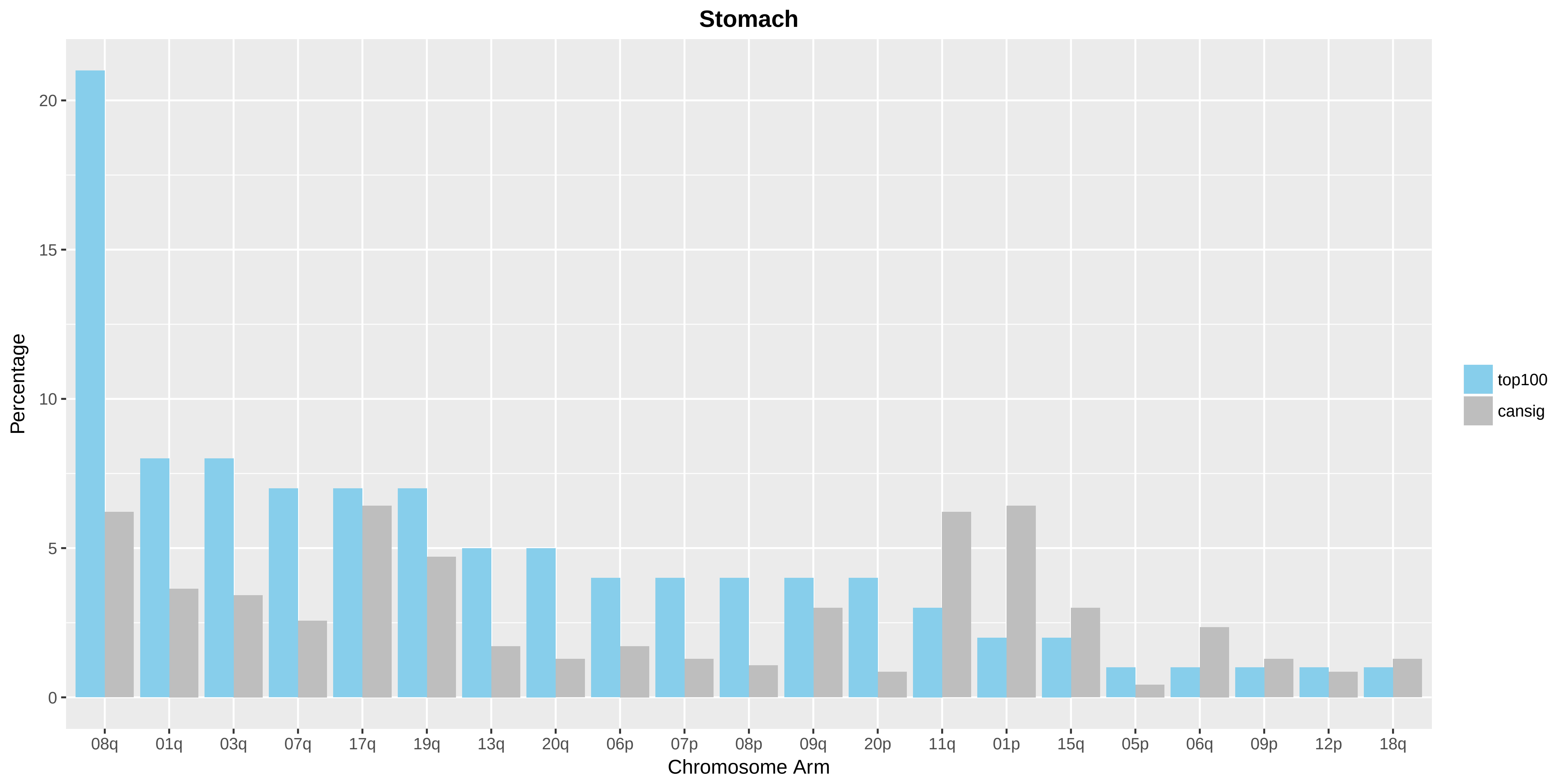

Stomach
Percentage
0
5
10
15
20
08q
01q
03q
07q
17q
19q
13q
20q
06p
07p
08p
09q
20p
11q
01p
15q
05p
06q
09p
12p
18q
Chromosome Arm
top100
cansig

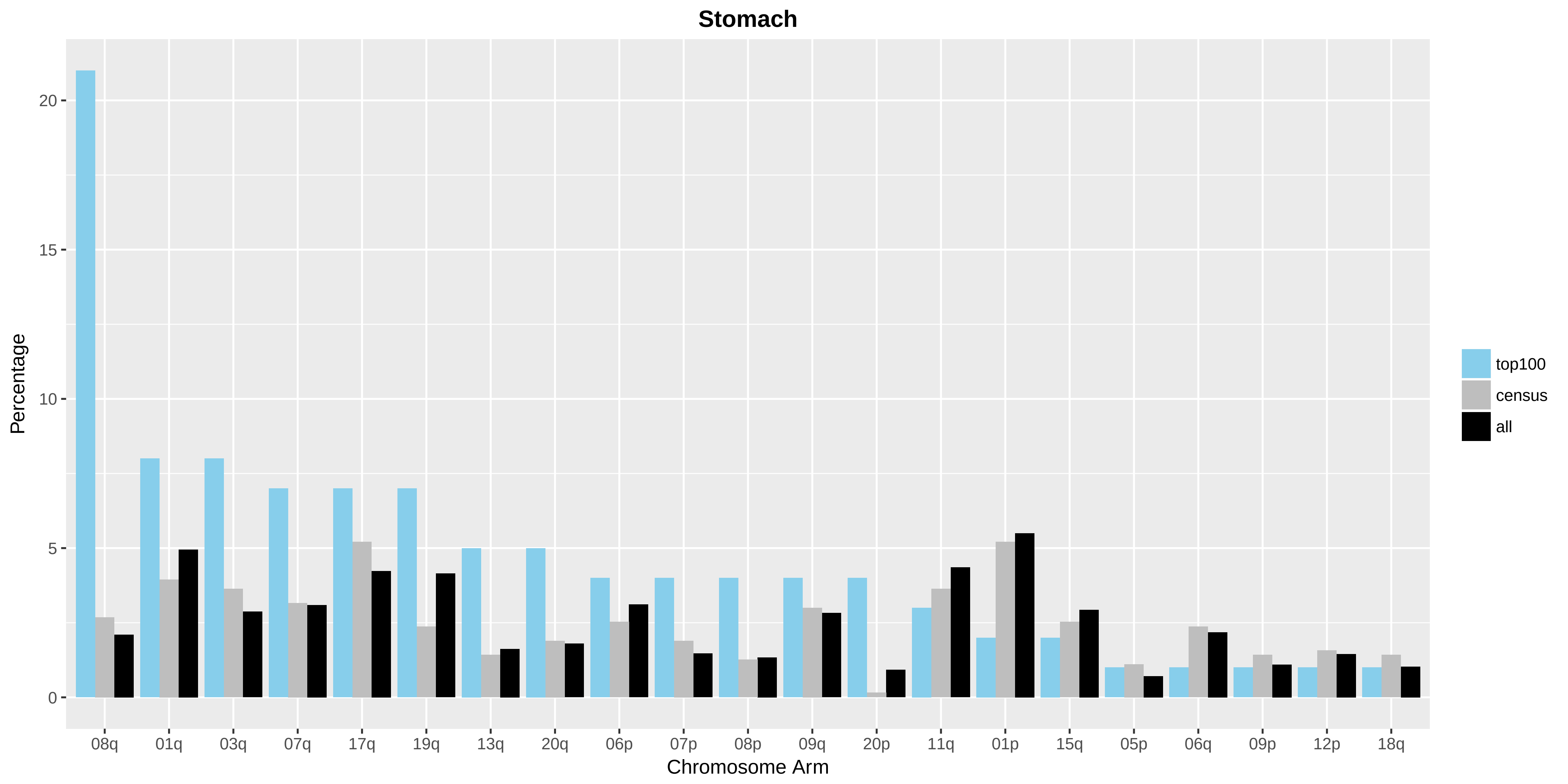

Stomach
Percentage
0
5
10
15
20
08q
01q
03q
07q
17q
19q
13q
20q
06p
07p
08p
09q
20p
11q
01p
15q
05p
06q
09p
12p
18q
Chromosome Arm
top100
census
all

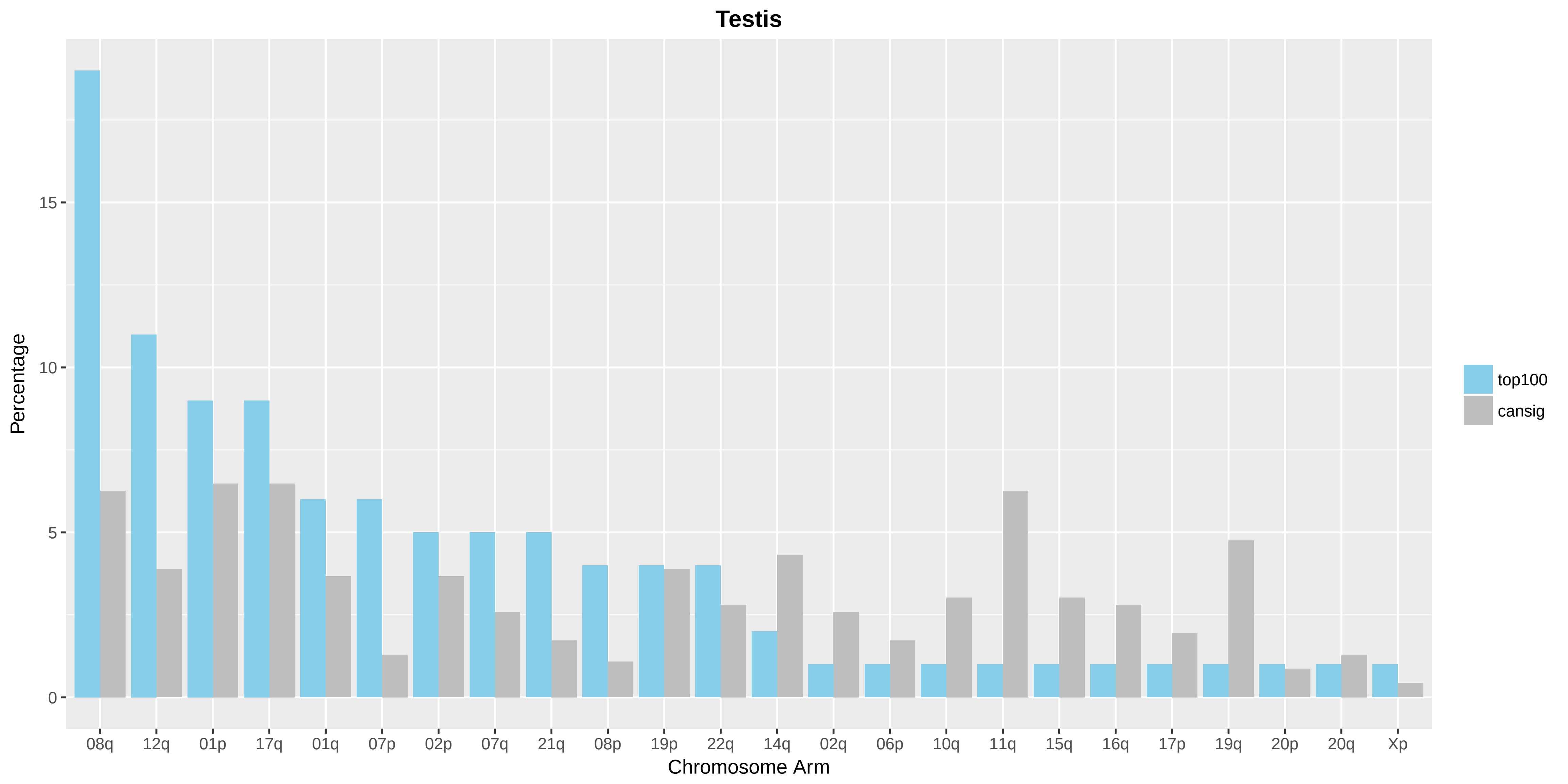
Testis
Percentage
0
5
10
15
08q
12q
01p
17q
01q
07p
02p
07q
21q
08p
19p
22q
14q
02q
06p
10q
11q
15q
16q
17p
19q
20p
20q
Xp
Chromosome Arm
top100
cansig

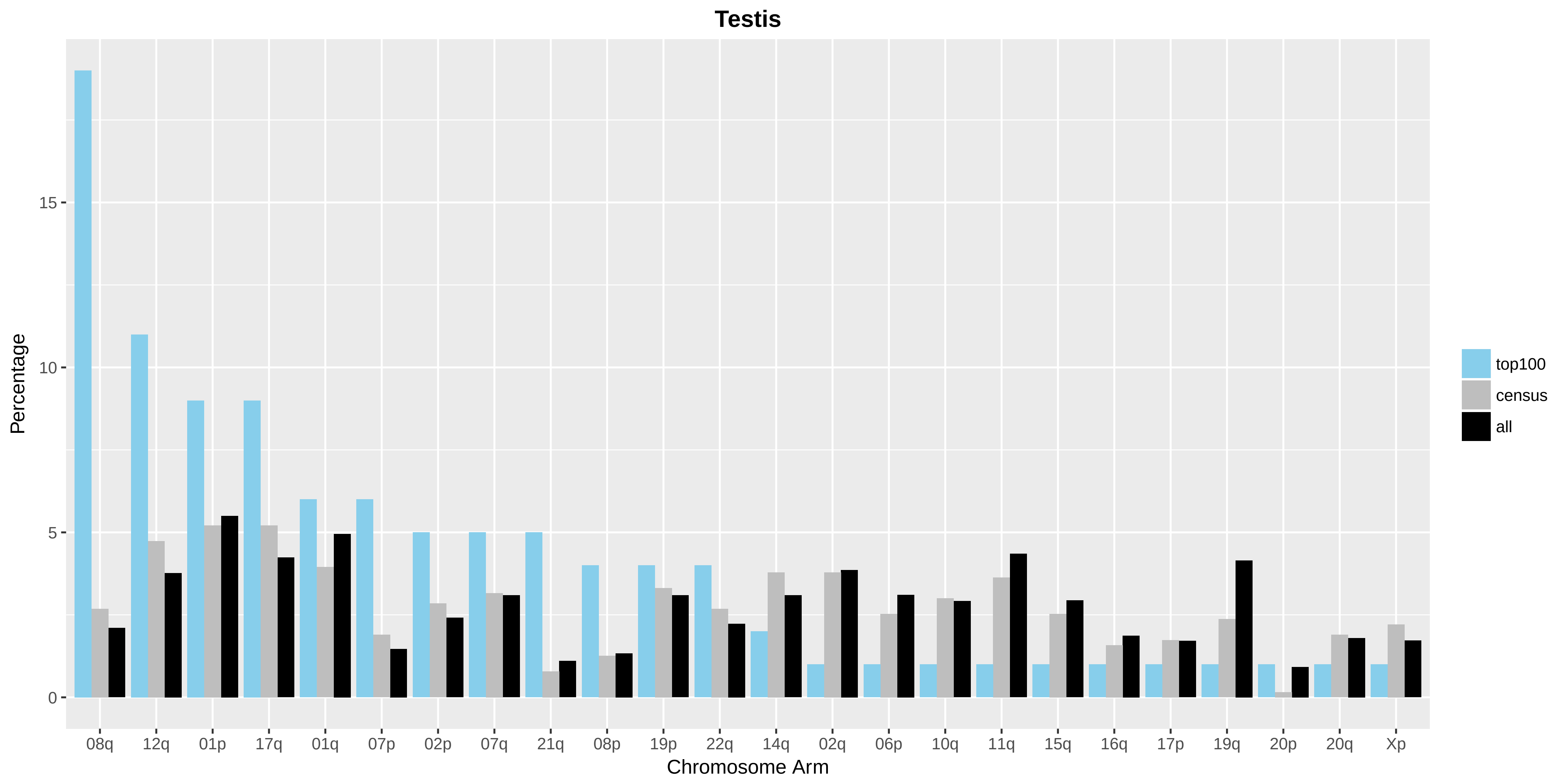
Testis
Percentage
0
5
10
15
08q
12q
01p
17q
01q
07p
02p
07q
21q
08p
19p
22q
14q
02q
06p
10q
11q
15q
16q
17p
19q
20p
20q
Xp
Chromosome Arm
top100
census
all

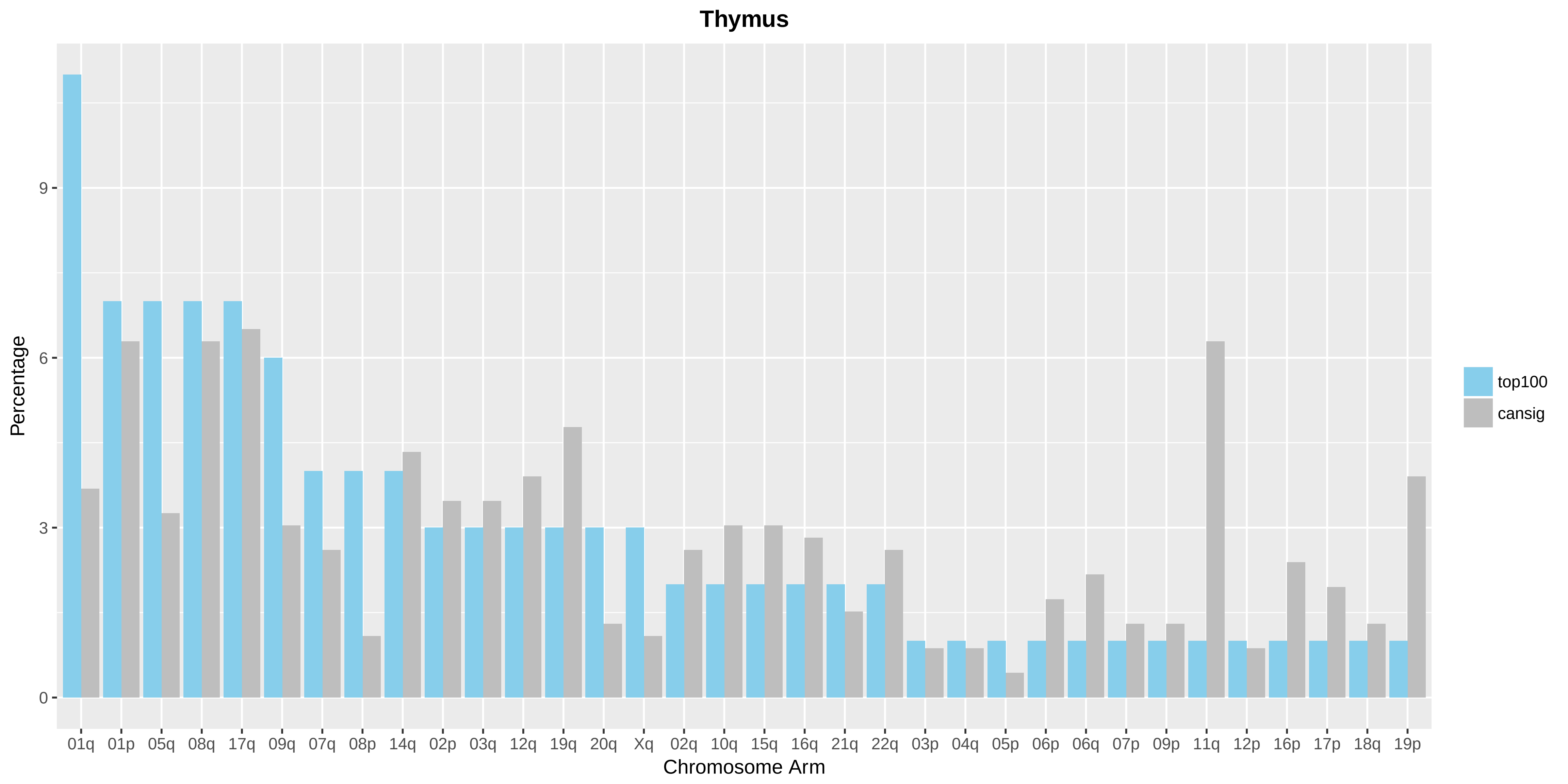

Thymus
Percentage
0
3
6
9
01q
01p
05q
08q
17q
09q
07q
08p
14q
02p
03q
12q
19q
20q
Xq
02q
10q
15q
16q
21q
22q
03p
04q
05p
06p
06q
07p
09p
11q
12p
16p
17p
18q
19p
Chromosome Arm
top100
cansig

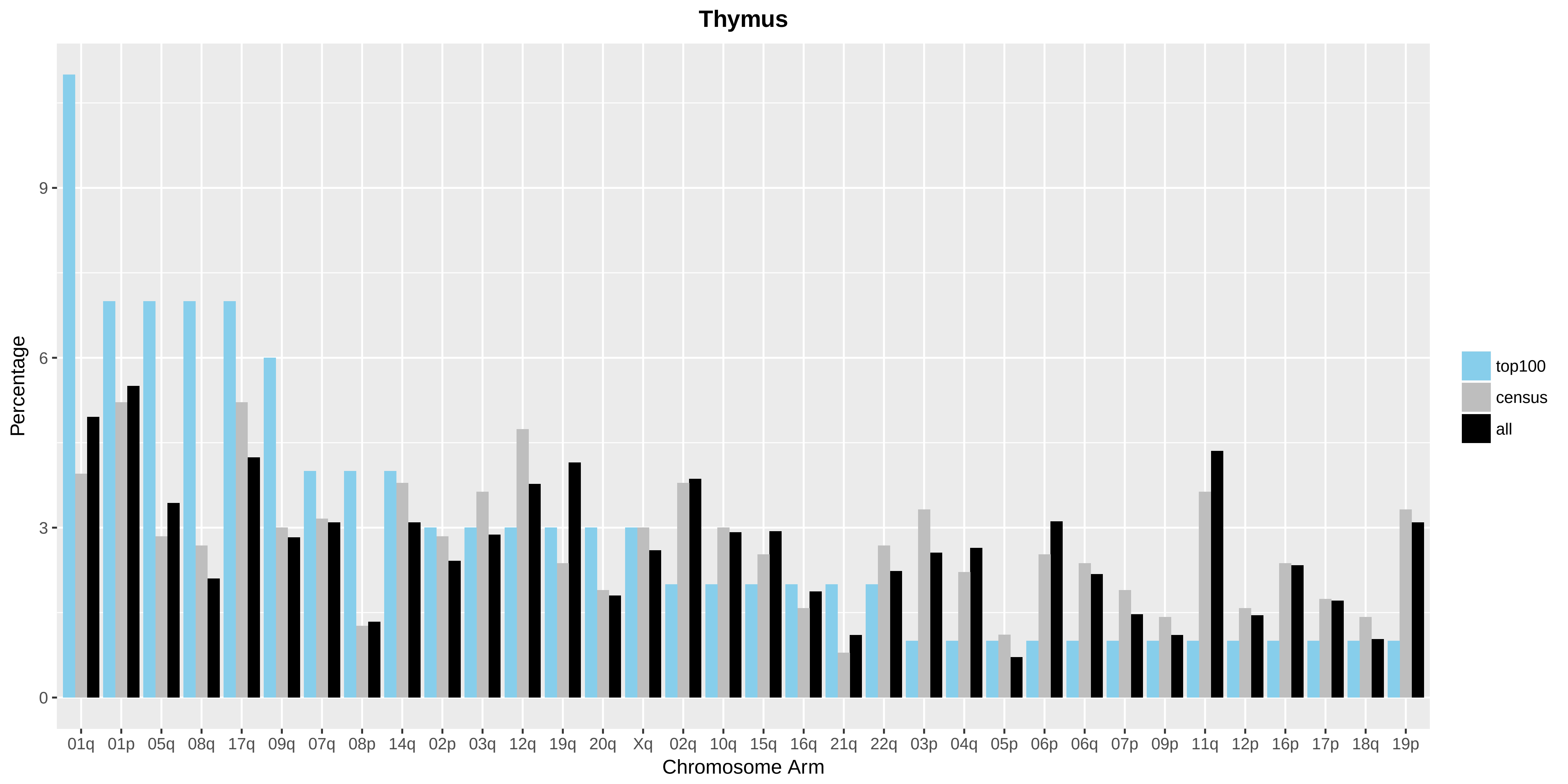

Thymus
Percentage
0
3
6
9
01q
01p
05q
08q
17q
09q
07q
08p
14q
02p
03q
12q
19q
20q
Xq
02q
10q
15q
16q
21q
22q
03p
04q
05p
06p
06q
07p
09p
11q
12p
16p
17p
18q
19p
Chromosome Arm
top100
census
all

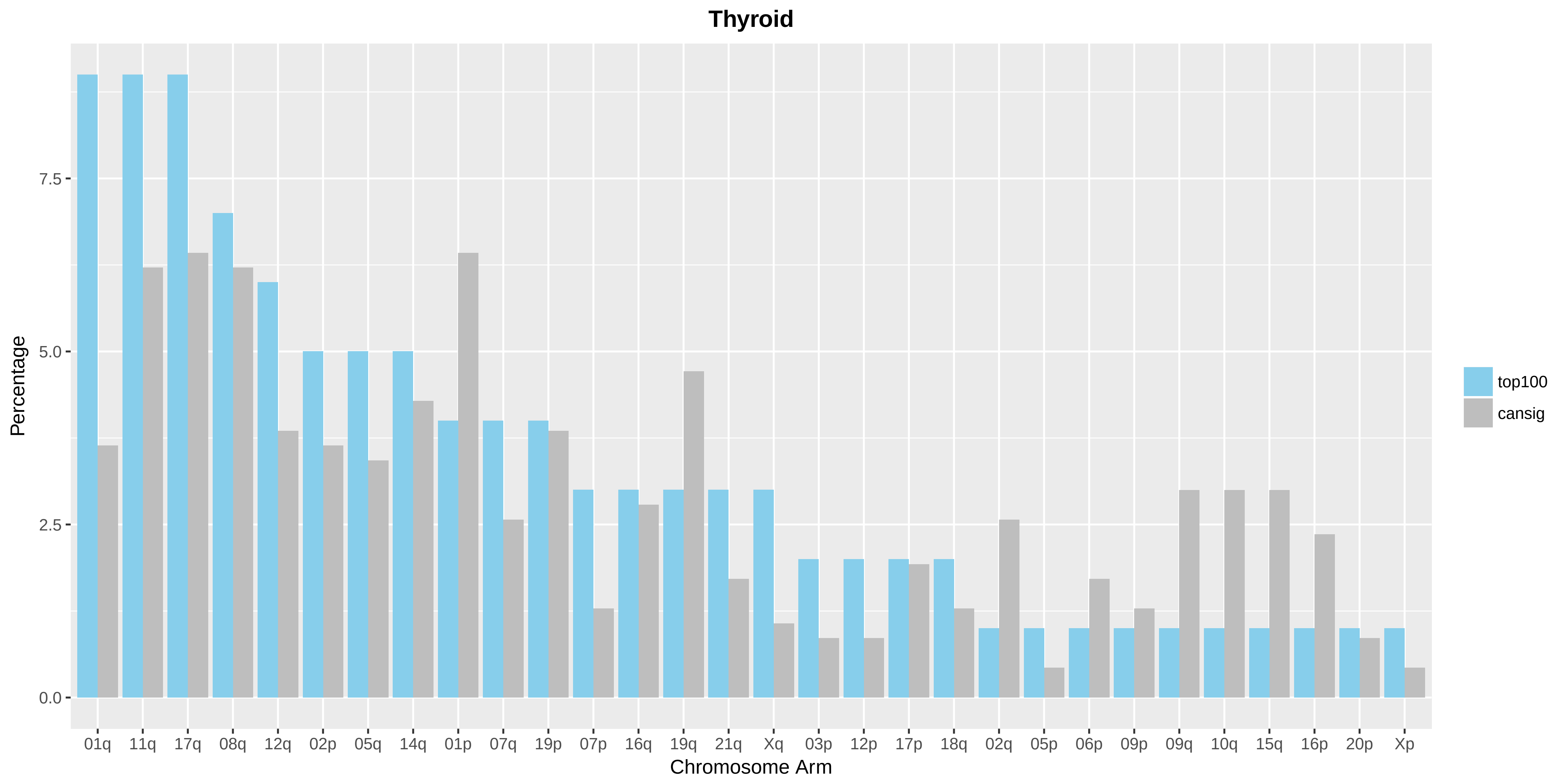
Thyroid
Percentage
0.0
2.5
5.0
7.5
01q
11q
17q
08q
12q
02p
05q
14q
01p
07q
19p
07p
16q
19q
21q
Xq
03p
12p
17p
18q
02q
05p
06p
09p
09q
10q
15q
16p
20p
Xp
Chromosome Arm
top100
cansig

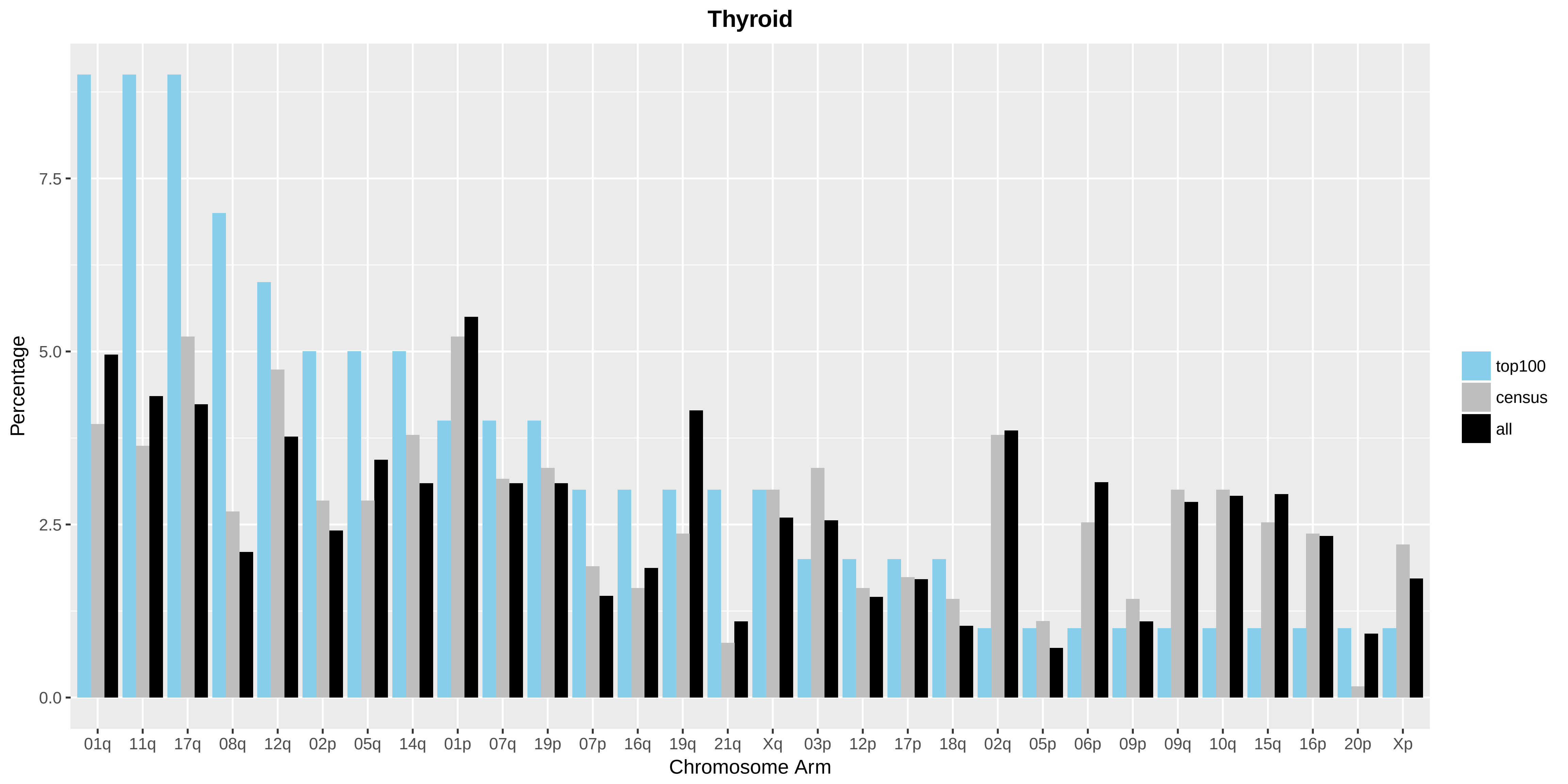

Thyroid
Percentage
7.5
5.0
2.5
0.0
01q 11q 17q 08q 12q 02p 05q 14q 01p 07q 19p 07p 16q 19q 21q Xq 03p 12p 17p 18q 02q 05p 06p 09p 09q 10q 15q 16p 20p Xp
Chromosome Arm
top100
census
all

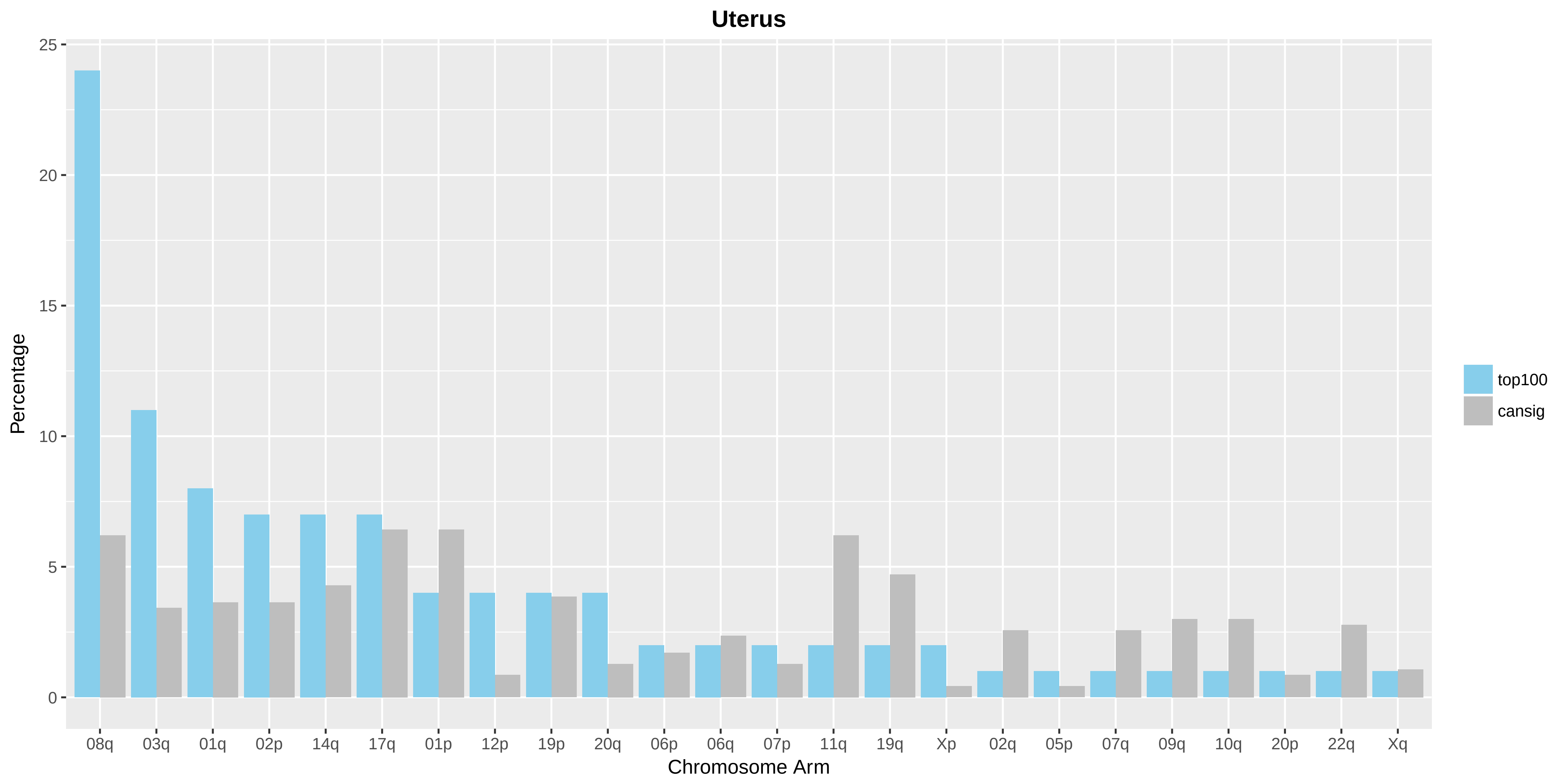

Uterus
Percentage
0
5
10
15
20
25
08q
03q
01q
02p
14q
17q
01p
12p
19p
20q
06p
06q
07p
11q
19q
Xp
02q
05p
07q
09q
10q
20p
22q
Xq
Chromosome Arm
top100
cansig

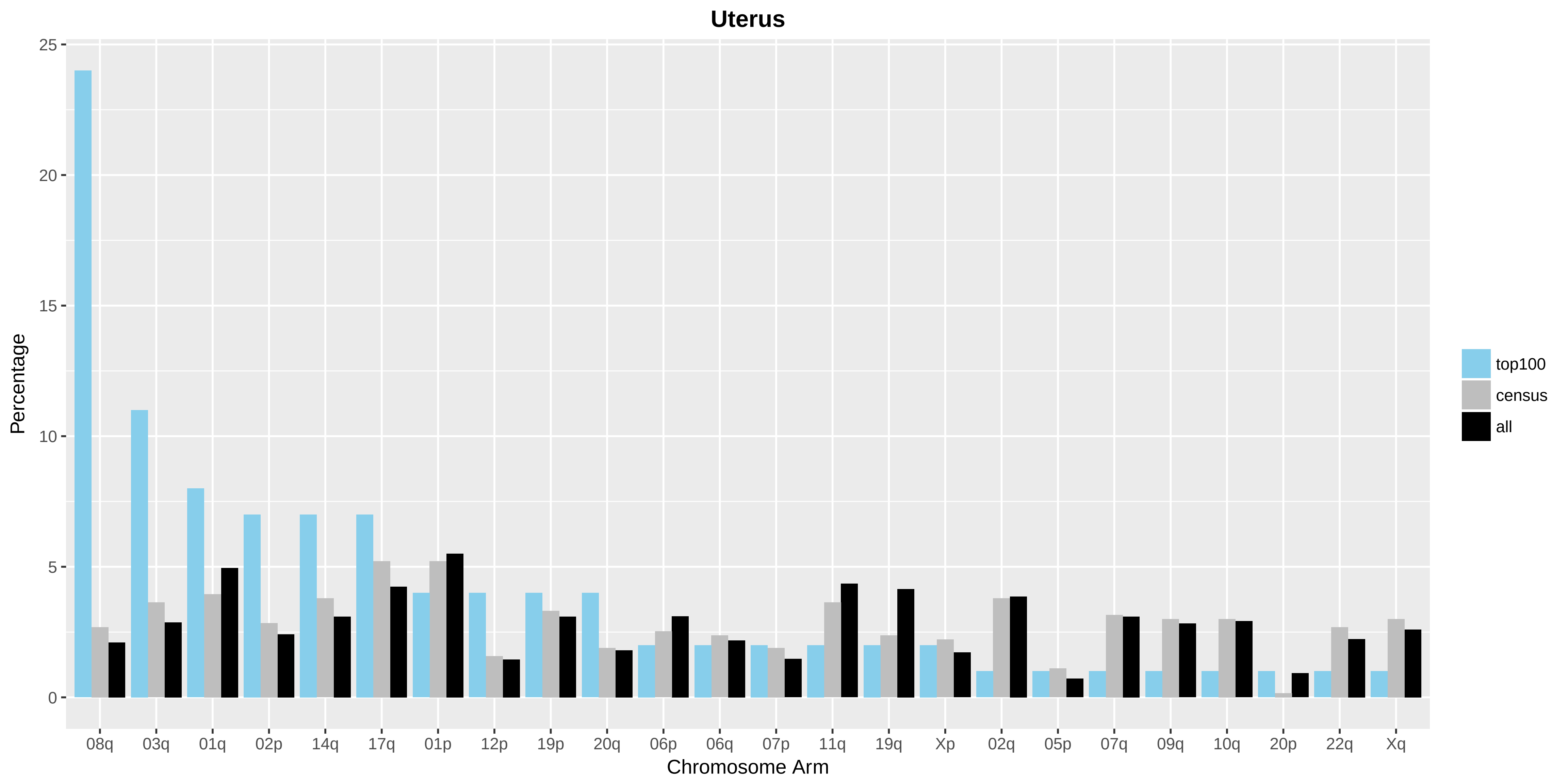
Uterus
Percentage
0
5
10
15
20
25
08q
03q
01q
02p
14q
17q
01p
12p
19p
20q
06p
06q
07p
11q
19q
Xp
02q
05p
07q
09q
10q
20p
22q
Xq
Chromosome Arm
top100
census
all
