# Supplementary material for: Overexpressed HSF1 cancer signature genes cluster in human chromosome 8q
Source: Hum Genomics. 2017 Dec 21;11:35. doi: 10.1186/s40246-017-0131-5 (PMC5740759; doi:10.1186/s40246-017-0131-5)
Supplement: Supplementary file 3 — Rank of HSF1 among primary sites. Syntenty visualization by Cinteny. An example showing the output web page of WebGestalt (PDF 738 kb) [file 40246_2017_131_MOESM3_ESM.pdf]

Additional File 3

Overexpressed HSF1 Cancer Signature Genes Cluster in Human Chromosome 8q

Christopher Q. Zhang[1, 4] (zhangcq@lafayette.edu), Heinric Williams[3,4] (hwilliams1@geisinger.edu), Thomas L. Prince[3,4,5] (tprince@geisinger.edu), Eric S. Ho[1, 2,5] (hoe@lafayette.edu)
[1]Department of Biology, [2]Department of Computer Science, Lafayette College, Easton, Pennsylvania, 18042, United States
[3]Urology Department, [4]Weis Research Center, Geisinger Medical Center, Danville, Pennsylvania, 17822, United States
[5]These authors contributed equally to this work
Corresponding author: Eric S. Ho
Email address: hoe@lafayette.edu

Figure S1. Average rank of HSF1-CanSig genes versus the rank of HSF1 in different primary tumor sites. HSF1 is the only HSF1-CanSig 8q gene in which its rank is always lower than the average.

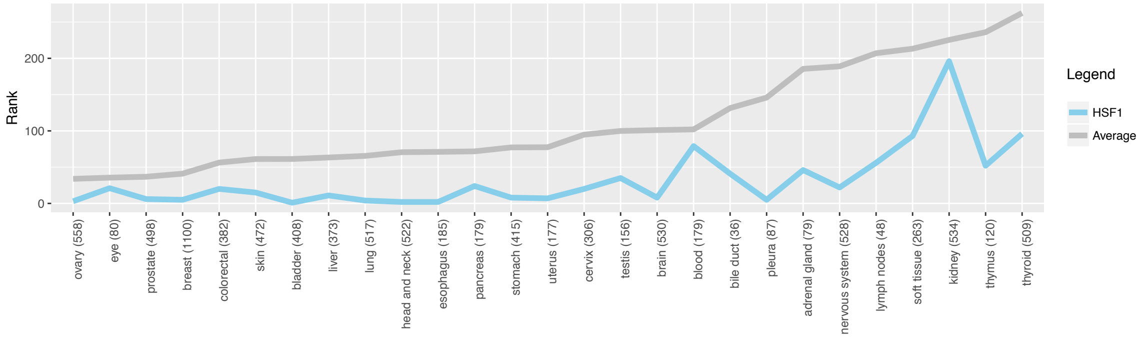

Figure S2. Syntenic Analysis between human, mouse, rat, and bovine by the whole chromosome. A. Human chromosome 8 versus mouse chromosome 15. B. Human chromosome 8 versus Rat chromosome 7. C. Human chromosome 8 versus Cow chromosome 14. D. Mouse chromosome 15 versus Rat chromosome 7. E. Mouse chromosome 15 versus cow chromosome 14. F. Rat chromosome 7 versus cow chromosome 14.

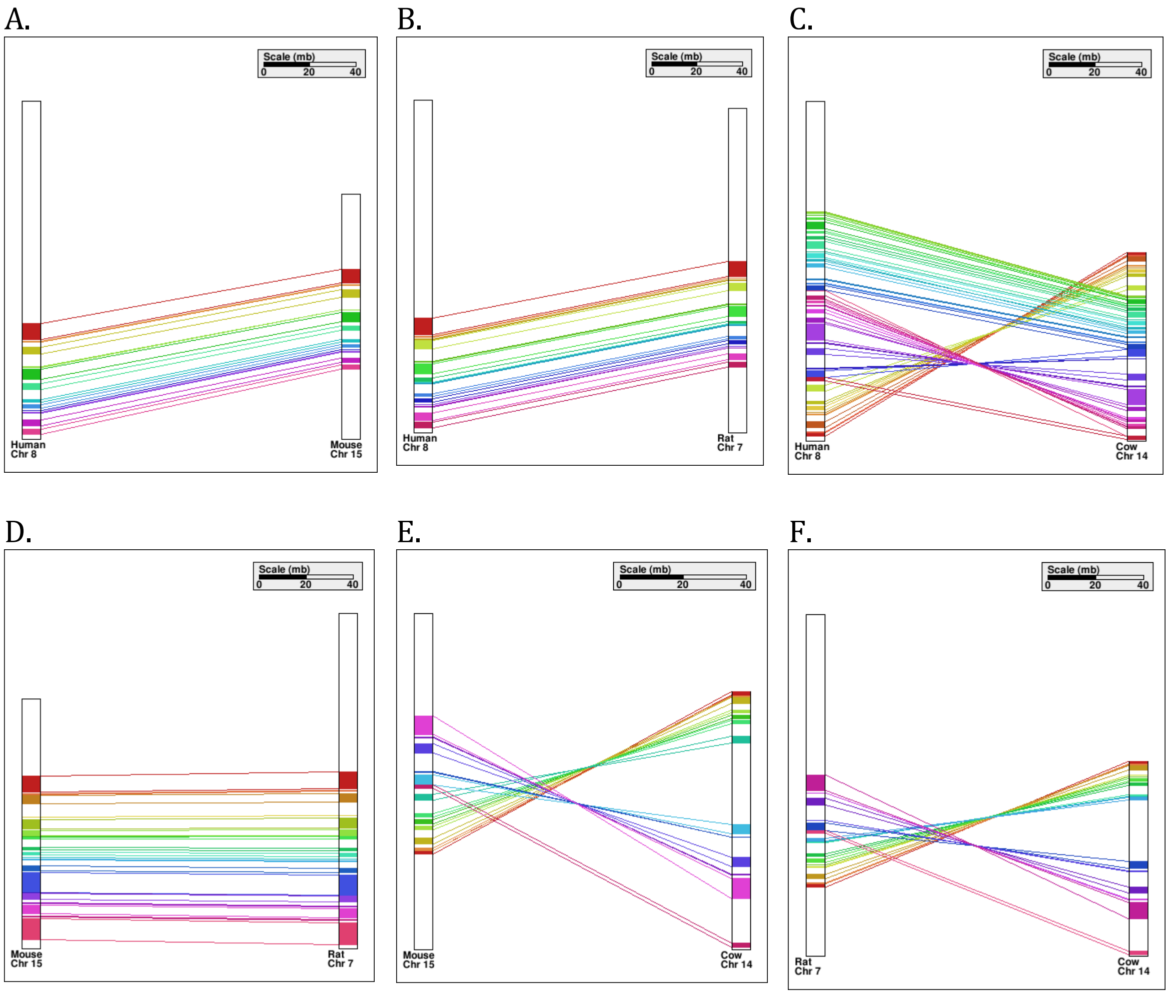

Figure S3. WebGestalt Analysis and hierarchical clustering analysis. A. The key information comes from “Summary of the analysis results” in the top left, and the “Enriched GO Terms” table.

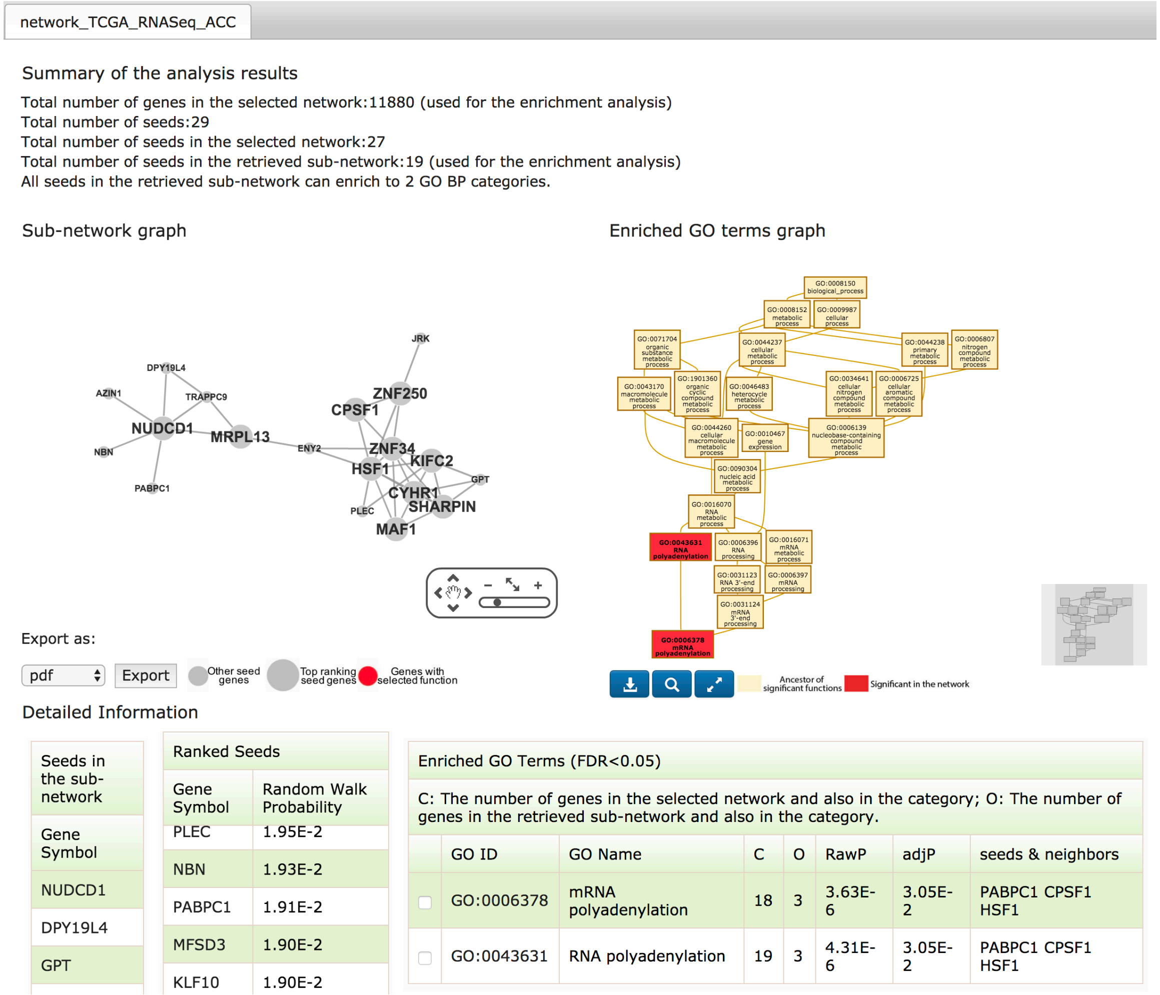

Summary of the analysis results

Total number of genes in the selected network:11880 (used for the enrichment analysis)
Total number of seeds:29
Total number of seeds in the selected network:27
Total number of seeds in the retrieved sub-network:19 (used for the enrichment analysis)
All seeds in the retrieved sub-network can enrich to 2 GO BP categories.

Detailed Information

Seeds in the sub-network
Gene Symbol
NUDCD1
DPY19L4
GPT

Ranked Seeds	
Gene Symbol	Random Walk Probability
PLEC	1.95E-2
NBN	1.93E-2
PABPC1	1.91E-2
MFSD3	1.90E-2
KLF10	1.90E-2

Enriched GO Terms (FDR<0.05)

C: The number of genes in the selected network and also in the category; O: The number of genes in the retrieved sub-network and also in the category.

GO ID	GO Name	C	O	RawP	adjP	seeds & neighbors
GO:0006378	mRNA polyadenylation	18	3	3.63E-6	3.05E-2	PABPC1 CPSF1 HSF1
GO:0043631	RNA polyadenylation	19	3	4.31E-6	3.05E-2	PABPC1 CPSF1 HSF1
