# Supplementary material for: Overexpressed HSF1 cancer signature genes cluster in human chromosome 8q
Source: Hum Genomics. 2017 Dec 21;11:35. doi: 10.1186/s40246-017-0131-5 (PMC5740759; doi:10.1186/s40246-017-0131-5)

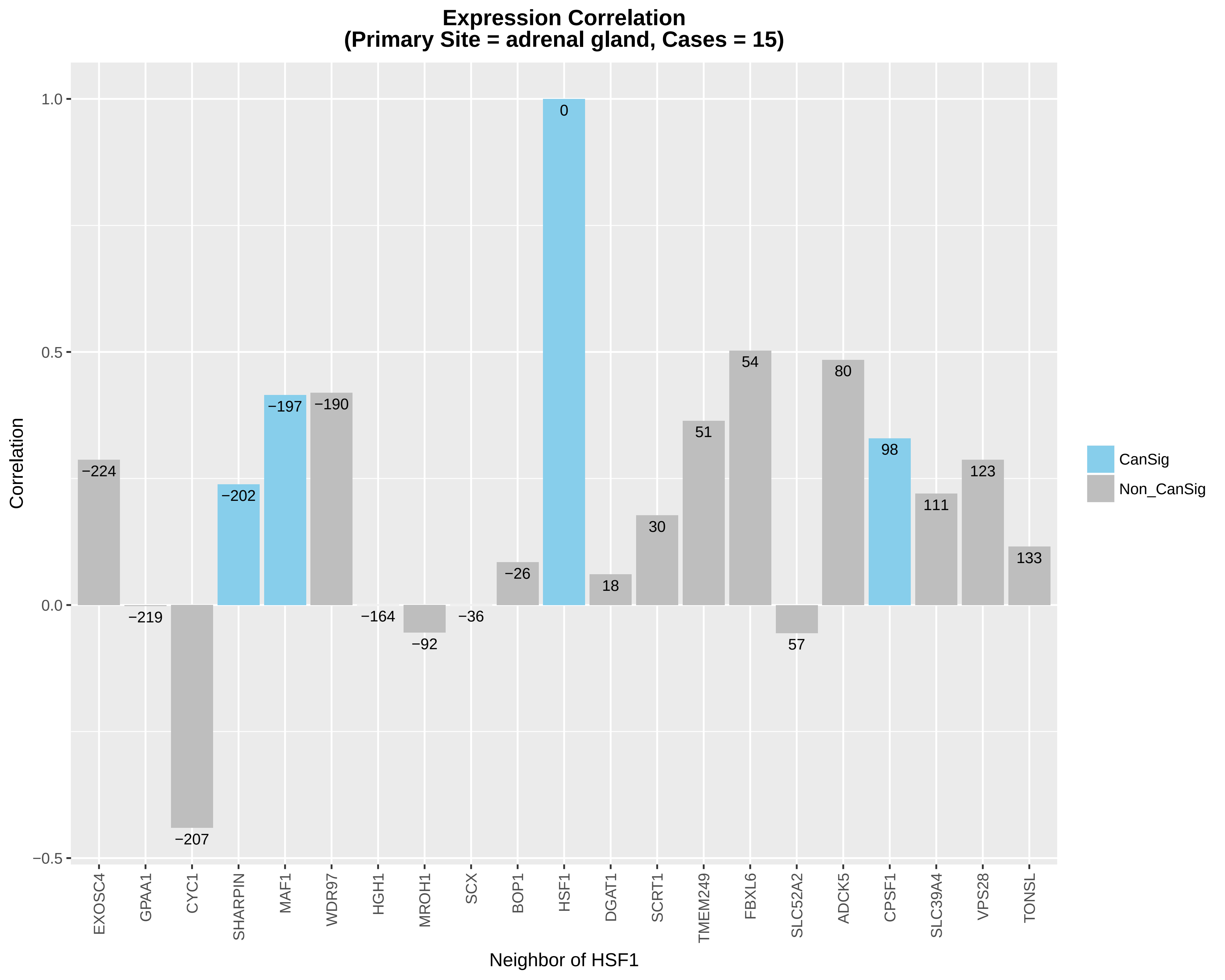

Expression Correlation
(Primary Site = adrenal gland, Cases = 15)
Correlation
1.0
0.5
0.0
−0.5
Neighbor of HSF1
EXOSC4
GPAA1
CYC1
SHARPIN
MAF1
WDR97
HGH1
MROH1
SCX
BOP1
HSF1
DGAT1
SCRT1
TMEM249
FBXL6
SLC52A2
ADCK5
CPSF1
SLC39A4
VPS28
TONSL
−224
−219
−207
−202
−197
−190
−164
−92
−36
−26
0
18
30
51
54
57
80
98
111
123
133
CanSig
Non_CanSig

Expression Correlation Matrix
(Primary Site = adrenal gland, Cases = 15)

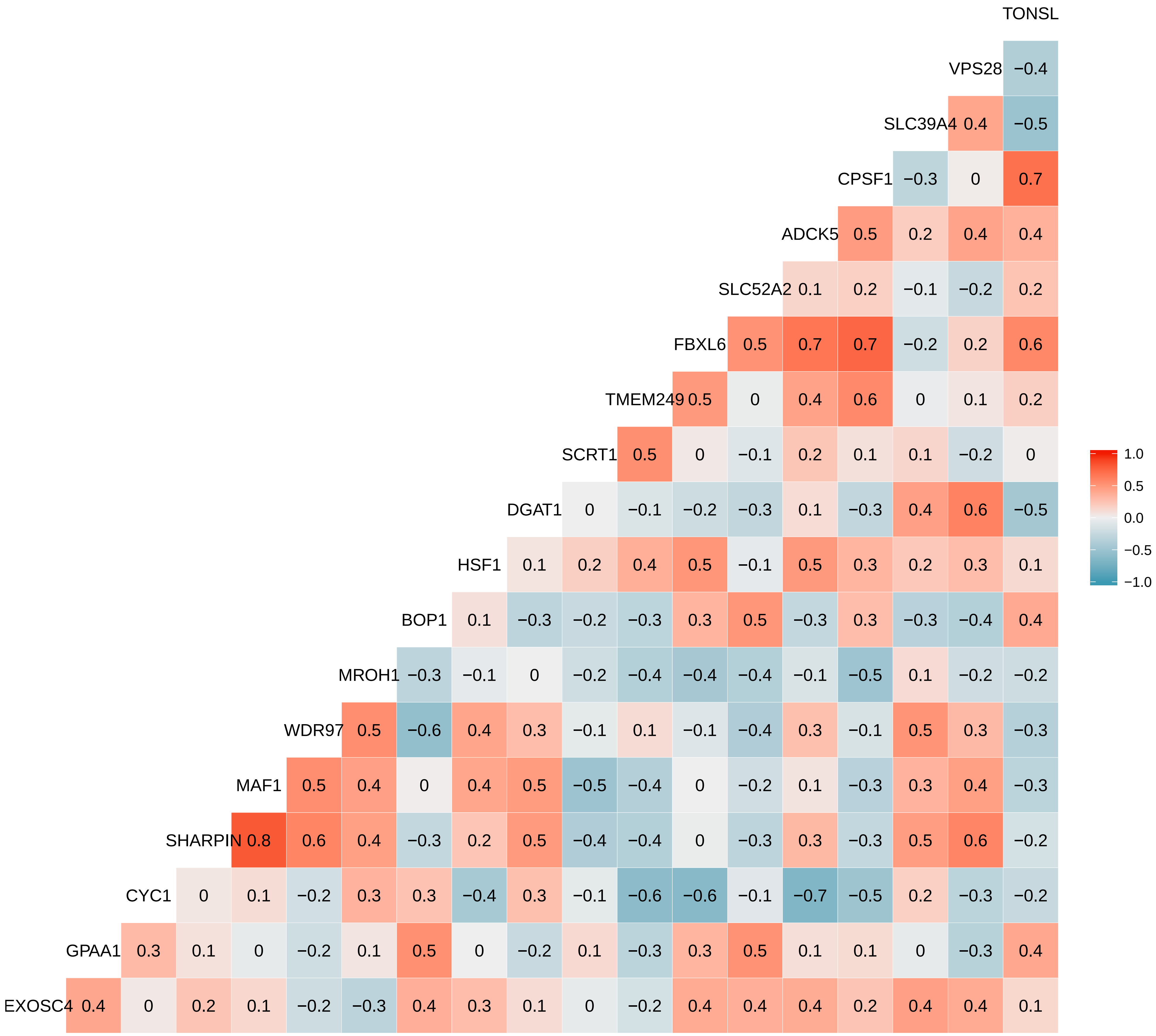

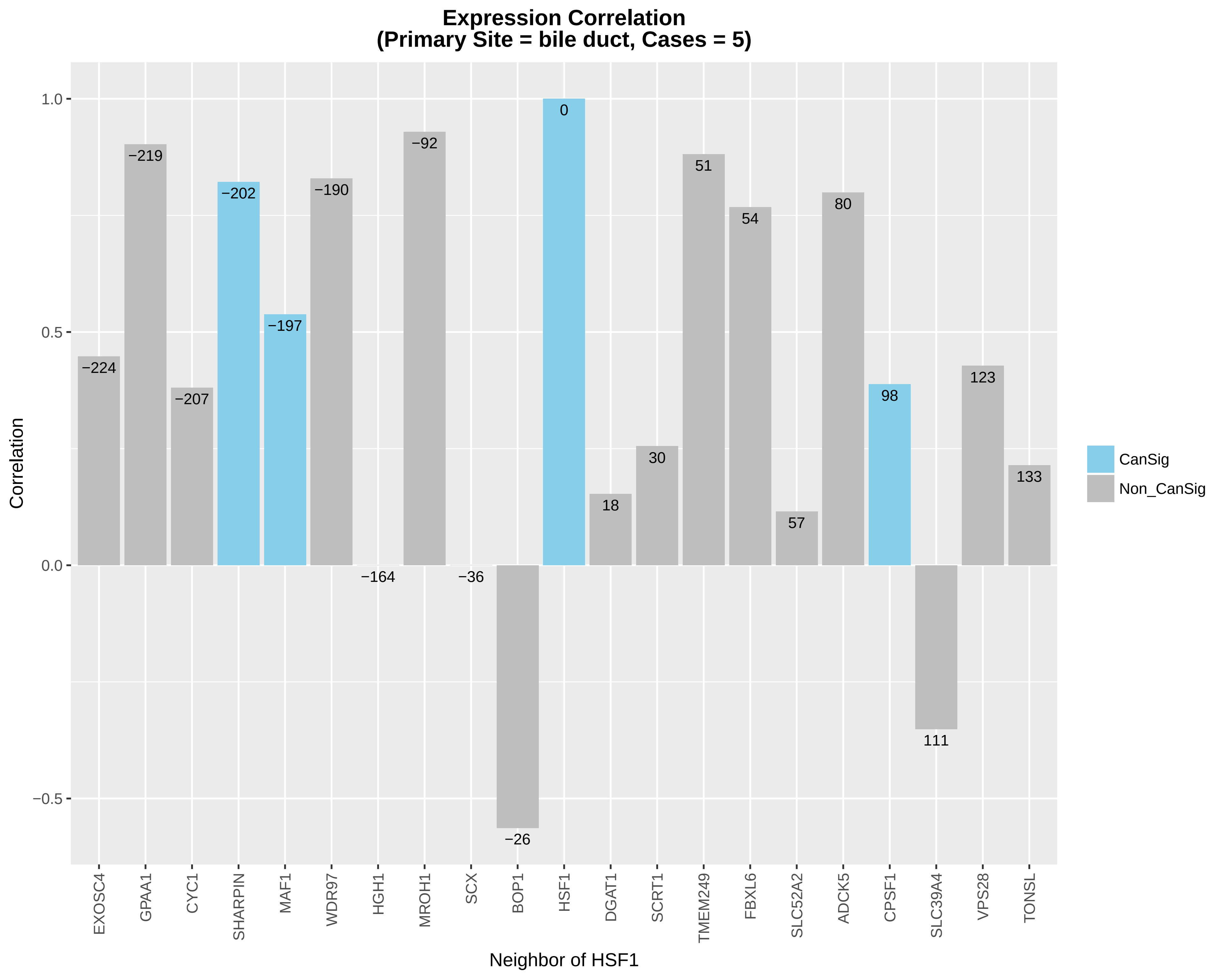

Expression Correlation
(Primary Site = bile duct, Cases = 5)
Correlation
1.0
0.5
0.0
−0.5
Neighbor of HSF1
EXOSC4
GPAA1
CYC1
SHARPIN
MAF1
WDR97
HGH1
MROH1
SCX
BOP1
HSF1
DGAT1
SCRT1
TMEM249
FBXL6
SLC52A2
ADCK5
CPSF1
SLC39A4
VPS28
TONSL
−224
−219
−207
−202
−197
−190
−164
−92
−36
−26
0
18
30
51
54
57
80
98
111
123
133
CanSig
Non_CanSig

Expression Correlation Matrix
(Primary Site = bile duct, Cases = 5)

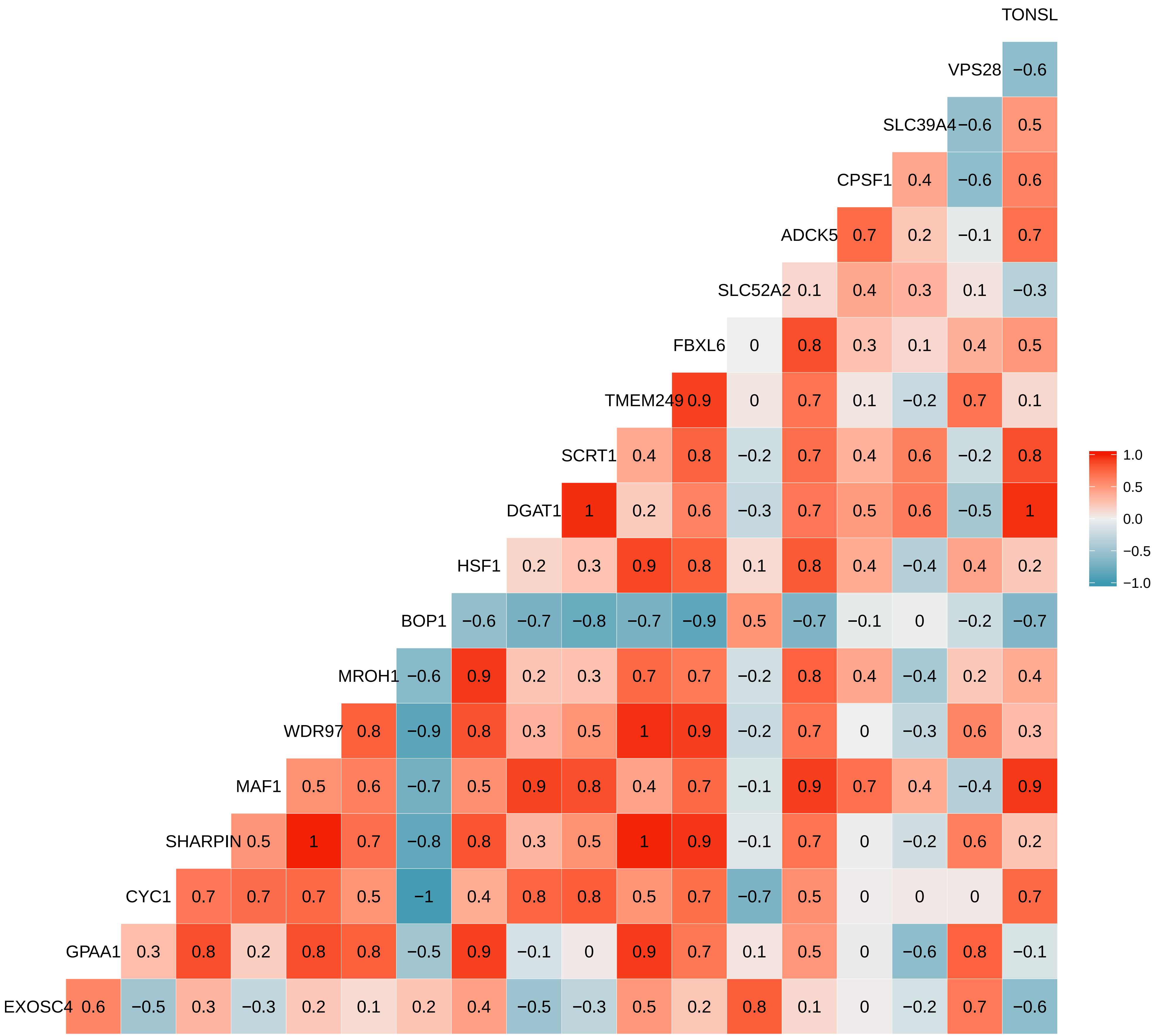

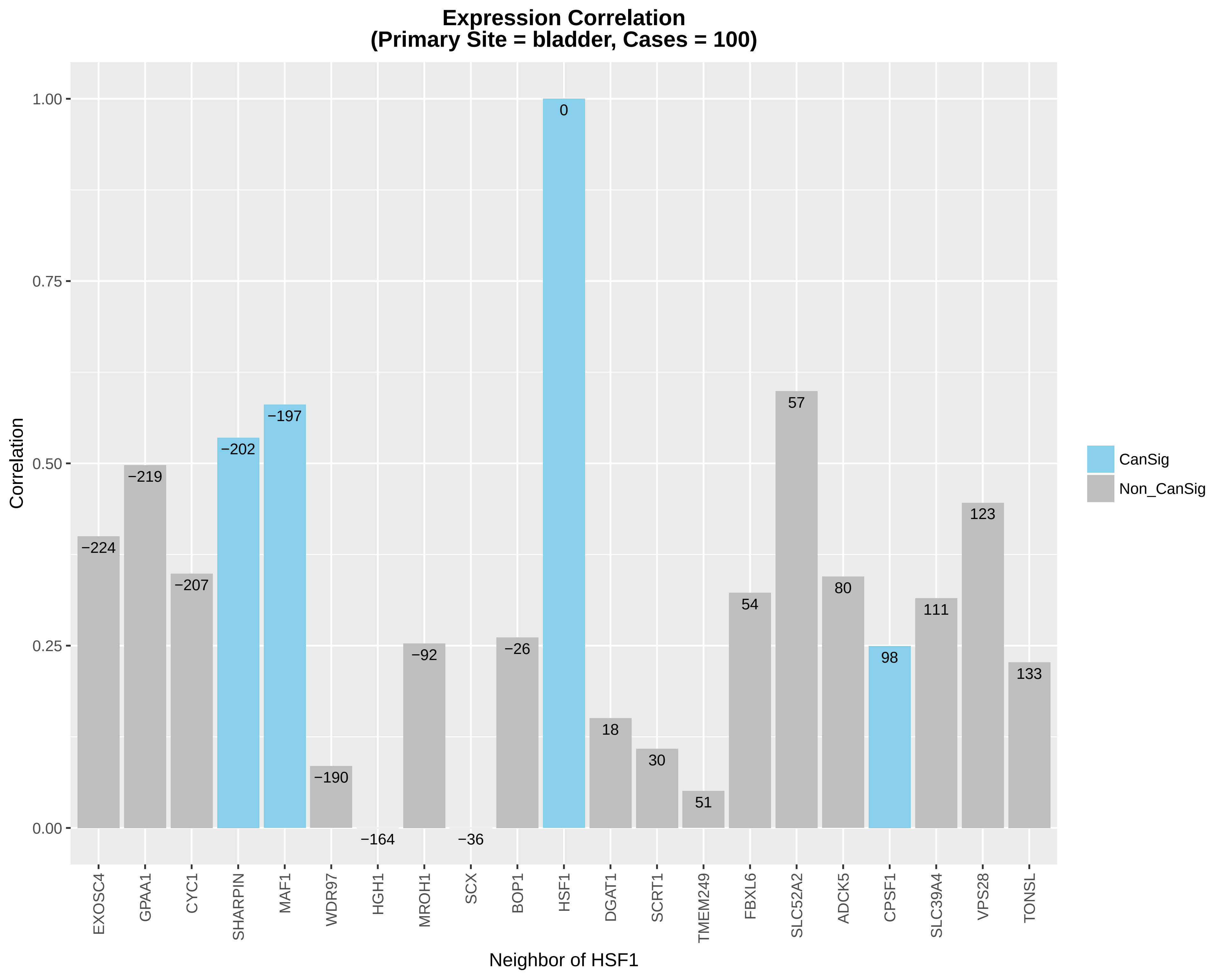

Expression Correlation
(Primary Site = bladder, Cases = 100)
Correlation
1.00
0.75
0.50
0.25
0.00
−224
−219
−207
−202
−197
−190
−164
−92
−36
−26
0
18
30
51
54
57
80
98
111
123
133
EXOSC4
GPAA1
CYC1
SHARPIN
MAF1
WDR97
HGH1
MROH1
SCX
BOP1
HSF1
DGAT1
SCRT1
TMEM249
FBXL6
SLC52A2
ADCK5
CPSF1
SLC39A4
VPS28
TONSL
Neighbor of HSF1
CanSig
Non_CanSig

Expression Correlation Matrix
(Primary Site = bladder, Cases = 100)

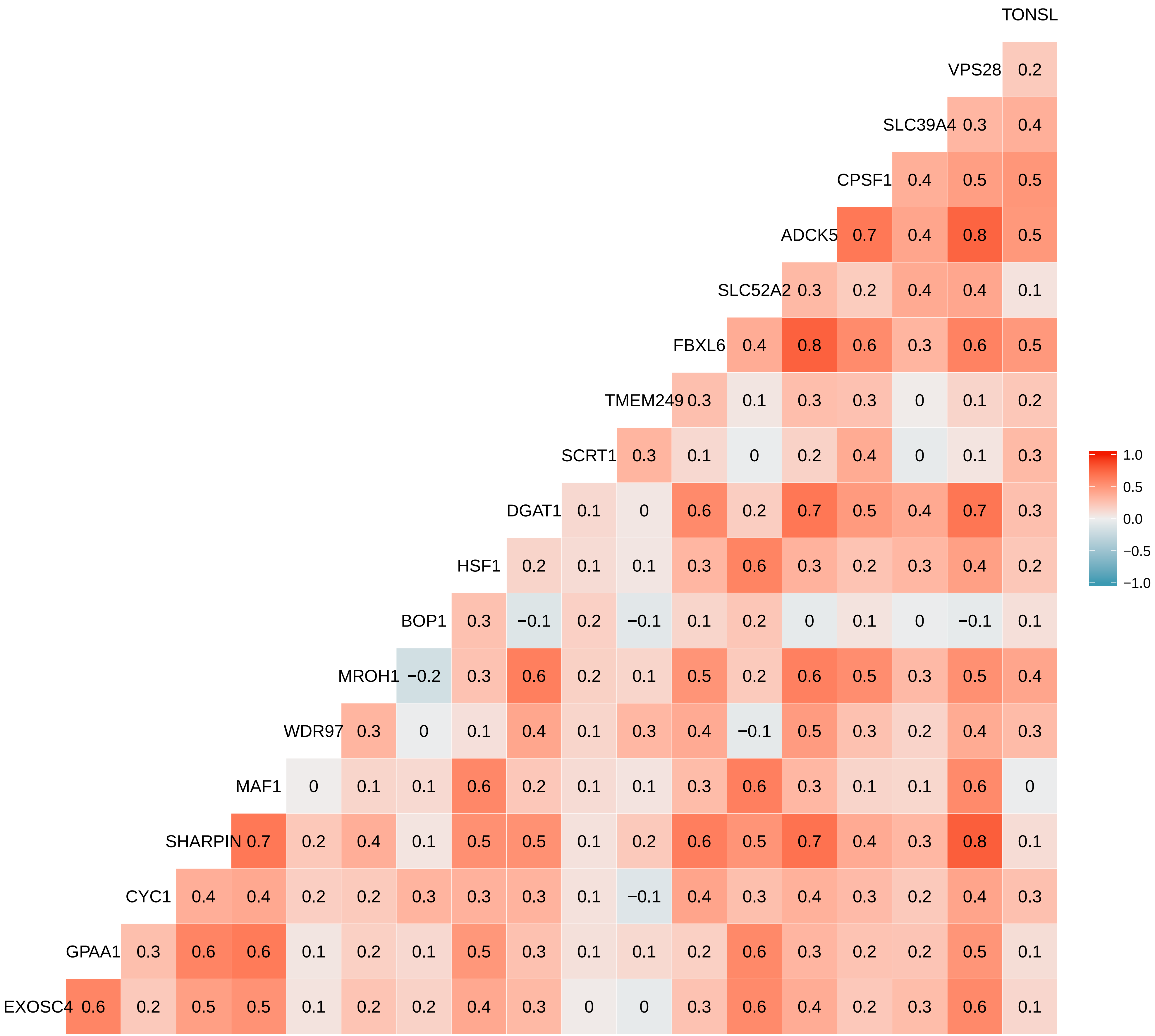

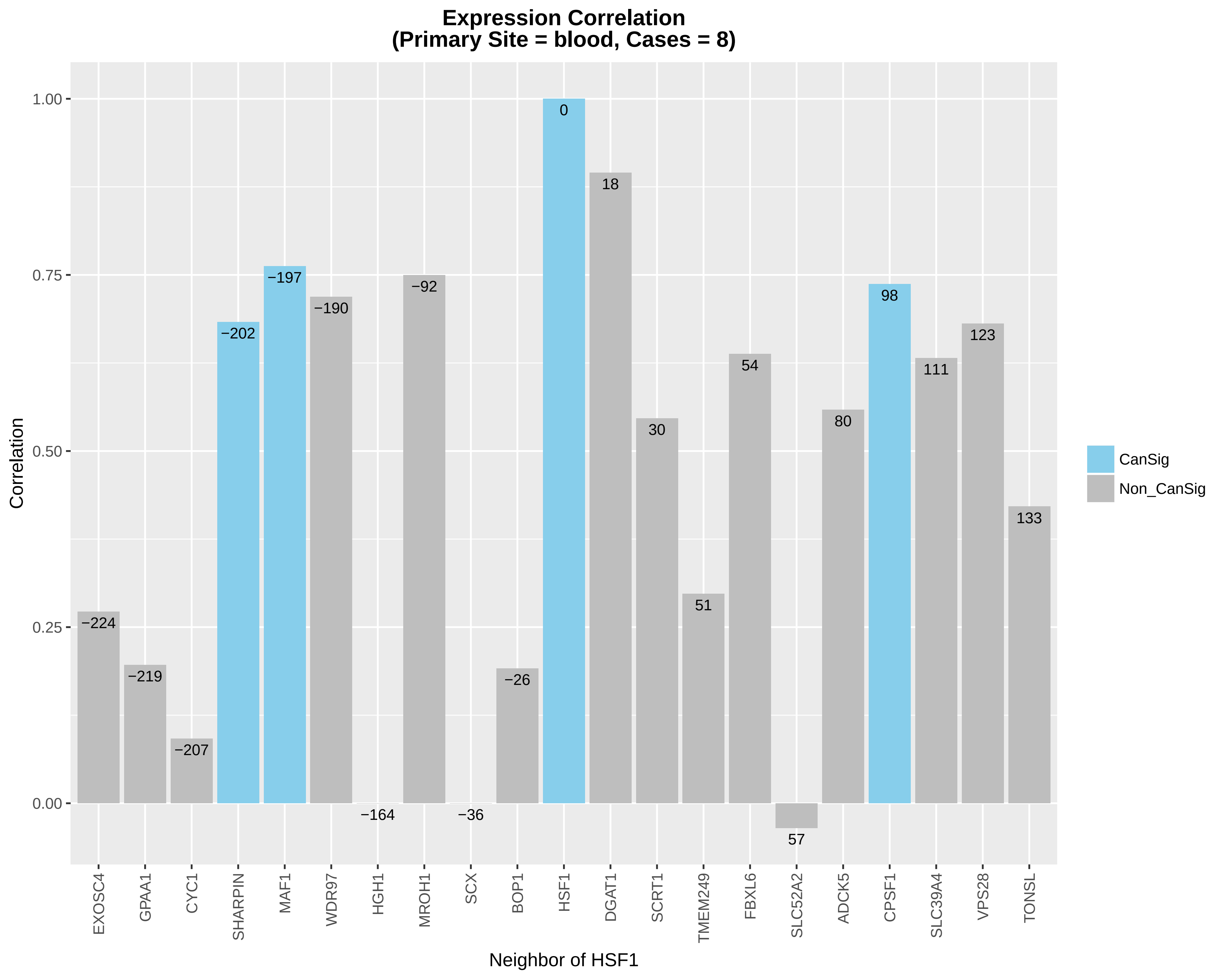

Expression Correlation
(Primary Site = blood, Cases = 8)
Correlation
1.00
0.75
0.50
0.25
0.00
−224
−219
−207
−202
−197
−190
−164
−92
−36
−26
0
18
30
51
54
57
80
98
111
123
133
EXOSC4
GPAA1
CYC1
SHARPIN
MAF1
WDR97
HGH1
MROH1
SCX
BOP1
HSF1
DGAT1
SCRT1
TMEM249
FBXL6
SLC52A2
ADCK5
CPSF1
SLC39A4
VPS28
TONSL
Neighbor of HSF1
CanSig
Non_CanSig

Expression Correlation Matrix
(Primary Site = blood, Cases = 8)

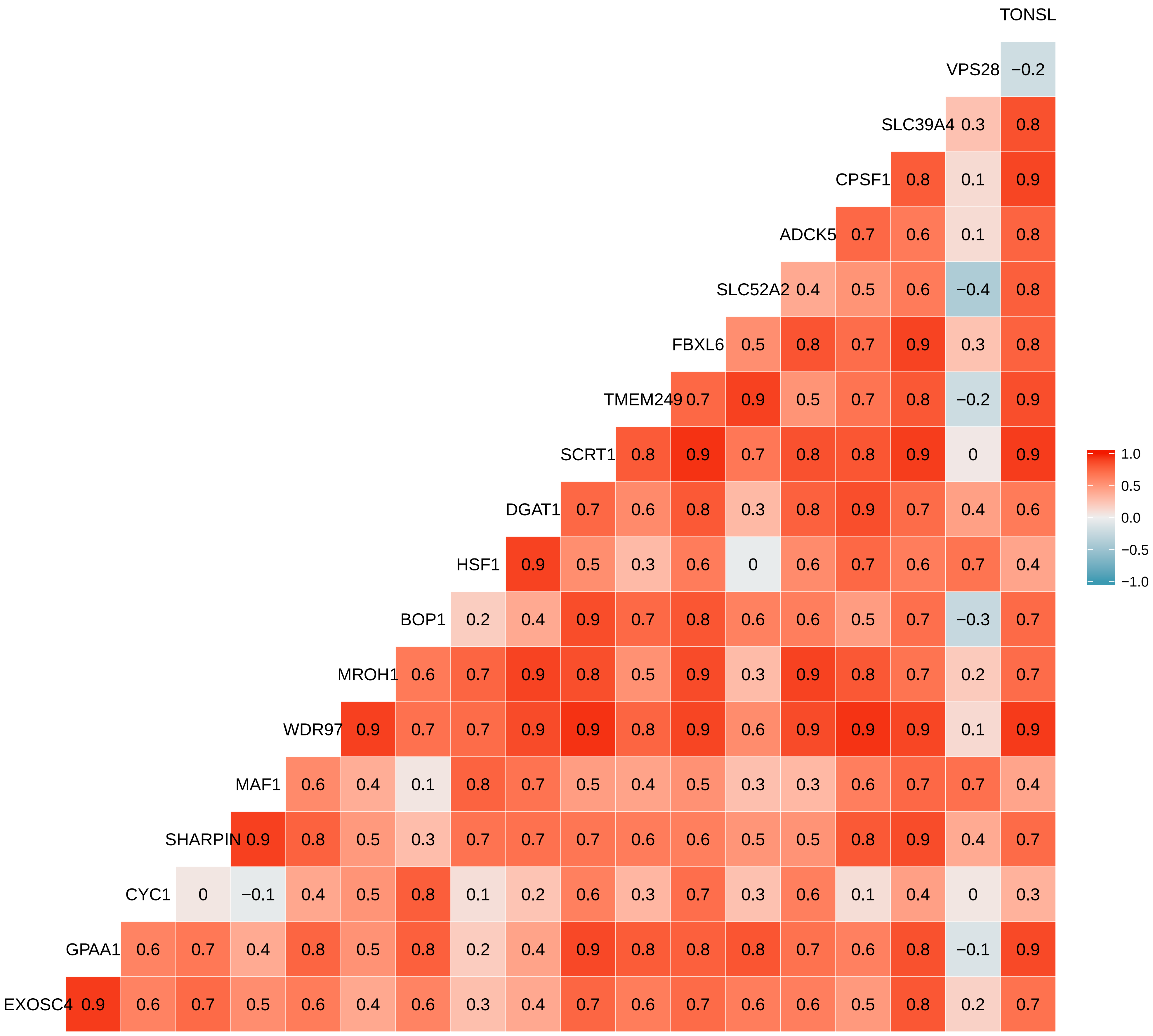

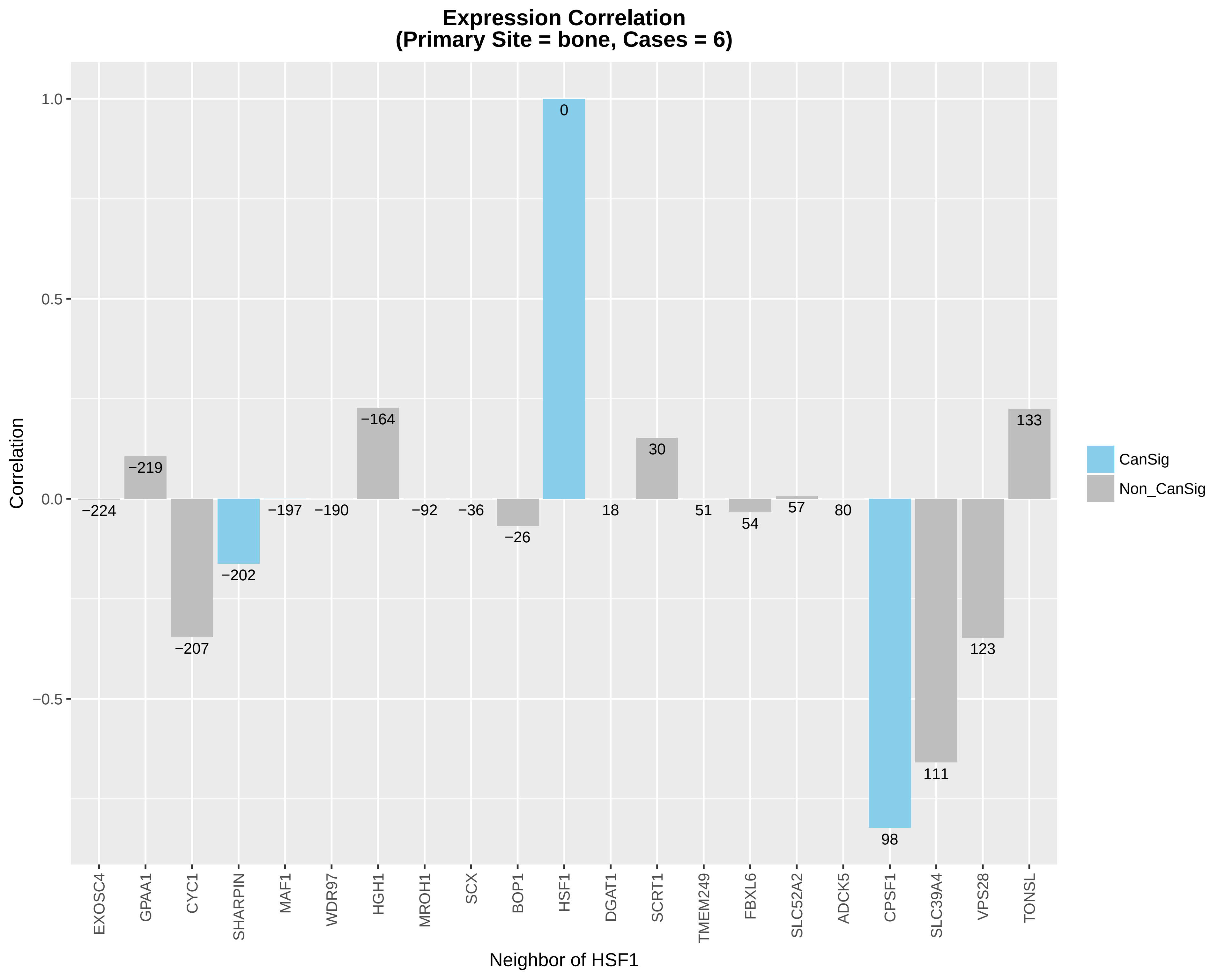
Expression Correlation
(Primary Site = bone, Cases = 6)
Correlation
1.0
0.5
0.0
−0.5
Neighbor of HSF1
EXOSC4
GPAA1
CYC1
SHARPIN
MAF1
WDR97
HGH1
MROH1
SCX
BOP1
HSF1
DGAT1
SCRT1
TMEM249
FBXL6
SLC52A2
ADCK5
CPSF1
SLC39A4
VPS28
TONSL
−224
−219
−207
−202
−197
−190
−164
−92
−36
−26
0
18
30
51
54
57
80
98
111
123
133
CanSig
Non_CanSig

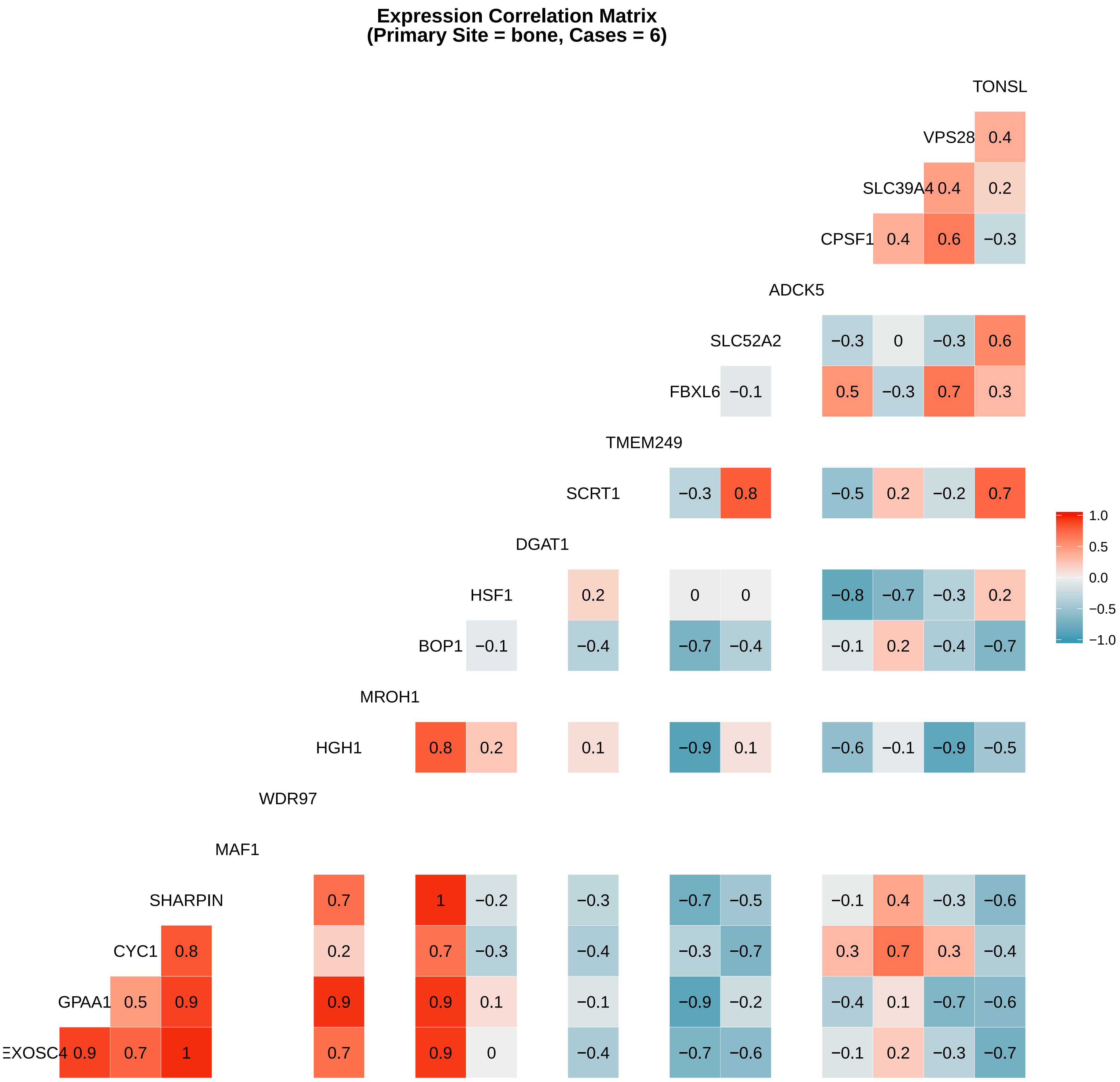
Expression Correlation Matrix
(Primary Site = bone, Cases = 6)
TONSL
VPS28
SLC39A4
CPSF1
ADCK5
SLC52A2
FBXL6
TMEM249
SCRT1
DGAT1
HSF1
BOP1
MROH1
HGH1
WDR97
MAF1
SHARPIN
CYC1
GPAA1
EXOSC4
1.0
0.5
0.0
−0.5
−1.0

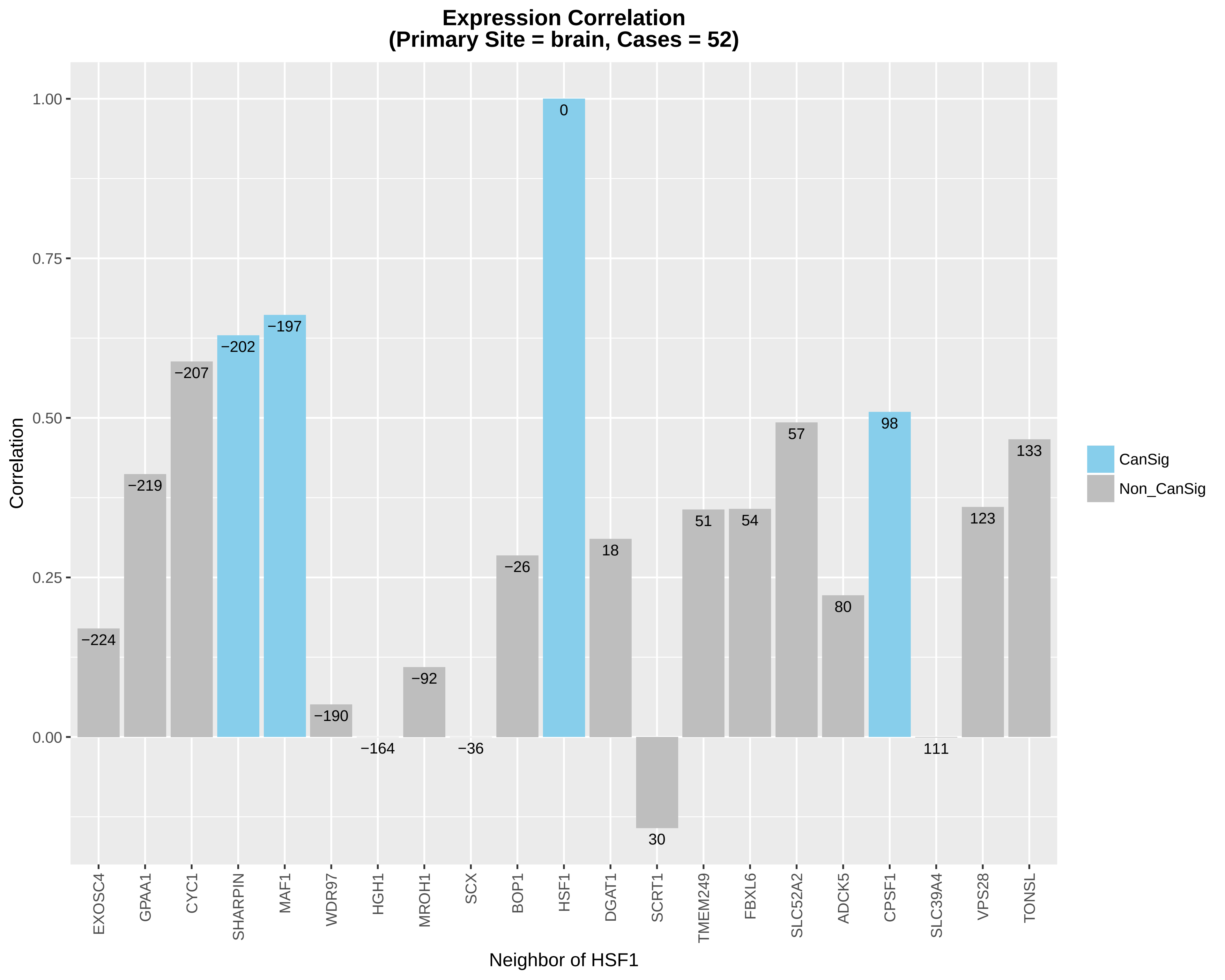

Expression Correlation
(Primary Site = brain, Cases = 52)
Correlation
1.00
0.75
0.50
0.25
0.00
0
−197
−202
−207
−219
−224
−190
−164
−92
−36
−26
18
30
51
54
57
80
98
111
123
133
EXOSC4
GPAA1
CYC1
SHARPIN
MAF1
WDR97
HGH1
MROH1
SCX
BOP1
HSF1
DGAT1
SCRT1
TMEM249
FBXL6
SLC52A2
ADCK5
CPSF1
SLC39A4
VPS28
TONSL
Neighbor of HSF1
CanSig
Non_CanSig

Expression Correlation Matrix
(Primary Site = brain, Cases = 52)

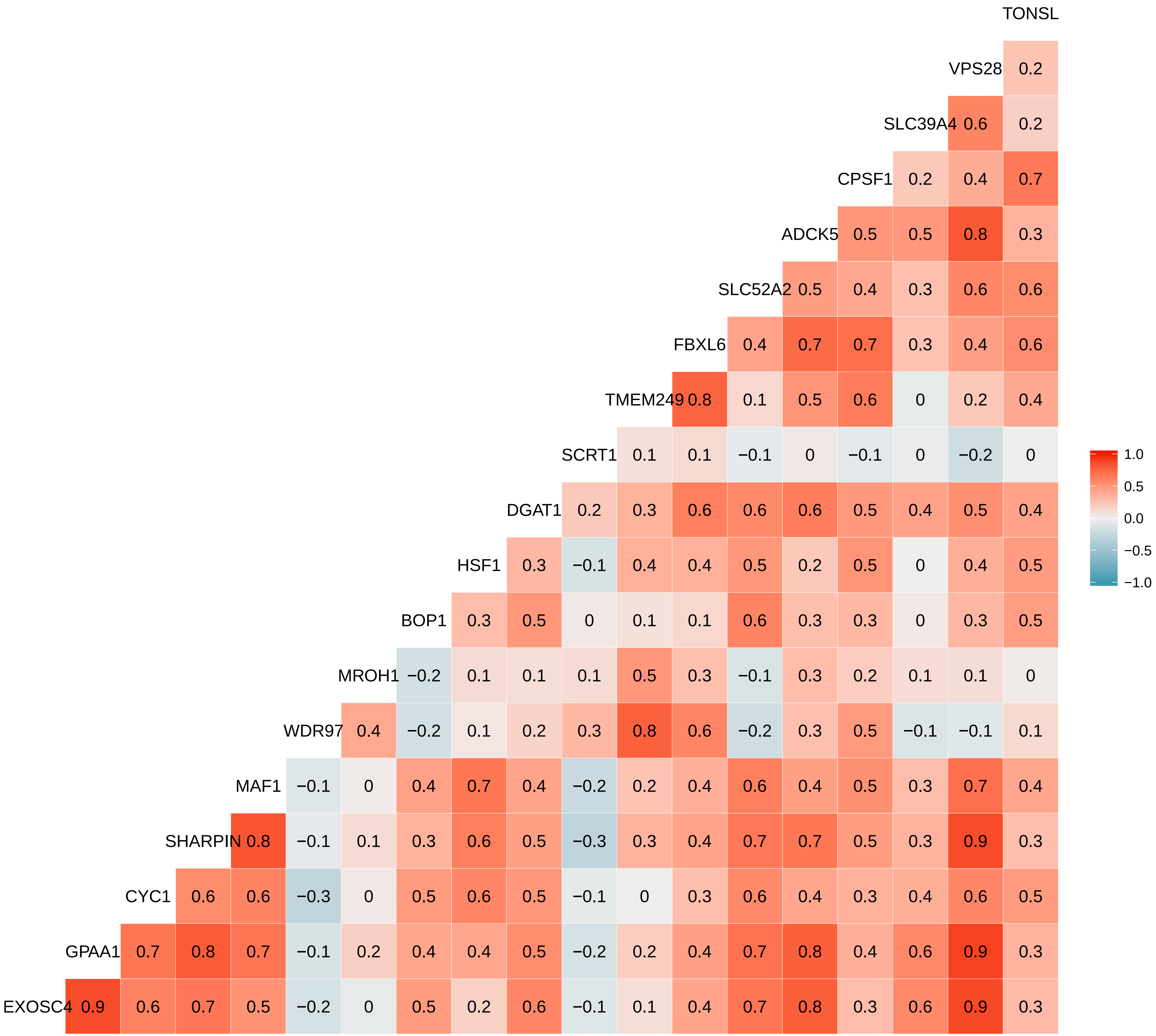

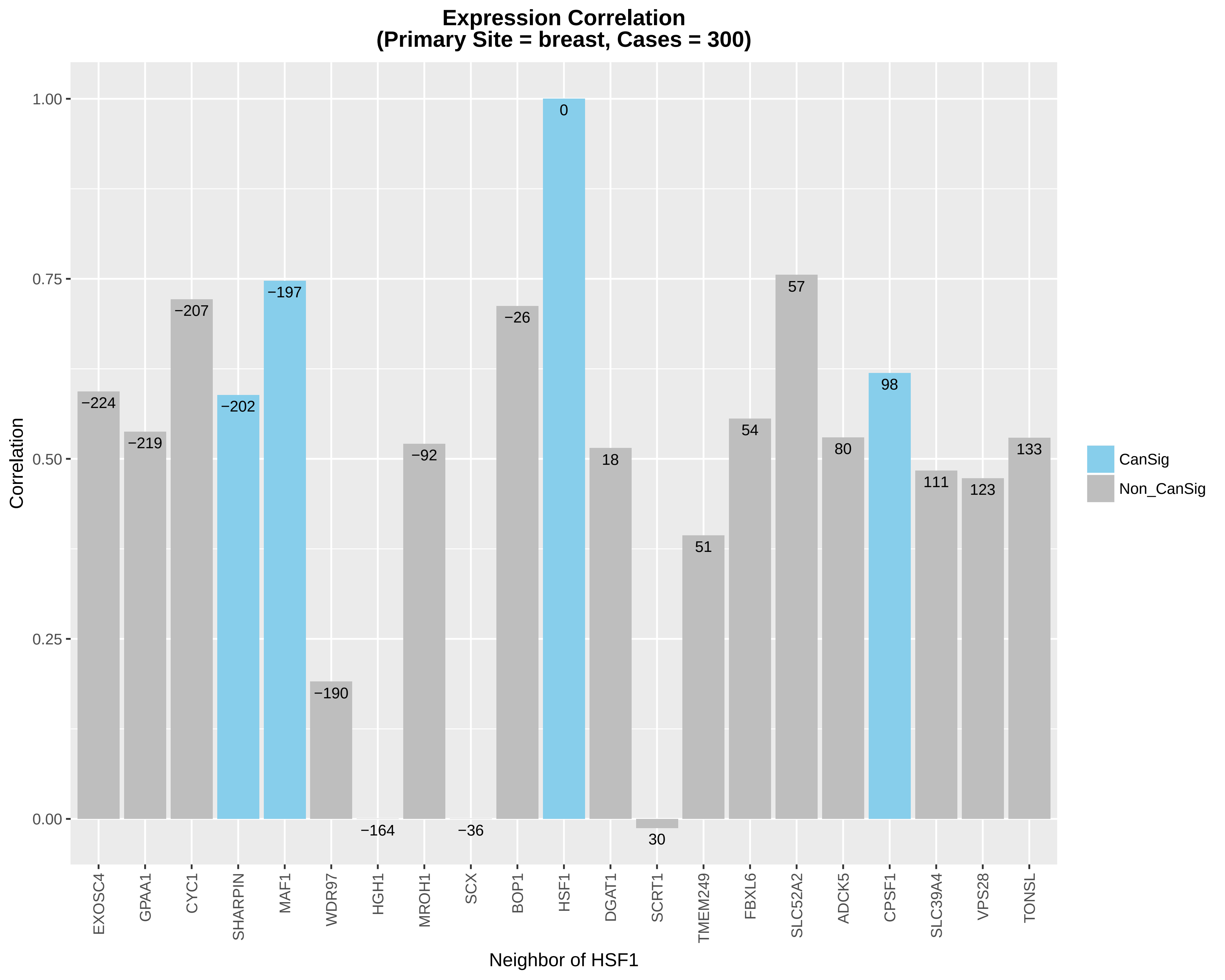

Expression Correlation
(Primary Site = breast, Cases = 300)
Correlation
1.00
0.75
0.50
0.25
0.00
−224
−219
−207
−202
−197
−190
−164
−92
−36
−26
0
18
30
51
54
57
80
98
111
123
133
EXOSC4
GPAA1
CYC1
SHARPIN
MAF1
WDR97
HGH1
MROH1
SCX
BOP1
HSF1
DGAT1
SCRT1
TMEM249
FBXL6
SLC52A2
ADCK5
CPSF1
SLC39A4
VPS28
TONSL
Neighbor of HSF1
CanSig
Non_CanSig

Expression Correlation Matrix
(Primary Site = breast, Cases = 300)

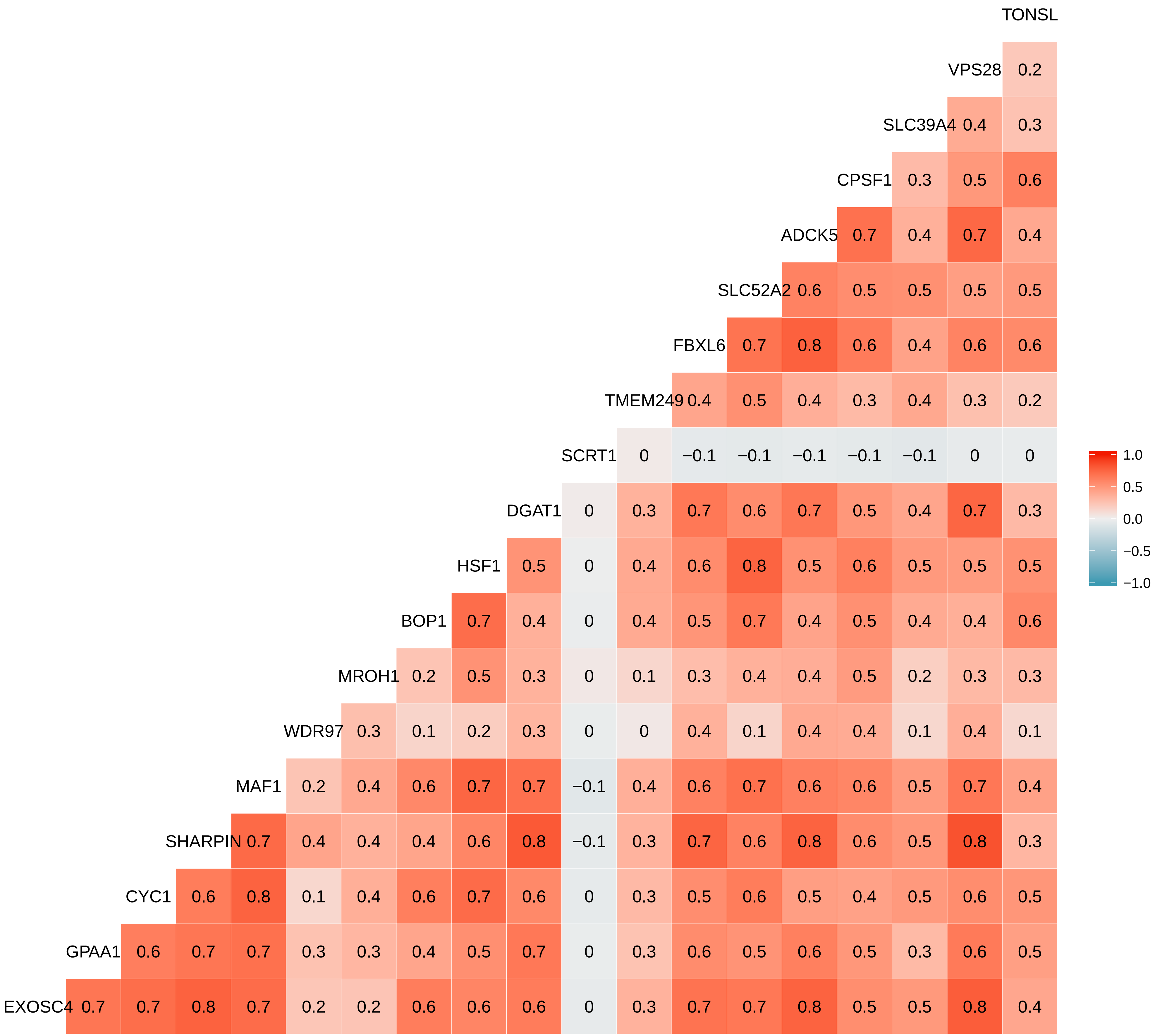

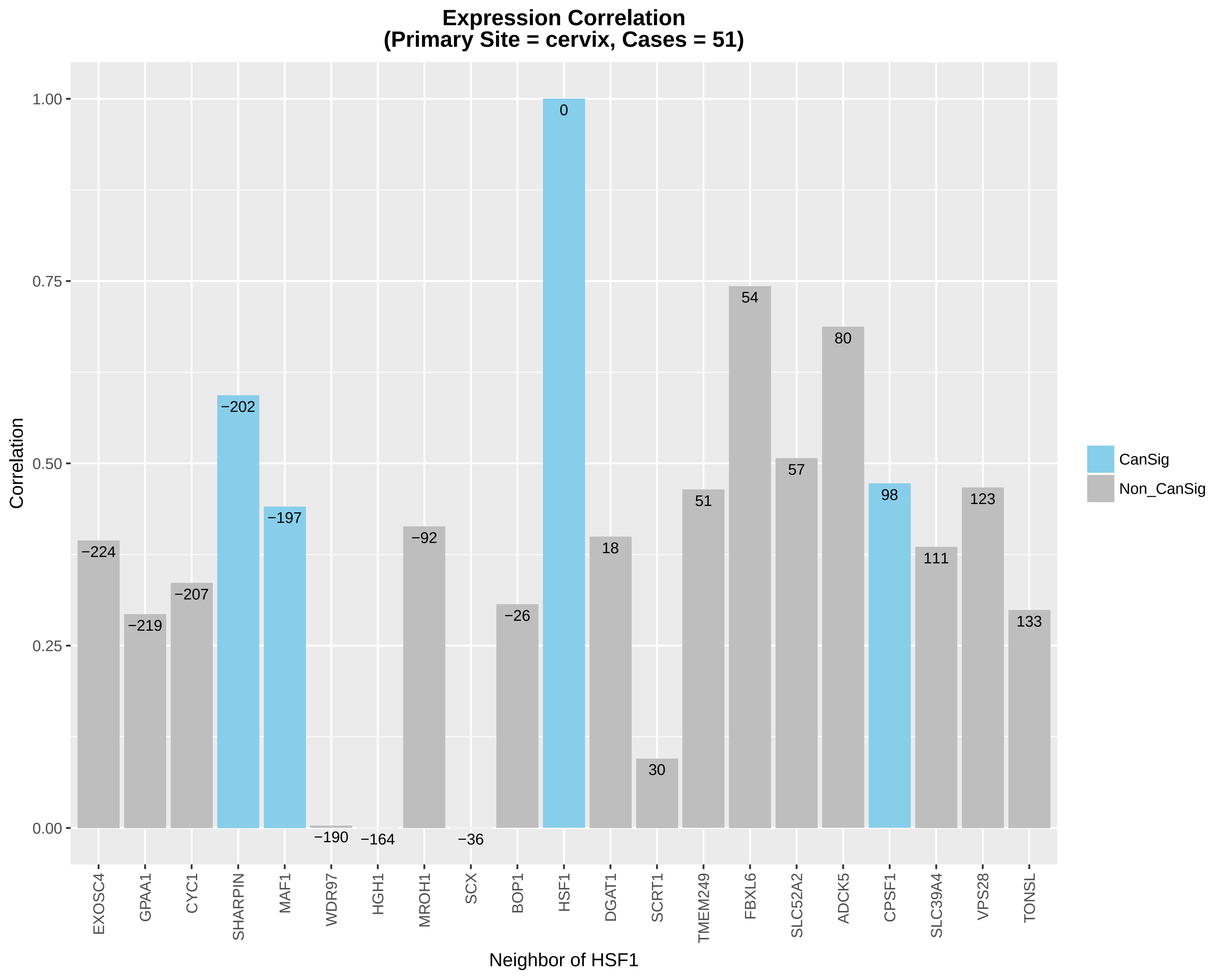
Expression Correlation
(Primary Site = cervix, Cases = 51)
Correlation
1.00
0.75
0.50
0.25
0.00
−224
−219
−207
−202
−197
−190
−164
−92
−36
−26
0
18
30
51
54
57
80
98
111
123
133
EXOSC4
GPAA1
CYC1
SHARPIN
MAF1
WDR97
HGH1
MROH1
SCX
BOP1
HSF1
DGAT1
SCRT1
TMEM249
FBXL6
SLC52A2
ADCK5
CPSF1
SLC39A4
VPS28
TONSL
Neighbor of HSF1
CanSig
Non_CanSig

Expression Correlation Matrix
(Primary Site = cervix, Cases = 51)

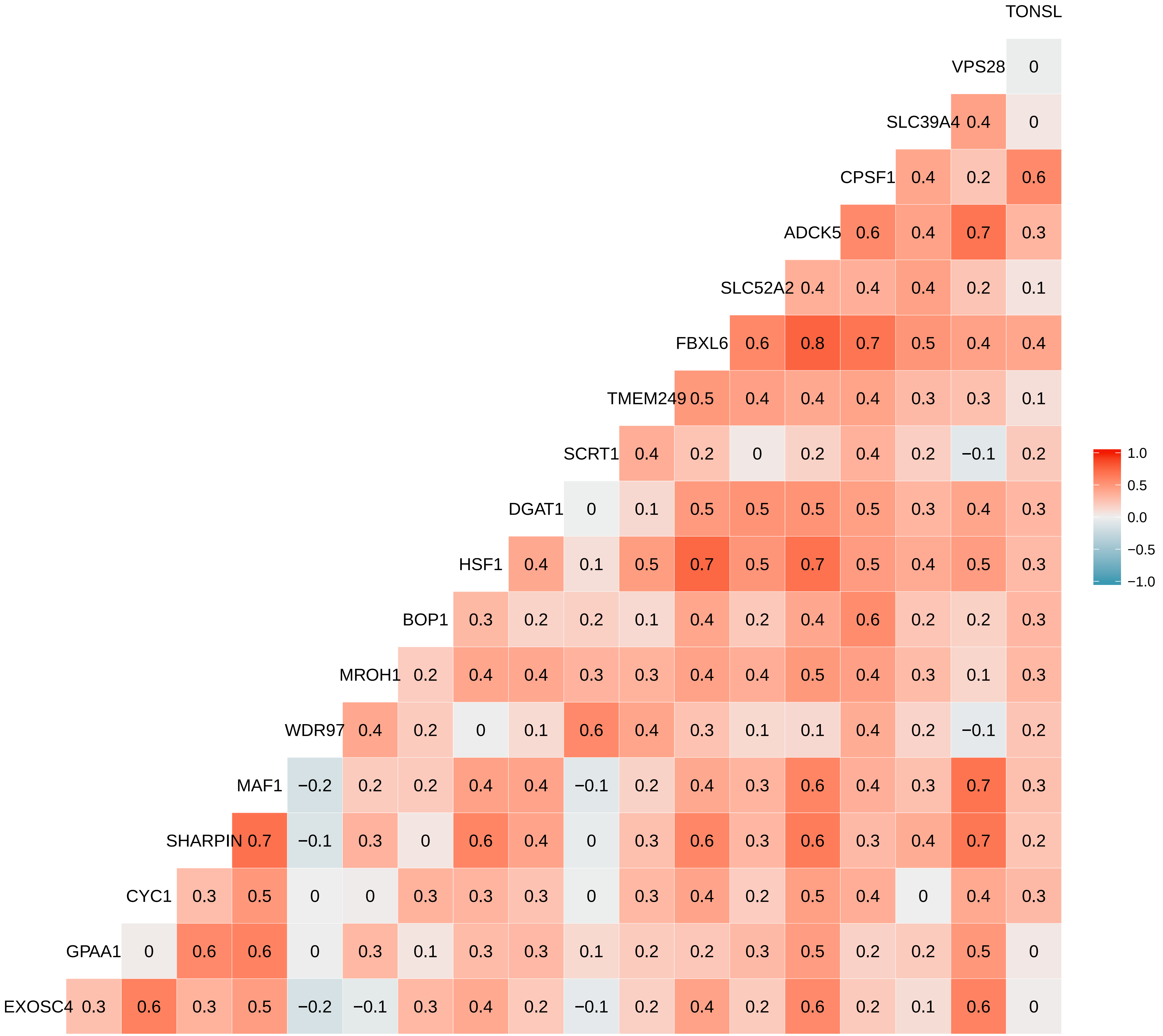

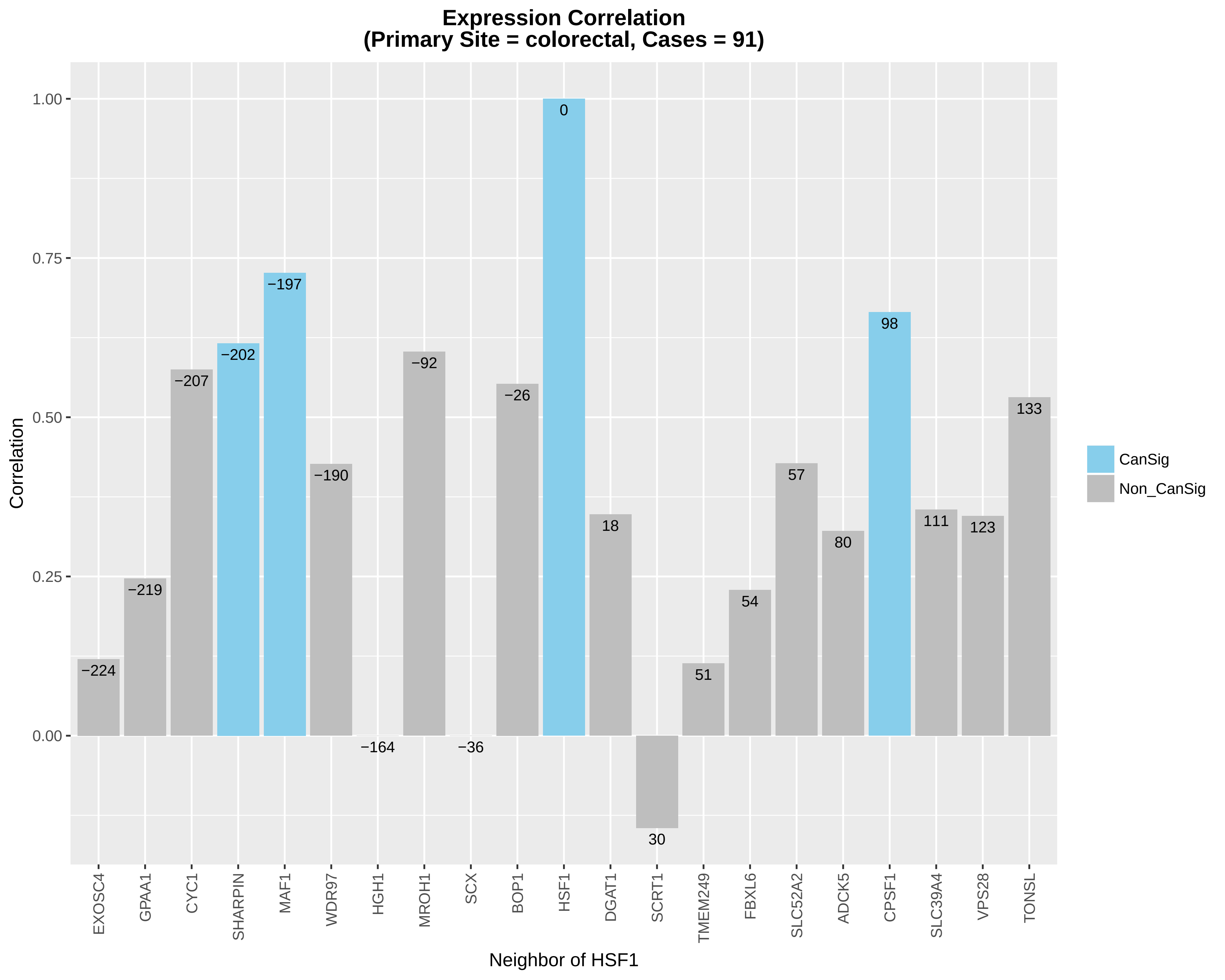

Expression Correlation
(Primary Site = colorectal, Cases = 91)
Correlation
1.00
0.75
0.50
0.25
0.00
−224
−219
−207
−202
−197
−190
−164
−92
−36
−26
0
18
30
51
54
57
80
98
111
123
133
EXOSC4
GPAA1
CYC1
SHARPIN
MAF1
WDR97
HGH1
MROH1
SCX
BOP1
HSF1
DGAT1
SCRT1
TMEM249
FBXL6
SLC52A2
ADCK5
CPSF1
SLC39A4
VPS28
TONSL
Neighbor of HSF1
CanSig
Non_CanSig

Expression Correlation Matrix
(Primary Site = colorectal, Cases = 91)

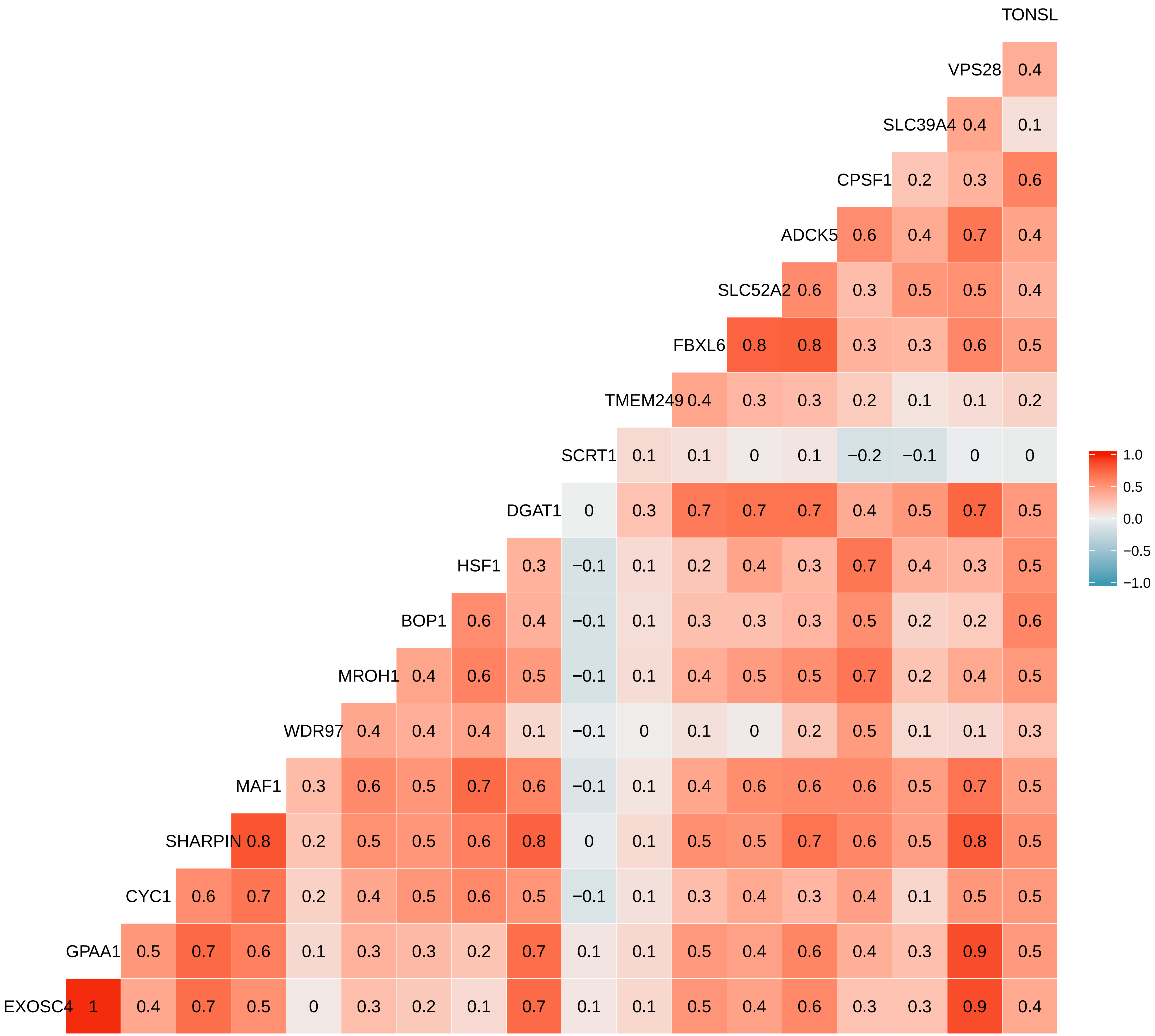

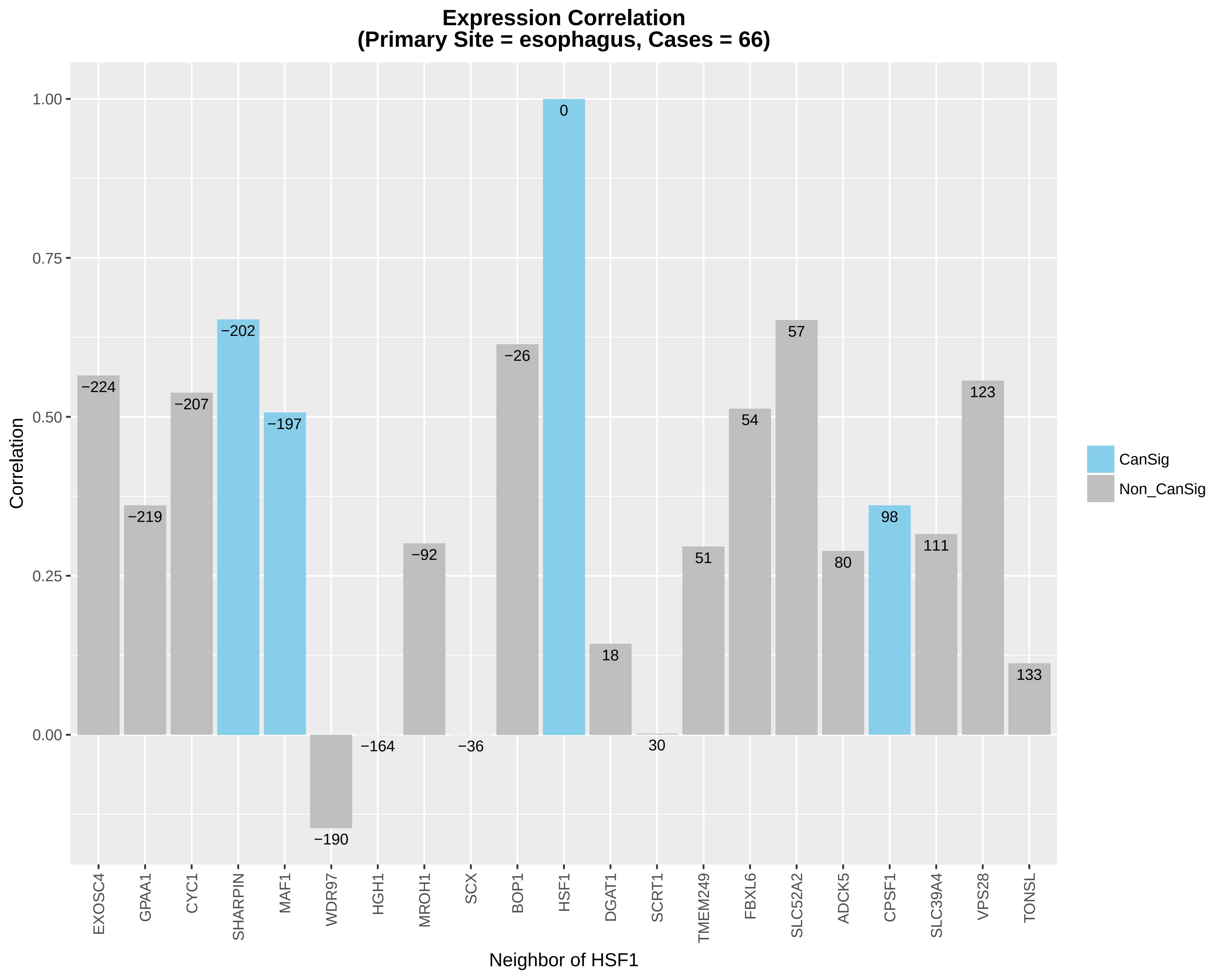
Expression Correlation
(Primary Site = esophagus, Cases = 66)
Correlation
1.00
0.75
0.50
0.25
0.00
−224
−219
−207
−202
−197
−190
−164
−92
−36
−26
0
18
30
51
54
57
80
98
111
123
133
EXOSC4
GPAA1
CYC1
SHARPIN
MAF1
WDR97
HGH1
MROH1
SCX
BOP1
HSF1
DGAT1
SCRT1
TMEM249
FBXL6
SLC52A2
ADCK5
CPSF1
SLC39A4
VPS28
TONSL
Neighbor of HSF1
CanSig
Non_CanSig

Expression Correlation Matrix
(Primary Site = esophagus, Cases = 66)

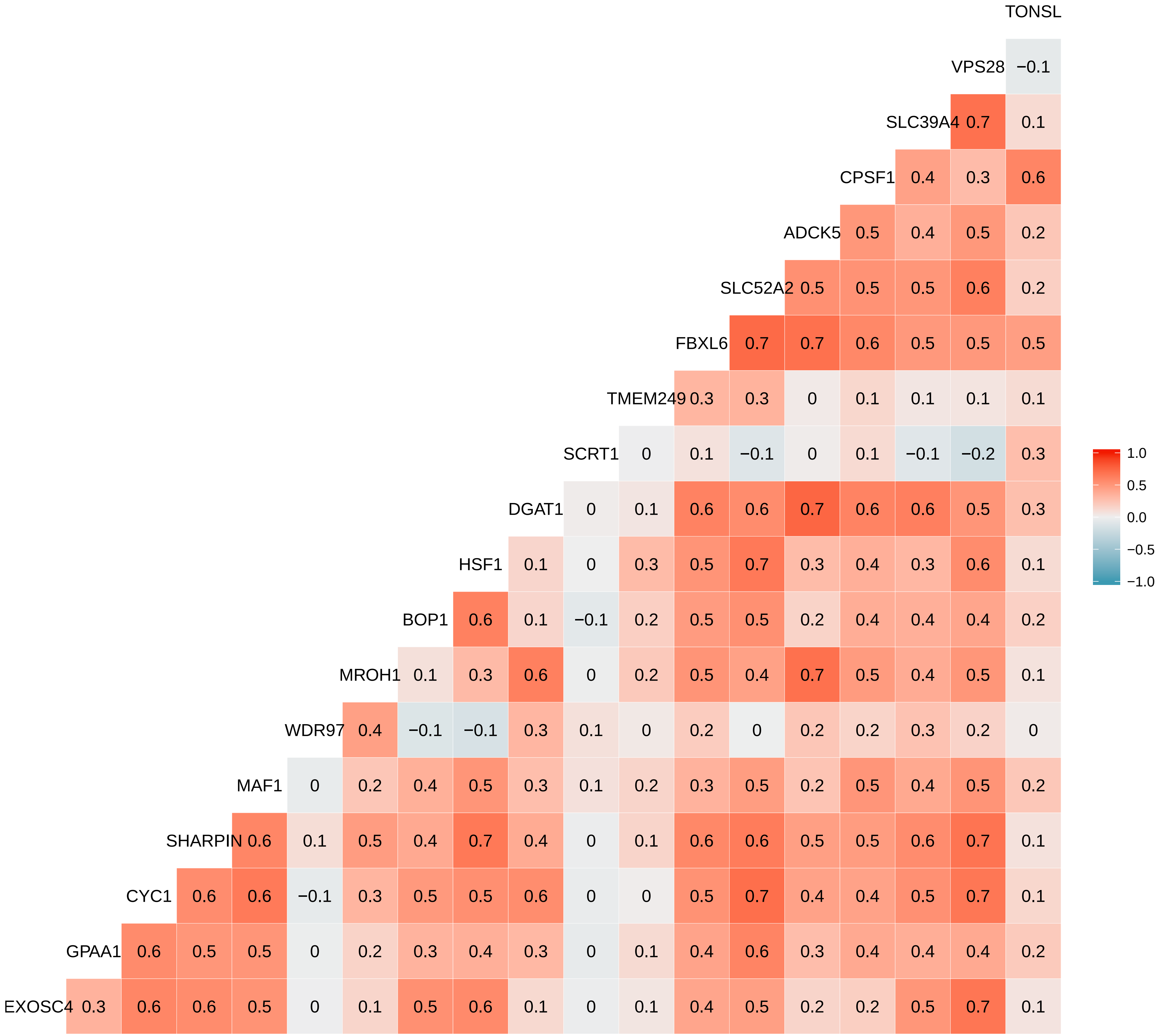

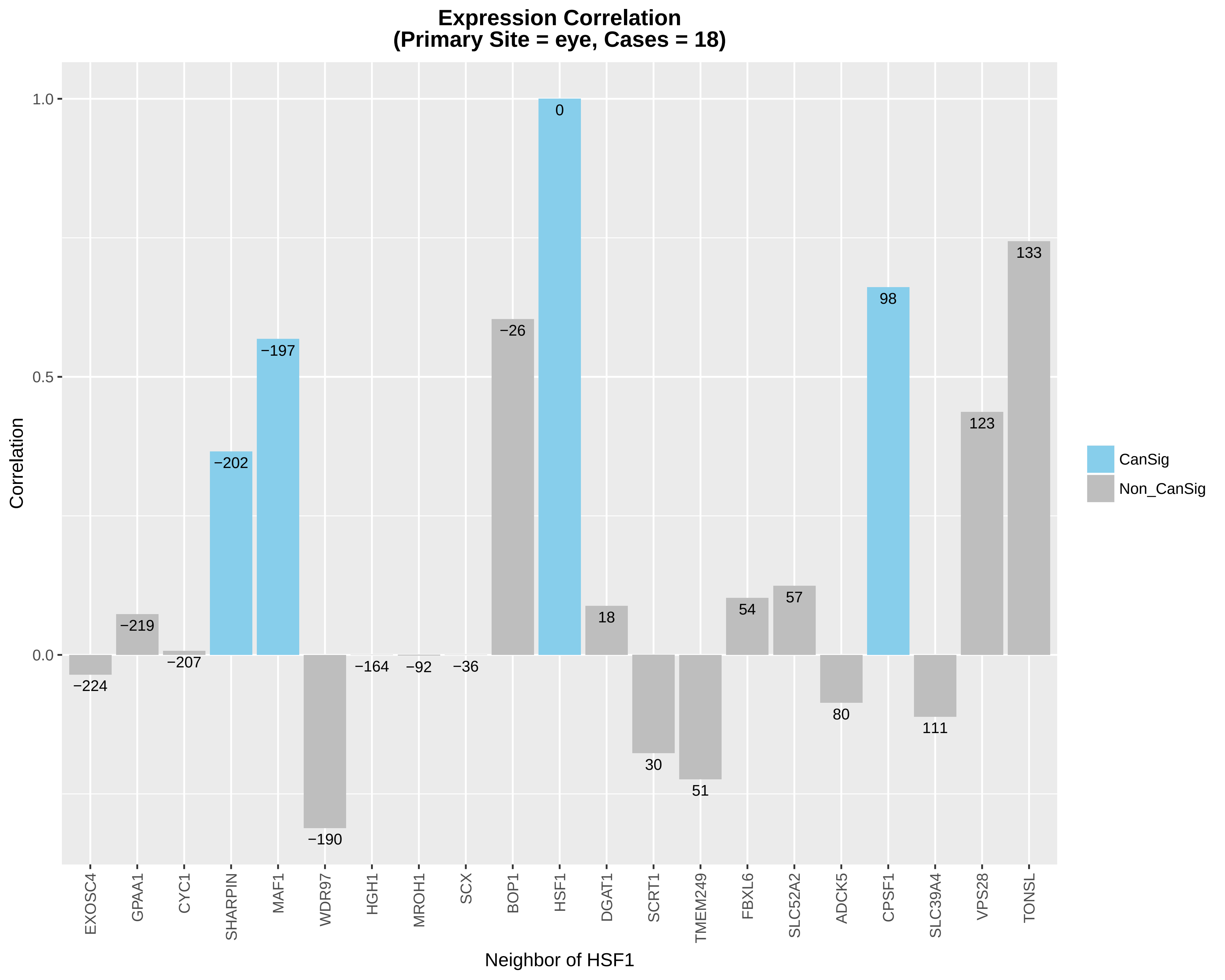

Expression Correlation
(Primary Site = eye, Cases = 18)
Correlation
1.0
0.5
0.0
0
−197
−202
−26
98
133
123
−219
−207
−224
18
54
57
−190
−164
−92
−36
30
51
80
111
EXOSC4
GPAA1
CYC1
SHARPIN
MAF1
WDR97
HGH1
MROH1
SCX
BOP1
HSF1
DGAT1
SCRT1
TMEM249
FBXL6
SLC52A2
ADCK5
CPSF1
SLC39A4
VPS28
TONSL
Neighbor of HSF1
CanSig
Non_CanSig

Expression Correlation Matrix
(Primary Site = eye, Cases = 18)

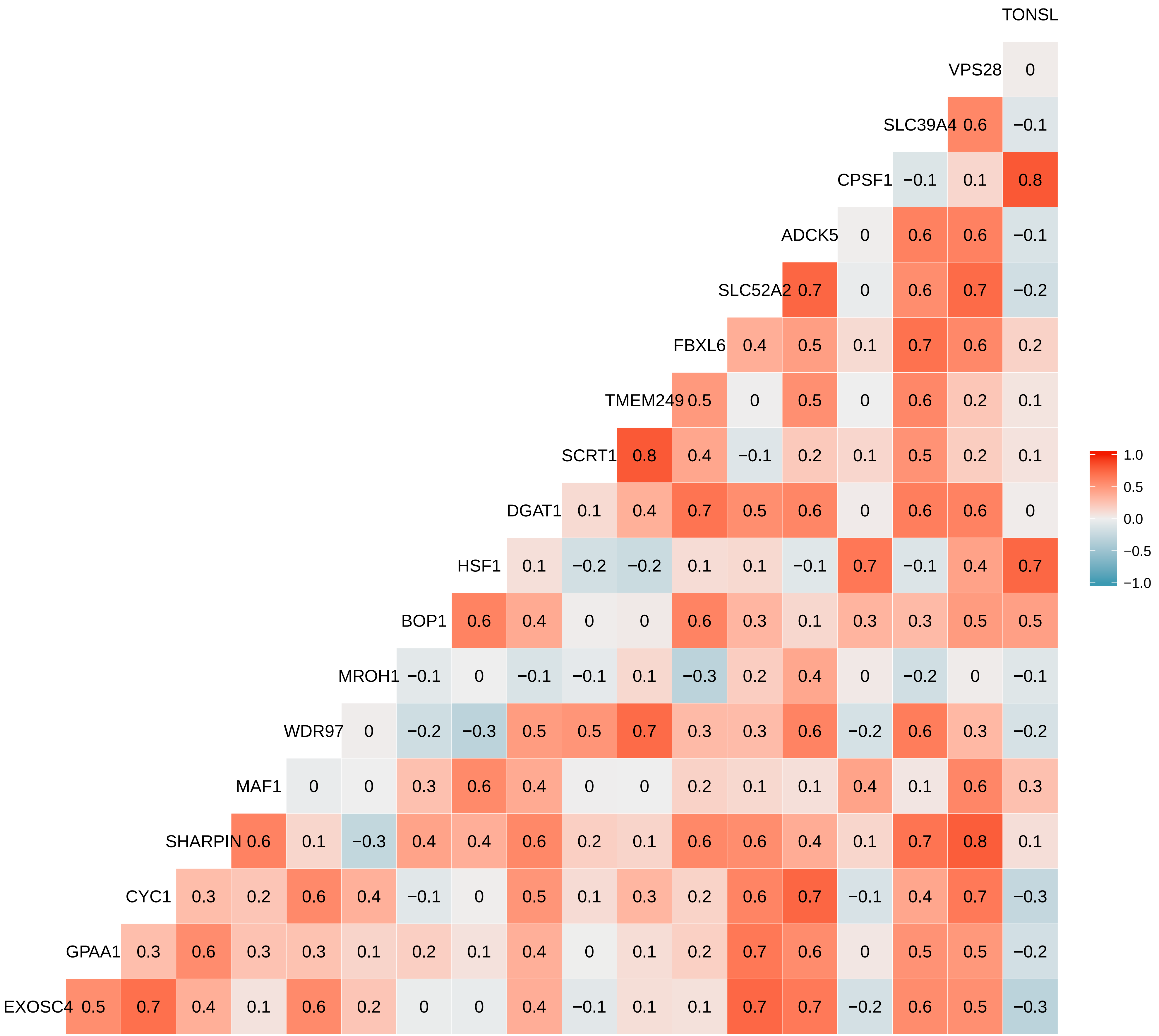

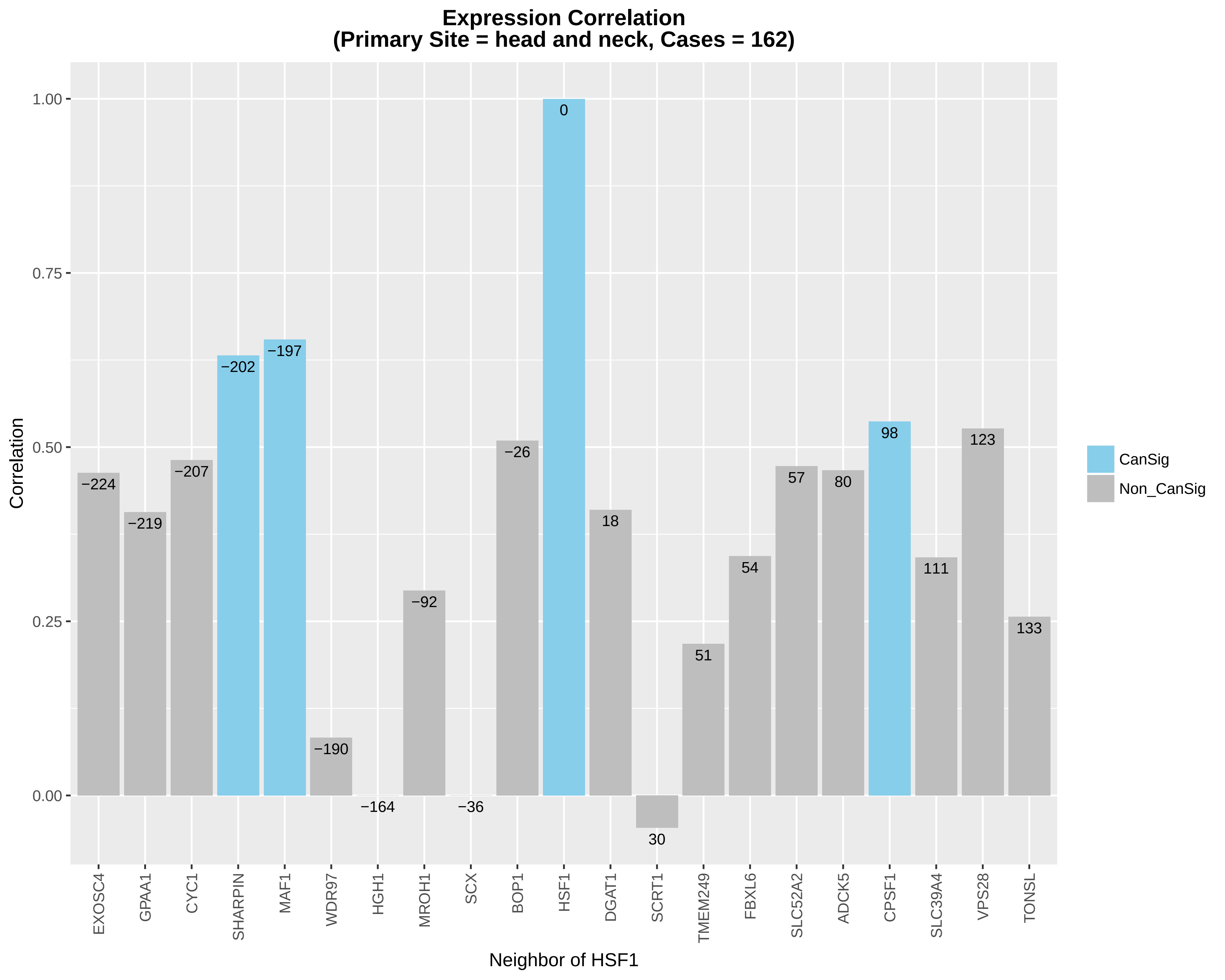

Expression Correlation
(Primary Site = head and neck, Cases = 162)
Correlation
1.00
0.75
0.50
0.25
0.00
0
−202
−197
−224
−219
−207
−190
−164
−92
−36
−26
18
30
51
54
57
80
98
111
123
133
CanSig
Non_CanSig
EXOSC4
GPAA1
CYC1
SHARPIN
MAF1
WDR97
HGH1
MROH1
SCX
BOP1
HSF1
DGAT1
SCRT1
TMEM249
FBXL6
SLC52A2
ADCK5
CPSF1
SLC39A4
VPS28
TONSL
Neighbor of HSF1

Expression Correlation Matrix
(Primary Site = head and neck, Cases = 162)

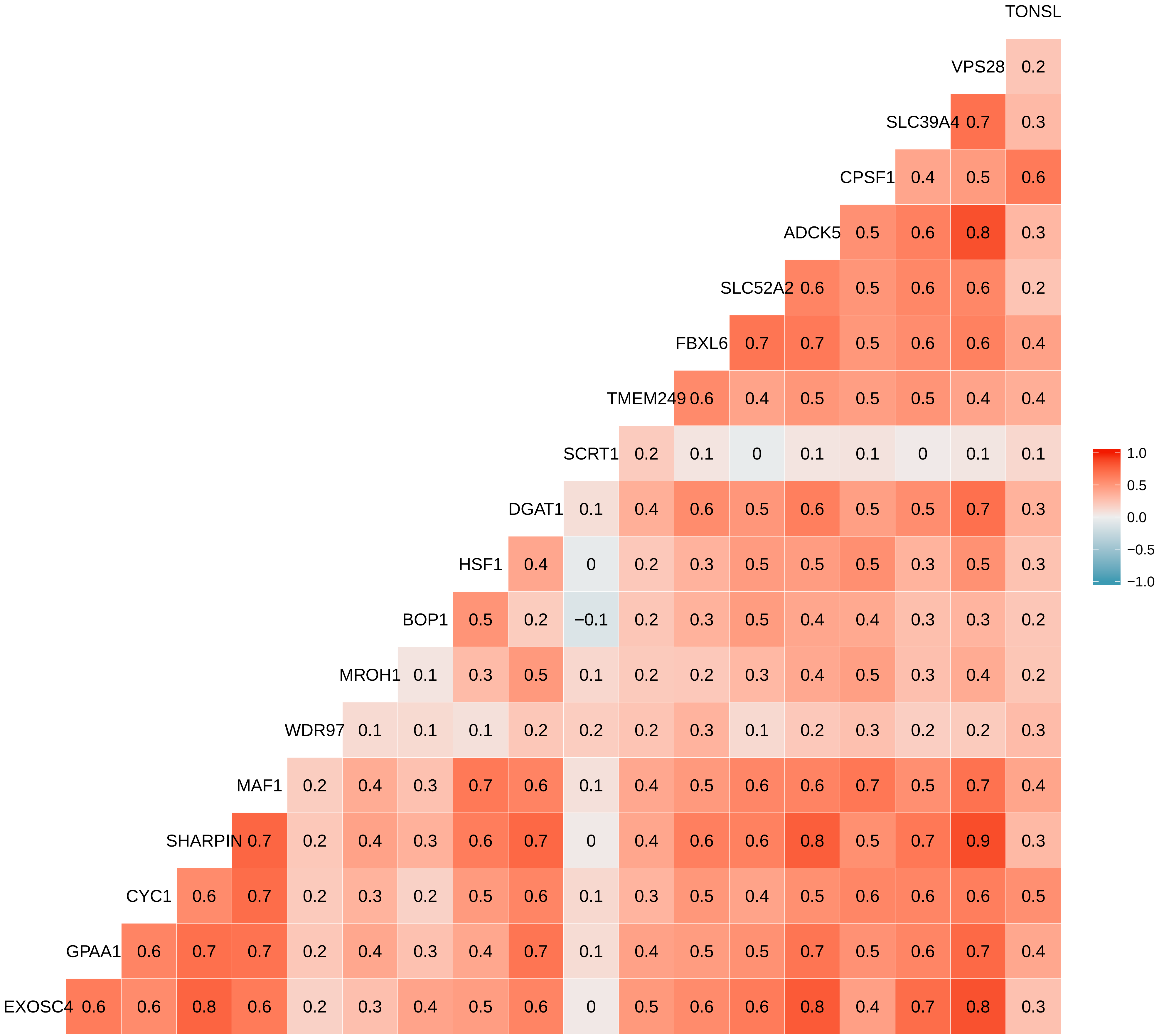

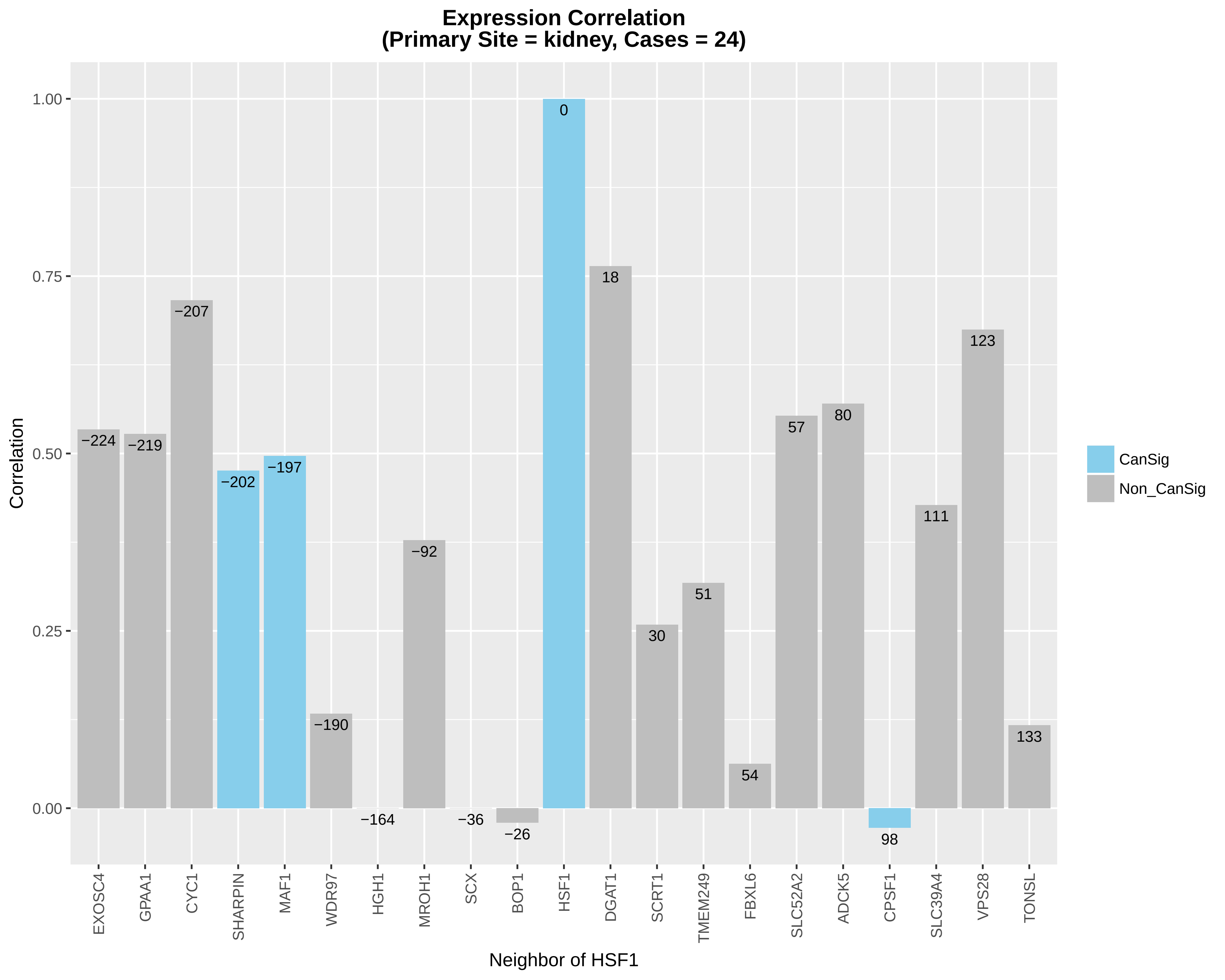

Expression Correlation
(Primary Site = kidney, Cases = 24)
Correlation
1.00
0.75
0.50
0.25
0.00
−224
−219
−207
−202
−197
−190
−164
−92
−36
−26
0
18
30
51
54
57
80
98
111
123
133
EXOSC4
GPAA1
CYC1
SHARPIN
MAF1
WDR97
HGH1
MROH1
SCX
BOP1
HSF1
DGAT1
SCRT1
TMEM249
FBXL6
SLC52A2
ADCK5
CPSF1
SLC39A4
VPS28
TONSL
Neighbor of HSF1
CanSig
Non_CanSig

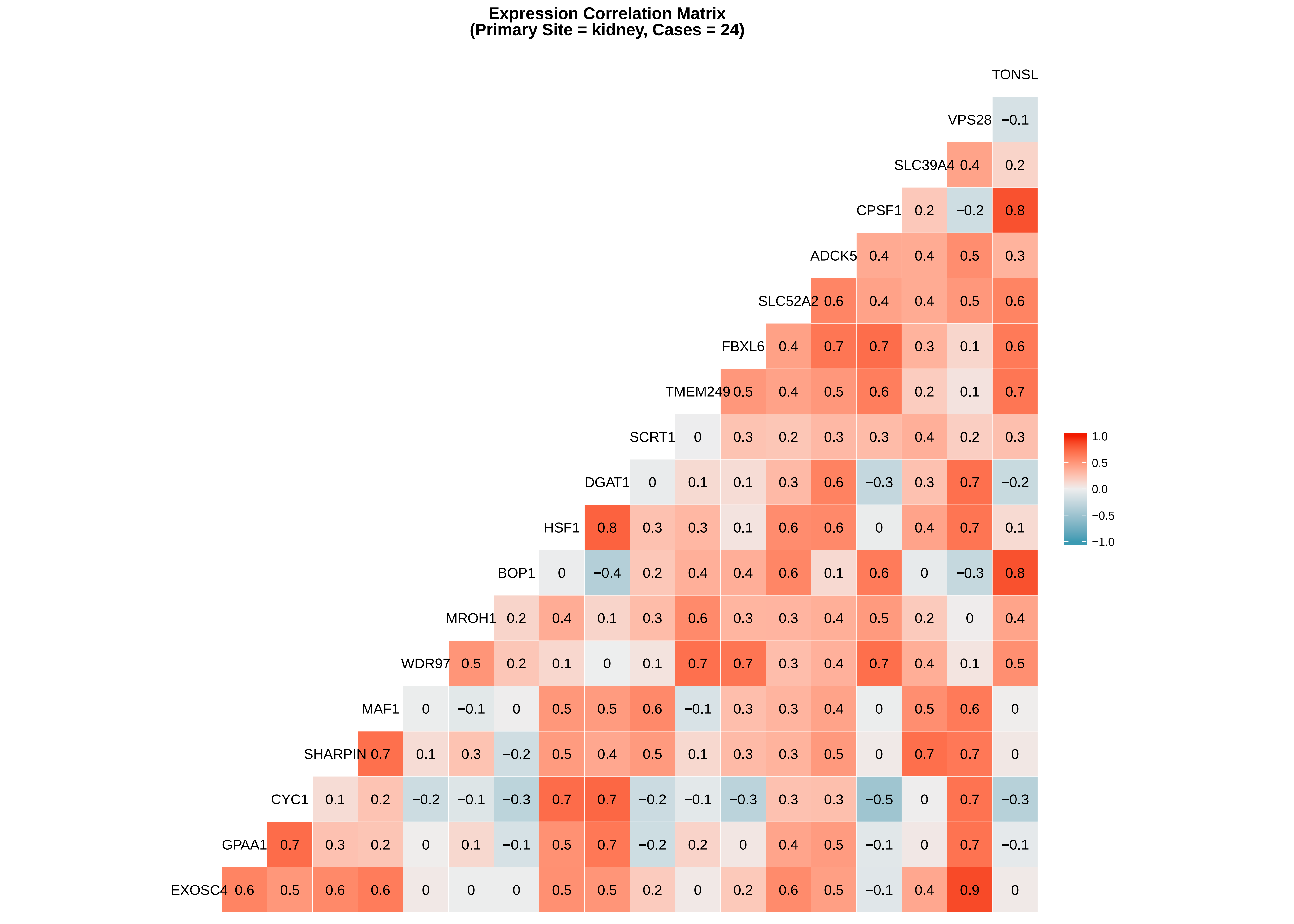
Expression Correlation Matrix
(Primary Site = kidney, Cases = 24)
TONSL
VPS28
SLC39A4
CPSF1
ADCK5
SLC52A2
FBXL6
TMEM249
SCRT1
DGAT1
HSF1
BOP1
MROH1
WDR97
MAF1
SHARPIN
CYC1
GPAA1
EXOSC4
1.0
0.5
0.0
−0.5
−1.0

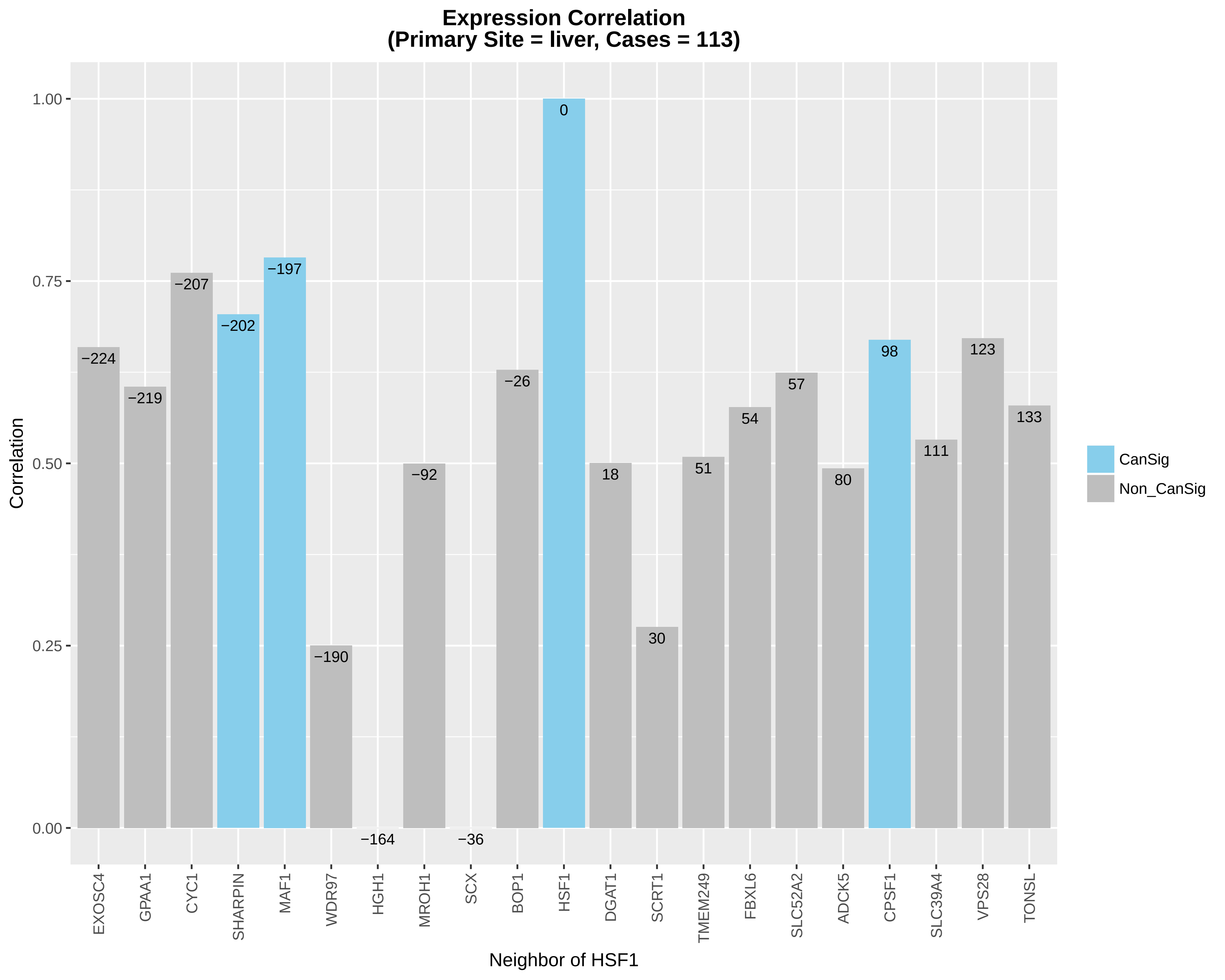

Expression Correlation
(Primary Site = liver, Cases = 113)
Correlation
1.00
0.75
0.50
0.25
0.00
−224
−219
−207
−202
−197
−190
−164
−92
−36
−26
0
18
30
51
54
57
80
98
111
123
133
EXOSC4
GPAA1
CYC1
SHARPIN
MAF1
WDR97
HGH1
MROH1
SCX
BOP1
HSF1
DGAT1
SCRT1
TMEM249
FBXL6
SLC52A2
ADCK5
CPSF1
SLC39A4
VPS28
TONSL
Neighbor of HSF1
CanSig
Non_CanSig

Expression Correlation Matrix
(Primary Site = liver, Cases = 113)

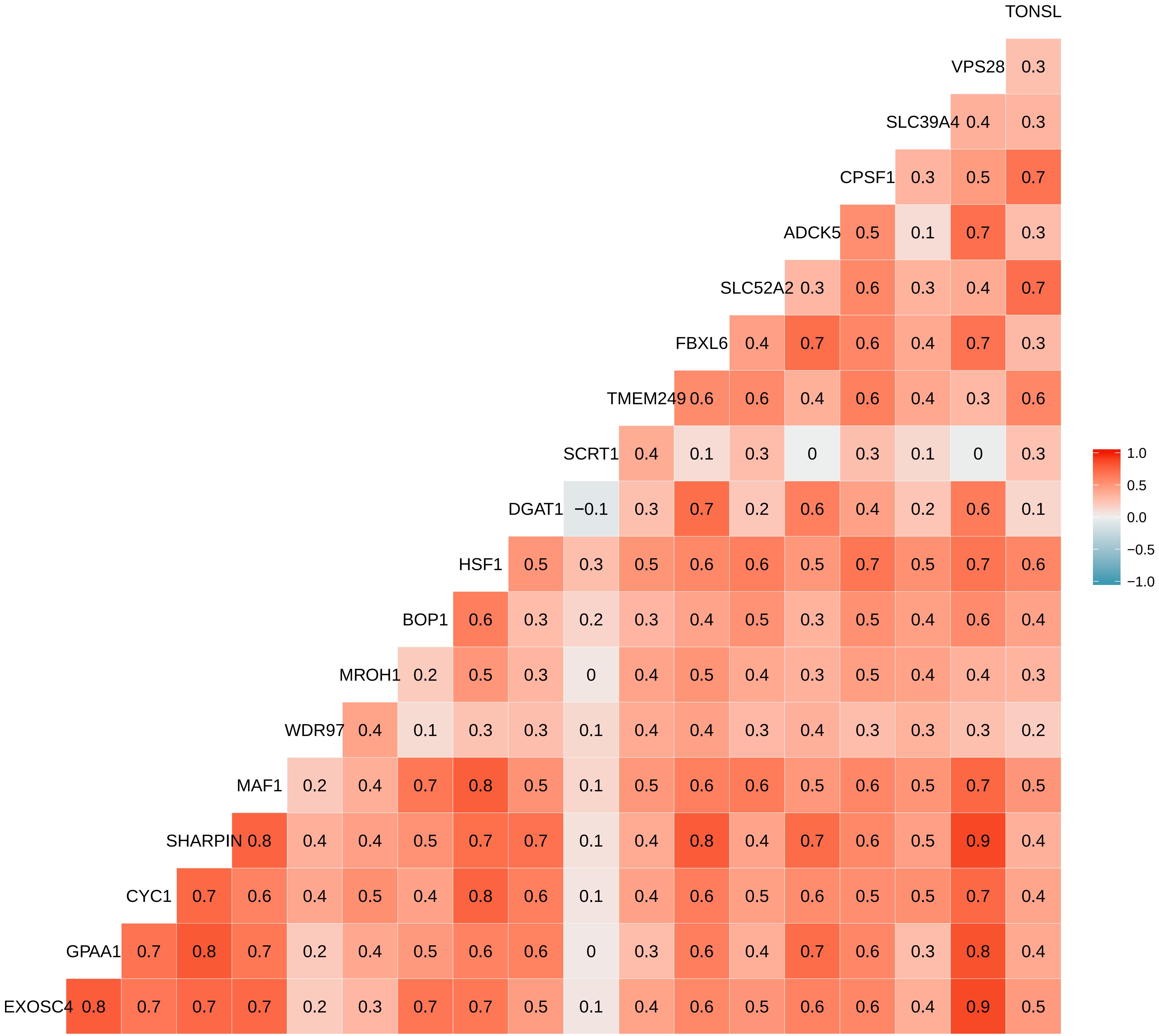

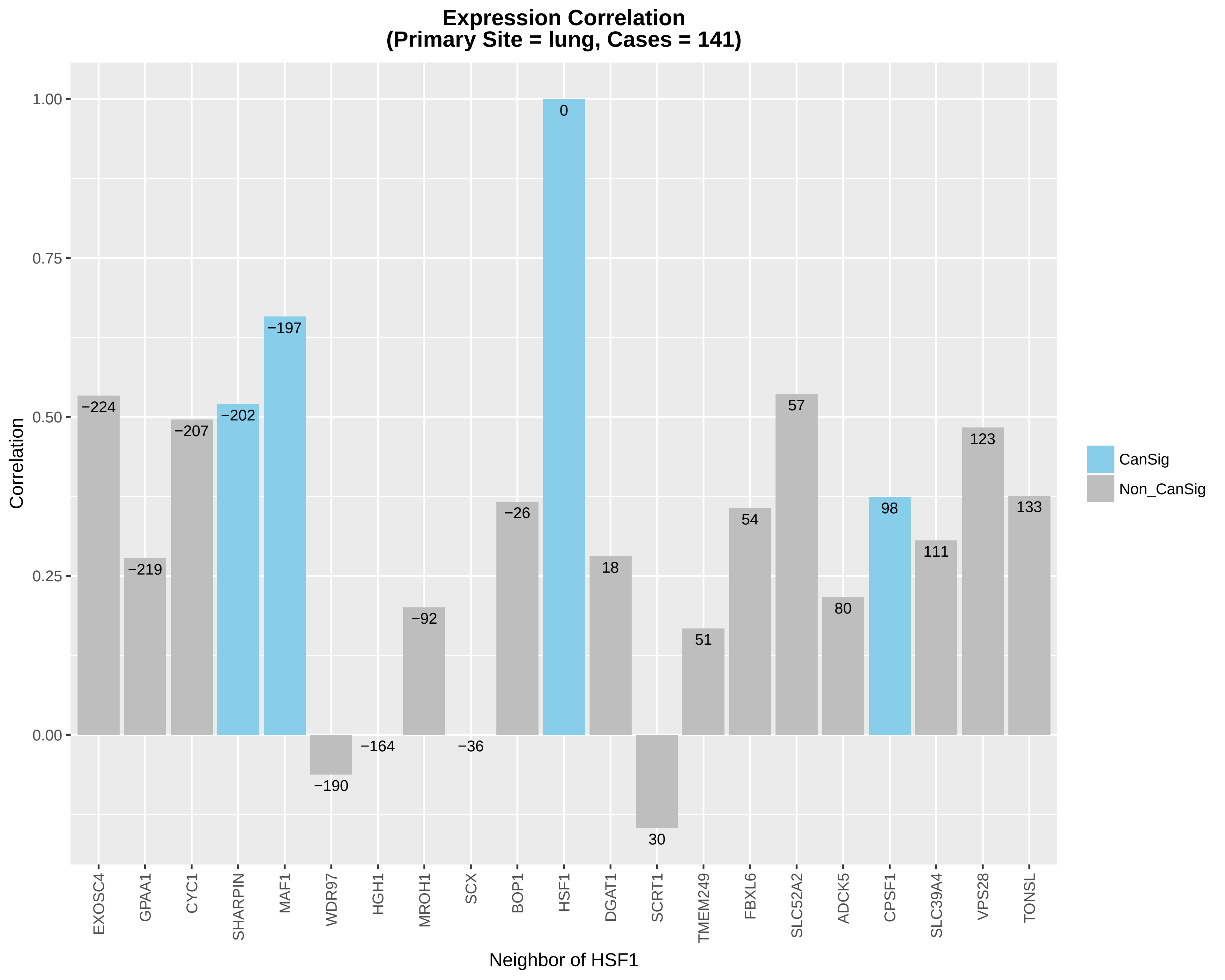

Expression Correlation
(Primary Site = lung, Cases = 141)
Correlation
1.00
0.75
0.50
0.25
0.00
Neighbor of HSF1
EXOSC4
GPAA1
CYC1
SHARPIN
MAF1
WDR97
HGH1
MROH1
SCX
BOP1
HSF1
DGAT1
SCRT1
TMEM249
FBXL6
SLC52A2
ADCK5
CPSF1
SLC39A4
VPS28
TONSL
−224
−219
−207
−202
−197
−190
−164
−92
−36
−26
0
18
30
51
54
57
80
98
111
123
133
CanSig
Non_CanSig

Expression Correlation Matrix
(Primary Site = lung, Cases = 141)

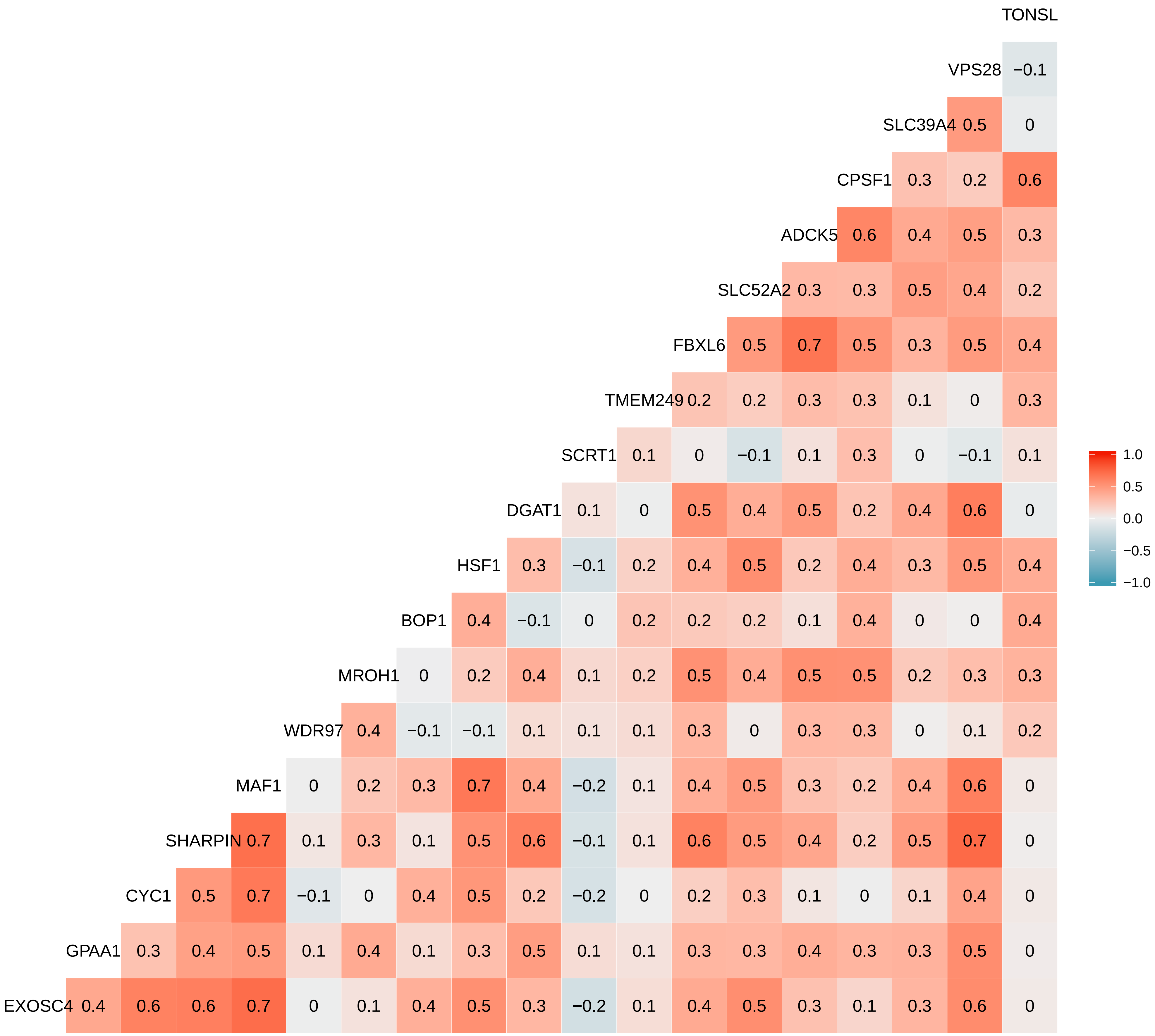

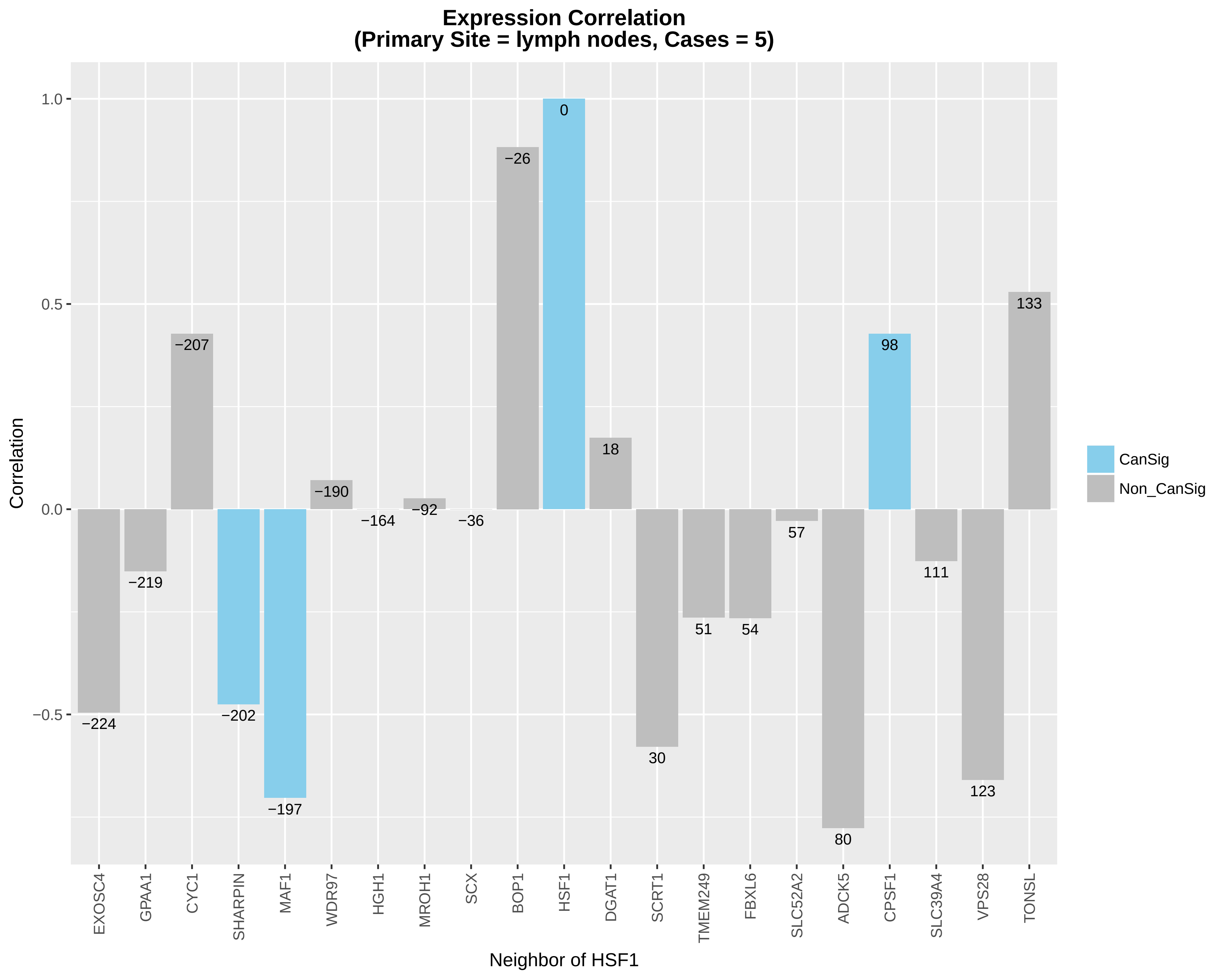

Expression Correlation
(Primary Site = lymph nodes, Cases = 5)
Correlation
1.0
0.5
0.0
−0.5
0
−26
−207
98
133
18
−190
−164
−92
−36
57
−219
111
51
54
−224
−202
30
123
−197
80
EXOSC4
GPAA1
CYC1
SHARPIN
MAF1
WDR97
HGH1
MROH1
SCX
BOP1
HSF1
DGAT1
SCRT1
TMEM249
FBXL6
SLC52A2
ADCK5
CPSF1
SLC39A4
VPS28
TONSL
Neighbor of HSF1
CanSig
Non_CanSig

Expression Correlation Matrix
(Primary Site = lymph nodes, Cases = 5)

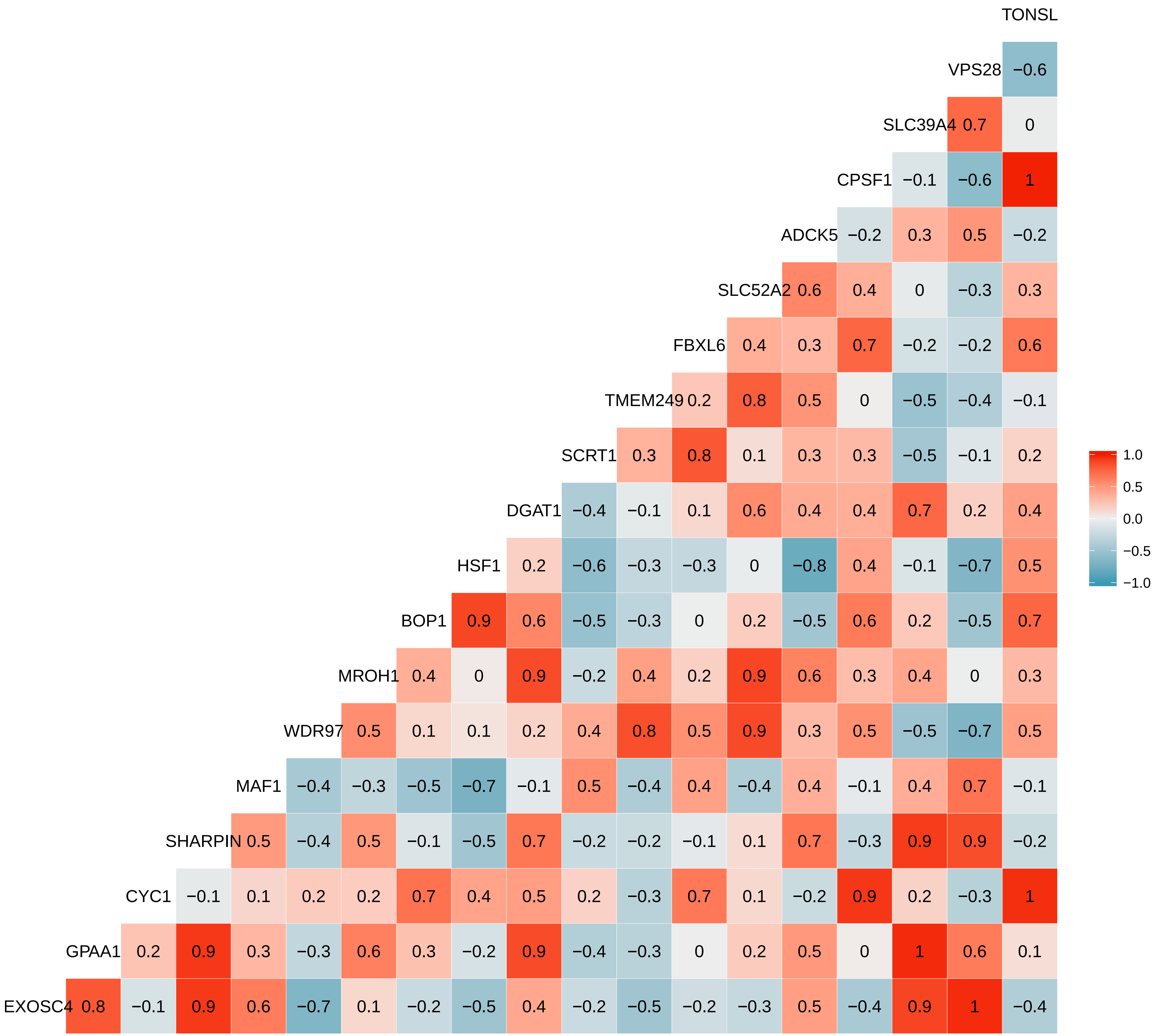

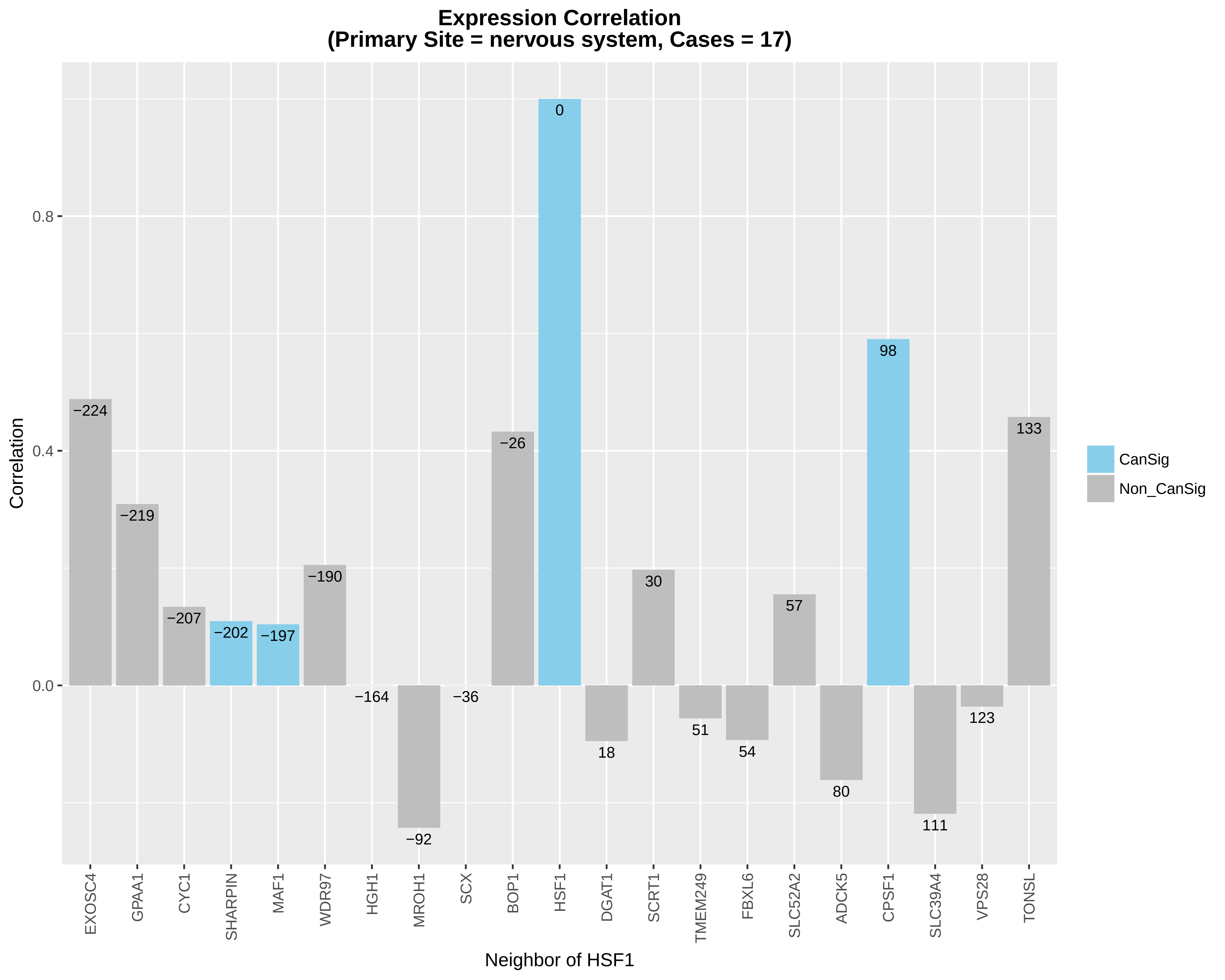

Expression Correlation
(Primary Site = nervous system, Cases = 17)
Correlation
0.8
0.4
0.0
−224
−219
−207
−202
−197
−190
−164
−92
−36
−26
0
18
30
51
54
57
80
98
111
123
133
EXOSC4
GPAA1
CYC1
SHARPIN
MAF1
WDR97
HGH1
MROH1
SCX
BOP1
HSF1
DGAT1
SCRT1
TMEM249
FBXL6
SLC52A2
ADCK5
CPSF1
SLC39A4
VPS28
TONSL
Neighbor of HSF1
CanSig
Non_CanSig

Expression Correlation Matrix
(Primary Site = nervous system, Cases = 17)

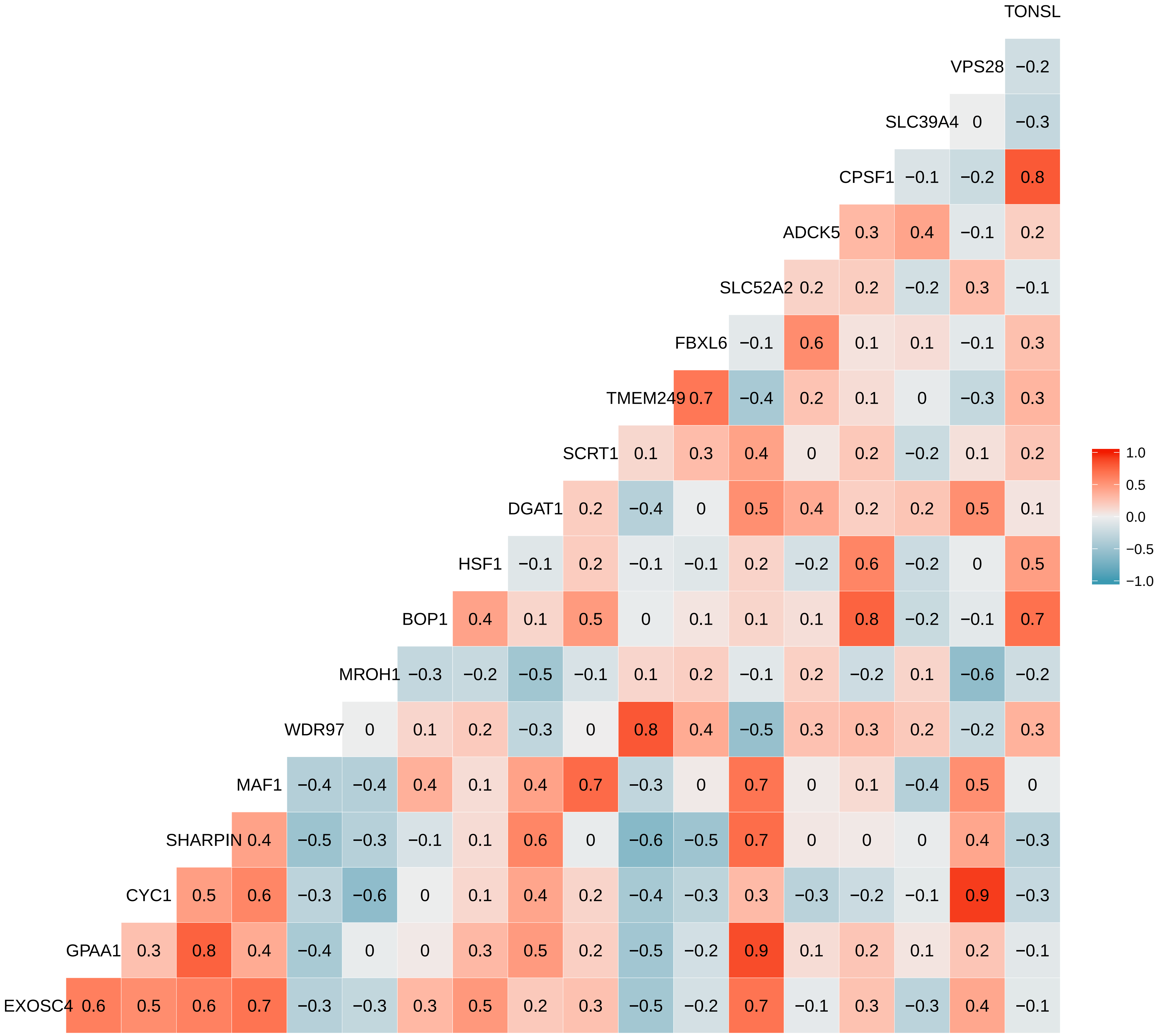

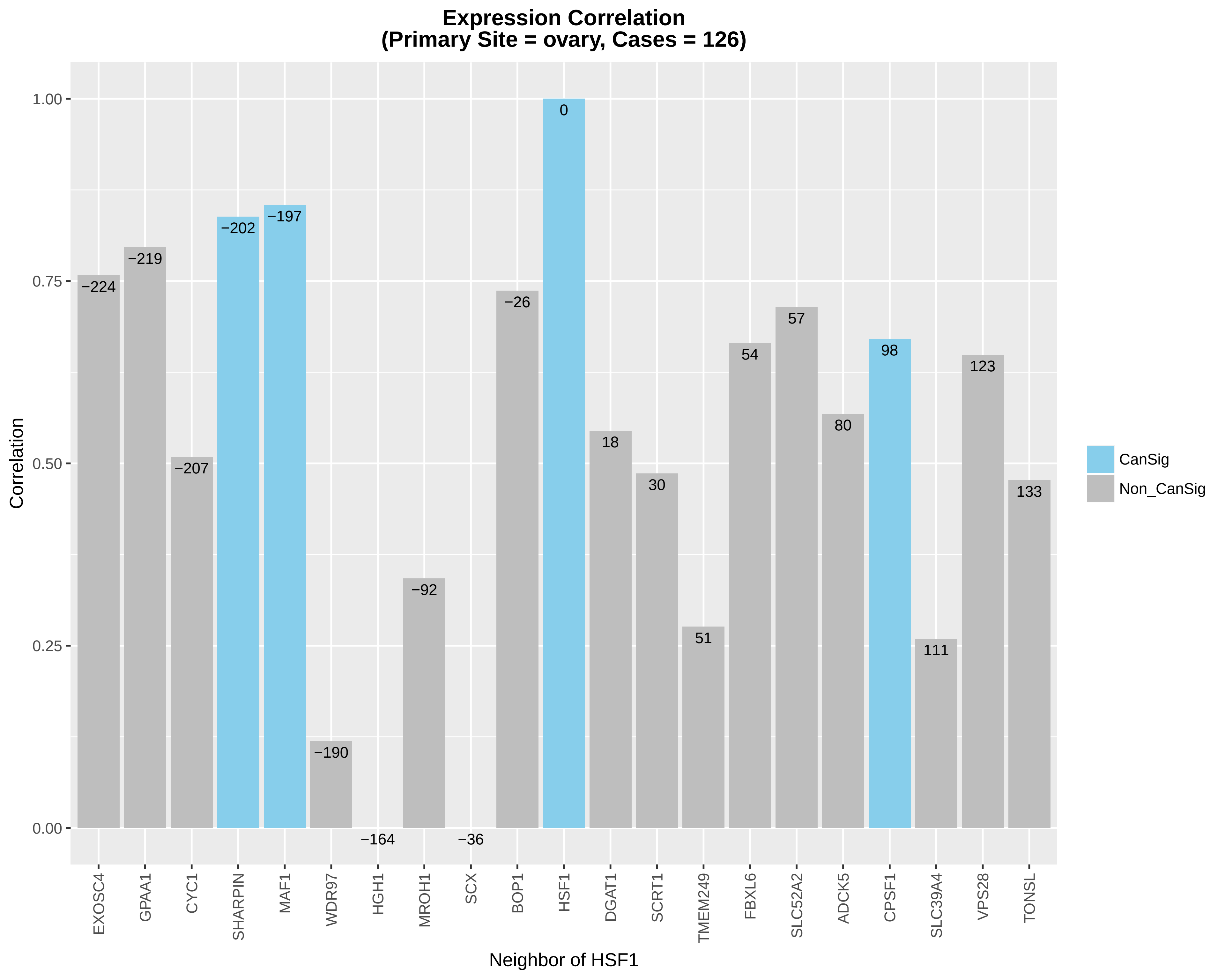
Expression Correlation
(Primary Site = ovary, Cases = 126)
Correlation
1.00
0.75
0.50
0.25
0.00
−224
−219
−207
−202
−197
−190
−164
−92
−36
−26
0
18
30
51
54
57
80
98
111
123
133
EXOSC4
GPAA1
CYC1
SHARPIN
MAF1
WDR97
HGH1
MROH1
SCX
BOP1
HSF1
DGAT1
SCRT1
TMEM249
FBXL6
SLC52A2
ADCK5
CPSF1
SLC39A4
VPS28
TONSL
Neighbor of HSF1
CanSig
Non_CanSig

Expression Correlation Matrix
(Primary Site = ovary, Cases = 126)

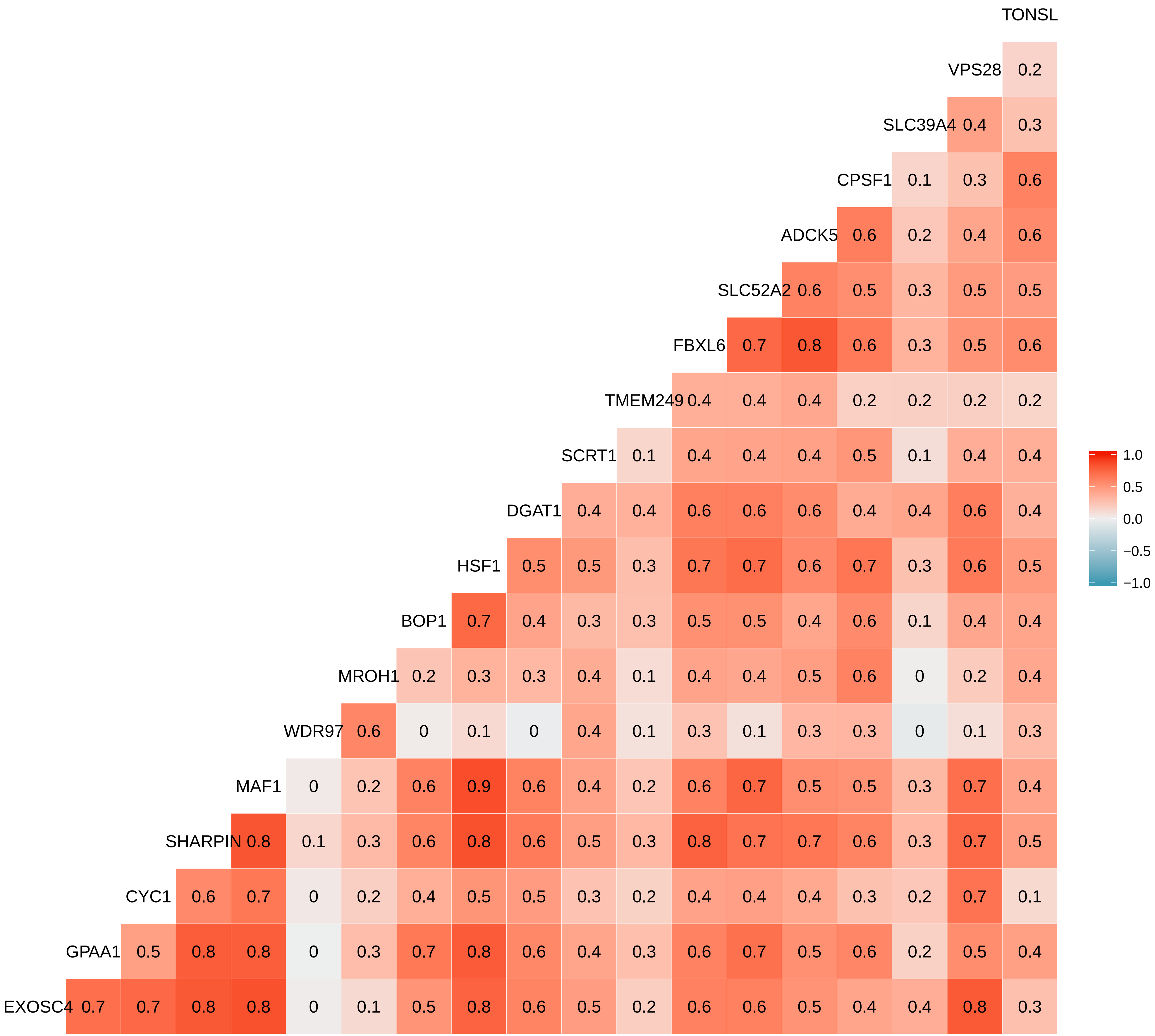

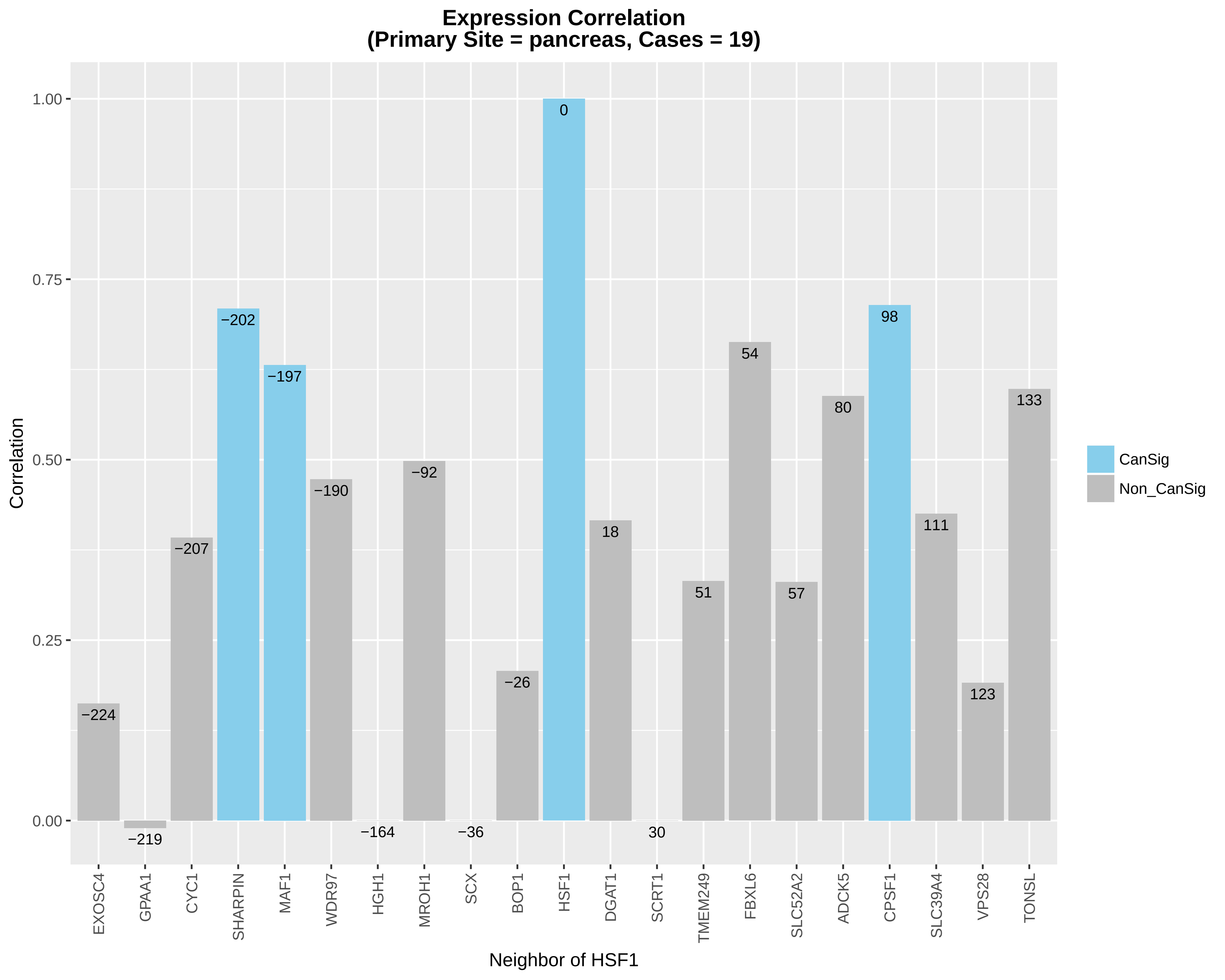

Expression Correlation
(Primary Site = pancreas, Cases = 19)
Correlation
1.00
0.75
0.50
0.25
0.00
−224
−219
−207
−202
−197
−190
−164
−92
−36
−26
0
18
30
51
54
57
80
98
111
123
133
EXOSC4
GPAA1
CYC1
SHARPIN
MAF1
WDR97
HGH1
MROH1
SCX
BOP1
HSF1
DGAT1
SCRT1
TMEM249
FBXL6
SLC52A2
ADCK5
CPSF1
SLC39A4
VPS28
TONSL
Neighbor of HSF1
CanSig
Non_CanSig

Expression Correlation Matrix
(Primary Site = pancreas, Cases = 19)

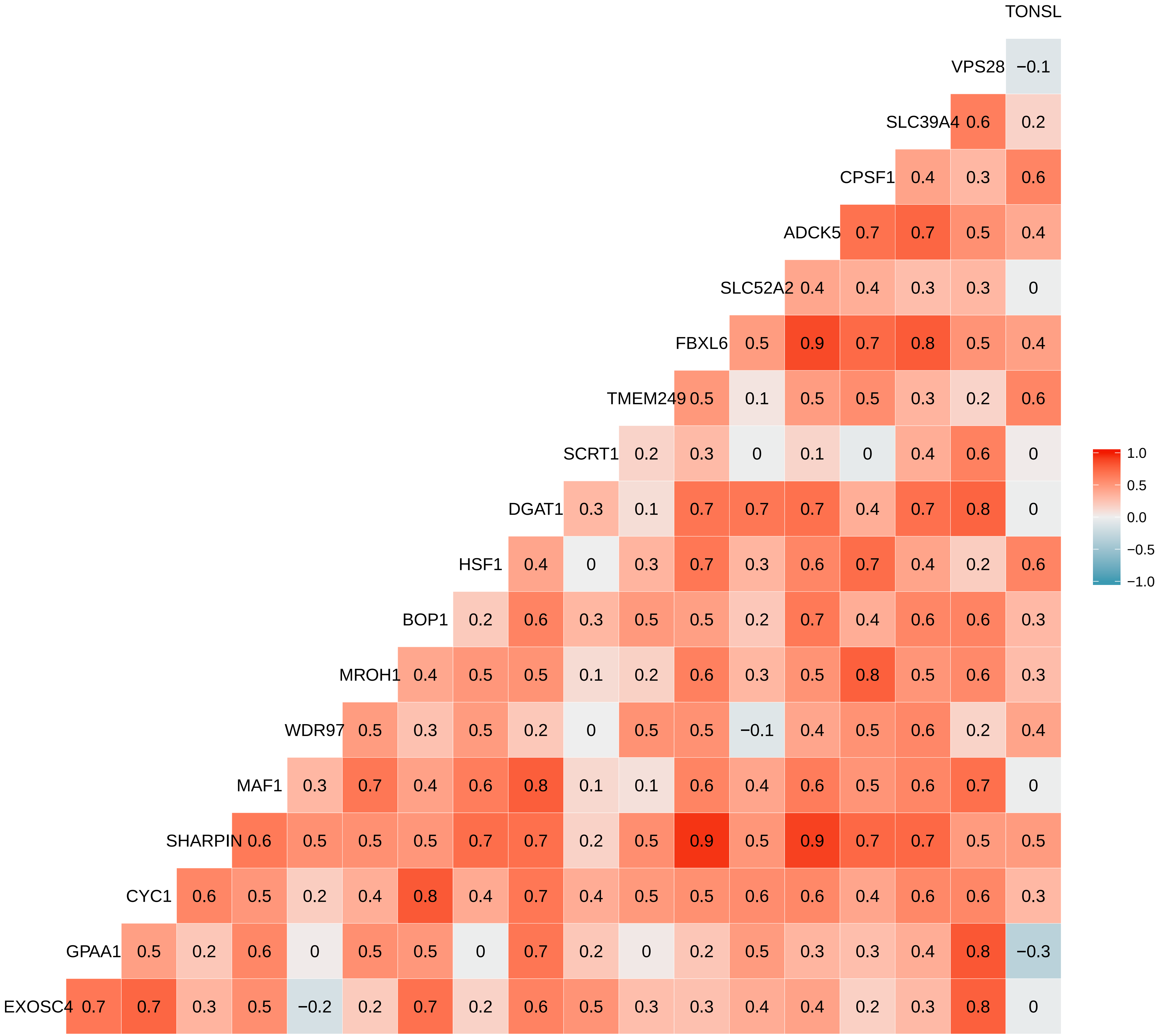

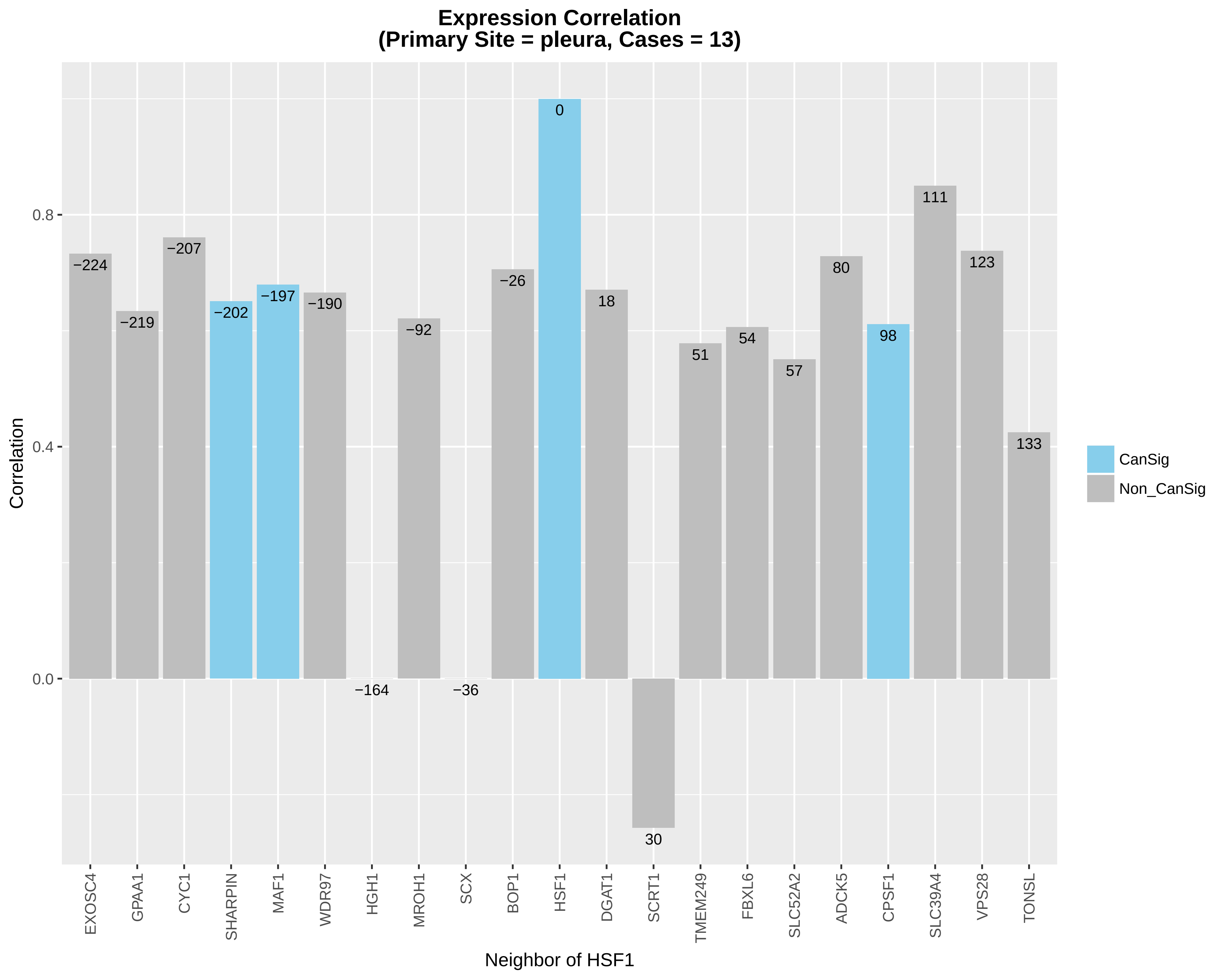
Expression Correlation
(Primary Site = pleura, Cases = 13)
Correlation
0.8
0.4
0.0
−224
−219
−207
−202
−197
−190
−164
−92
−36
−26
0
18
30
51
54
57
80
98
111
123
133
EXOSC4
GPAA1
CYC1
SHARPIN
MAF1
WDR97
HGH1
MROH1
SCX
BOP1
HSF1
DGAT1
SCRT1
TMEM249
FBXL6
SLC52A2
ADCK5
CPSF1
SLC39A4
VPS28
TONSL
Neighbor of HSF1
CanSig
Non_CanSig

Expression Correlation Matrix
(Primary Site = pleura, Cases = 13)

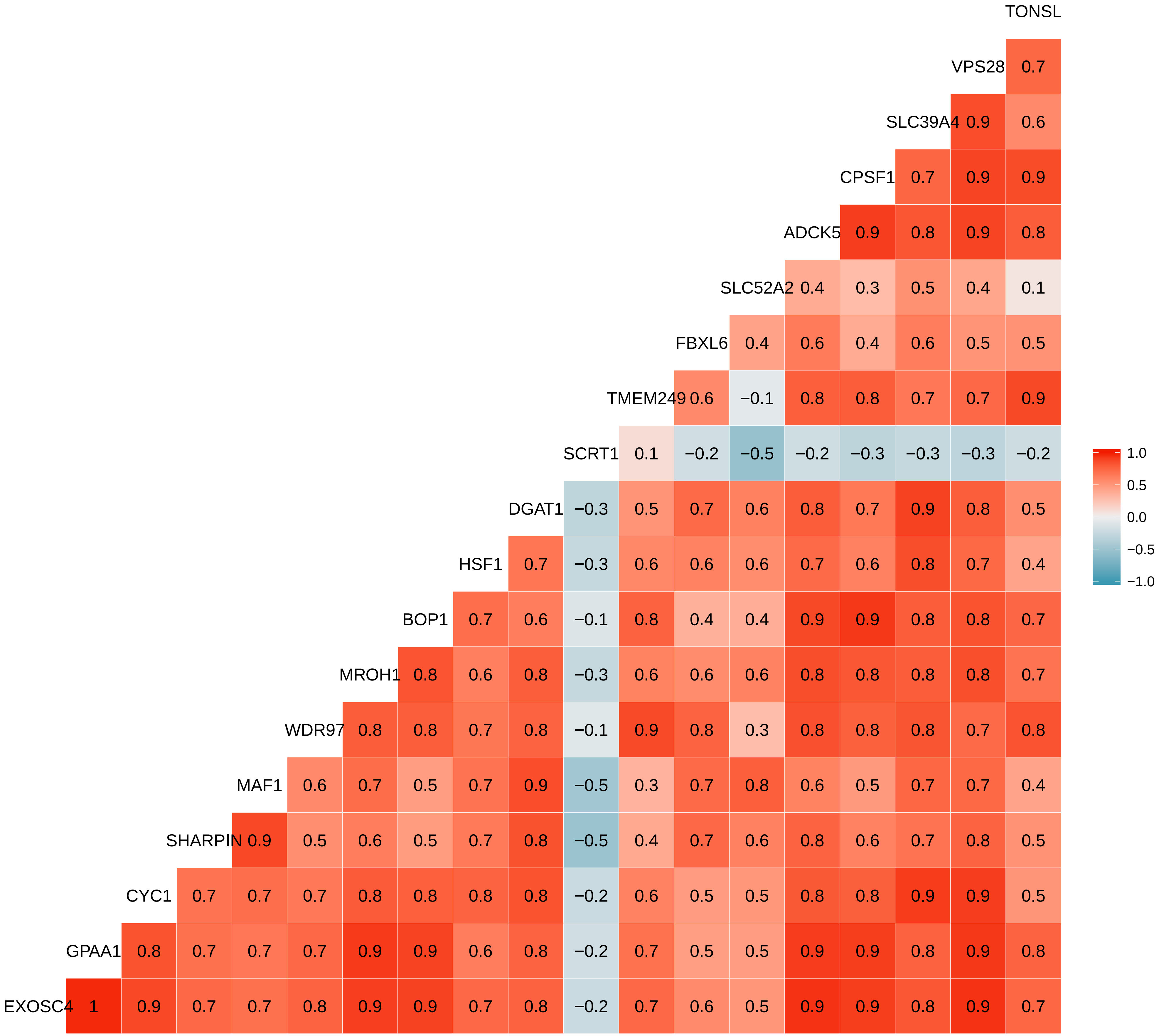

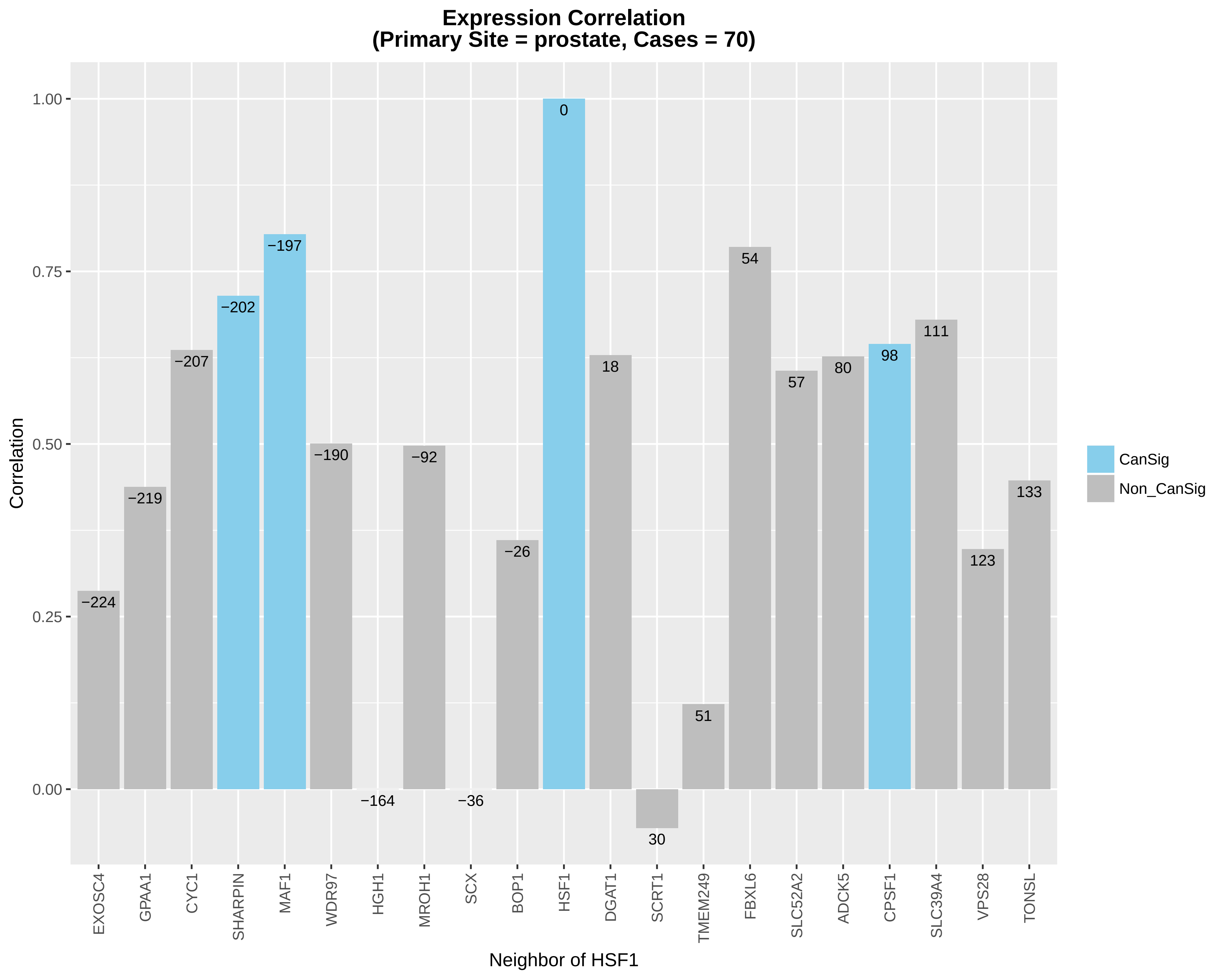
Expression Correlation
(Primary Site = prostate, Cases = 70)
Correlation
1.00
0.75
0.50
0.25
0.00
−224
−219
−207
−202
−197
−190
−164
−92
−36
−26
0
18
30
51
54
57
80
98
111
123
133
EXOSC4
GPAA1
CYC1
SHARPIN
MAF1
WDR97
HGH1
MROH1
SCX
BOP1
HSF1
DGAT1
SCRT1
TMEM249
FBXL6
SLC52A2
ADCK5
CPSF1
SLC39A4
VPS28
TONSL
Neighbor of HSF1
CanSig
Non_CanSig

Expression Correlation Matrix
(Primary Site = prostate, Cases = 70)

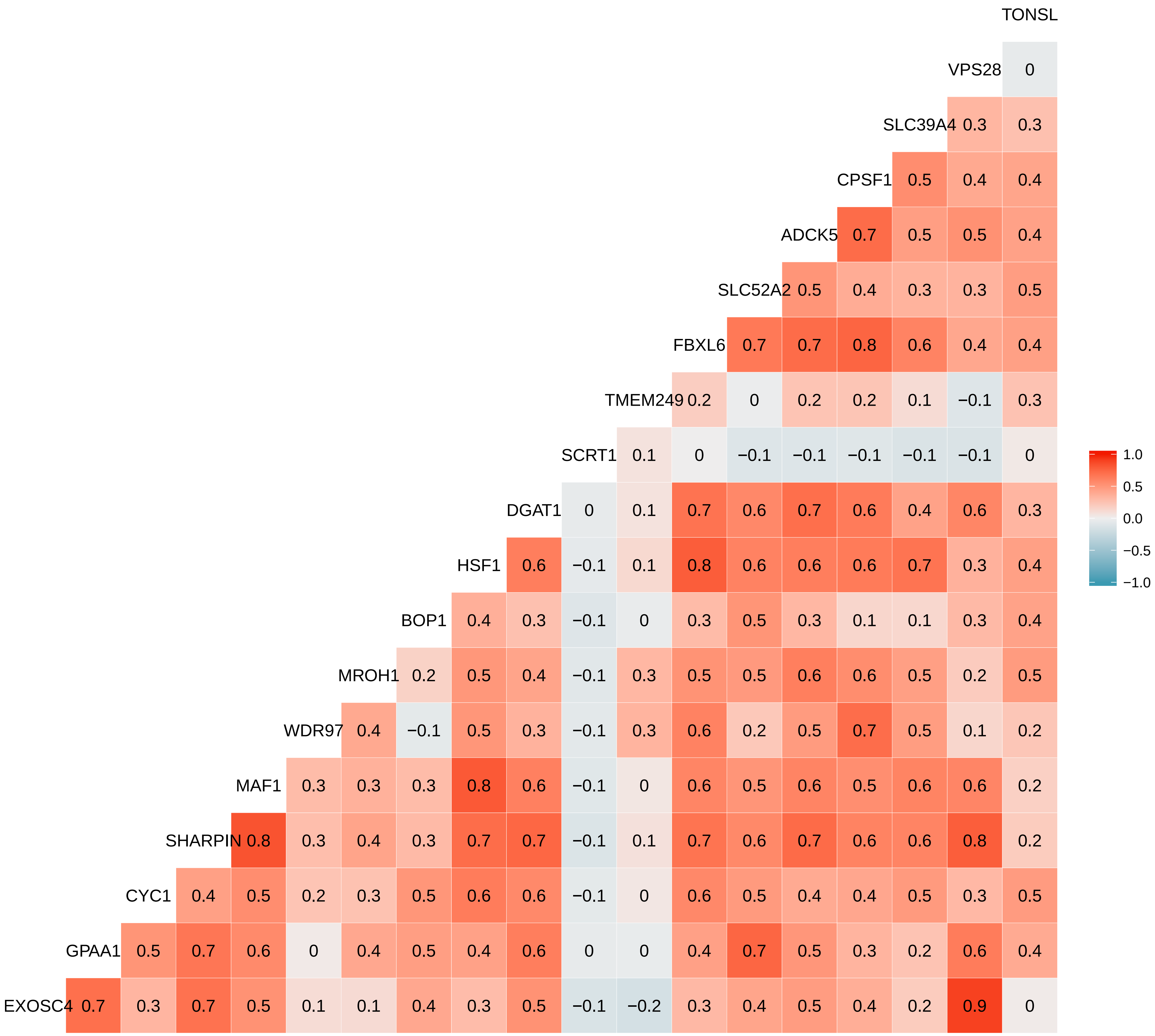

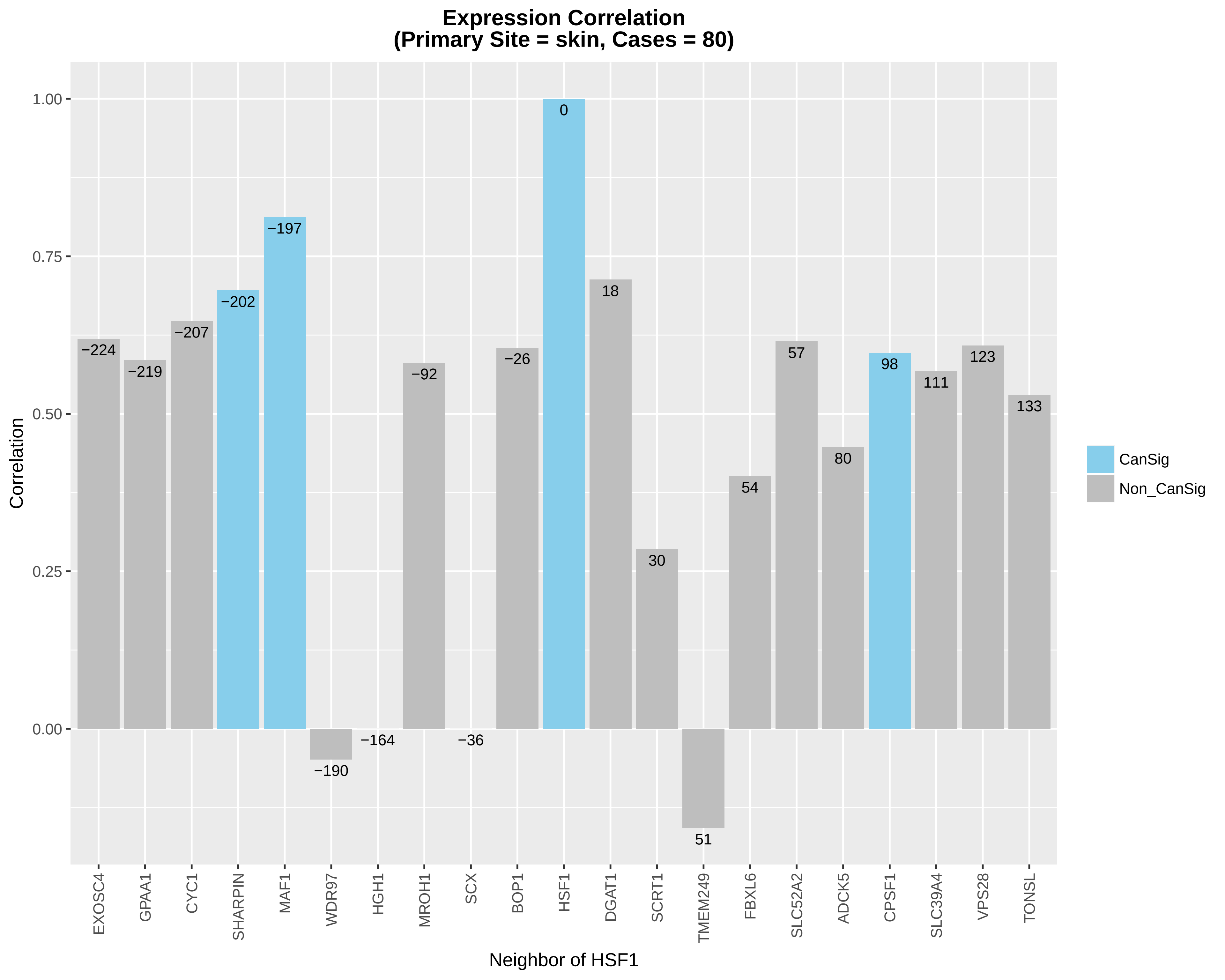

Expression Correlation
(Primary Site = skin, Cases = 80)
Correlation
1.00
0.75
0.50
0.25
0.00
−224
−219
−207
−202
−197
−190
−164
−92
−36
−26
0
18
30
51
54
57
80
98
111
123
133
EXOSC4
GPAA1
CYC1
SHARPIN
MAF1
WDR97
HGH1
MROH1
SCX
BOP1
HSF1
DGAT1
SCRT1
TMEM249
FBXL6
SLC52A2
ADCK5
CPSF1
SLC39A4
VPS28
TONSL
Neighbor of HSF1
CanSig
Non_CanSig

Expression Correlation Matrix
(Primary Site = skin, Cases = 80)

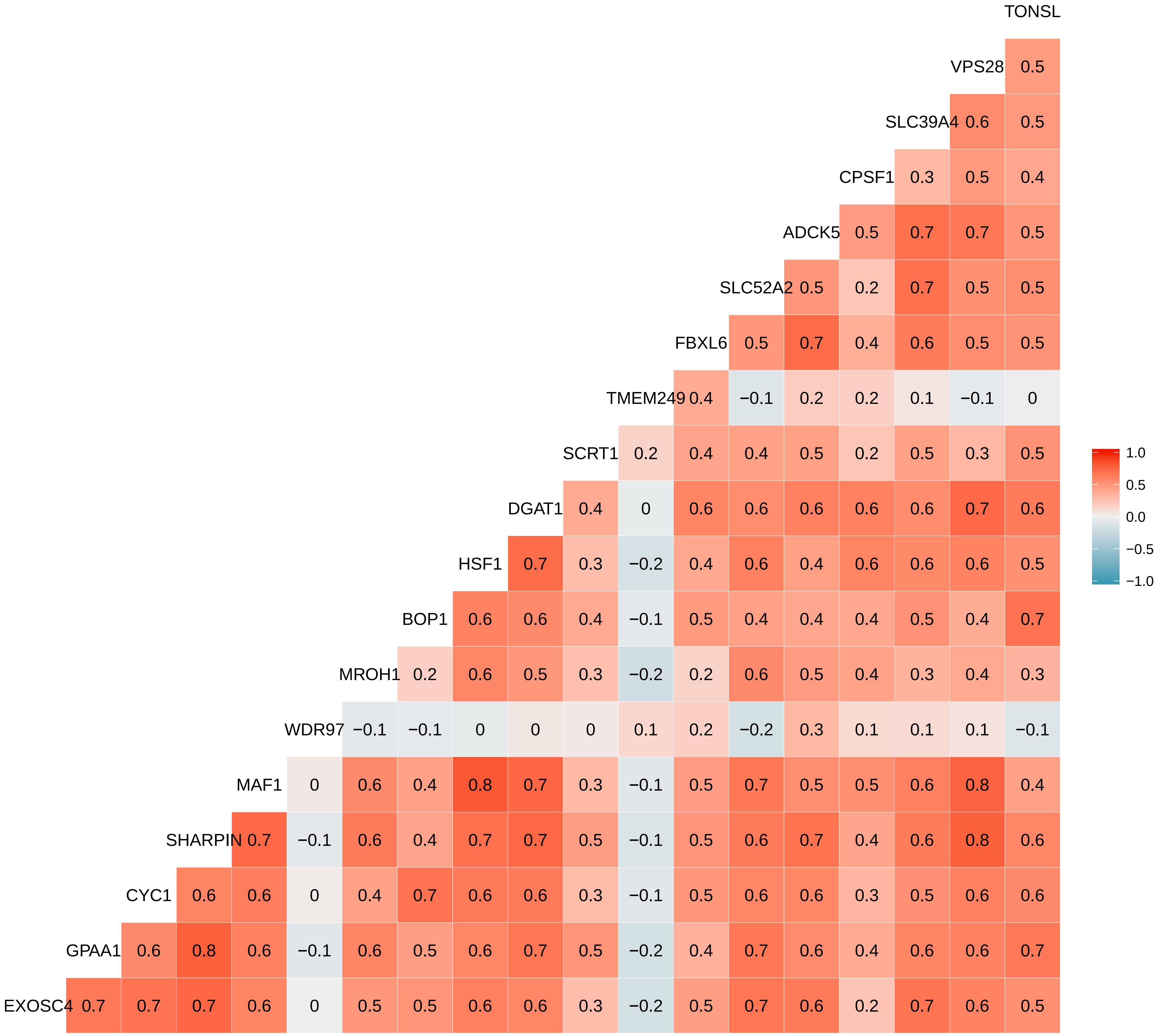

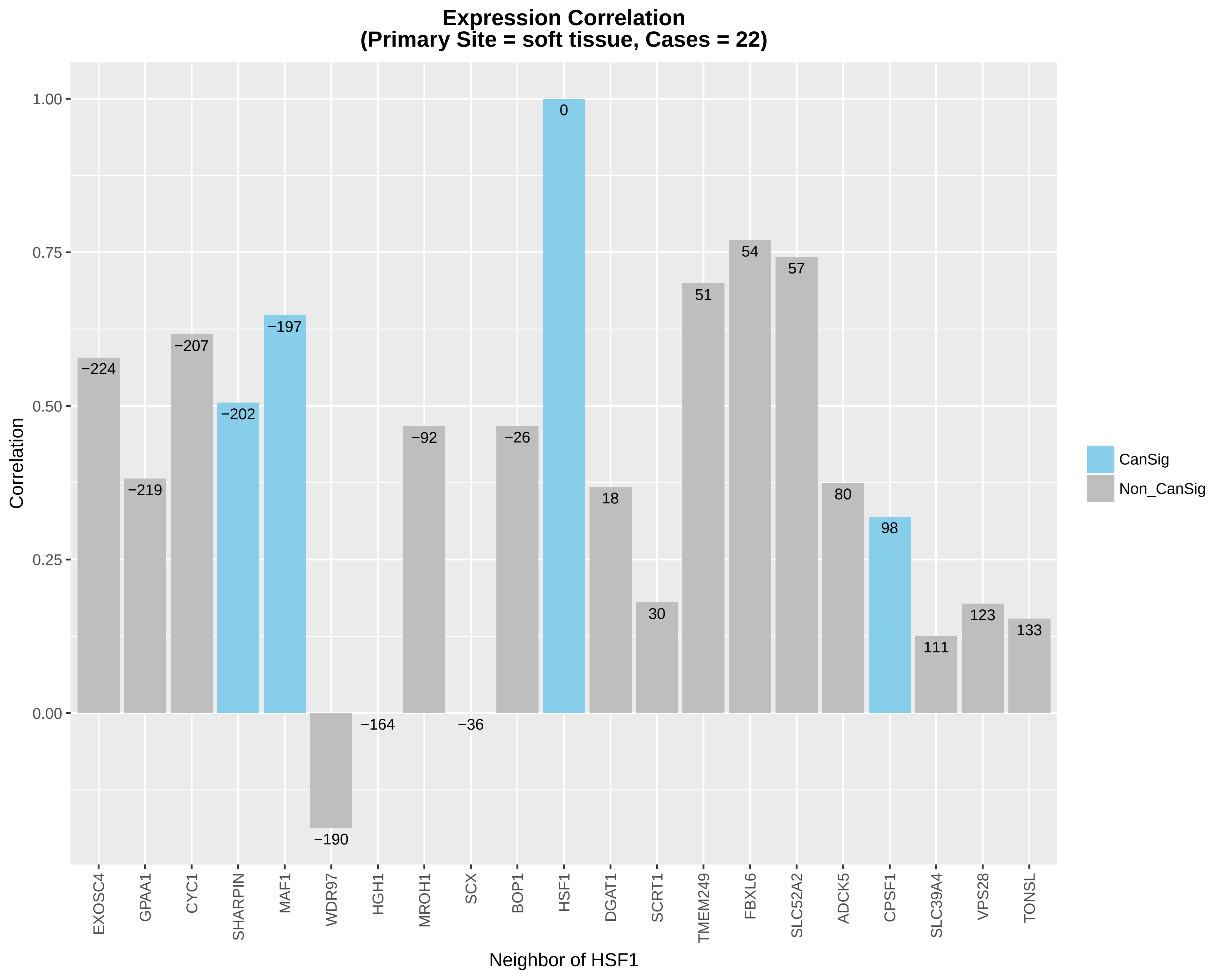
Expression Correlation
(Primary Site = soft tissue, Cases = 22)
Correlation
1.00
0.75
0.50
0.25
0.00
Neighbor of HSF1
EXOSC4
GPAA1
CYC1
SHARPIN
MAF1
WDR97
HGH1
MROH1
SCX
BOP1
HSF1
DGAT1
SCRT1
TMEM249
FBXL6
SLC52A2
ADCK5
CPSF1
SLC39A4
VPS28
TONSL
−224
−219
−207
−202
−197
−190
−164
−92
−36
−26
0
18
30
51
54
57
80
98
111
123
133
CanSig
Non_CanSig

Expression Correlation Matrix
(Primary Site = soft tissue, Cases = 22)

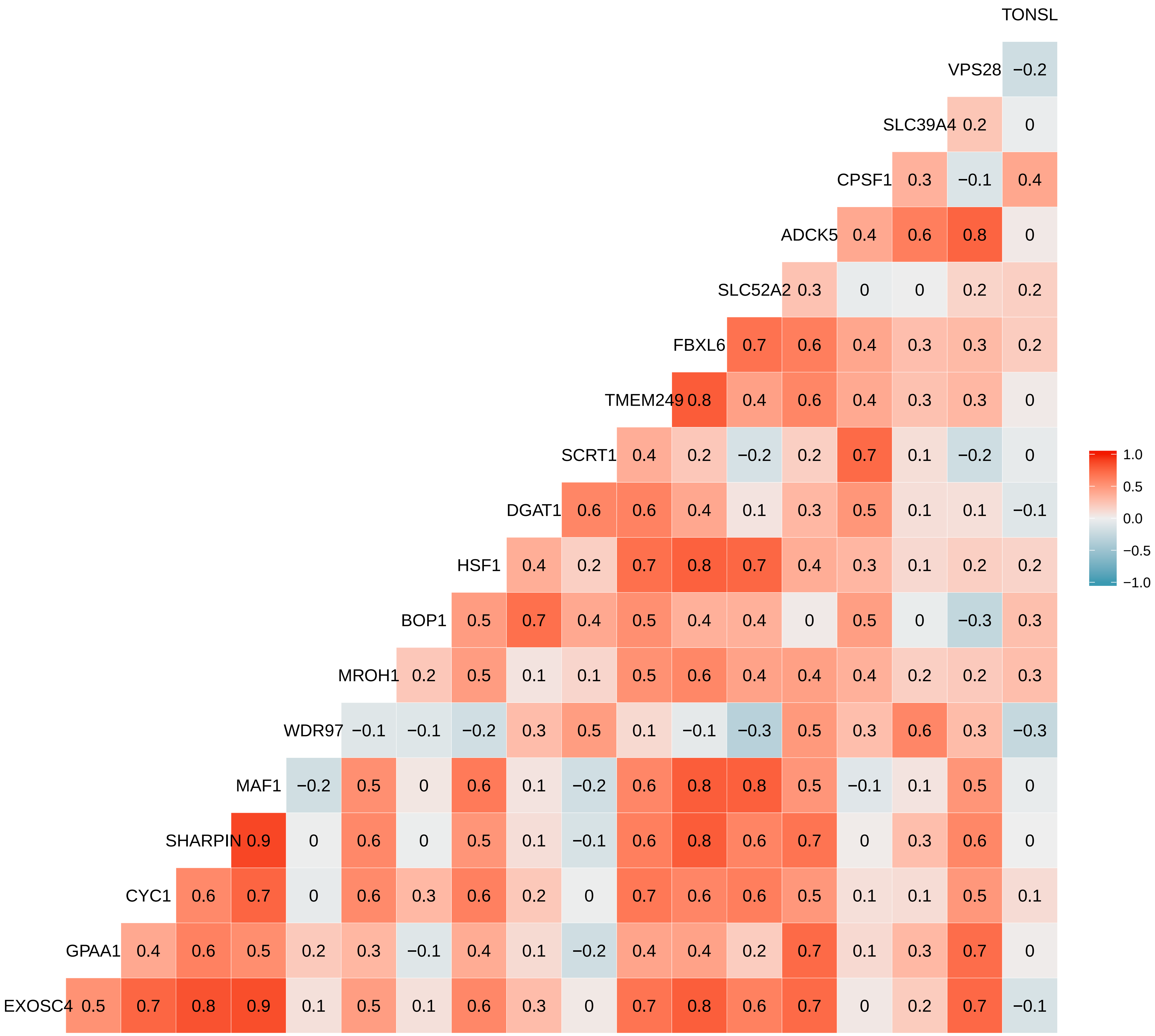

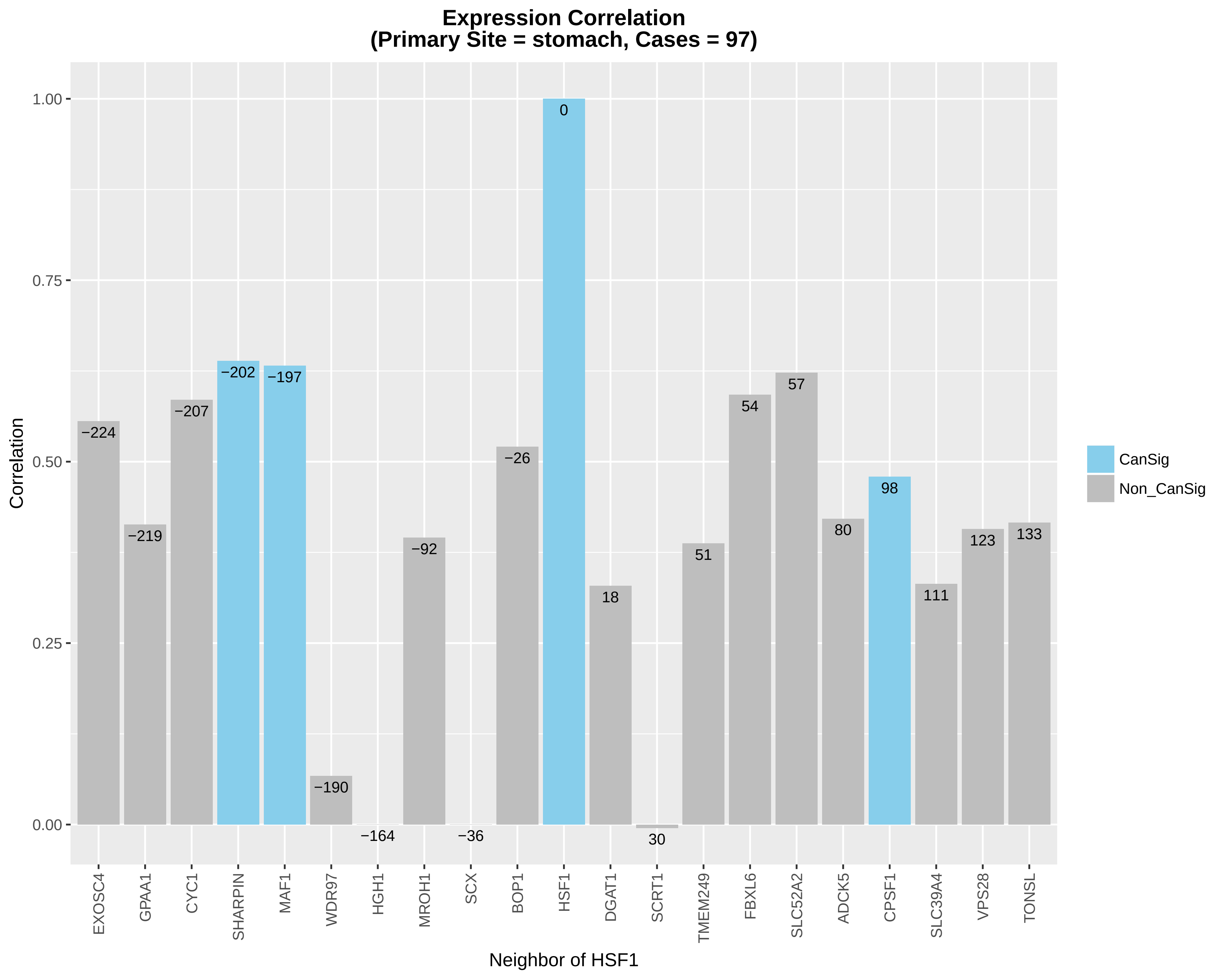

Expression Correlation
(Primary Site = stomach, Cases = 97)
Correlation
1.00
0.75
0.50
0.25
0.00
−224
−219
−207
−202
−197
−190
−164
−92
−36
−26
0
18
30
51
54
57
80
98
111
123
133
EXOSC4
GPAA1
CYC1
SHARPIN
MAF1
WDR97
HGH1
MROH1
SCX
BOP1
HSF1
DGAT1
SCRT1
TMEM249
FBXL6
SLC52A2
ADCK5
CPSF1
SLC39A4
VPS28
TONSL
Neighbor of HSF1
CanSig
Non_CanSig

Expression Correlation Matrix
(Primary Site = stomach, Cases = 97)

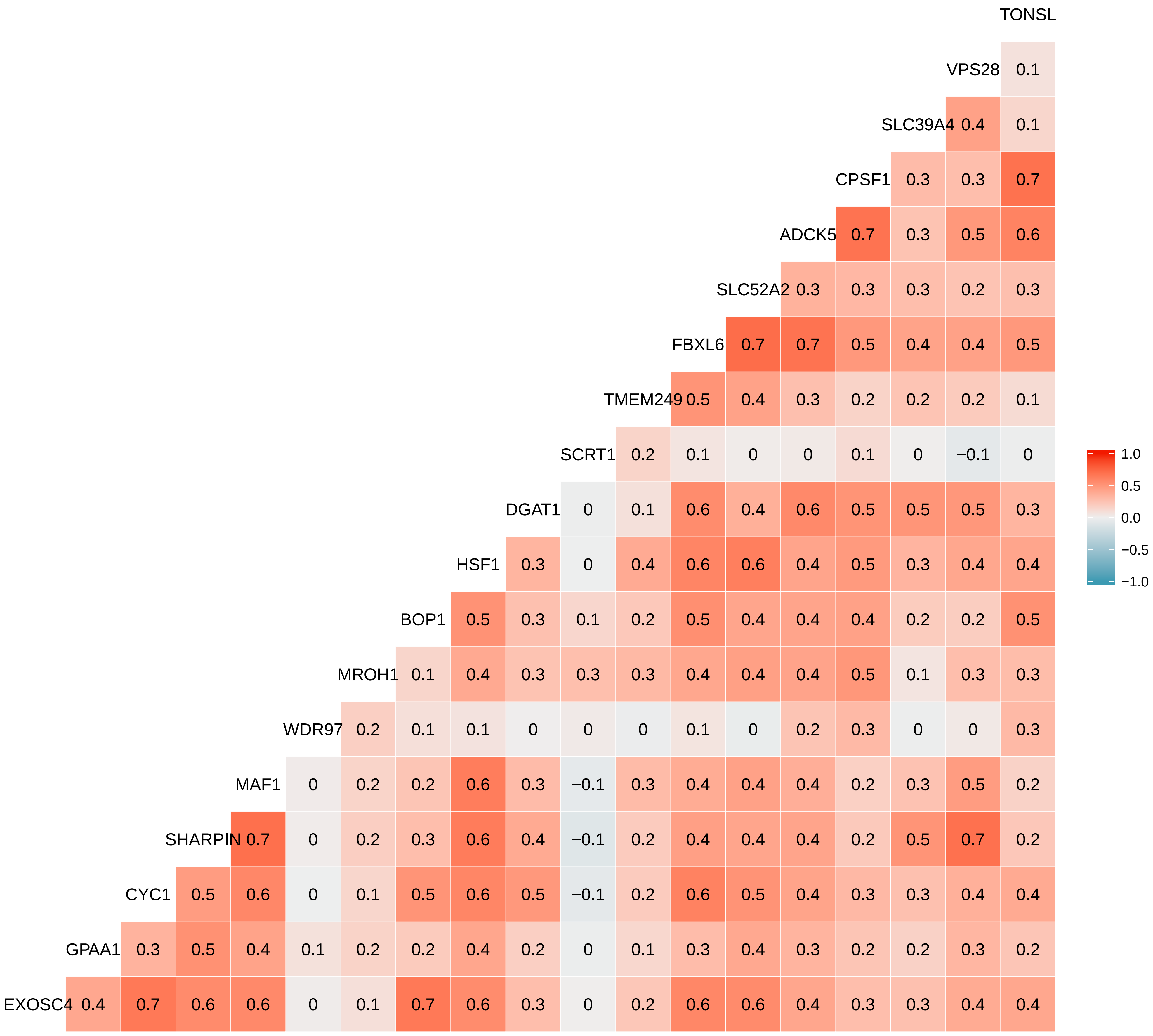

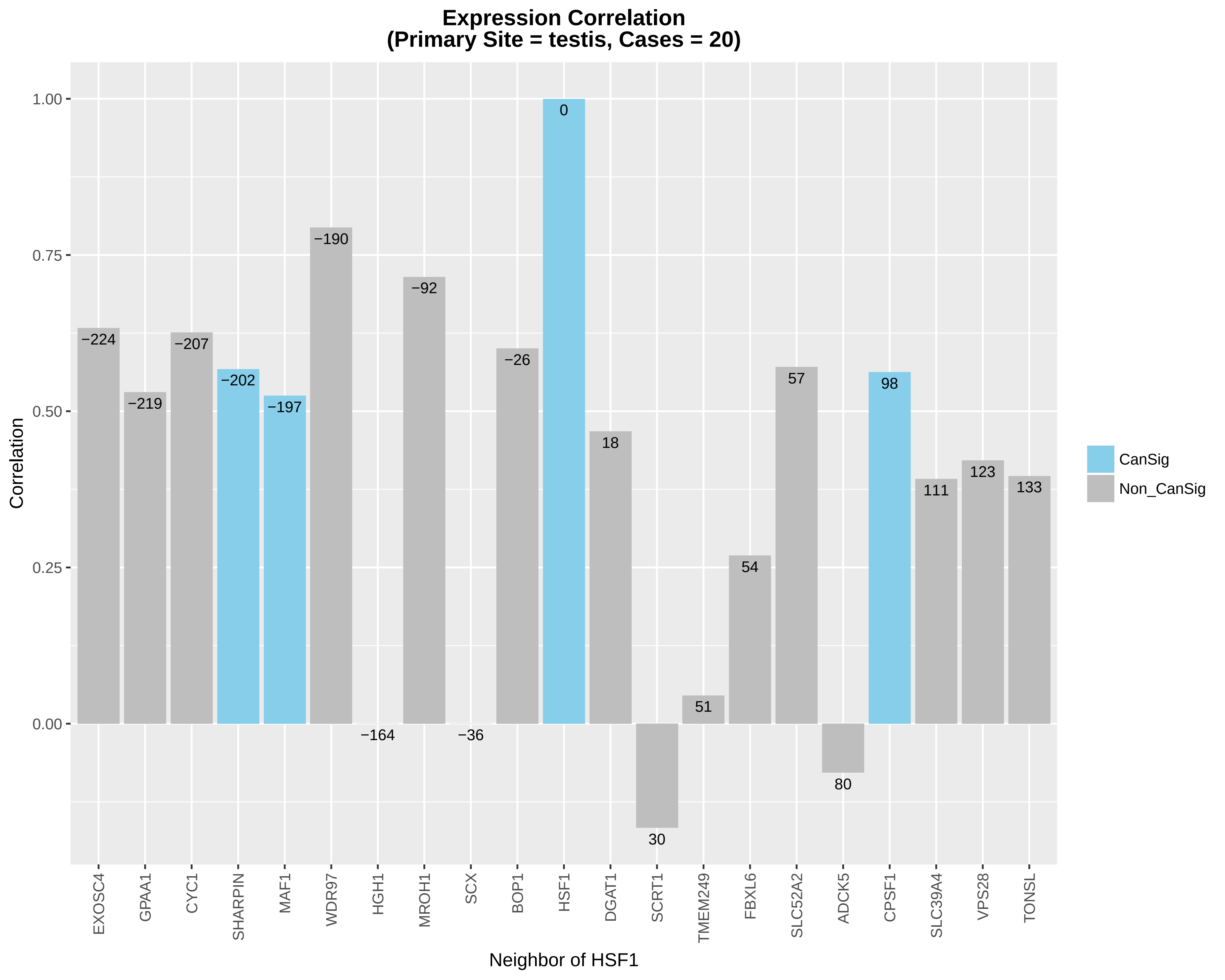

Expression Correlation
(Primary Site = testis, Cases = 20)
Correlation
1.00
0.75
0.50
0.25
0.00
−224
−219
−207
−202
−197
−190
−164
−92
−36
−26
0
18
30
51
54
57
80
98
111
123
133
EXOSC4
GPAA1
CYC1
SHARPIN
MAF1
WDR97
HGH1
MROH1
SCX
BOP1
HSF1
DGAT1
SCRT1
TMEM249
FBXL6
SLC52A2
ADCK5
CPSF1
SLC39A4
VPS28
TONSL
Neighbor of HSF1
CanSig
Non_CanSig

Expression Correlation Matrix
(Primary Site = testis, Cases = 20)

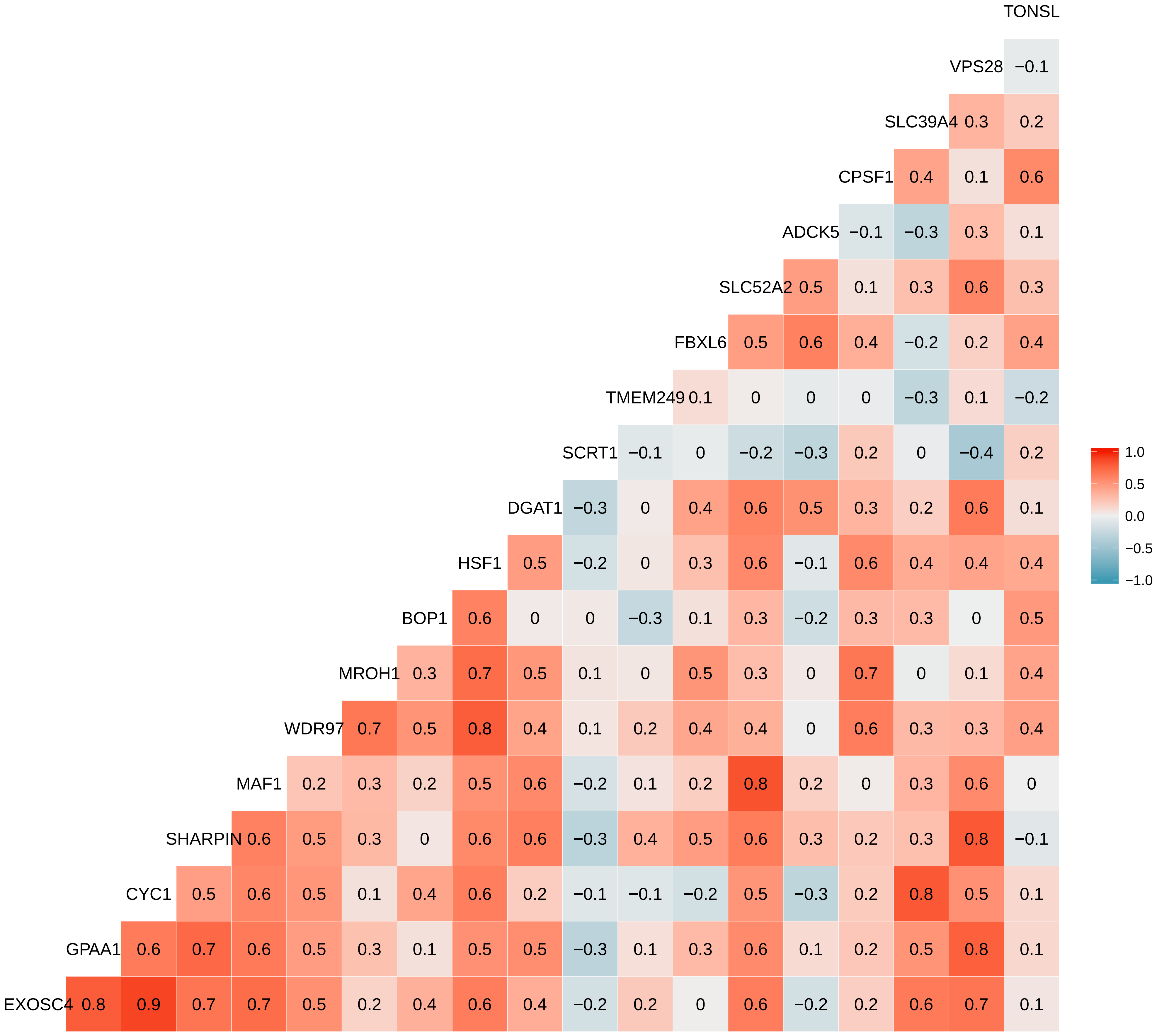

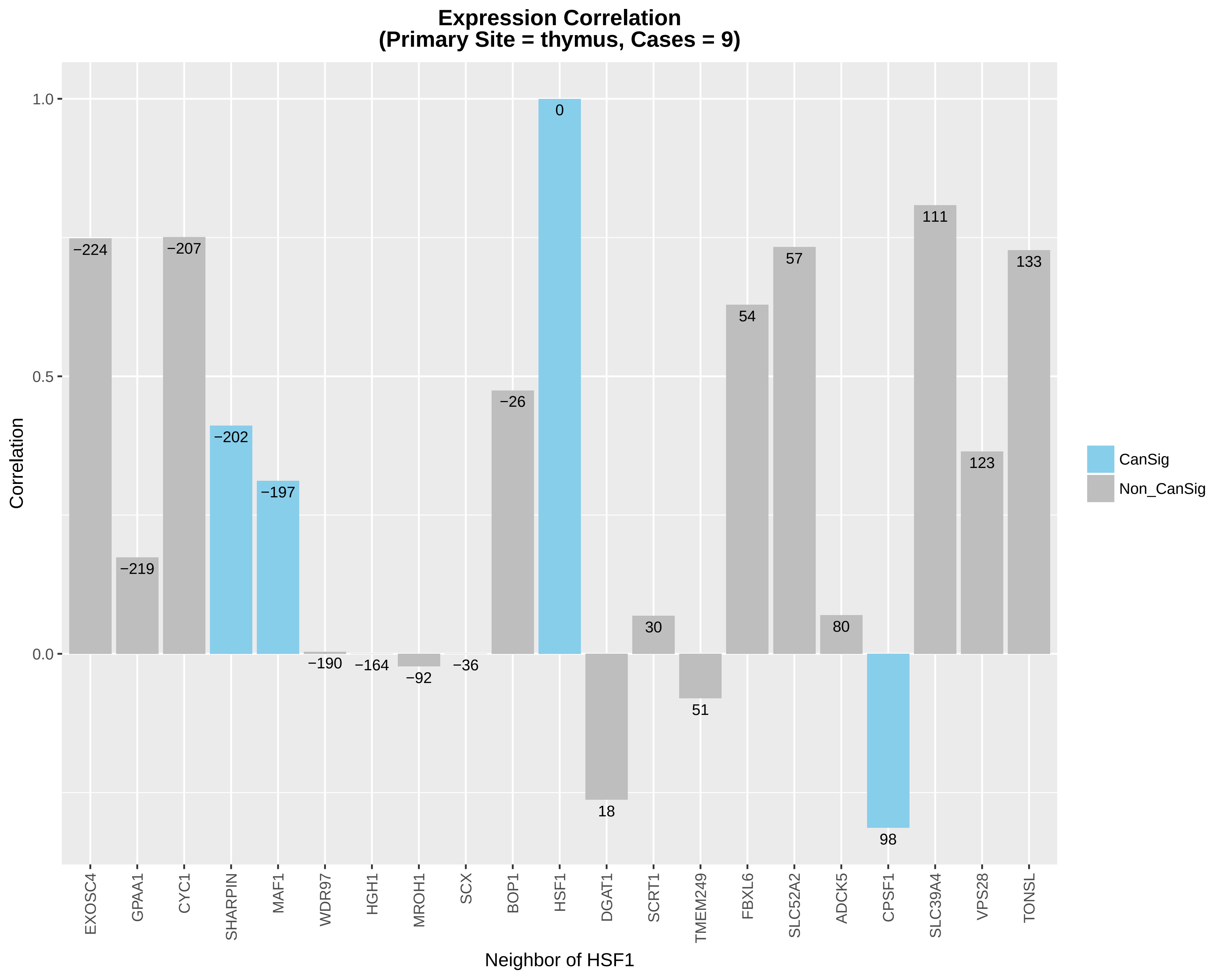

Expression Correlation
(Primary Site = thymus, Cases = 9)
Correlation
Neighbor of HSF1
1.0
0.5
0.0
−224
−219
−207
−202
−197
−190
−164
−92
−36
−26
0
18
30
51
54
57
80
98
111
123
133
EXOSC4
GPAA1
CYC1
SHARPIN
MAF1
WDR97
HGH1
MROH1
SCX
BOP1
HSF1
DGAT1
SCRT1
TMEM249
FBXL6
SLC52A2
ADCK5
CPSF1
SLC39A4
VPS28
TONSL
CanSig
Non_CanSig

Expression Correlation Matrix
(Primary Site = thymus, Cases = 9)

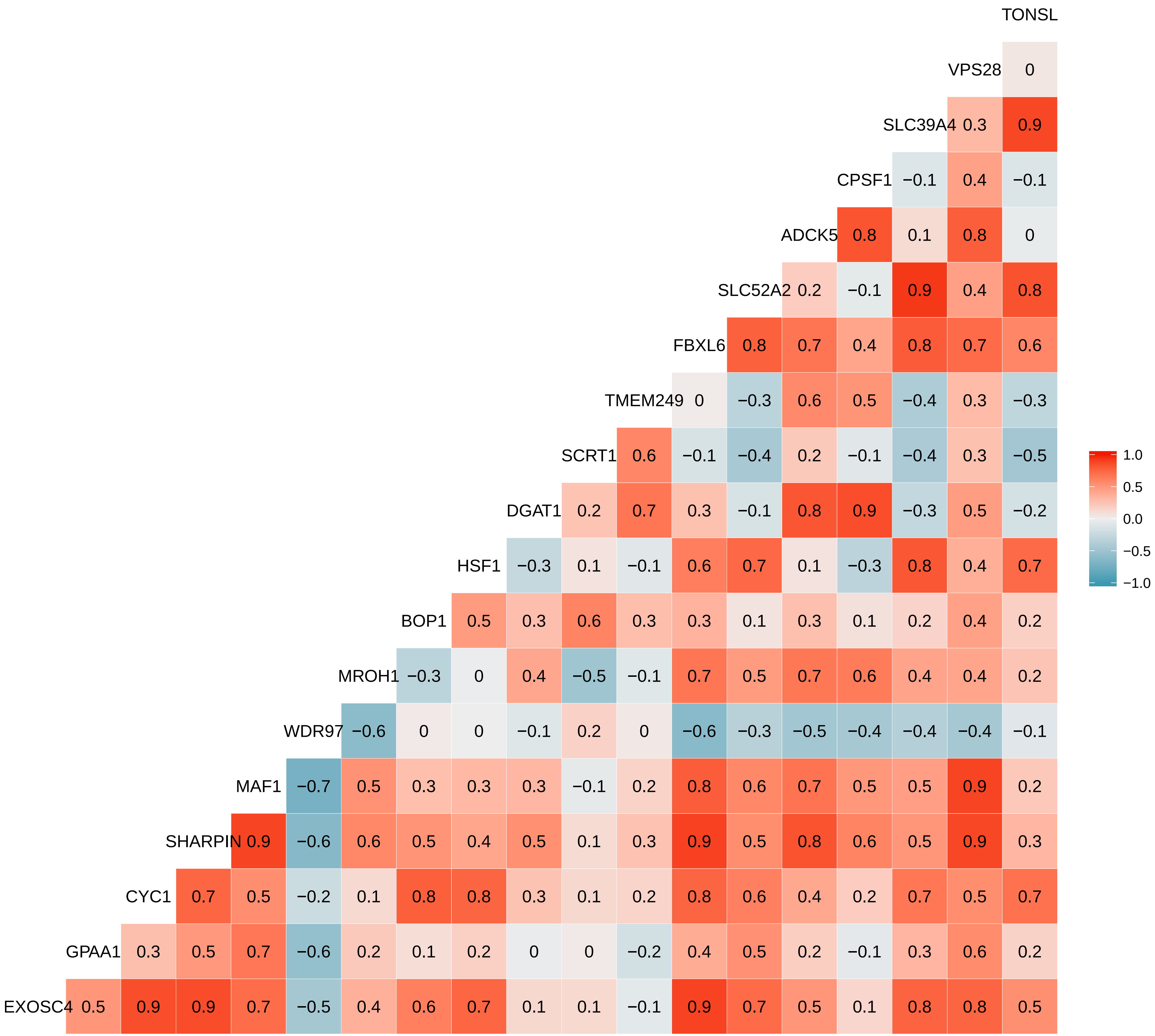

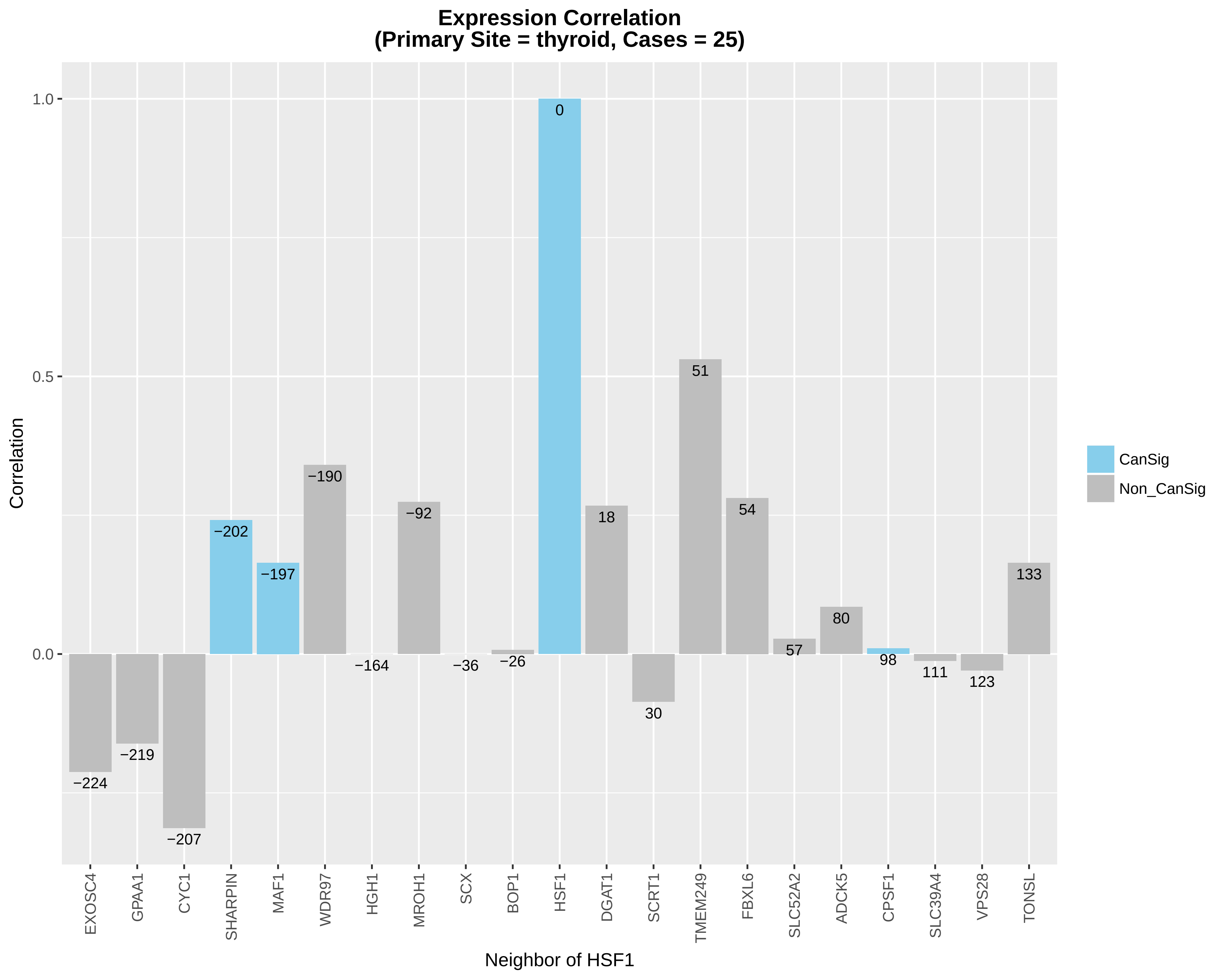

Expression Correlation
(Primary Site = thyroid, Cases = 25)
Correlation
1.0
0.5
0.0
0
51
−190
54
−92
18
−202
−197
133
80
57
98
−164
−36
−26
111
123
30
−224
−219
−207
CanSig
Non_CanSig
EXOSC4
GPAA1
CYC1
SHARPIN
MAF1
WDR97
HGH1
MROH1
SCX
BOP1
HSF1
DGAT1
SCRT1
TMEM249
FBXL6
SLC52A2
ADCK5
CPSF1
SLC39A4
VPS28
TONSL
Neighbor of HSF1

Expression Correlation Matrix
(Primary Site = thyroid, Cases = 25)

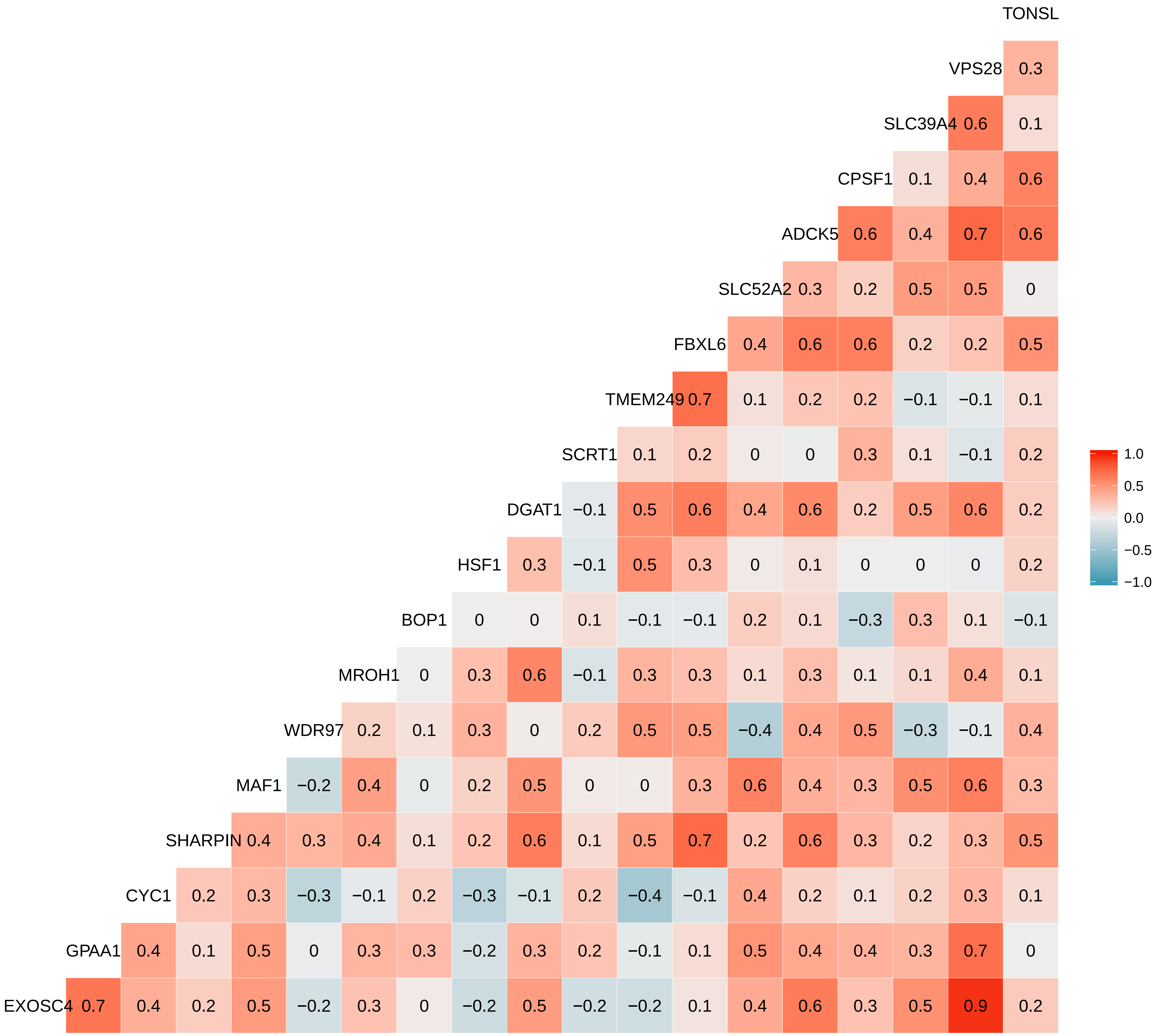

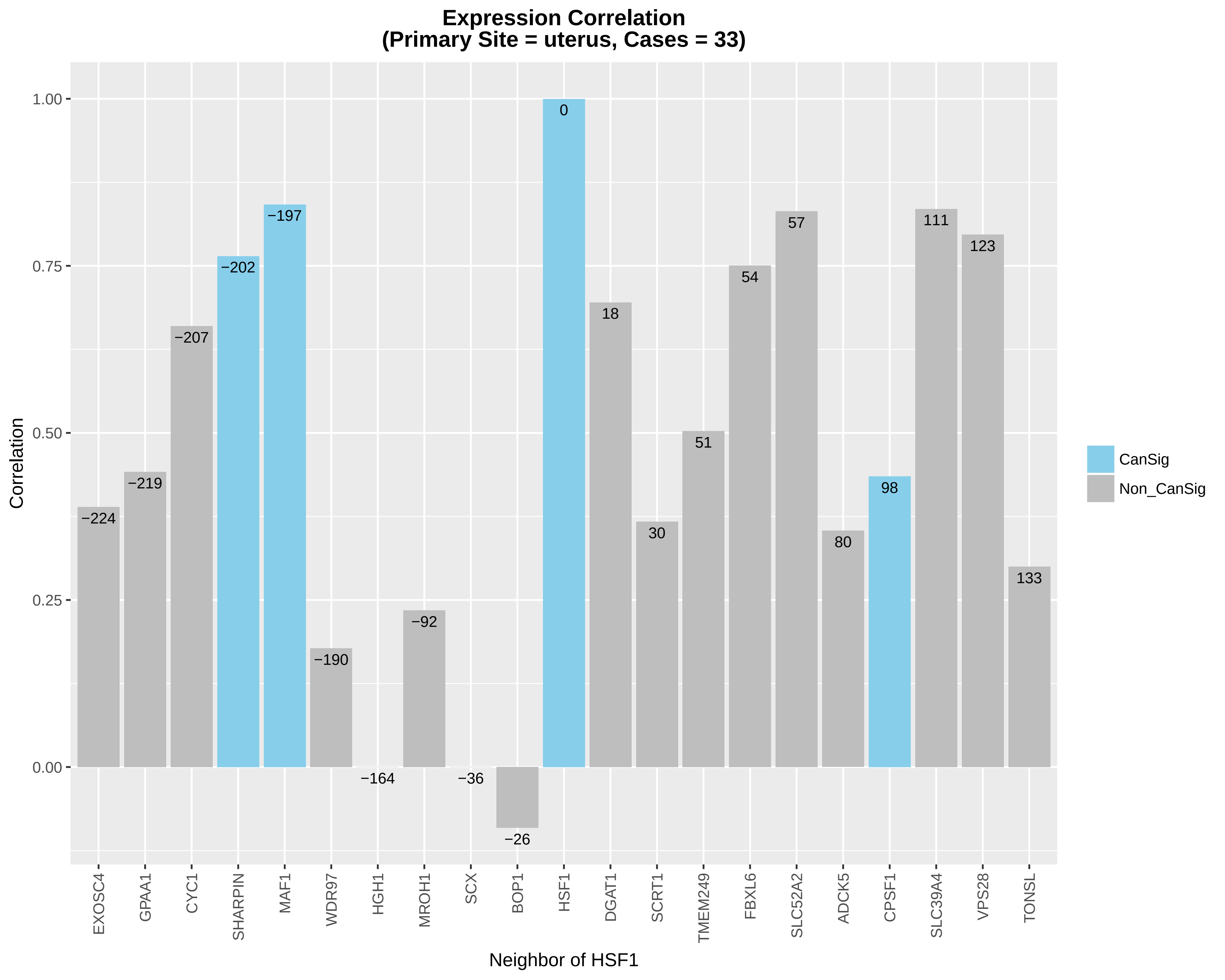
Expression Correlation
(Primary Site = uterus, Cases = 33)
Correlation
1.00
0.75
0.50
0.25
0.00
−224
−219
−207
−202
−197
−190
−164
−92
−36
−26
0
18
30
51
54
57
80
98
111
123
133
EXOSC4
GPAA1
CYC1
SHARPIN
MAF1
WDR97
HGH1
MROH1
SCX
BOP1
HSF1
DGAT1
SCRT1
TMEM249
FBXL6
SLC52A2
ADCK5
CPSF1
SLC39A4
VPS28
TONSL
Neighbor of HSF1
CanSig
Non_CanSig

Expression Correlation Matrix
(Primary Site = uterus, Cases = 33)

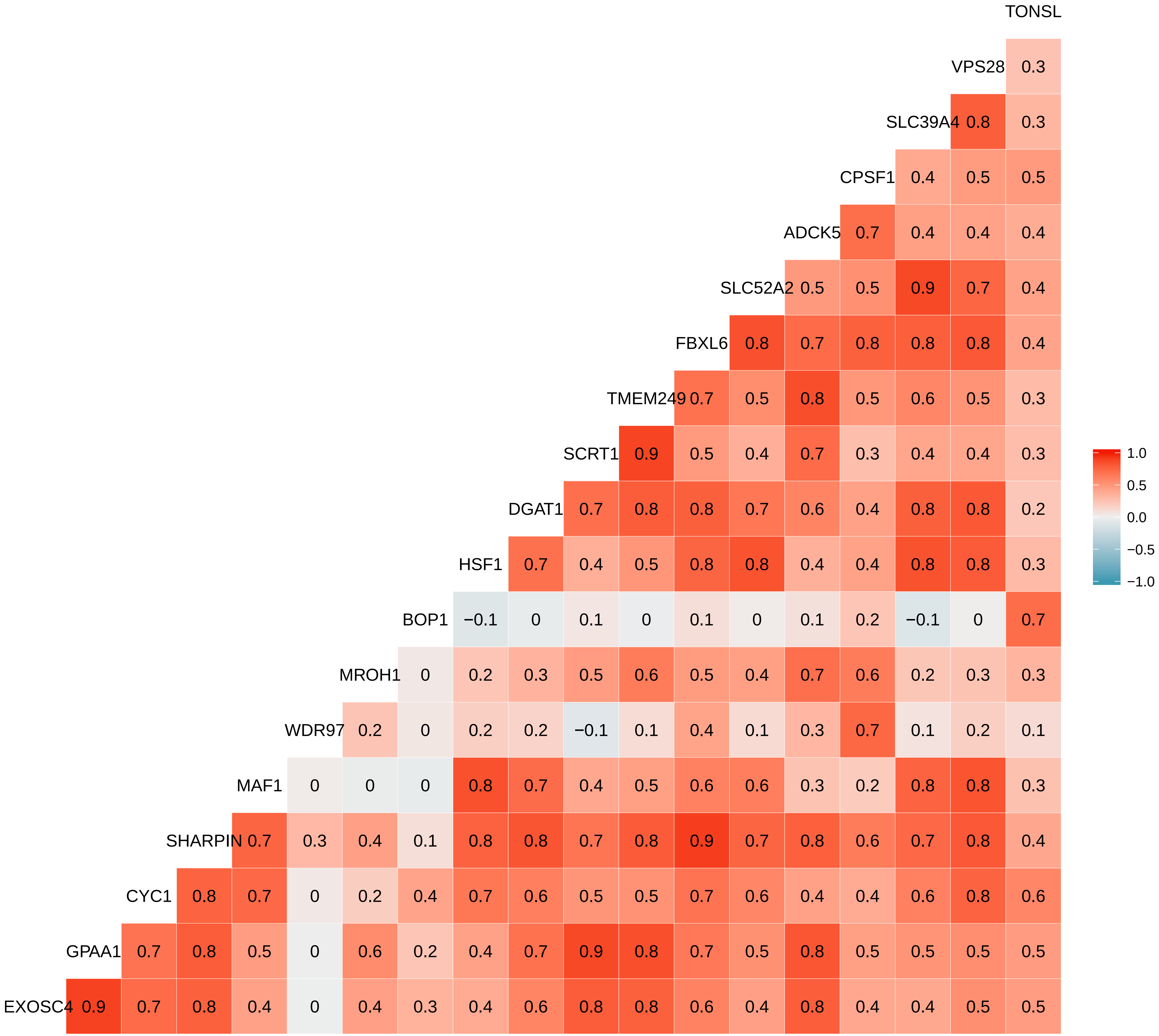

Supplement: Supplementary file 4 — Expression correlation analysis of syntenic genes for each primary tumor sites. (PDF 240 kb) [file 40246_2017_131_MOESM4_ESM.pdf]
